# Rhodium(I)-Catalyzed Annulation of Bicyclo[1.1.0]butyl-Substituted Dihydroquinolines and Dihydropyridines

**DOI:** 10.1021/jacs.4c04081

**Published:** 2024-05-20

**Authors:** Matteo Borgini, Qi-Nan Huang, Pan-Pan Chen, Steven J. Geib, K. N. Houk, Peter Wipf

**Affiliations:** # Department of Chemistry, University of Pittsburgh, Pittsburgh Pennsylvania 15260, United States; □ Department of Chemistry and Biochemistry, University of California, Los Angeles, California 90095, United States; ‡ Department of Chemistry and Biochemistry, Augusta University, Augusta, Georgia 30912, United States; † College of Chemistry and Chemical Engineering, Hunan University, Changsha, Hunan 410082, People’s Republic of China

## Abstract

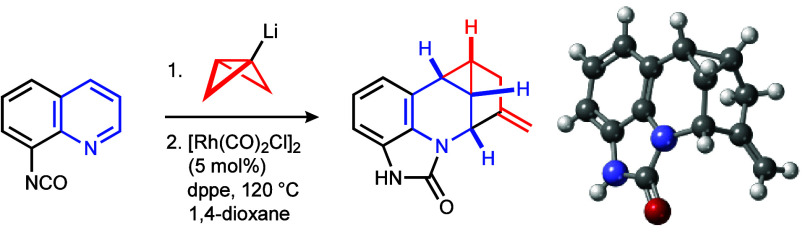

Bicyclo[1.1.0]butane-containing compounds feature a unique chemical reactivity, trigger “strain-release” reaction cascades, and provide novel scaffolds with considerable utility in the drug discovery field. We report the synthesis of new bicyclo[1.1.0]butane-linked heterocycles by a nucleophilic addition of bicyclo[1.1.0]butyl anions to 8-isocyanatoquinoline, or, alternatively, iminium cations derived from quinolines and pyridines. The resulting bicyclo[1.1.0]butanes are converted with high regioselectivity to unprecedented bridged heterocycles in a rhodium(I)-catalyzed annulative rearrangement. The addition/rearrangement process tolerates a surprisingly large range of functional groups. Subsequent chemo- and stereoselective synthetic transformations of urea, alkene, cyclopropane, and aniline moieties of the 1-methylene-5-azacyclopropa[*cd*]indene scaffolds provide several additional new heterocyclic building blocks. X-ray structure-validated quantum mechanical DFT calculations of the reaction pathway indicate the intermediacy of rhodium carbenoid and metallocyclobutane species.

The release of molecular strain energy as a driving force in chemical transformations, often referred to as “strain-release” reactions,^1^ has led to unprecedented chemical transformations that provide access to novel scaffolds of interest to medicinal and material chemists.^2,3^ Bicyclo[1.1.0]butanes (BCBs) are one of the most strained (60–68 kcal/mol), yet readily isolable carbocycles and rank among the most versatile strain-release reagents. In unsubstituted BCBs, the bridgehead and lateral C–C bonds have similar lengths of 1.46 to 1.50 Å and are spatially organized in a signature “butterfly” geometry where the two “wings” are separated by a 123° angle. As a consequence of this conformation, the hybridization of the two bridgehead carbon atoms is dominated by high p- and π-character.^4^ Consequently, BCBs react at the bridgehead C–C bond with a broad range of nucleophiles, electrophiles, radicals, π-systems, and carbenes, demonstrating the σ/π-bond ambiguity of this bond. In the last five years, the study of BCB-containing molecules has surged, and new applications in medicinal chemistry as warheads for covalent inhibition, bioisosters of *ortho*- and *meta*-substituted benzenes, and chemical probes have been reported.^5^

BCBs can be synthesized by transannular cyclizations,^6−8^ cyclopropanations,^9,10^ and side-chain cyclizations that can be further divided into epoxysulfone-based^11−14^ and dibromocyclopropane-based routes. In the latter method, dibromocyclopropane is used as a precursor for a bromide-substituted BCB (BCB-Br) by treatment with methyllithium. The unstable and volatile BCB-Br is then reacted with *t*-BuLi for a Li–Br exchange to form bicyclo[1.1.0]butyllithium (BCB-Li).^5^ BCB-Li can be further treated with freshly prepared MgBr_2_·Et_2_O or MgCl_2_·LiCl to form the more selective bicyclo[1.1.0]butyl magnesium bromide (BCB-MgBr) and bicyclo[1.1.0]butyl magnesium chloride–lithium chloride (BCB-MgCl·LiCl) reagents, respectively.^15^ Another practical method to generate BCB-Li or BCB-MgCl·LiCl consists of the treatment of the bench-stable 1-(*p*-tolylsulfinyl)bicyclo[1.1.0]butane (BCB-sulfoxide) with *t*-BuLi or *i*-PrMgCl·LiCl, respectively.^15^ These reactive organometallic reagents can be trapped with suitable electrophiles to generate BCB-containing amines,^15−18^ alcohols,^18^ sulfoxides,^19−21^ esters,^22^ boronates,^15^ ketones,^23^ and amides^23^ (Scheme 1A). After *N*- or *O*-allylation of imine or aldehyde addition products, the resulting bicyclo[1.1.0]butylalkylamines and -ethers undergo rhodium(I)-catalyzed cycloisomerizations to yield cyclopropane-fused pyrrolidines, azepines, furans, and oxepanes in high stereo- and regiocontrol (Scheme 1B).^18,24^ Herein, we report the trapping of BCB-Li and BCB-MgCl reagents with quinolines **1** or iminium salts **3** derived from quinoline, pyridine, and related heterocycles to generate BCB-containing dihydroquinolines **2** and dihydropyridines **4**. The rhodium(I)-catalyzed rearrangement of these new addition products allows access to the novel 1-methylene-5-azacyclopropa[*cd*]indene scaffolds **5** and **6** (Scheme 1C).^25^

**Scheme 1:**
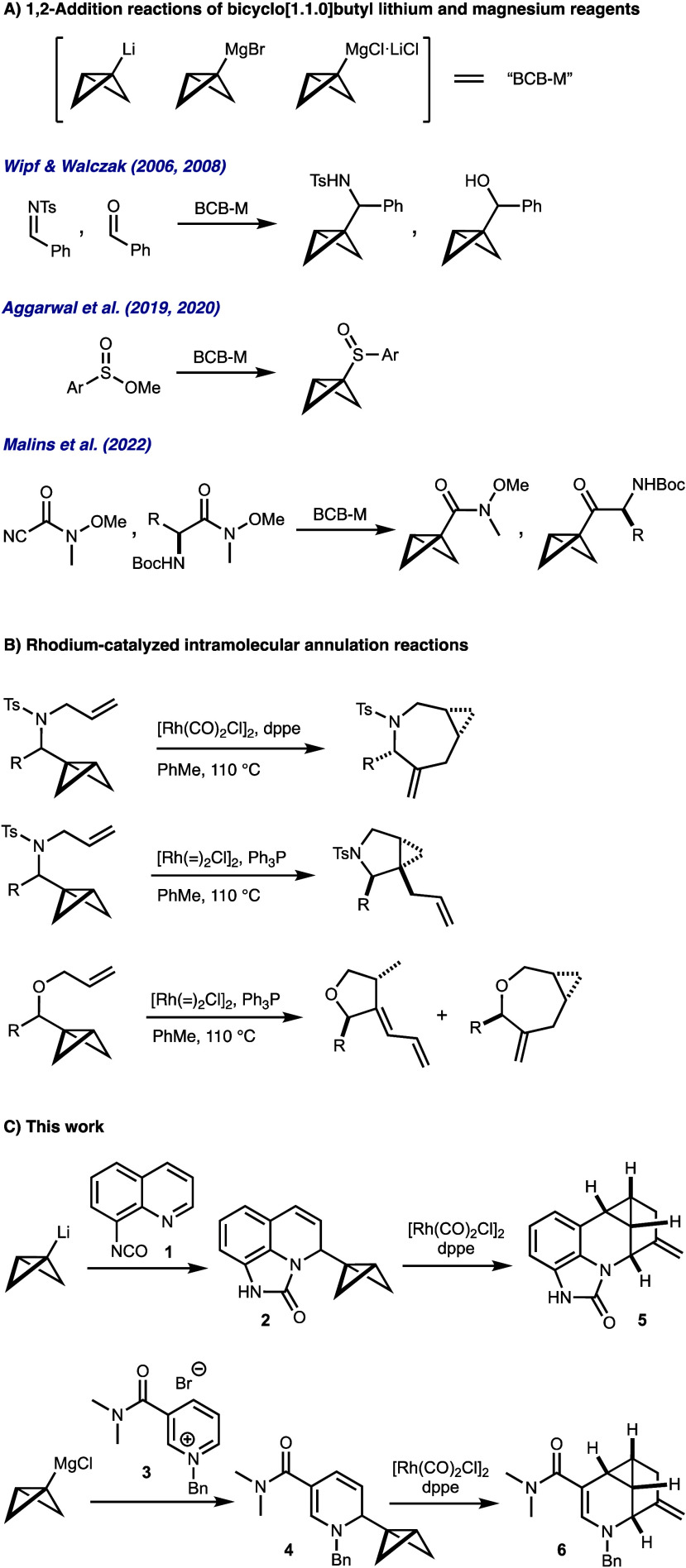
Selected Transformations of BCB-Containing Reagents

## Reaction Optimization

**Scheme 2:**
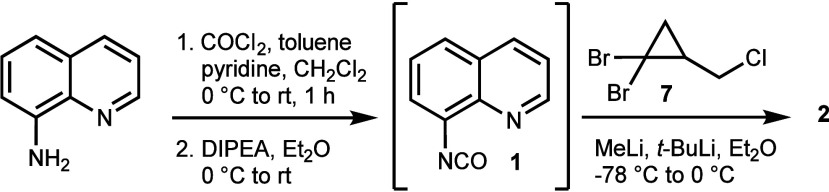
Preparation of 4*H*-Imidazo[4,5,1-*ij*]quinolin-2(1*H*)-one **2** from *in Situ* Prepared Isocyanate **1** and BCB-Li Reagent Prepared from Trihalocyclopropane **7**

**Scheme 3:**
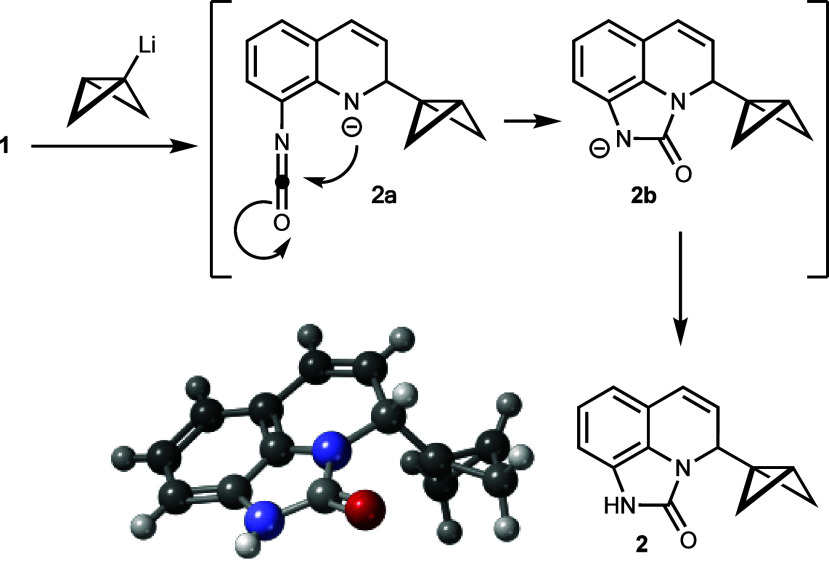
Formation of Tricyclic Urea **2** by an Intramolecular Trapping of the Amide Anion Resulting from BCB-Li Addition to Quinoline **1** and X-ray Structure of **2** (CCDC 2340745)

We first explored the rhodium(I)-catalyzed rearrangement of the bench-stable BCB-dihydroquinoline **2**, which was synthetically accessible from 8-aminoquinoline via the corresponding isocyanate **1** (Scheme 2). Product formation involved the *in situ* cyclization of intermediate **2a**, obtained after the addition of the BCB-Li reagent to **1**, which generated the unprecedented tricyclic urea **2b**, most likely by the addition of the dihydroquinoline amide anion to the isocyanate (Scheme 3).

The structure of **2** was secured by an X-ray analysis. For the conversion of **2** into pentacycle **5**, we screened optimal ligand and solvent combinations (Table 1; see SI, Table S1, for a complete set of conditions). In the presence of 10 mol % of triphenylphosphine and 5 mol % of the dimeric rhodium precatalyst [Rh(CO)_2_Cl]_2_ in toluene at 120 °C, 18% of the annulation product **5** was obtained based on NMR analysis of the reaction mixture (entry 1). The structure of **5** was confirmed by an X-ray analysis. While the yield was essentially unchanged in the presence of the bidentate ligand dppp (entry 2), using dppe increased it to 69% (entry 3). A switch in solvent to 1,4-dioxane and the use of tribenzylphosphine, dcpe, dppb, or dfppe provided low to moderate yields of 24–51% (entries 4–7). However, the combination of 1,4-dioxane as a solvent and dppe as a ligand at 120 °C for 30 min significantly increased the yield to 80% (77% isolated yield, entry 8). Under these conditions, the Rh(I)-catalyzed rearrangement could also be performed on a gram-scale, providing product **5** in 85% yield in the presence of 7.5 mol % catalyst and 15 mol % ligand. In contrast, lowering the reaction temperature to 80–90 °C and extending the reaction time to 60 min reduced the yield to 46–53% (entries 9 and 10). Accordingly, 1,4-dioxane/dppe/30 min/120 °C were selected for further investigations.

**Table 1:**
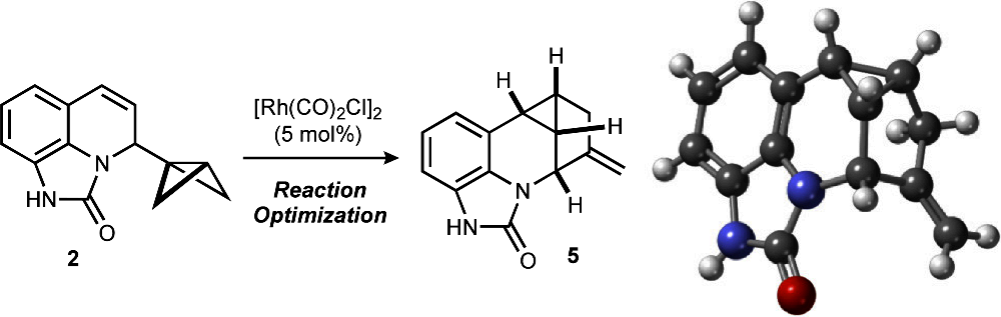
Optimization of the [Rh(CO)_2_Cl]_2_-Catalyzed Conversion of **2** to Pentacyclic **5** (CCDC 2340746)^a^

entry	ligand^b^	solvent	temp	yield^c^
1	PPh_3_	toluene	120 °C	18%
2	dppp	toluene	120 °C	19%
3	dppe	toluene	120 °C	69%
4	PBn_3_	1,4-dioxane	120 °C	36%
5	dcpe	1,4-dioxane	120 °C	39%
6	dppb	1,4-dioxane	120 °C	24%
7	dfppe	1,4-dioxane	120 °C	51%
8	dppe	1,4-dioxane	120 °C	80%; 77%;^d^ 85%^e^
9^f^	dppe	1,4-dioxane	80 °C	53%
10	dppe	1,4-dioxane	90 °C	46%

a Reactions were performed in a sealed microwave vial with a PTFE cap in a degassed 0.05 M solution for 30 min unless otherwise noted.

b 10 mol % ligand.

c NMR yield unless otherwise noted.

d Isolated yield.

e 1 g scale with 7.5 mol % Rh-precatalyst and 15 mol % ligand.

f 60 min reaction time.

## Substrate Scope

**Scheme 4:**
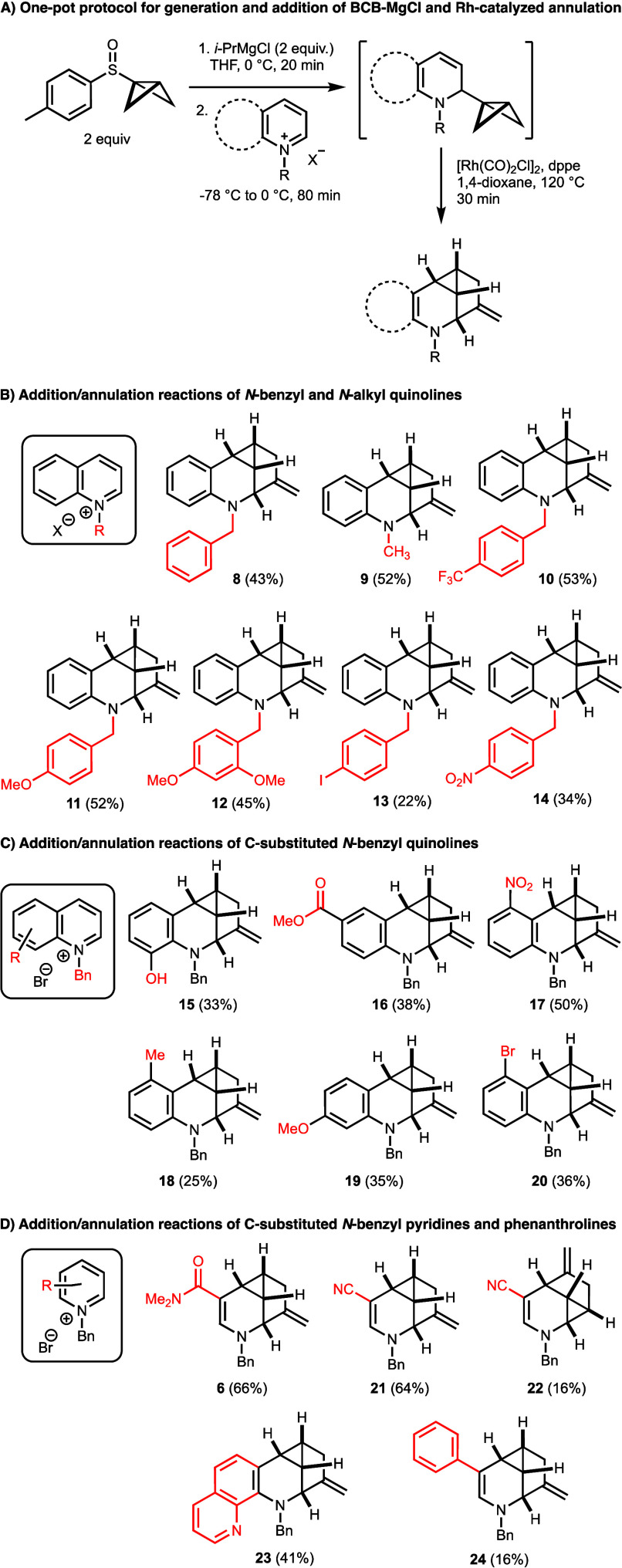
Studies of Reaction Scope with Heterocyclic Substrates: (A) One-Pot/Two-Step Process Using *in Situ* Generated BCB-MgCl; (B) *N*-Benzyl and *N*-Alkyl Quinoline Substrates; (C) Substituted *N*-Benzyl Quinoline Substrates; (D) *N*-Benzyl Pyridine and 1,10-Phenanthroline Substrates Yields are isolated yields over the two reaction steps and are based on quinolinium or pyridinium halides.

Although BCB-urea **2** did not demonstrate any stability issues during purification and storage, we noticed during our investigations of the substrate scope of this transformation that analogs of **2** without *N*-acyl groups were prone to decomposition during purification. Accordingly, we developed a one-pot/two-step process using *in situ* generated BCB-MgCl (Scheme 4A). Treatment of 2 equiv of 1-(*p*-tolylsulfinyl)bicyclo[1.1.0]butane with 2 equiv of isopropyl magnesium chloride followed by 1 equiv of a quaternary quinolinium or pyridinium halide generated the 1,2-adduct, which was not isolated but directly treated with rhodium precatalyst and dppe ligand in 1,4-dioxane at 120 °C for 30 min. This protocol allowed the preparation of a broad range of 1*H*-5-azacyclopropa[*cd*]indenes in moderate to good (16–66%) overall yields from the corresponding quaternary ammonium salts (Scheme 4B–D). Both *N*-benzyl and -alkyl substituents were tolerated, as shown for compounds **8** and **9**, which were obtained in 43% and 52% yield, respectively (Scheme 4B). The presence of electron-withdrawing or -donating groups on the benzyl moiety did not have a significant effect on the reaction yield, consistently providing ca. 50% product, with the exception of iodide **13** and nitro compound **14**, which were nonetheless isolated in 22% and 34% yield, respectively, in spite of their susceptibility to organolithium reagents and the formation of a larger amount of polar byproducts during the chromatographic purifications. Consistently, we also observed a small amount of a byproduct resulting from the Rh-catalyzed cycloisomerization, where the bicyclo[1.1.0]butyl moiety was converted into a buta-1,3-dien-2-yl substituent. These dienes are susceptible to decomposition under the reaction and isolation conditions.

Quite impressively, the two-step reaction process also worked well with C-substituted quinolinium ions, even in the presence of reactive functional groups such as a hydroxy group at the C-8 position and a methyl ester at C-5, providing the desired products **15** and **16** in 33% and 38% yield, respectively (Scheme 4C). Interestingly, the presence of electron-withdrawing substituents facilitated the regioselective addition of BCB-MgCl to the α-carbon of the quinolinium ion and provided significant electronic stabilization of the adduct, such as compound **17**, which was obtained in 50% yield. Electron-donating substituents such as 5-methyl and 7-methoxy groups, in contrast, provided the desired products in lower yields, probably due to corresponding deactivating effects. Finally, the screening of pyridinium and 1,10-phenanthrolinium ions also yielded excellent results with the two-step reaction protocol (Scheme 4D). Products bearing amide or nitrile groups in conjugation with the basic nitrogen, such as **6** (66% yield) and **21** (64% yield), proved to be the most readily formed and isolated. Moreover, when 1-benzyl-3-cyanopyridin-1-ium bromide was used as the electrophile, the regioisomer **22** was obtained in 16% yield as a minor component in addition to **21**, demonstrating that a C-4 BCB adduct also undergoes the Rh-catalyzed annulation reaction. The yield of the phenyl-substituted tetrahydropyridine **24** was decreased due to decomposition of the reactive enamine during chromatography on SiO_2_.

## Further Synthetic Transformations

**Scheme 5:**
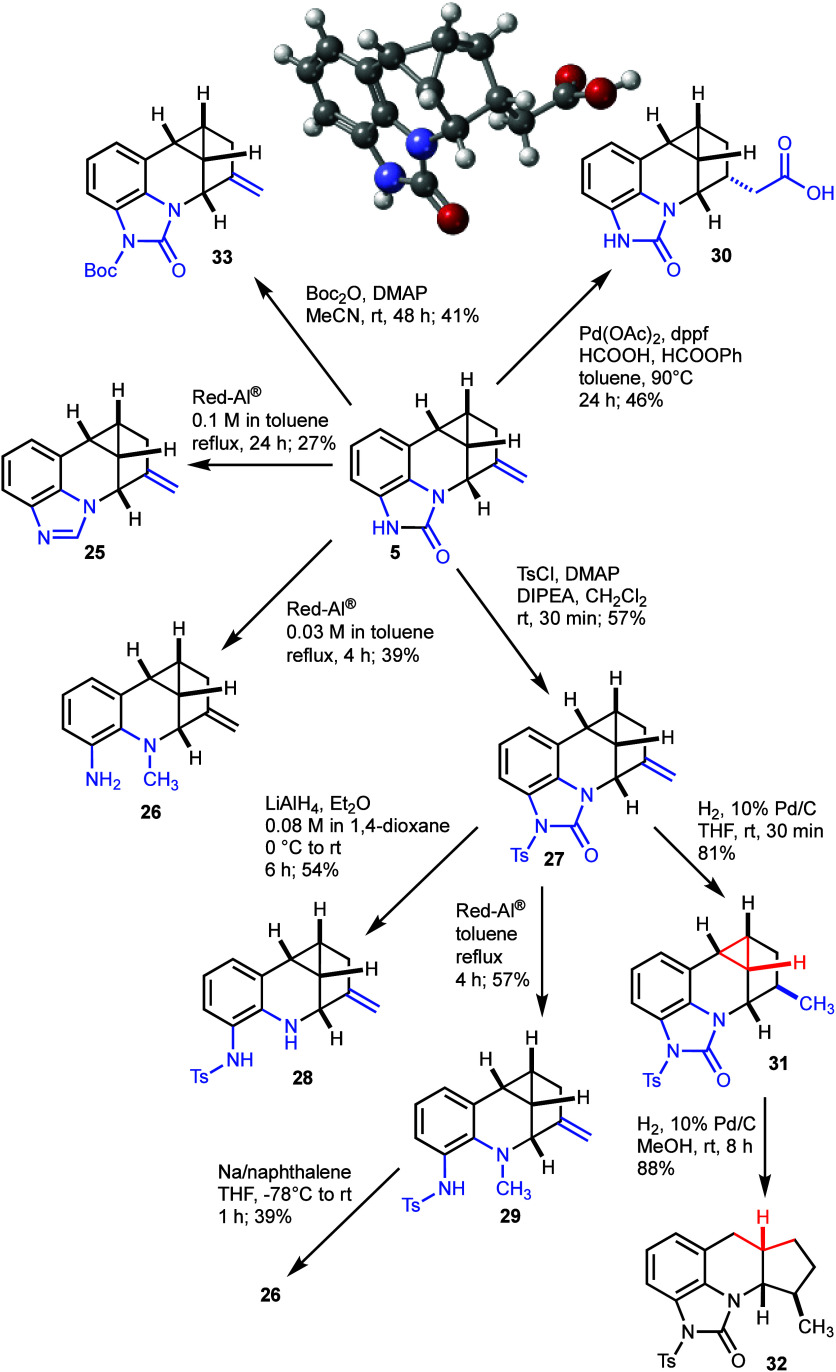
Selective Functional Group Interconversions of Cyclic Urea and Alkene Moieties in **5**, Cyclopropane Ring-Opening of **31**, and X-ray Structure of Acid **30** (CCDC 2341591)

The novel functionality and ring scaffold of cyclic urea **5** inspired us to explore a range of synthetic transformations that provided access to diamines and amino acids as well as scaffold isomers (Scheme 5). A selective reduction with 60% Red-Al proved to be strongly dependent on the concentration of **5** in toluene, providing benzimidazole **25** and diamine **26** in 27% and 39% yield, respectively, at 0.1 M vs 0.03 M concentration in the reaction mixture. The direct formation of an imidazole from an urea under reductive conditions is, to the best of our knowledge, an unprecedented and useful new transformation, in light of the considerable utility of benzimidazoles in medicinal chemistry.^26^ After *N*-tosylation of urea **5**, LiAlH_4_ reduction of the resulting **27** gave aniline **28** in 54% yield. Treatment of **27** with Red-Al, in contrast, yielded 57% of the *N*-methyl aniline **29**, which could also be converted to diamine **26** in 39% yield after tosyl deprotection of **29** using sodium naphthalenide.

Selective functionalization of the 1,1-disubstituted alkene in **5** was also readily feasible. Hydrocarboxylation^27^ with Pd(OAc)_2_ in the presence of dppf, formic acid, and phenyl formate led to the desired carboxylic acid **30** in 46% yield in a regioselective and stereoselective fashion. The structure of **30** was secured by an X-ray analysis (Scheme 5). The exocyclic alkene moiety can also be reduced without opening of the cyclopropane ring using hydrogen with Pd/C in THF to give **31** in 81% yield. Resubjecting **31** to hydrogenation in a protic solvent such as MeOH afforded the hexahydro-1*H*-cyclopenta[*b*]quinoline scaffold **32** in 88% yield by reduction of the strained bridged cyclopropane ring to a fused bicycle.^25^

**Scheme 6:**
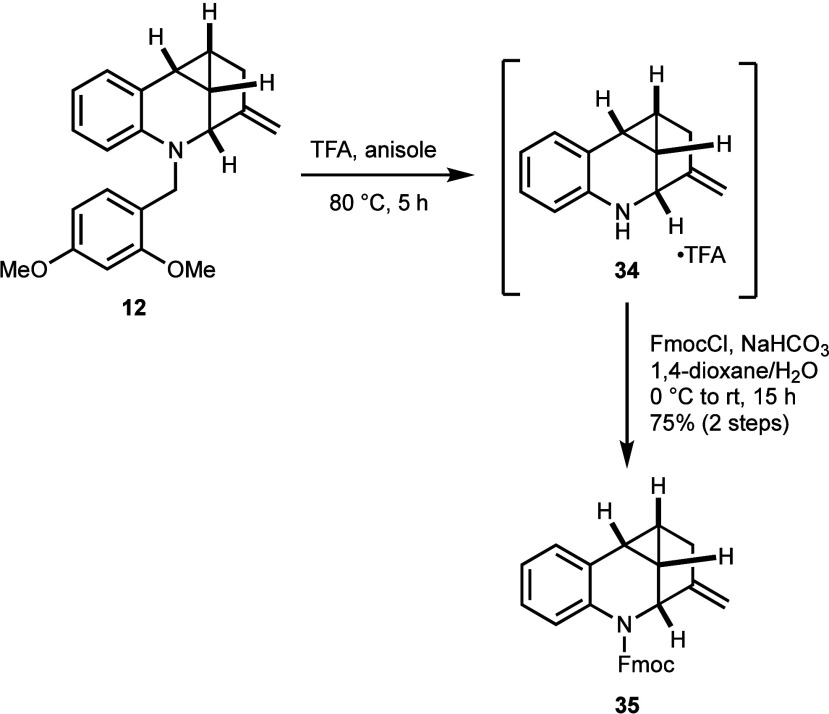
Deprotection of 2,4-Dimethoxybenzyl Amine **12** and Conversion of the Intermediate Aniline Salt **34** to the Fmoc-Carbamate **35**

The 2,4-dimethoxybenzyl group proved to be the most suitable *N*-protective group for removal under mild conditions that optimally preserved the cyclopropane and alkene functions in 1-methylene-5-azacyclopropa[*cd*]indenes (Scheme 6). Treatment of **12** with trifluoroacetic acid and anisole generated the corresponding aniline trifluoroacetate, which was further converted for full characterization to the corresponding Fmoc-protected **35** in 75% yield over two steps.

## Mechanistic Analysis

**Figure 1:**
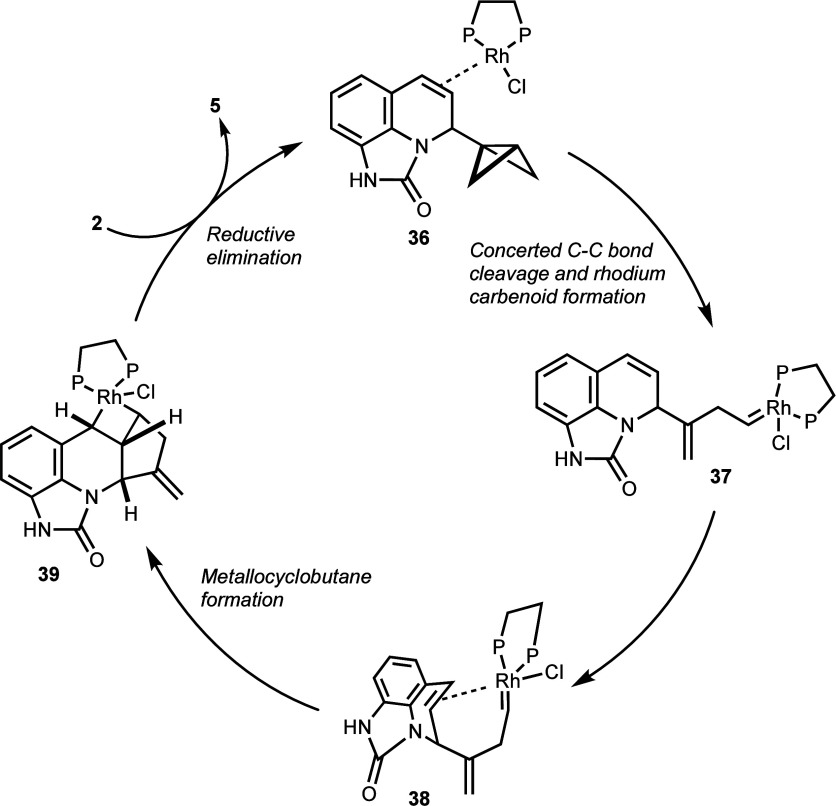
Mechanistic hypothesis for the conversion of BCB **2** to 2-methylene-hexahydro-1*H*-3-azabenzo[*f*]cyclopropa[*cd*]indene **5** via rhodium carbenoid intermediates **37** and **38**.

Based on our previous computational analyses of the Rh(I)-catalyzed rearrangement of BCBs using [Rh(CO)_2_Cl]_2_ as catalyst and 1,2-bis(diphenylphosphino)ethane as ligand,^28^ we envisioned that the conversion of BCB-adduct **2** involves an initial coordination of a Rh(I)-species to the *endo*-olefin to form Rh(I)-π-complex **36** (Figure 1). Subsequently, attack of the rhodium catalyst at the external carbon of the BCB via a double σ-bond insertion produces the Rh-carbenoid species **37**. At this point, the rhodium catalyst can coordinate with the *endo*-olefin to give **38**, which is then converted to the metallacycle intermediate **39**. Product **5** results after C–C bond formation and reductive elimination, regenerating the active rhodium(I) catalyst for the next catalytic cycle. This mechanistic hypothesis and the subtleties of ligand effects and stereochemical preferences were further examined in a computational study.

## Computational Analysis

To validate the proposed mechanism and further investigate the origin of diastereoselectivity, we conducted transition state analyses. Density functional theory (DFT) calculations were performed under standard conditions, employing the PBE0-D3(BJ)/def2-SVP method for geometry optimization and the PBE0-D3(BJ)/def2-TZVPP-SMD(1,4-dioxane) method for single-point energy calculations, as detailed in the Supporting Information.^29−32^ The structures of **2**, **5**, and **30**, as obtained experimentally, were compared to their DFT-optimized geometries. The maximum root-mean-square deviation (RMSD) observed was 0.067 (SI, Figure S11). This agreement supports the validity of the computational methods.

**Figure 2:**
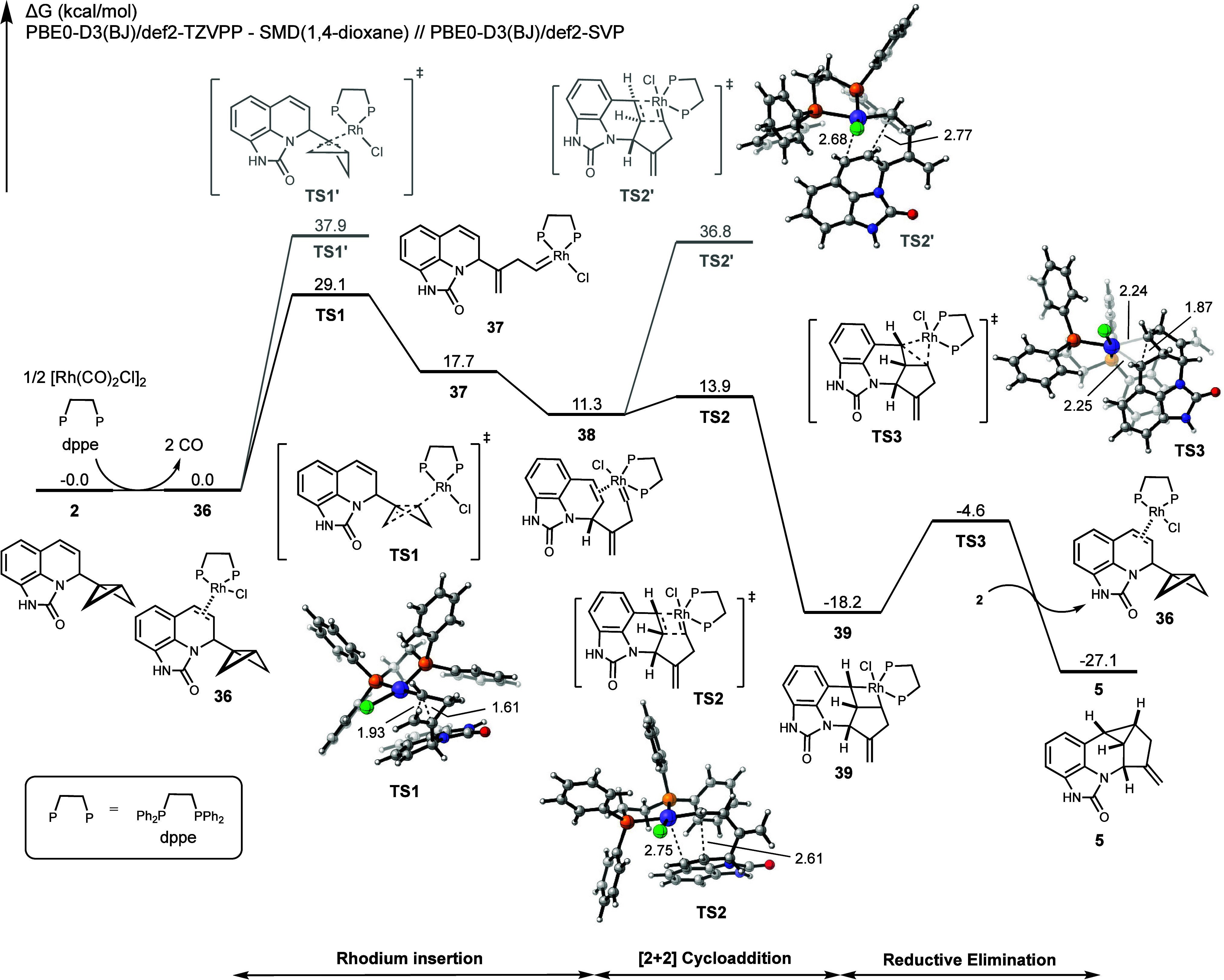
Overall free energy profile of the conversion of bicyclobutane **2** to pentacycle **5**. Bond lengths are in Å.

We initially examined the catalyst initiation stage, starting with bicyclobutane **2**. The formation of the resting state **36** involves the binding of the catalyst and ligand and occurs without loss or gain in Δ*G* (Figure 2). Subsequently, complex **36** undergoes a concerted double σ-bond insertion via **TS1**, producing Rh-carbenoid species **37**. This step has an energy barrier of 29.1 kcal/mol, making it the rate-determining step of the entire catalytic cycle. An intrinsic reaction coordinate (IRC) analysis was performed to confirm that this process is concerted (SI, Figure S8).

The rhodium spontaneously binds to the proximal side of the double bond in the dihydropiperidine, forming the more stable conformer **38**. The latter species undergoes a cycloaddition through **TS2** to yield metallacyclobutane intermediate **39**, which finally undergoes reductive elimination via **TS3** to produce the experimentally observed product **5** and regenerates the catalyst–substrate complex **36**.

**Figure 3:**
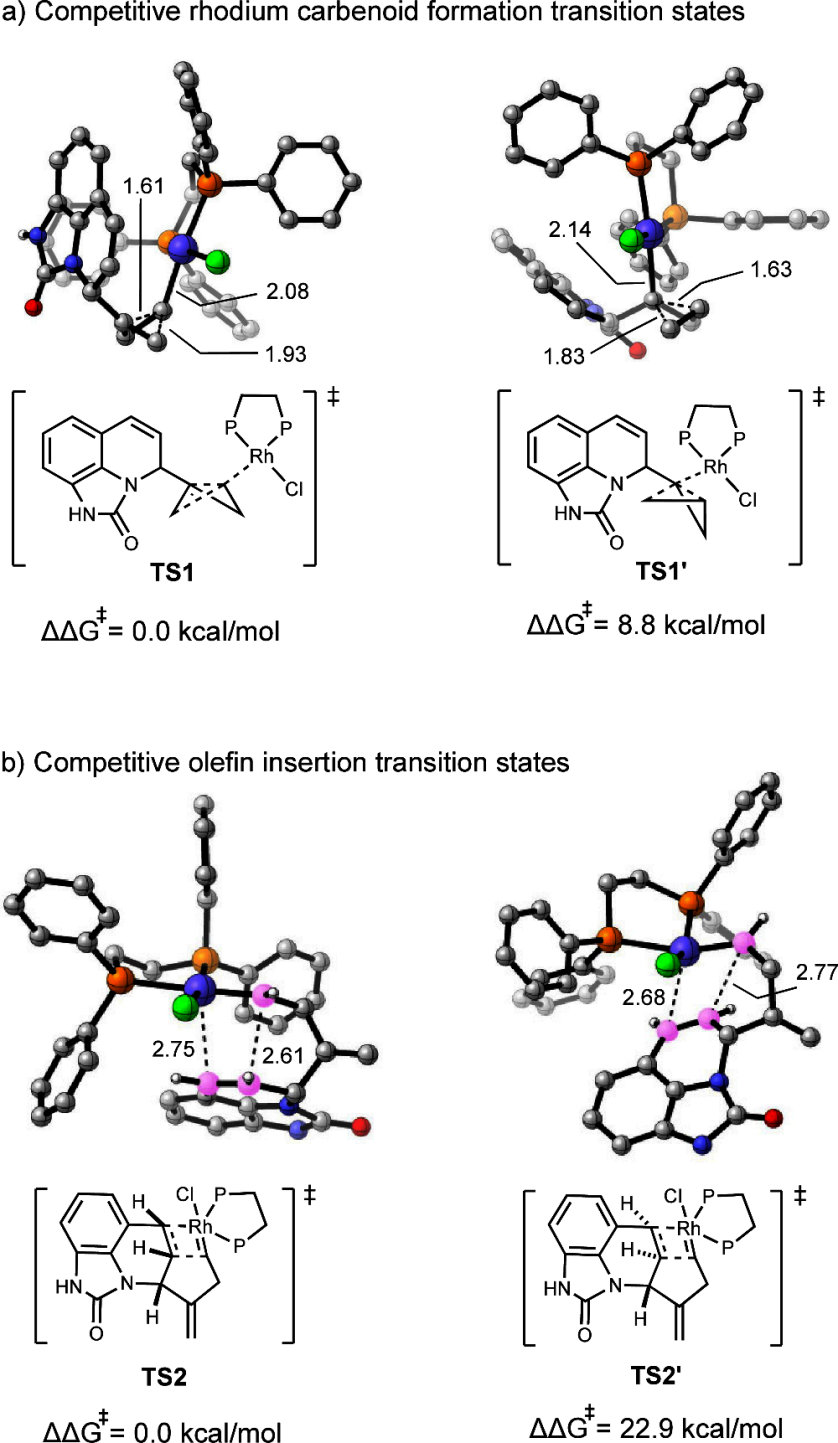
DFT-optimized structures of (a) rhodium carbenoid formation and (b) olefin insertion transition states. Calculations were performed at the PBE0-D3(BJ)/def2-TZVPP-SMD(1,4-dioxane) // PBE0-D3(BJ)/def2-SVP level of theory. Bond lengths are in Å.

The cleavage of the internal C–C bond of the bicyclobutane, accompanied by carbenoid formation, may place the rhodium complex at the external carbon through transition state **TS1** or, alternatively, at the internal carbon via the transition state **TS1′**. **TS1′** is less favorable compared to **TS1**, with a Gibbs free energy difference of 8.8 kcal/mol (Figure 3a). This indicates that the preferred pathway for C–C bond cleavage involves external carbenoid formation. A possible reason for this energy difference is the lower steric repulsion when the catalyst attacks the unsubstituted external carbon in **TS1**. In **TS1′**, attack on the more sterically hindered internal carbon atom causes a larger distortion in the catalyst and the substrate. Distortion–interaction analyses^32,33^ confirmed this hypothesis (Figure S9). In order to investigate the stereoselectivity of the reaction, we also examined the transition states of the two cycloisomerization steps of the intermedial bicyclobutane complex **38** (Figure 3b). We note that in **TS2′** the short carbon chain generated from the opening of the bicyclobutane exhibits a higher tension, hindering the formation of the four-membered transition state. In contrast to **TS2**, the four-membered ring in **TS2′** is subjected to a greater distortion and has longer bond lengths. **TS2′** is therefore significantly higher in energy than **TS2** by 22.9 kcal/mol in terms of Gibbs free energy. The persistence of this energy difference, even after the removal of the rhodium complex, confirms that ring strain is the primary factor driving the facial selectivity of the cycloaddition step (SI, Figure S10).

In conclusion, we have developed a general method for the synthesis of BCB-containing dihydroquinolines and dihydropyridines through the addition of BCB-MgCl to the α-carbon of 8-isocyanatoquinoline or, alternatively, quaternary ammonium ions of quinoline and pyridine heterocycles. The resulting BCB-containing heterocycles allowed us to significantly expand the rhodium(I)-catalyzed annulation of *N*-allyl bicyclobutyl alkylamines to access novel 1-methylene-5-azacyclopropa[*cd*]indenes. Computational analyses of the reaction pathway confirm that the preferred pathway for C–C bond cleavage involves the formation of external rhodium carbenoid **37**. Furthermore, the DFT analysis indicates that the regio- and stereospecificity of the annulation reaction are driven by the ring strain in the formation of rhodium cyclobutene **39**.

Both the BCB-addition and the rearrangement steps, as well as the selective functional group interconversions of the resulting 1-methylene-5-azacyclopropa[*cd*]indenes, tolerate a wide variety of functionalities and are therefore suitable for the preparation of a broad range of useful heterocyclic building blocks for future applications in medicinal chemistry and organic synthesis. The ease of access and the robustness of further synthetic manipulations of the novel scaffolds **5** and **6** suggest considerable opportunities for additional expansion of the chemistry of heterocyclic BCBs.

## Supporting Information Available

Experimental details and spectral data for all new compounds and details of the computational analysis, including 3D figures and tables with comparisons of DFT-optimized geometries and X-ray structures (PDF)

## ABBREVIATIONS

BCB: bicyclo[1.1.0]butane
dppp: 1,3-bis(diphenylphosphino)propane
dppe: 2-bis(diphenylphosphino)ethane
dcpe: bis(dicyclohexylphos-phino)ethane
dppb: 1,4-bis(diphenylphosphino)butane
dfppe: 1,2-ethanediylbis[bis(pentafluorophenyl)phosphine]
