# Supplementary material for: Rhodium(I)-Catalyzed Annulation of Bicyclo[1.1.0]butyl-Substituted Dihydroquinolines and Dihydropyridines
Source: J Am Chem Soc. 2024 May 20;146(22):14927–34. doi: 10.1021/jacs.4c04081 (PMC11157536; doi:10.1021/jacs.4c04081)
Supplement: Supplementary file 1 — ja4c04081_si_001.pdf [file ja4c04081_si_001.pdf]

Supporting information

Rhodium(I)-Catalyzed Annulation of Bicyclo[1.1.0]butyl-Substituted Dihydroquinolines and Dihydropyridines

Matteo Borgini,[#,‡] Qi-Nan Huang,[^,†] Pan-Pan Chen,[^] Steven J. Geib,[#] K. N. Houk,[^,*] and Peter Wipf[#,*]

[#]Department of Chemistry, University of Pittsburgh, Pittsburgh PA 15260, USA
[^]Department of Chemistry and Biochemistry, University of California, Los Angeles, CA 90095, USA
[‡]Department of Chemistry and Biochemistry, Augusta University, Augusta GA 30912, USA
[†]College of Chemistry and Chemical Engineering, Hunan University, Changsha, Hunan 410082, P. R. China

Table of Contents

General methods for chemical synthesis and analysis S1

Synthesis of 4-(bicyclo[1.1.0]butan-1-yl)-4*H*-imidazo[4,5,1-*ij*]quinolin-2(1*H*)-one (**2**) S2

Optimization of the reaction conditions for the conversion of **13** into (2aSR,2a^{1}SR,2bRS,7bRS)-1-methylene-1,2,2a,2a^1,2b,7b-hexahydro-6,7a-diazabenzo[*cd*]cyclopropa[*fg*]-*as*-indacen-7(6*H*)-one (**5**) and scale-up ... S4

Protocols for the synthesis of quinolinium and pyridinium ions S5

Substrate scope for the addition of BCB-MgCl into quaternary ammonium salts followed by Rh(I)-catalyzed rearrangement S8

Derivatization of rearranged products S17

Computational details S22

References S82

Copies of ^{1}H NMR, ^{13}C NMR, COSY, DEPT and HSQC spectra S83

General methods for chemical synthesis and analysis. Unless stated otherwise, all reactions were performed under an atmosphere of N_2 that was passed through a column (10x2 cm) of Drierite®. Degassed solvents were prepared by freeze-pump-thaw techniques. *N,N*-Diisoproylethylamine (DIPEA) was freshly distilled over CaH_2 before use. All glassware and stir bars were flame-dried or stored in a drying oven prior to use. Reactions were monitored by TLC (thin layer chromatography) analysis using pre-coated silica gel 60 F254, and spots were visualized using UV light at 254 nm and 385 nm. Purifications by chromatography were performed on SiO_2. ^{1}H / ^{13}C NMR spectra were recorded on Bruker Avance 300/76 MHz, Bruker Avance 400/101 MHz, Bruker Avance 500/126 MHz or Bruker Avance 601/151 MHz instruments. High resolution mass spectra were obtained on a Micromass UK Limited, Q-TOF Ultima API or a Thermo Scientific Exactive Orbitrap LC-MS. IR

spectra were obtained using neat samples on a PerkinElmer 100 IR ATR spectrometer. Melting points were obtained using a Mel-Temp instrument and are not corrected. Chemical shifts were reported in parts per million (ppm) with the residual solvent peak ($CDCl_3$: 7.26 ppm for ^1H, 77.16 ppm for ^{13}C; DMSO-d_6: 2.50 ppm for ^1H, 39.52 ppm for ^{13}C) used as the internal standard. Chemical shifts were tabulated as follows: chemical shift, multiplicity (s = singlet, d = doublet, t = triplet, q = quartet, h = sextet, dd = doublet of doublet, dt = doublet of triplet, ddd = doublet of doublet of doublet, tt = triplet of triplet, dtd = double of triplet of doublet, dddt = doublet of doublet of doublet of triplet, ddq = doublet of doublet of quartet, dtt = doublet of doublet of triplet, tqd = triplet of quartet of doublet, dpd = doubled of pentet of doubled, tdd = triplet of doublet of doublet, qdd = quartet of doublet of doublet, m = multiplet, br s = broad singlet), coupling constant(s), and integration.

Synthesis of 4-(bicyclo[1.1.0]butan-1-yl)-4*H*-imidazo[4,5,1-*ij*]quinolin-2(1*H*)-one (2).

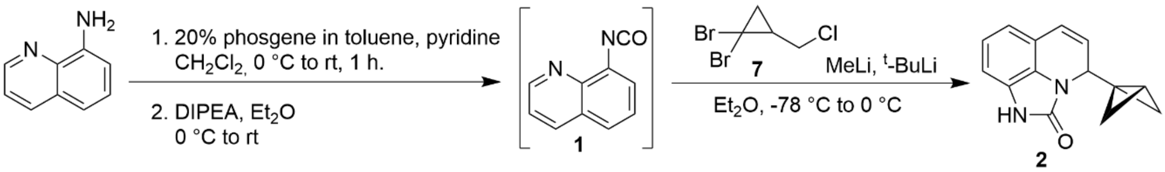

8-Isocyanatoquinoline (1). To a solution of phosgene (18.2 mL, 34.7 mmol, 2 eq, 20% in toluene) in CH_2Cl_2 (8 mL), pyridine (1.41 mL, 17.3 mmol, 1 eq) was added at 0 °C. Thereafter, a CH_2Cl_2 solution (15 mL) of 8-aminoquinoline (2.50 g, 17.3 mmol, 1 eq) was added dropwise at 0 °C and the reaction mixture was stirred at room temperature for 1 h. The orange precipitate was filtered, washed with CH_2Cl_2, and dried under high vacuum to give 8-isocyanatoquinoline hydrochloride (2.65 g, 15.6 mmol, 90% yield) as an orange solid. A small aliquot was dissolved in MeOH and analyzed by LC-MS showing the $[M+H]^+$ of the desired methylcarbamate. The stability of 8-isocyanatoquinoline hydrochloride has been monitored for up to six months at -20 °C showing no decomposition. The product was directly used in the next step.

A three-neck round bottom flask was equipped with a frit and a second three-neck round bottom flask and both were flame-dried and flushed with nitrogen (Figure S1). 8-Isocyanatoquinoline hydrochloride (2.50 g, 12.1 mmol, 1 eq) was added (Figure S2) followed by Et_2O (30 mL) (Figure S3), and the mixture was cooled to 0 °C. Then, freshly distilled DIPEA (4.20 mL, 24.2 mmol, 2 eq) was added dropwise and the reaction mixture was stirred for 1 h at 0 °C, followed by an additional hour at room temperature (Figure S4). The apparatus was flipped in order to separate the yellow solid from the colorless mother liquor (Figure S5). Fresh Et_2O (10 mL) was used to rinse. The mother liquor was concentrated under vacuum to give **1** as a white solid (Figures S6 and S7). Compound **1** was directly used in the next step.

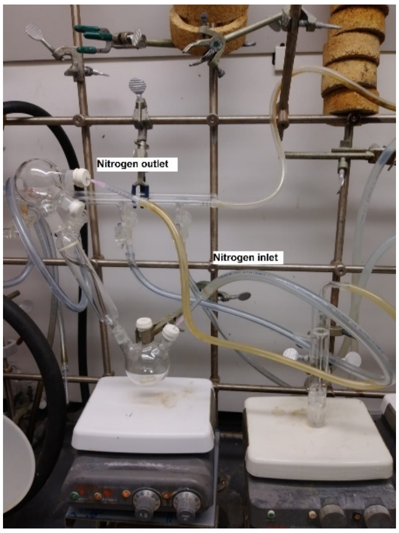

Figure S1

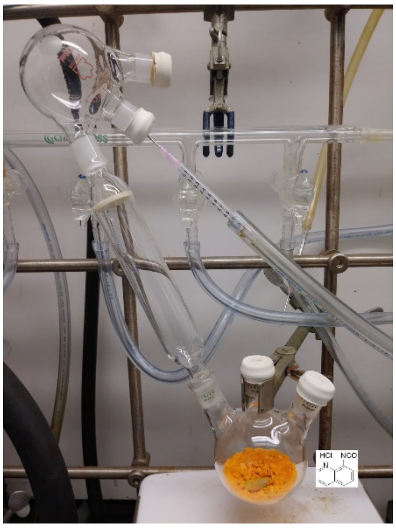

Figure S2

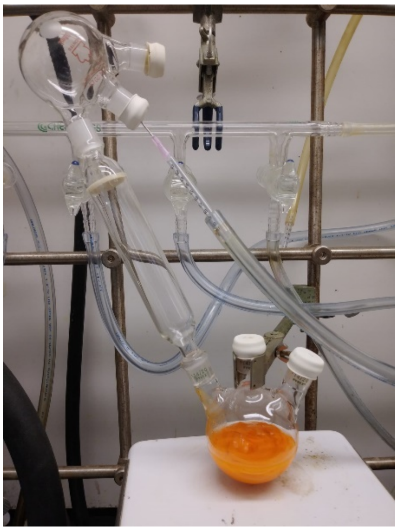

Figure S3

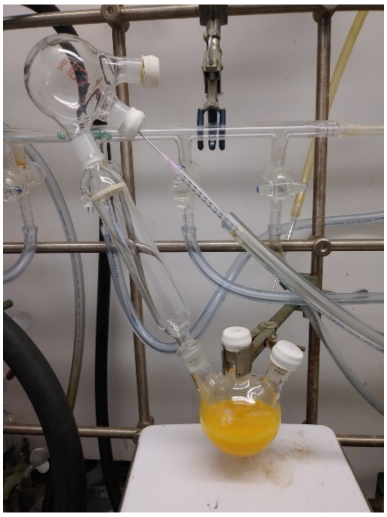

Figure S4

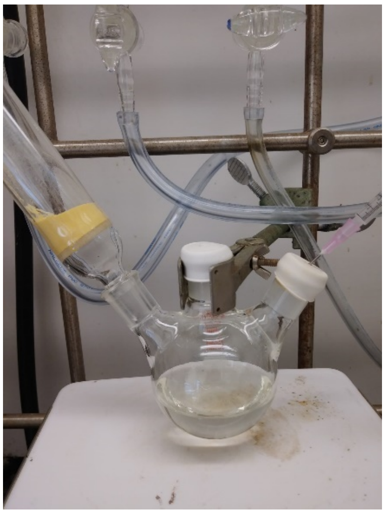

Figure S5

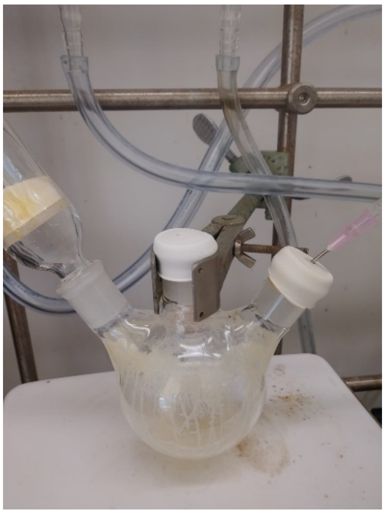

Figure S6

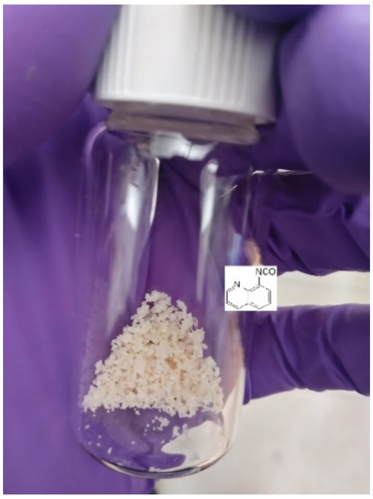

Figure S7

4-(Bicyclo[1.1.0]butan-1-yl)-4*H*-imidazo[4,5,1-*ij*]quinolin-2(1*H*)-one (**2**). A flame-dried nitrogen-flushed 100-mL Schlenk flask was charged with compound **7**[1] (1.39 g, 5.58 mmol, 1 eq) and Et_2O (11.5 mL, 0.5 M). The solution was cooled to –78 °C (dry ice/acetone), then methyl lithium (1.80 mL of 3.1 M solution in diethoxymethane, 5.58 mmol, 1 eq) was added dropwise under N_2. The reaction mixture was stirred for 30 min at –78 °C followed by 1 h at –50 °C (dry ice/acetonitrile). The solution was then cooled to –78 °C and vacuum was introduced for 5 min to remove MeBr. *t*-Butyl lithium (3.28 mL of a 1.7 M solution, 5.58 mmol, 1 eq) was then added dropwise under N_2 and the mixture was stirred for 1 h at -78 °C. An Et_2O solution (7 mL) of the isocyanate **1** (0.95 g, 5.58 mmol, 1 eq) was added dropwise and the reaction mixture was stirred for 2 h at –78 °C followed by 20 min at 0 °C. Saturated NH_4Cl was added and the mixture was extracted with Et_2O/H_2O. The organic layer was washed with brine, dried ($MgSO_4$), and concentrated. The crude residue was purified by chromatography on SiO_2 (30% ethyl acetate in hexanes) to give **2** (0.41 g, 1.84 mmol, 33%) as a pale-yellow solid: **Mp** 143.1 - 145.5 °C; **IR** (ATR, neat) 3192, 3039, 2932, 1686, 1479, 1384, 1324, 1161, 1142, 1099, 1077, 752, 738 cm^{-1}; ^{1}**H NMR** (601 MHz, Chloroform-*d*) δ 9.61 (s, 1 H), 7.05 – 6.84 (m, 2 H), 6.79 (q, *J* =

4.3 Hz, 1 H), 6.57 (dd, *J* = 10.0, 1.7 Hz, 1 H), 5.66 (dd, *J* = 9.9, 4.1 Hz, 1 H), 5.47 (dd, *J* = 4.2, 1.7 Hz, 1 H), 1.75 – 1.72 (m, 2 H), 1.67 (td, *J* = 2.8, 1.4 Hz, 1H), 0.84 (d, *J* = 1.4 Hz, 1 H), 0.66 (d, *J* = 1.3 Hz, 1 H); ^{13}**C NMR** (151 MHz, Chloroform-*d*) δ 155.8, 126.9, 125.5, 124.4, 123.1, 121.6, 117.3, 116.3, 109.6, 52.8, 33.3, 31.5, 13.9, 1.6; **HRMS** (ESI) *m/z* calcd for $C_{14}H_{11}N_2O$ [M + H]$^+$, 223.0866; found, 223.0874. For the solved X-ray structure of this compound, please see CCDC deposition 2340745.

Optimization of the reaction conditions for the conversion of 2 into (2a*SR*,2a^1*SR*,2b*RS*,7b*RS*)-1-methylene-1,2,2a,2a^1,2b,7b-hexahydro-6,7a-diazabenzo[*cd*]cyclopropa[*fg*]-*as*-indacen-7(6*H*)-one (5).

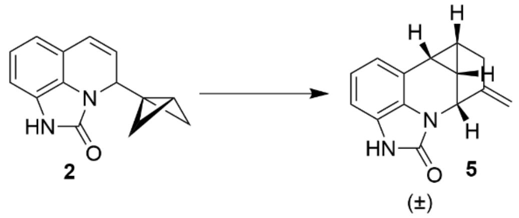

Table 1. Optimization conditions

Entry	Catalyst (5 mol%) / Ligand (10 mol%)	Solvent [0.05 M]	Temp (°C)	Time	NMR yield
1	$[Rh(CO)_2Cl]_2$ / dppe	Toluene[a]	120 °C	30 min	69%
2	$[Rh(CO)_2Cl]_2$ / dppe	1,4-dioxane	120 °C	30 min	80% (77%)[b]
3	$[Rh(CO)_2Cl]_2$ / dppp	Toluene[a]	120 °C	30 min	19%
4	$[Rh(CO)_2Cl]_2$ / PPh_3	Toluene[a]	120 °C	30 min	18%
5	$[Rh(CO)_2Cl]_2$ / dppe	1,4-dioxane	90 °C	30 min	46%
6	$[Rh(CO)_2Cl]_2$ / dppe	1,4-dioxane	80 °C	60 min	53%
7	$[Rh(CO)_2Cl]_2$ / dfppe	1,4-dioxane	120 °C	30 min	51%
8	$[Rh(CO)_2Cl]_2$ / tribenzylphosphine	1,4-dioxane	120 °C	30 min	36%
9	$[Rh(CO)_2Cl]_2$ / dcpe	1,4-dioxane	120 °C	30 min	39%
10	$[Rh(CO)_2Cl]_2$ / dppb	1,4-dioxane	120 °C	30 min	24%
11	$Pd(dba)_2$ / PPh_3	1,4-dioxane	120 °C	30 min	0% [c]
12	Bis(tricyclohexylphosphine)palladium(0)	1,4-dioxane	120 °C	120 min	0% [c]
13	$Pd(dba)_2$ / tricyclohexylphosphine	1,4-dioxane	120 °C	120 min	0% [c]
14	$Pd(dba)_2$ / tributylphosphine	1,4-dioxane	120 °C	120 min	0% [c]

a = The reaction mixture was degassed before heating; b = Isolated yield; c = unreacted starting material.

(2a*SR*,2a^1*SR*,2b*RS*,7b*RS*)-1-Methylene-1,2,2a,2a^1,2b,7b-hexahydro-6,7a-diazabenzo[*cd*]cyclopropa[*fg*]-as-indacen-7(6*H*)-one (**5**). To a flame dried microwave vial (2.0–5.0 mL size), **2** (23 mg, 0.10 mmol, 1 eq), 1,2-bis(diphenylphosphino)ethane (4.1 mg, 0.01 mmol, 10 mol%), and $[Rh(CO)_2Cl]_2$ (2 mg, 5.1 µmol, 5 mol%) were added subsequently. Then, 1,4 dioxane (2 mL, 0.05 M) was added and the microwave vial was sealed with a silicon/PTFE crimp cap. The reaction mixture was heated in a pre-heated sand bath at 120 °C for 30 min under nitrogen, cooled to room temperature and concentrated *in vacuo*. The crude residue was dissolved in 5% MeOH in CH_2Cl_2 and filtered through a pad of celite® (5% MeOH in CH_2Cl_2 to rinse). The solvents were evaporated and the resulting residue was purified by chromatography on SiO_2 (1% MeOH in CH_2Cl_2) to give **5** (17.7 mg, 0.08 mmol, 77%) as a white solid: **Mp** 223.6 - 226.1 °C; **IR** (ATR, neat) 3117, 3050, 2924, 2846, 2775, 2705, 1683, 1485, 1395, 1318, 1152, 1131, 1076, 909, 747, 735 cm^{-1}; ^{1}**H NMR** (400 MHz, Chloroform-*d*) δ 9.28

(br s, 1 H), 7.08 (d, *J* = 7.6 Hz, 1 H), 6.99 (t, *J* = 7.7 Hz, 1 H), 6.90 (d, *J* = 7.7 Hz, 1 H), 5.28 – 5.23 (m, 2 H), 4.74 – 4.70 (m, 1 H), 2.52 (dd, *J* = 17.5, 7.0 Hz, 1 H), 2.36 (t, *J* = 8.1 Hz, 1 H), 2.24 (q, *J* = 7.3 Hz, 1 H), 2.18 – 2.09 (m, 1 H), 1.78 (d, *J* = 17.4 Hz, 1 H); **^{13}C NMR** (76 MHz, DMSO-d_6) δ 155.9, 153.9, 127.0, 126.4, 120.8, 120.4, 115.6, 108.4, 106.5, 54.1, 28.9, 27.0, 19.5, 18.3; **HRMS** (ESI) *m/z* calcd for $C_{14}H_{13}N_2O$ [M + H]$^+$, 225.1022; found, 225.1065. For the solved X-ray structure of this compound, please see CCDC deposition 2340746.

Scale up to 1 g. To a flame-dried 250-mL Schlenk flask, **2** (1.01 g, 4.50 mmol, 1 eq) and 1,2-bis(diphenylphosphino)ethane (0.272 g, 0.68 mmol, 15 mol%) were added. Then, $[Rh(CO)_2Cl]_2$ (131 mg, 0.34 mmol, 7.5 mol%) was added followed by 1,4-dioxane (56 mL, 0.08 M). The Schlenk flask was heated in a preheated sand bath at 120 °C for 45 min under argon. The reaction mixture was cooled down and the solvent evaporated. The crude residue was dissolved in 5% MeOH in CH_2Cl_2 and filtered through a pad of celite (5% MeOH in CH_2Cl_2 to rinse). The solvents were evaporated, and the resulting mixture was purified by chromatography on SiO_2 (1% MeOH in CH_2Cl_2) to give **5** (0.85 g, 3.81 mmol, 85%) as a white solid.

Protocols for the synthesis of quinolinium and pyridinium ions.

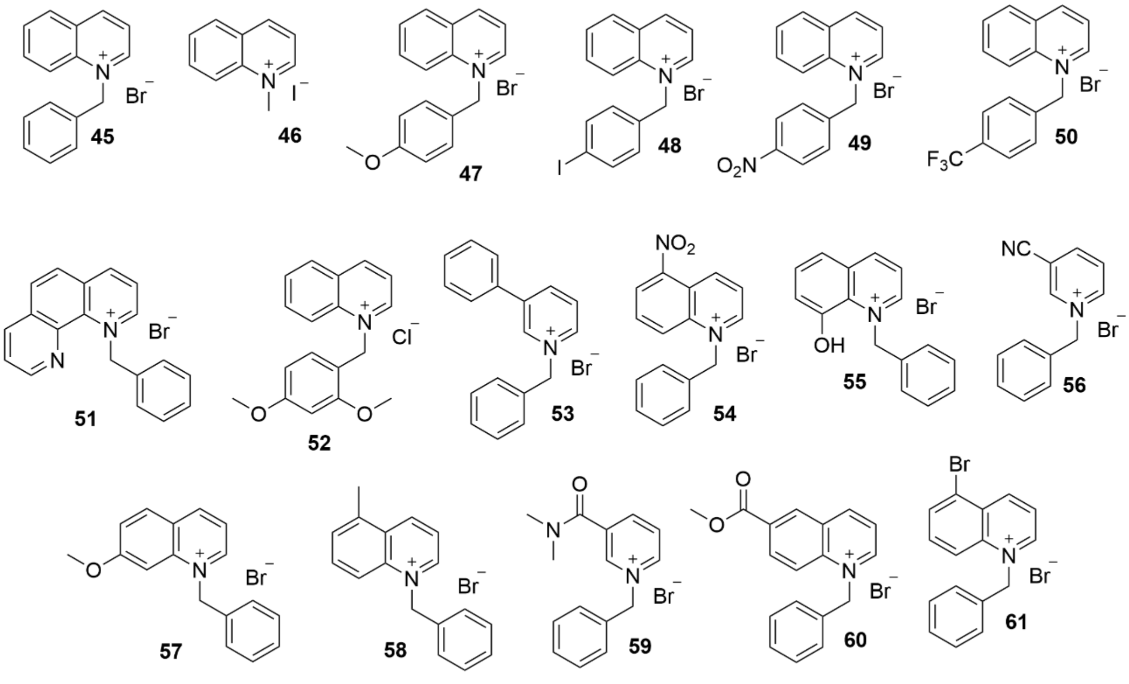

General protocol A for the synthesis of quaternary ammonium salts 45, 46, 47, 48, 49, 50, 53, 54, 55, 56, 57, 58.[2,3,4,5]

To a MeCN solution (0.4 M) of the corresponding haloalkane (1 eq), the quinoline or pyridine derivative (1 eq) was added. The reaction mixture was heated at reflux for 2 h and then cooled down to room temperature. The desired product was obtained as specified below.

1-Benzylquinolin-1-ium bromide (**45**). The solvent was evaporated and the residue washed with ethyl acetate (2x) to afford **45** as a pale pink solid: **^{1}H NMR** (300 MHz, DMSO-d_6) δ 9.78 (dd, *J* = 5.9, 1.5 Hz, 1 H), 9.39 (dd, *J* = 8.6, 1.3 Hz, 1 H), 8.57 – 8.47 (m, 2 H), 8.31 (dd, *J* = 8.4, 5.8 Hz, 1 H), 8.22 (ddd, *J* = 8.7, 7.0, 1.5 Hz, 1 H), 8.03 (ddd, *J* = 8.1, 7.1, 0.9 Hz, 1 H), 7.39 (s, 3 H), 7.38 (td, *J* = 6.7, 2.8 Hz, 2 H), 6.40 (s, 2 H).

1-Methylquinolin-1-ium iodide (**46**). Toluene was used instead of MeCN. After cooling to room temperature, the precipitate was collected via vacuum filtration and washed sequentially with Et_2O (3x) and petroleum

ether (3x). The residue was further dried under reduced pressure to give **46** as a yellow solid: ^{1}**H NMR** (400 MHz, Chloroform-*d*) δ 9.51 (dd, *J* = 5.8, 1.5 Hz, 1 H), 9.29 (d, *J* = 8.4 Hz, 1 H), 8.54 – 8.47 (m, 2 H), 8.30 (ddd, *J* = 8.8, 7.0, 1.5 Hz, 1 H), 8.18 (dd, *J* = 8.4, 5.7 Hz, 1 H), 8.07 (ddd, *J* = 8.1, 7.0, 0.9 Hz, 1 H), 4.64 (s, 3 H).

1-(4-Methoxybenzyl)quinolin-1-ium bromide (**47**). The desired product crystallized spontaneously forming pale-yellow crystals: ^{1}**H NMR** (500 MHz, DMSO-d_6) δ 9.79 (dd, *J* = 5.8, 1.5 Hz, 1 H), 9.38 (d, *J* = 8.3 Hz, 1 H), 8.63 (d, *J* = 9.0 Hz, 1 H), 8.51 (dd, *J* = 8.3, 1.5 Hz, 1 H), 8.28 (dd, *J* = 8.4, 5.8 Hz, 1 H), 8.23 (ddd, *J* = 8.8, 7.0, 1.5 Hz, 1 H), 8.02 (t, *J* = 7.6 Hz, 1 H), 7.46 – 7.40 (m, 2 H), 6.98 – 6.87 (m, 2 H), 6.33 (s, 2 H), 3.72 (s, 3 H).

1-(4-Iodobenzyl)quinolin-1-ium bromide (**48**). The mixture was concentrated to a ca. 2-mL volume, and Et_2O (5 mL) was added to form a precipitate. The supernatant was removed, and the residue was washed with Et_2O (2x) and dried under high vacuum to afford **48** as a pale-yellow solid: ^{1}**H NMR** (300 MHz, DMSO-d_6) δ 9.72 (dd, *J* = 5.9, 1.5 Hz, 1 H), 9.38 (d, *J* = 8.5 Hz, 1 H), 8.54 – 8.44 (m, 2 H), 8.34 – 8.16 (m, 2 H), 8.03 (ddd, *J* = 8.1, 7.0, 0.9 Hz, 1 H), 7.82 – 7.68 (m, 2 H), 7.30 – 7.14 (m, 2 H), 6.33 (s, 2 H).

1-(4-Nitrobenzyl)quinolin-1-ium bromide (**49**). The mixture was concentrated to a ca. 2-mL volume, and Et_2O (5 mL) was added to form a precipitate. The supernatant was removed, and the residue was washed with Et_2O (2x) and dried under high vacuum to afford **49** as an orange solid: ^{1}**H NMR** (300 MHz, DMSO-d_6) δ 9.81 (dd, *J* = 5.8, 1.5 Hz, 1 H), 9.44 (dd, *J* = 8.4, 1.4 Hz, 1 H), 8.55 (dd, *J* = 8.2, 1.5 Hz, 1 H), 8.45 – 8.29 (m, 2 H), 8.28 – 8.15 (m, 3 H), 8.04 (ddd, *J* = 8.1, 7.0, 0.9 Hz, 1 H), 7.67 – 7.56 (m, 2 H), 6.57 (s, 2 H).

1-(4-(Trifluoromethyl)benzyl)quinolin-1-ium bromide (**50**). The mixture was concentrated to a ca. 2-mL volume, and Et_2O (5 mL) was added to form a precipitate. The supernatant was removed, and the residue was washed with Et_2O (2x) and dried under high vacuum to afford **50** as a white solid: ^{1}**H NMR** (300 MHz, DMSO-d_6) δ 9.78 (dd, *J* = 5.9, 1.5 Hz, 1 H), 9.42 (d, *J* = 8.4 Hz, 1 H), 8.54 (dd, *J* = 8.3, 1.5 Hz, 1 H), 8.44 (d, *J* = 8.9 Hz, 1 H), 8.33 (dd, *J* = 8.4, 5.8 Hz, 1 H), 8.22 (ddd, *J* = 8.8, 7.0, 1.5 Hz, 1 H), 8.04 (t, *J* = 7.6 Hz, 1 H), 7.77 (d, *J* = 8.2 Hz, 2 H), 7.57 (d, *J* = 8.1 Hz, 2 H), 6.50 (s, 2 H).

1-Benzyl-3-phenylpyridin-1-ium bromide (**53**). The solvent was evaporated and the resulting solid was washed with Et_2O (3x) and dried under high vacuum to give **53** as a white solid: ^{1}**H NMR** (300 MHz, DMSO-d_6) δ 9.85 (s, 1 H), 9.23 (d, *J* = 6.0 Hz, 1 H), 8.97 (d, *J* = 8.2 Hz, 1 H), 8.25 (dd, *J* = 8.2, 6.0 Hz, 1 H), 8.00 – 7.91 (m, 2 H), 7.73 – 7.66 (m, 2 H), 7.66 – 7.51 (m, 3 H), 7.65 – 7.7.52 (m, 3 H), 6.04 (s, 2 H).

1-Benzyl-5-nitroquinolin-1-ium bromide (**54**). The solvent was evaporated and the resulting solid was solubilized with water and extracted with CH_2Cl_2 (5x). The water layer was freeze-dried to afford **54** as a yellow solid: ^{1}**H NMR** (300 MHz, DMSO-d_6) δ 9.93 (dd, *J* = 5.8, 1.3 Hz, 1 H), 9.61 (dt, *J* = 9.0, 1.1 Hz, 1 H), 8.90 (dt, *J* = 9.1, 1.0 Hz, 1 H), 8.73 (dd, *J* = 7.8, 0.8 Hz, 1 H), 8.50 (dd, *J* = 9.0, 5.7 Hz, 1 H), 8.36 (dd, *J* = 9.1, 7.8 Hz, 1 H), 7.49 – 7.33 (m, 5 H), 6.49 (s, 2 H).

1-Benzyl-8-hydroxyquinolin-1-ium bromide (**55**). The solvent was evaporated and the resulting solid was solubilized with water and extracted with CH_2Cl_2 (5x). The water layer was freeze-dried to afford **55** as a yellow solid: ^{1}**H NMR** (300 MHz, DMSO-d_6) δ 11.94 (s, 1 H), 9.52 (dd, *J* = 5.8, 1.5 Hz, 1 H), 9.26 (dd, *J* = 8.4, 1.4 Hz, 1 H), 8.16 (dd, *J* = 8.4, 5.8 Hz, 1 H), 7.90 (dd, *J* = 8.1, 1.5 Hz, 1 H), 7.82 (t, *J* = 7.9 Hz, 1 H), 7.52 (dd, *J* = 7.8, 1.5 Hz, 1 H), 7.40 – 7.28 (m, 3 H), 7.22 – 7.11 (m, 2H), 6.60 (s, 2 H).

1-Benzyl-3-cyanopyridin-1-ium bromide (**56**). The solvent was evaporated and the resulting solid was washed with Et_2O (3x) and dried under high vacuum to give **56** as a white solid: ^{1}**H NMR** (300 MHz, DMSO-d_6) δ 10.03 (d, *J* = 1.6 Hz, 1 H), 9.45 (dt, *J* = 6.2, 1.3 Hz, 1 H), 9.12 (dt, *J* = 8.1, 1.4 Hz, 1 H), 8.37 (dd, *J* = 8.1, 6.2 Hz, 1 H), 7.67 – 7.54 (m, 2 H), 7.53 – 7.39 (m, 3 H), 5.92 (s, 2 H).

1-Benzyl-7-methoxyquinolin-1-ium bromide (**57**). A precipitate was formed after cooling the reaction mixture to room temperature. The solid was filtered and washed with MeCN (3x). The residue was dried under high vacuum to afford **57** as a pale-yellow solid: ^{1}**H NMR** (500 MHz, DMSO-d_6) δ 9.58 (dd, *J* = 6.0, 1.5

Hz, 1 H), 9.22 (d, *J* = 8.1 Hz, 1 H), 8.41 (d, *J* = 9.1 Hz, 1 H), 8.07 (dd, *J* = 8.2, 5.9 Hz, 1 H), 7.72 – 7.64 (m, 2 H), 7.46 – 7.38 (m, 4 H), 7.40 – 7.33 (m, 1 H), 6.32 (s, 2 H), 3.98 (s, 3 H).

1-Benzyl-5-methylquinolin-1-ium bromide (**58**): The reaction mixture was treated with 0.5 mL of CH_2Cl_2 to form a grey precipitated. The solid was filtered, washed with CH_2Cl_2 (3X), and dried under high vacuum to give **58** as a white solid: **^{1}H NMR** (500 MHz, DMSO-*d*$_6$) δ 9.75 (dd, *J* = 5.8, 1.4 Hz, 1 H), 9.45 (dt, *J* = 8.7, 1.2 Hz, 1 H), 8.33 (d, *J* = 9.0 Hz, 1 H), 8.29 (dd, *J* = 8.6, 5.7 Hz, 1 H), 8.08 (dd, *J* = 9.0, 7.2 Hz, 1 H), 7.88 – 7.83 (m, 1 H), 7.47 – 7.35 (m, 2 H), 7.38 – 7.30 (m, 3 H), 6.38 (s, 2 H), 2.85 (s, 3 H).

General protocol B for the synthesis of quaternary ammonium salts 51, 52, 59, 60, 61.[2,3,4]

To an acetone solution (0.6 M) of the corresponding quinoline or pyridine derivative (1 eq), the differently substituted benzyl bromide (1 eq) was added. The resulting mixture was stirred at room temperature for 24 h. The desired product was obtained as specified below.

1-Benzyl-1,10-phenanthrolin-1-ium bromide (**51**). The solvent was evaporated and the resulting solid was washed with Et_2O (3x) and dried under high vacuum to give **51** as a white solid: **^{1}H NMR** (300 MHz, DMSO-*d*$_6$) δ 9.82 (dd, *J* = 6.0, 1.5 Hz, 1 H), 9.18 (dd, *J* = 4.3, 1.8 Hz, 1 H), 9.12 – 9.03 (m, 2 H), 8.74 (dd, *J* = 8.3, 1.9 Hz, 1 H), 8.55 (dd, *J* = 8.2, 5.9 Hz, 1 H), 8.42 (s, 2 H), 7.81 – 7.70 (m, 2 H), 7.31 – 7.19 (m, 5 H).

1-(2,4-Dimethoxybenzyl)quinolin-1-ium chloride (**52**). The solvent was evaporated and the resulting solid was washed with Et_2O (3x) and dried under high vacuum to give **52** as a white solid: **^{1}H NMR** (300 MHz, DMSO-*d*$_6$) δ 9.59 (d, *J* = 5.8 Hz, 1 H), 9.34 (d, *J* = 8.3 Hz, 1 H), 8.64 (d, *J* = 9.0 Hz, 1 H), 8.50 (d, *J* = 8.2 Hz, 1 H), 8.35 – 8.15 (m, 2 H), 8.10 – 7.82 (m, 2 H), 7.43 (d, *J* = 8.4 Hz, 1 H), 6.63 (s, 1 H), 6.60 – 6.53 (m, 1 H), 6.20 (s, 2 H), 3.75 (s, 6 H).

1-Benzyl-3-(dimethylcarbamoyl)pyridin-1-ium bromide (**59**). The solvent was evaporated and the resulting solid was washed with Et_2O (3x) and dried under high vacuum to give **59** as a white solid: **^{1}H NMR** (500 MHz, DMSO-*d*$_6$) δ 9.49 (d, *J* = 1.7 Hz, 1 H), 9.30 (dt, *J* = 6.1, 1.4 Hz, 1 H), 8.71 (dt, *J* = 8.0, 1.5 Hz, 1 H), 8.25 (dd, *J* = 8.0, 6.1 Hz, 1 H), 7.63 – 7.57 (m, 2 H), 7.49 – 7.39 (m, 3 H), 5.91 (s, 2 H), 3.04 (s, 3 H), 2.96 (s, 3 H).

1-Benzyl-6-(methoxycarbonyl)quinolin-1-ium bromide (**60**). The solvent was evaporated and the resulting solid was partitioned between H_2O and ethyl acetate. The water layer was washed with ethyl acetate (3x) and freeze-dried to give **60** as a yellow solid: **^{1}H NMR** (300 MHz, DMSO-*d*$_6$) δ 9.89 (dd, *J* = 5.9, 1.5 Hz, 1 H), 9.58 (d, *J* = 8.4 Hz, 1 H), 9.17 (d, *J* = 1.9 Hz, 1 H), 8.65 (d, *J* = 9.3 Hz, 1 H), 8.57 (dd, *J* = 9.3, 2.0 Hz, 1 H), 8.40 (dd, *J* = 8.4, 5.8 Hz, 1 H), 7.40 (s, 3 H), 7.46 – 7.33 (m, 2 H), 6.43 (s, 2 H), 3.97 (s, 3 H).

1-Benzyl-5-bromoquinolin-1-ium bromide (**61**). The solvent was evaporated and the resulting solid was washed with Et_2O (3x) and dried under high vacuum to give **61** as a yellow solid: **^{1}H NMR** (500 MHz, DMSO-*d*$_6$) δ 9.84 (dd, *J* = 5.8, 1.4 Hz, 1 H), 9.45 (d, *J* = 8.7 Hz, 1 H), 8.54 (d, *J* = 9.1 Hz, 1 H), 8.40 (dd, *J* = 8.0, 4.5 Hz, 2 H), 8.10 (dd, *J* = 9.0, 7.6 Hz, 1 H), 7.47 – 7.33 (m, 5 H), 6.42 (s, 2 H).

Substrate scope for the addition of BCB-MgCl into quaternary ammonium salts followed by Rh(I)-catalyzed rearrangement.

General Protocol A.

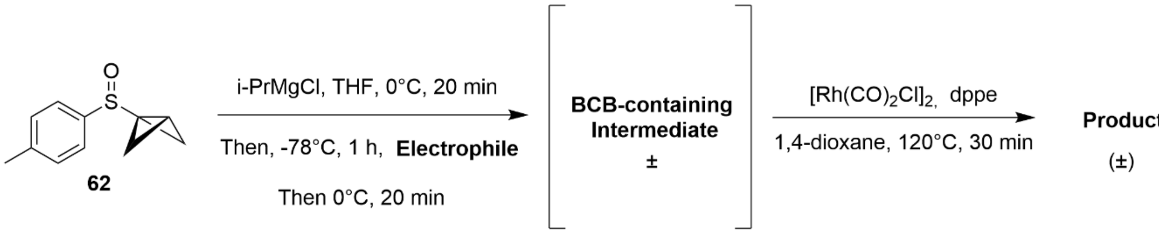

To a flame-dried flask containing a 0 °C solution of **62**[1] (2 eq) in THF (0.25 M) was added *i*-PrMgCl (2 eq). The reaction mixture was stirred for 20 min at 0 °C under nitrogen, cooled to -78 °C, treated with the quaternary ammonium salt (1 eq), stirred for 1 h at -78 °C, warmed to 0 °C, and stirred for an additional 20 min. The mixture was quenched with sat. NH_4Cl, extracted with ethyl acetate (3x), washed with brine, dried ($MgSO_4$), filtered, and concentrated under vacuum. The crude residue was transferred into a microwave vial (2.0–5.0 mL size) using CH_2Cl_2 and was used in the next step without further purification.

The crude residue (1 eq) was dissolved in 1,4 dioxane (distilled over Na/benzophenone, 0.05 M) and then 1,2-bis(diphenylphosphino)ethane (10 mol%) and $[Rh(CO)_2Cl]_2$ (5 mol%) were added subsequently. The vial was sealed with a silicon/PTFE crimp cap and heated in a pre-heated sand bath at 120 °C for 30 min under nitrogen. The reaction mixture was cooled to room temperature and concentrated *in vacuo*. The crude residue was dissolved in 5% MeOH in CH_2Cl_2, filtered through a pad of celite® (5% MeOH in CH_2Cl_2 to rinse), concentrated *in vacuo*, and the resulting residue was further purified as indicated.

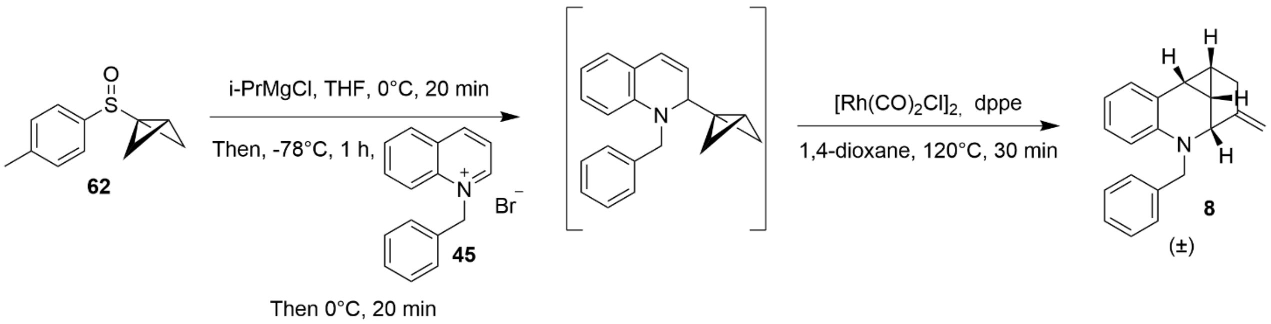

(2a*RS*,2a[1]*SR*,7b*RS*,7c*SR*)-3-Benzyl-2-methylene-2,2a,2a[1],3,7b,7c-hexahydro-1*H*-3-azabenzo[*f*]cyclopropa[*cd*]indene (**8**). Using General Protocol A and **45** (50 mg, 0.17 mmol) as a starting material, purification by chromatography on SiO_2 (100% hexanes to 2% ethyl acetate in hexanes) gave **8** (19.4 mg, 0.07 mmol, 43 %) as a colorless solid: **Mp** 68.2 – 70.8 °C; **IR** (ATR, CH_2Cl_2) 3059, 3027, 2975, 2934, 1498, 1450, 1420, 1340, 1232, 1193, 1163, 1029 cm^{-1}; **^{1}H NMR** (500 MHz, Chloroform-*d*) δ 7.39 (d, *J* = 7.1 Hz, 1 H), 7.34 (t, *J* = 7.6 Hz, 1 H), 7.29 – 7.23 (m, 2 H), 6.98 (ddd, *J* = 8.7, 7.5, 1.7 Hz, 1 H), 6.70 (td, *J* = 7.3, 1.1 Hz, 1 H), 6.49 (d, *J* = 8.2 Hz, 1 H), 4.88 – 4.85 (m, 1 H), 4.73 (d, *J* = 2.0 Hz, 1 H), 4.54 (d, *J* = 15.3 Hz, 1 H), 4.32 – 4.27 (m, 1 H), 4.23 (d, *J* = 15.3 Hz, 1 H), 2.58 – 2.43 (m, 1 H), 2.15 – 2.10 (m, 2 H), 1.87 (dtt, *J* = 17.3, 2.6, 1.2 Hz, 1 H), 1.71 – 1.64 (m, 1 H). **^{13}C NMR** (126 MHz, Chloroform-*d*) δ 155.7, 145.9, 139.0, 130.8, 128.7, 127.7, 127.1, 126.9, 121.9, 117.6, 113.1, 107.9, 63.8, 54.6, 31.5, 23.6, 19.1, 18.3; **HRMS** (ESI) *m/z* calcd for $C_{20}H_{20}N$ $[M + H]^+$, 274.1590; found, 274.1590.

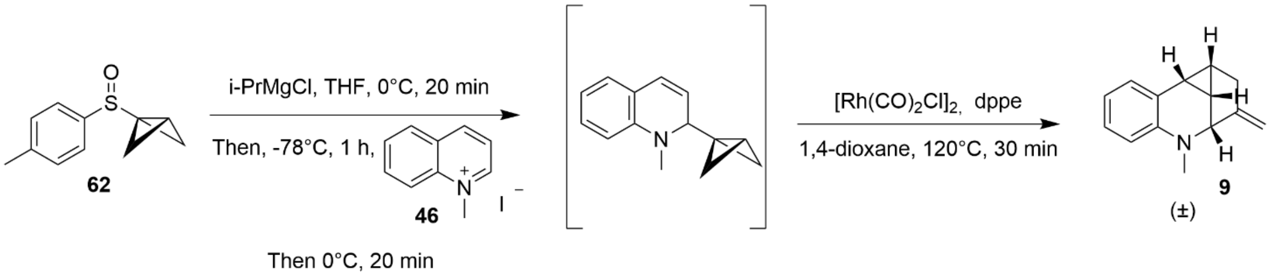

(2a*RS*,2a[1]*SR*,7b*RS*,7c*SR*)-3-Methyl-2-methylene-2,2a,2a[1],3,7b,7c-hexahydro-1*H*-3-azabenzo[*f*]cyclopropa[*cd*]indene (**9**). Using General Protocol A and **46** (0.1 g, 0.37 mmol) as a starting material, purification by chromatography on SiO_2 (2.5 % ethyl acetate in hexanes) gave **9** (36.4 mg, 0.18 mmol, 52%) as a colorless oil: **IR** (ATR, CH_2Cl_2) 3041, 2934, 2865, 2805, 1600, 1495, 1472, 1287, 1257, 1199 cm^{-1}; ^{1}**H NMR** (601 MHz, Chloroform-*d*) δ 7.22 (dd, *J* = 7.4, 1.7 Hz, 1 H), 7.09 (td, *J* = 7.8, 1.7 Hz, 1 H), 6.73 (td, *J* = 7.4, 1.2 Hz, 1 H), 6.53 (dd, *J* = 8.2, 1.1 Hz, 1 H), 4.83 – 4.79 (m, 1 H), 4.73 (p, *J* = 1.5 Hz, 1 H), 4.18 (d, *J* = 6.7 Hz, 1 H), 2.83 (s, 3 H), 2.49 (ddt, *J* = 17.4, 6.2, 2.0 Hz, 1 H), 2.17 (dt, *J* = 8.3, 6.7 Hz, 1 H), 2.11 (t, *J* = 8.3 Hz, 1 H), 1.75 (dtt, *J* = 17.3, 2.7, 1.3 Hz, 1 H), 1.70 (dtd, *J* = 7.9, 6.5, 1.2 Hz, 1 H); ^{13}**C NMR** (151 MHz, Chloroform-*d*) δ 154.5, 146.2, 130.5, 127.1, 121.2, 117.5, 112.0, 107.9, 66.5, 38.5, 31.2, 24.0, 19.1, 18.4; **HRMS** (ESI) *m/z* calcd for $C_{14}H_{16}N$ $[M + H]^+$, 198.1277; found, 198.1280.

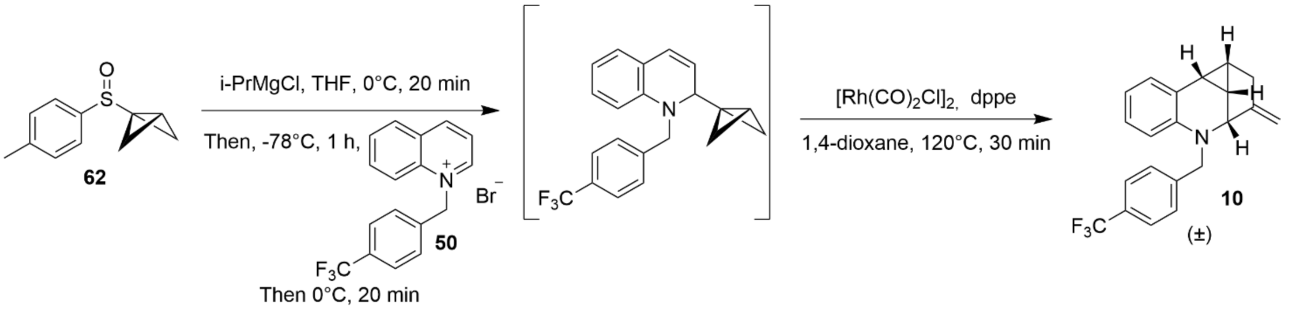

(2a*RS*,2a[1]*SR*,7b*RS*,7c*SR*)-2-Methylene-3-(4-(trifluoromethyl)benzyl)-2,2a,2a[1],3,7b,7c-hexahydro-1*H*-3-azabenzo[*f*]cyclopropa[*cd*]indene (**10**). Using General Protocol A and **50** (60 mg, 0.16 mmol) as a starting material, purification by chromatography on SiO_2 (2% ethyl acetate in hexanes), then reverse phase chromatography (100% H_2O to 100% MeCN) gave **10** (26.3 mg, 0.08 mmol, 53%) as a colorless oil: **IR** (ATR, CH_2Cl_2) 3059, 3027, 2934, 1629, 1450, 1305, 1163, 1128, 1094, 1077, 982, 956 cm^{-1}; ^{1}**H NMR** (500 MHz, Chloroform-*d*) δ 7.59 (d, *J* = 8.1 Hz, 2 H), 7.50 (d, *J* = 8.0 Hz, 2 H), 7.28 – 7.24 (m, 1 H), 6.97 (td, *J* = 7.8, 1.7 Hz, 1 H), 6.72 (td, *J* = 7.3, 1.1 Hz, 1 H), 6.38 (d, *J* = 8.2 Hz, 1 H), 4.86 (d, *J* = 2.7 Hz, 1 H), 4.75 (q, *J* = 1.9 Hz, 1 H), 4.56 (d, *J* = 16.0 Hz, 1 H), 4.31 (d, *J* = 15.9 Hz, 1 H), 4.27 (d, *J* = 5.4 Hz, 1 H), 2.53 (ddt, *J* = 17.3, 6.3, 2.1 Hz, 1 H), 2.17 – 2.14 (m, 2 H), 1.88 (dtd, *J* = 17.3, 2.7, 1.4 Hz, 1 H), 1.71 (q, *J* = 7.0 Hz, 1 H); ^{13}**C NMR** (126 MHz, Chloroform-*d*) δ 155.6, 145.4, 143.4, 130.9, 127.7, 127.5, 127.0, 126.5 (q, *J* = 272.2 Hz), 125.7 (q, *J* = 3.7 Hz), 122.1, 118.1, 113.2, 108.0, 64.8, 54.6, 31.5, 23.7, 19.2, 18.3; ^{19}**F NMR** (471 MHz, Chloroform-*d*) δ -62.35; **HRMS** (ESI) *m/z* calcd for $C_{21}H_{19}NF_3$ $[M + H]^+$, 342.1464; found, 342.1461.

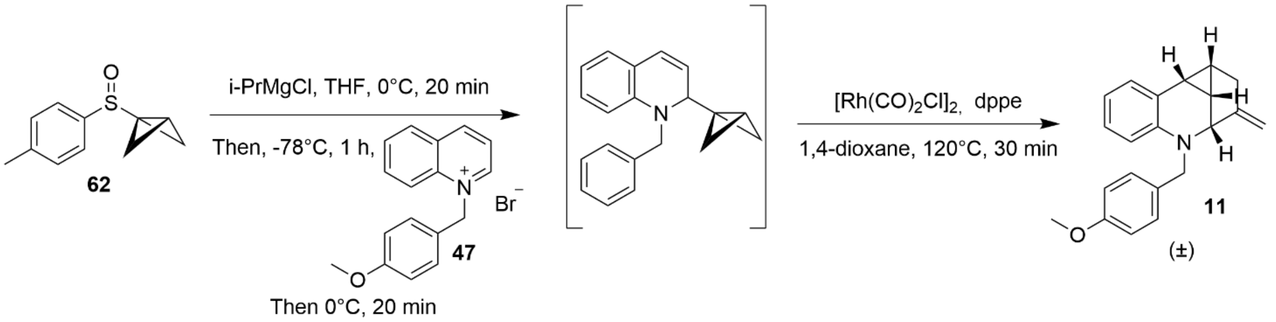

(2a*RS*,2a[1]*SR*,7b*RS*,7c*SR*)-3-(4-Methoxybenzyl)-2-methylene-2,2a,2a[1],3,7b,7c-hexahydro-1*H*-3-azabenzo[*f*]cyclopropa[*cd*]indene (**11**). Using General Protocol A and **47** (50 mg, 0.15 mmol) as a starting material, purification by chromatography on SiO_2 (100% hexanes to 2% ethyl acetate in hexanes) gave **11** (23.7 mg, 0.08 mmol, 52%) as a colorless oil: **IR** (ATR, CH_2Cl_2) 3027, 3059, 2934, 2816, 1549, 1450, 1359, 1232, 1094, 1029, 839 cm^{-1}; **^{1}H NMR** (300 MHz, Chloroform-*d*) δ 7.30 (d, *J* = 8.6 Hz, 2 H), 7.23 (dd, *J* = 7.4, 1.7 Hz, 1 H), 6.99 (td, *J* = 7.8, 1.7 Hz, 1 H), 6.88 (d, *J* = 8.6 Hz, 2 H), 6.69 (td, *J* = 7.4, 1.2 Hz, 1 H), 6.51 (d, *J* = 8.2 Hz, 1 H), 4.85 – 4.82 (m, 1 H), 4.73 – 4.69 (m, 1 H), 4.48 (d, *J* = 14.9 Hz, 1 H), 4.27 (d, *J* = 5.7 Hz, 1 H), 4.13 (d, *J* = 14.9 Hz, 1 H), 3.81 (s, 3 H), 2.49 (dd, *J* = 17.3, 6.2 Hz, 1 H), 2.14 – 2.03 (m, 2 H), 1.85 (d, *J* = 17.3 Hz, 1 H), 1.65 (q, *J* = 7.0 Hz, 1 H); **^{13}C NMR** (126 MHz, Chloroform-*d*) δ 158.8, 155.7, 146.1, 130.9, 130.8, 128.9, 126.9, 122.0, 117.6, 114.2, 113.1, 107.8, 63.3, 55.4, 53.8, 31.6, 29.9, 23.5, 19.1, 18.3; **HRMS** (ESI) *m/z* calcd for $C_{21}H_{22}NO$ $[M + H]^+$, 304.1696; found, 304.1677.

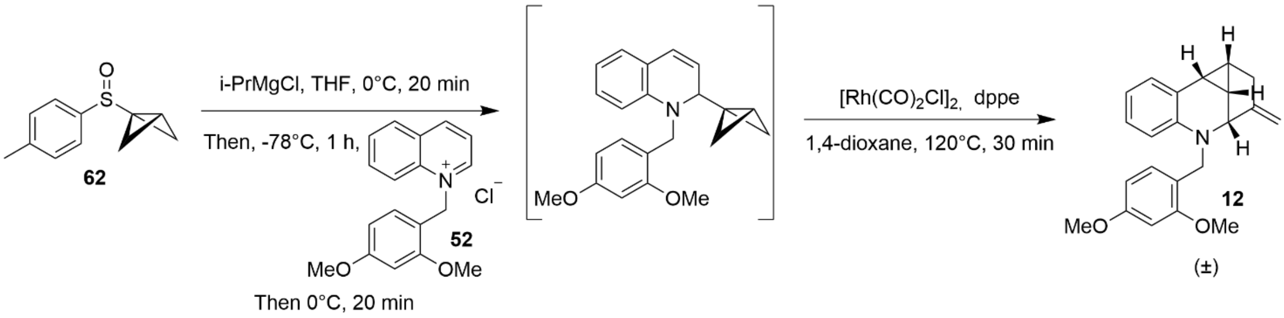

(2a*RS*,2a[1]*SR*,7b*RS*,7c*SR*)-3-(2,4-Dimethoxybenzyl)-2-methylene-2,2a,2a[1],3,7b,7c-hexahydro-1*H*-3-azabenzo[*f*]cyclopropa[*cd*]indene (**12**). Using General Protocol A and **52** (0.1 g, 0.19 mmol) as a starting material, purification by reverse phase chromatography on C18-SiO_2 (100% H_2O to 100% MeCN) followed by chromatography on SiO_2 (4% ethyl acetate in hexanes) gave **12** (28.1 mg, 0.08 mmol, 45%) as a colorless solid: **Mp** 94.8 – 97.4 °C; **IR** (ATR, CH_2Cl_2) 3030, 2929, 2886, 1578, 1569, 1491, 1450, 1295, 1251, 1196, 1029 cm^{-1}; **^{1}H NMR** (601 MHz, Chloroform-*d*) δ 7.24 – 7.19 (m, 2 H), 6.99 – 6.94 (m, 1 H), 6.65 (tt, *J* = 7.4, 1.2 Hz, 1 H), 6.48 (t, *J* = 1.8 Hz, 1 H), 6.43 – 6.41 (m, 2 H), 4.97 – 4.94 (m, 1 H), 4.73 – 4.71 (m, 1 H), 4.46 (d, *J* = 16.2 Hz, 1 H), 4.32 – 4.28 (m, 1 H), 4.20 (d, *J* = 16.2 Hz, 1 H), 3.85 (s, 2 H), 3.79 (s, 3 H), 2.51 (dd, *J* = 17.2, 6.0 Hz, 1 H), 2.12 – 2.07 (m, 2H), 1.89 (dtd, *J* = 17.1, 2.6, 1.3 Hz, 1 H), 1.66 (q, *J* = 7.0 Hz, 1 H); **^{13}C NMR** (151 MHz, Chloroform-*d*) δ 159.9, 158.1, 156.0, 145.9, 130.7, 129.2, 126.9, 121.4, 119.0, 117.0, 112.9, 107.8, 104.2, 98.5, 64.5, 55.5, 55.4, 48.8, 31.5, 23.5, 19.0, 18.4; **HRMS** (ESI) *m/z* calcd for $C_{22}H_{24}NO_2$ $[M + H]^+$, 334.1802; found, 334.1795.

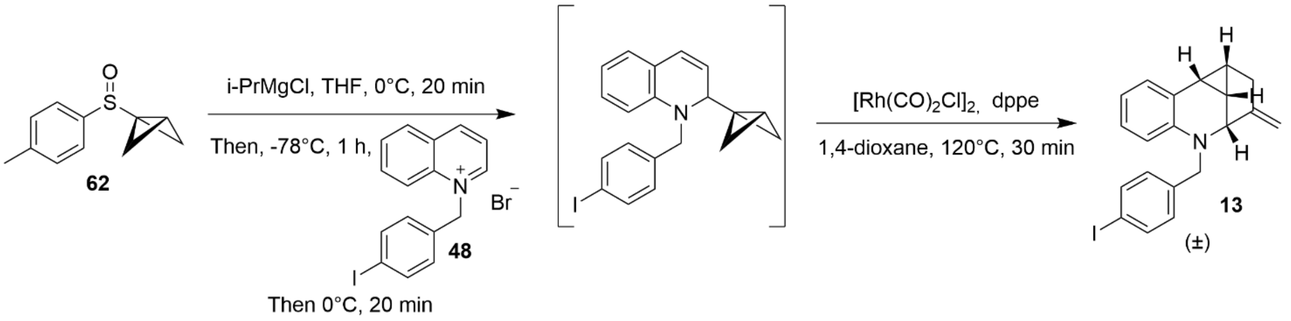

(2a*RS*,2a[1]*SR*,7b*RS*,7c*SR*)-3-(4-Iodobenzyl)-2-methylene-2,2a,2a[1],3,7b,7c-hexahydro-1*H*-3-azabenzo[*f*]cyclopropa[*cd*]indene (**13**). Using General Protocol A and **48** (70 mg, 0.16 mmol) as a starting material, purification by chromatography on SiO_2 (100 % hexanes to 4 % Et_2O in hexanes) gave **13** (13.2 mg, 0.03 mmol, 22%) as a colorless oil: **IR** (ATR, CH_2Cl_2) 3027, 3059, 2975, 2934, 1050, 1420, 1358, 1340, 1163, 1094, 1077, 956 cm^{-1}; **^{1}H NMR** (300 MHz, Chloroform-*d*) δ 7.67 – 7.63 (m, 2 H), 7.24 (dd, *J* = 7.4, 1.7 Hz, 1 H), 7.14 (d, *J* = 8.2 Hz, 2 H), 7.01 – 6.93 (m, 1 H), 6.71 (td, *J* = 7.4, 1.2 Hz, 1 H), 6.40 (d, *J* = 8.1 Hz, 1 H), 4.84 (d, *J* = 2.7 Hz, 1 H), 4.73 (q, *J* = 2.0 Hz, 1 H), 4.46 (d, *J* = 15.6 Hz, 1 H), 4.26 (d, *J* = 4.7 Hz, 1 H), 4.18 (d, *J* = 15.6 Hz, 1 H), 2.51 (ddt, *J* = 17.4, 6.6, 2.1 Hz, 1 H), 2.16 – 2.09 (m, 2 H), 1.86 (dtt, *J* = 17.3, 2.6, 1.2 Hz, 1 H), 1.74 – 1.64 (m, 1 H); **^{13}C NMR** (151 MHz, Chloroform-*d*) δ 155.6, 145.6, 138.9, 137.8, 130.8, 129.6, 126.9, 122.0, 117.9, 113.2, 108.0, 92.2, 64.4, 54.3, 31.5, 23.7, 19.2, 18.3; **HRMS** (ESI) *m/z* calcd for $C_{20}H_{19}NI$ $[M + H]^+$, 400.0557; found, 400.0558.

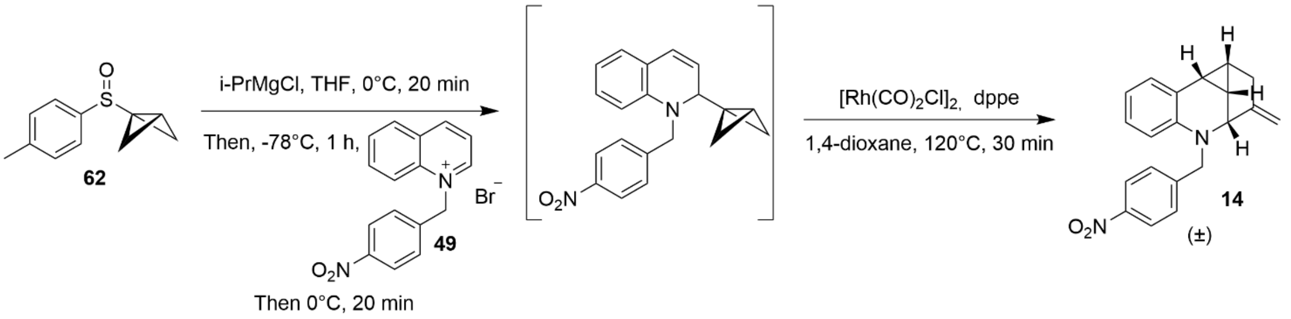

(2a*RS*,2a[1]*SR*,7b*RS*,7c*SR*)-2-Methylene-3-(4-nitrobenzyl)-2,2a,2a[1],3,7b,7c-hexahydro-1*H*-3-azabenzo[*f*]cyclopropa[*cd*]indene (**14**). Using General Protocol A and **49** (50 mg, 0.14 mmol) as a starting material, purification by chromatography on SiO_2 (2% ethyl acetate in hexanes); gave **14** (15.4 mg, 0.05 mmol, 34%) as a yellow solid: **Mp** 57.0 - 59.4 °C; **IR** (ATR, CH_2Cl_2) 3059, 3027, 2816, 1690, 1450, 1420, 1359, 1340, 1232, 1163, 839 cm^{-1}; **^{1}H NMR** (601 MHz, Chloroform-*d*) δ 8.19 (d, *J* = 8.7 Hz, 2 H), 7.55 (d, *J* = 8.4 Hz, 2 H), 7.29 – 7.22 (m, 1 H), 6.94 (td, *J* = 7.8, 1.7 Hz, 1 H), 6.73 (td, *J* = 7.4, 1.1 Hz, 1 H), 6.28 (d, *J* = 8.2 Hz, 1 H), 4.87 (d, *J* = 2.7 Hz, 1 H), 4.76 (q, *J* = 1.9 Hz, 1 H), 4.57 (d, *J* = 16.5 Hz, 1 H), 4.37 (d, *J* = 16.5 Hz, 1 H), 4.29 (d, *J* = 6.6 Hz, 1 H), 2.54 (dd, *J* = 17.3, 6.2 Hz, 1 H), 2.24 – 2.12 (m, 2 H), 1.88 (ddq, *J* = 17.3, 2.5, 1.3 Hz, 1 H), 1.77 – 1.68 (m, 1 H); **^{13}C NMR** (151 MHz, Chloroform-*d*) δ 155.5, 147.3, 147.2, 145.0, 131.0, 128.0, 126.9, 124.0, 122.2, 118.4, 113.3, 108.2, 65.6, 54.9, 31.4, 23.8, 19.3, 18.3; **HRMS** (ESI) *m/z* calcd for $C_{20}H_{17}N_2O_2$ $[M + H]^+$, 317.1284; found, 317.1288.

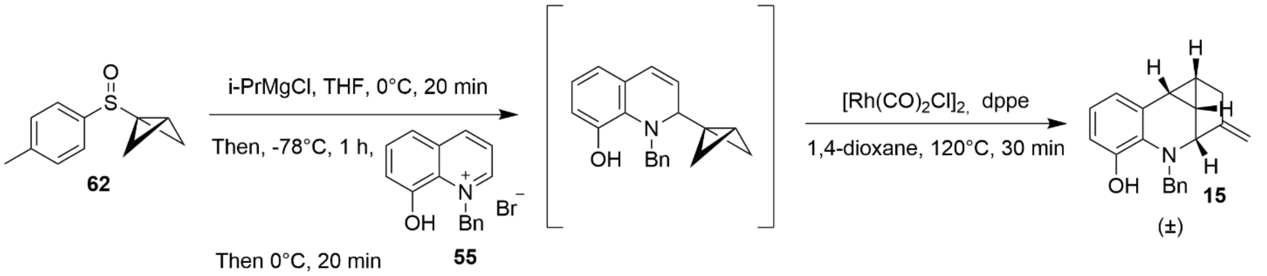

(2a*RS*,2a[1]*SR*,7b*RS*,7c*SR*)-3-Benzyl-2-methylene-2,2a,2a[1],3,7b,7c-hexahydro-1*H*-3-azabenzo[*f*]cyclopropa[*cd*]inden-4-ol (**15**). Using General Protocol A and **55** (50 mg, 0.16 mmol) as a starting material, purification by chromatography on SiO_2 (2% ethyl acetate in hexanes), followed by a second chromatography on SiO_2 (100% CH_2Cl_2) gave **15** (15 mg, 0.05 mmol, 33%) as a colorless oil: **IR** (ATR, CH_2Cl_2) 3047, 2932, 2856, 1589, 1237, 1129, 1077, 982, 956 cm^{-1}; **^{1}H NMR** (601 MHz, Benzene-d_6) δ 7.25 – 7.21 (m, 2 H), 7.14 – 7.07 (m, 2 H), 6.98 (dd, *J* = 8.0, 1.5 Hz, 2 H), 6.93 (t, *J* = 7.8 Hz, 1 H), 6.84 (dd, *J* = 7.5, 1.5 Hz, 1 H), 6.68 (br s, 1 H), 4.95 – 4.89 (m, 1 H), 4.58 – 4.54 (m, 1 H), 4.09 – 4.03 (m, 1 H), 3.86 (d, *J* = 13.8 Hz, 1 H), 3.73 (d, *J* = 13.7 Hz, 1 H), 2.27 (ddt, *J* = 17.7, 5.6, 2.6 Hz, 1 H), 1.99 (dq, *J* = 17.6, 2.2 Hz, 1 H), 1.76 (t, *J* = 8.0 Hz, 1 H), 1.53 – 1.45 (m, 1 H), 1.25 (dt, *J* = 8.4, 6.2 Hz, 1 H); **^{13}C NMR** (151 MHz, Benzene-d_6) δ 151.7, 138.6, 135.1, 129.6, 129.0, 128.9, 125.6, 121.9, 112.6, 109.6, 62.3, 59.5, 31.2, 24.2, 17.3, 16.5; **HRMS** (ESI) *m/z* calcd for $C_{20}H_{20}NO$ [M + H]$^+$, 290.1539; found, 290.1537.

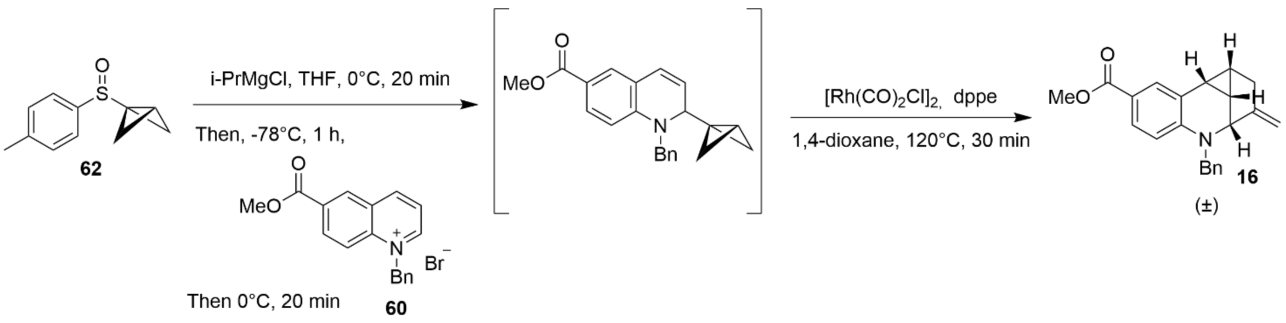

Methyl (2a*RS*,2a[1]*SR*,7b*RS*,7c*SR*)-3-benzyl-2-methylene-2,2a,2a1,3,7b,7c-hexahydro-1*H*-3-azabenzo[*f*]cyclopropa[*cd*]indene-6-carboxylate (**16**). Using General Protocol A and **60** (50 mg, 0.14 mmol) as a starting material, purification by reverse phase chromatography on C18-SiO_2 (100% H_2O to 100% MeCN) followed by chromatography on SiO_2 (5% ethyl acetate in hexanes) gave **16** (17.6 mg, 0.05 mmol, 38%) as a colorless oil: **IR** (ATR, CH_2Cl_2) 3027, 2934, 2929, 2851, 1700, 1601, 1504, 1450, 1447, 1271, 1193, 1163, 1125 cm^{-1}; **^{1}H NMR** (601 MHz, Chloroform-*d*) δ 7.95 – 7.93 (m, 1 H), 7.66 (dt, *J* = 8.7, 1.7 Hz, 1 H), 7.37 – 7.30 (m, 5 H), 6.47 (d, *J* = 8.7 Hz, 1 H), 4.89 – 4.86 (m, 1 H), 4.75 – 4.72 (m, 1 H), 4.65 (d, *J* = 15.7 Hz, 1 H), 4.35 – 4.30 (m, 2 H), 3.84 (s, 2 H), 2.52 (dd, *J* = 17.3, 6.2 Hz, 1 H), 2.19 (t, *J* = 8.3 Hz, 1 H), 2.11 (dt, *J* = 8.7, 6.6 Hz, 1 H), 1.86 (ddq, *J* = 17.3, 2.7, 1.4 Hz, 1 H), 1.76 – 1.71 (m, 1 H); ^{13}C NMR (151 MHz, Chloroform-*d*) δ 167.6, 155.6, 149.4, 137.9, 132.5, 129.2, 128.9, 127.4, 126.7, 120.5, 118.4, 111.8, 108.1, 64.0, 54.2, 51.7, 31.2, 23.9, 18.5, 18.5; **HRMS** (ESI) *m/z* calcd for $C_{22}H_{22}NO_2$ [M + H]$^+$, 332.1645; found, 332.1651.

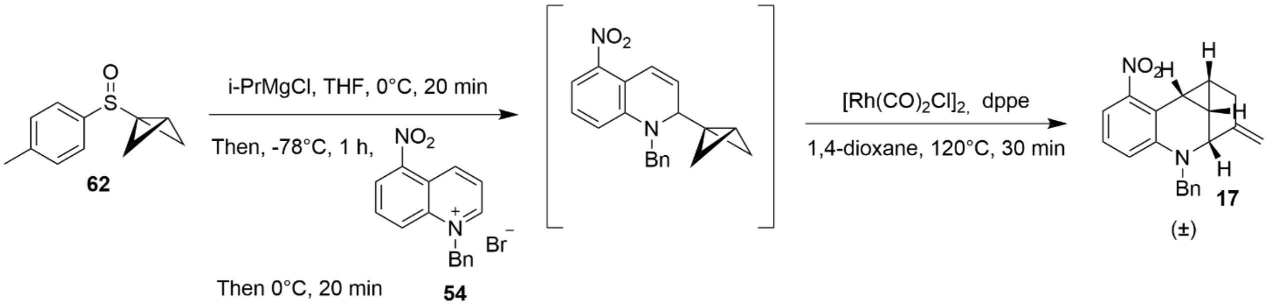

(2a*RS*,2a[1]*SR*,7b*RS*,7c*SR*)-3-Benzyl-2-methylene-7-nitro-2,2a,2a[1],3,7b,7c-hexahydro-1*H*-3-azabenzo[*f*]cyclopropa[*cd*]indene (**17**). Using General Protocol A and **54** (50 mg, 0.14 mmol) as a starting material, purification by reverse phase chromatography on C18-SiO_2 (100% H_2O to 100% MeCN) followed by chromatography on SiO_2 (5% ethyl acetate in hexanes) gave **17** (23.1 mg, 0.07 mmol, 50%) as a yellow oil: **IR** (ATR, CH_2Cl_2) 3352, 3027, 2929, 2986, 1501, 1486, 1450, 1340, 1291, 1128, 1077, 7989 cm^{-1}; **^{1}H NMR** (601 MHz, Methanol-d_4) δ 7.38 (d, *J* = 7.2 Hz, 2 H), 7.35 – 7.32 (m, 2 H), 7.27 – 7.23 (m, 1 H), 7.18 (dd, *J* = 8.1, 1.1 Hz, 1 H), 7.06 (t, *J* = 8.2 Hz, 1 H), 6.73 (d, *J* = 8.2 Hz, 1 H), 4.99 (q, *J* = 2.2 Hz, 1 H), 4.78 (q, *J* = 2.1 Hz, 1 H), 4.61 (d, *J* = 15.6 Hz, 1 H), 4.53 – 4.51 (m, 1 H), 4.49 (d, *J* = 15.6 Hz, 1 H), 2.61 (ddt, *J* = 17.6, 6.1, 2.4 Hz, 1 H), 2.28 – 2.22 (m, 2 H), 1.93 (ddt, *J* = 17.6, 3.3, 2.0 Hz, 1 H), 1.82 (q, *J* = 6.8 Hz, 1 H); **^{13}C NMR** (151 MHz, Methanol-d_4) δ 156.1, 153.3, 148.6, 139.6, 129.7, 128.4, 128.3, 127.7, 118.5, 118.2, 113.6, 109.6, 65.6, 56.7, 32.7, 24.4, 20.7, 17.3; **HRMS** (ESI) *m/z* calcd for $C_{20}H_{19}N_2O_2$ [M + H]$^+$, 319.1441; found, 319.1442.

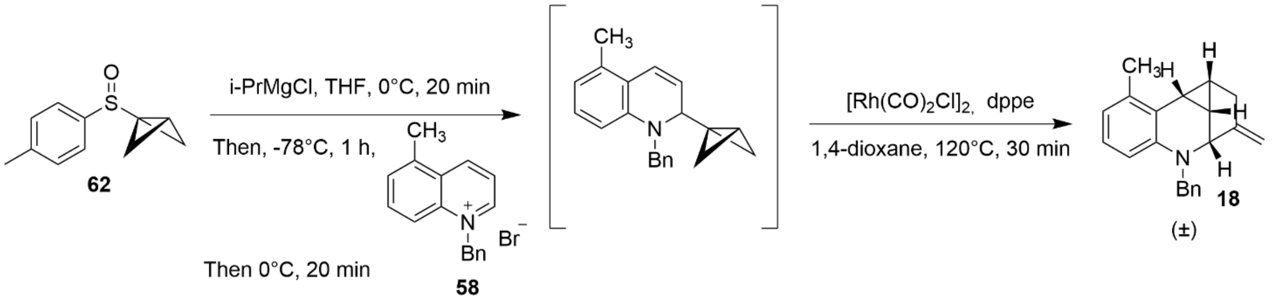

(2a*RS*,2a[1]*SR*,7b*RS*,7c*SR*)-3-Benzyl-7-methyl-2-methylene-2,2a,2a[1],3,7b,7c-hexahydro-1*H*-3-azabenzo[*f*]cyclopropa[*cd*]indene (**18**). Using General Protocol A and **58** (50 mg, 0.16 mmol) as a starting material, purification by reverse phase chromatography on C18-SiO_2 (100% H_2O to 100% MeCN) followed by preparative TLC on SiO_2 (2% ethyl acetate in hexanes) gave **18** (11.6 mg, 0.04 mmol, 25%) as a colorless oil: **IR** (ATR, CH_2Cl_2) 3043, 2931, 2862, 1584, 1474, 1450, 1267, 1152, 1089 cm^{-1}; **^{1}H NMR** (601 MHz, Chloroform-*d*) δ 7.40 (d, *J* = 7.2 Hz, 2 H), 7.34 (t, *J* = 7.6 Hz, 2 H), 7.27 – 7.24 (m, 1 H), 6.91 (t, *J* = 7.8 Hz, 1 H), 6.60 (d, *J* = 7.5 Hz, 1 H), 6.39 (d, *J* = 8.2 Hz, 1 H), 4.87 (d, *J* = 2.8 Hz, 1 H), 4.71 (q, *J* = 2.0 Hz, 1 H), 4.54 (d, *J* = 15.2 Hz, 1 H), 4.30 (dd, *J* = 6.8, 1.5 Hz, 1 H), 4.24 (d, *J* = 15.2 Hz, 1 H), 2.50 (ddt, *J* = 17.3, 6.1, 2.2 Hz, 1 H), 2.32 (s, 3 H), 2.14 (dt, *J* = 8.3, 6.6 Hz, 1 H), 1.93 (t, *J* = 8.2 Hz, 1 H), 1.82 – 1.76 (m, 1 H), 1.73 (dt, *J* = 8.1, 6.2 Hz, 1 H); **^{13}C NMR** (151 MHz, Chloroform-*d*) δ 155.7, 146.3, 139.3, 138.5, 128.7, 127.7, 127.0, 126.4, 120.9, 119.4, 111.3, 108.2, 63.8, 55.2, 31.9, 21.9, 19.8, 19.6, 16.5; **HRMS** (ESI) *m/z* calcd for $C_{21}H_{22}N$ [M + H]$^+$, 288.1747; found, 288.1747.

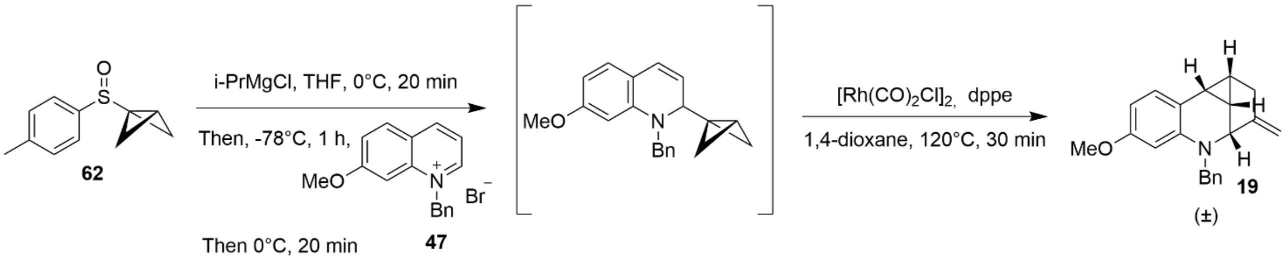

(2a*RS*,2a¹*SR*,7b*RS*,7c*SR*)-3-Benzyl-5-methoxy-2-methylene-2,2a,2a1,3,7b,7c-hexahydro-1*H*-3-azabenzo[*f*]cyclopropa[*cd*]indene (**19**). Using General Protocol A and **47** (50 mg, 0.15 mmol) as a starting material, purification by reverse phase chromatography on C18-SiO_2 (100% H_2O to 100% MeCN) followed by preparative TLC on SiO_2 (2% ethyl acetate in hexanes) gave **19** (15.9 mg, 0.05 mmol, 35%) as a colorless oil: **IR** (ATR, CH_2Cl_2) 3050, 3014, 2954, 2848, 1616, 1506, 1463, 1450, 1422, 1342, 1266, 1204, 1161 cm^{-1}; **^{1}H NMR** (601 MHz, Chloroform-*d*) δ 7.42 – 7.37 (m, 2 H), 7.34 (dd, *J* = 8.5, 6.8 Hz, 2 H), 7.32 – 7.29 (m, 1 H), 7.15 (d, *J* = 8.2 Hz, 1 H), 6.28 (dd, *J* = 8.2, 2.5 Hz, 1 H), 6.10 (d, *J* = 2.5 Hz, 1 H), 4.87 – 4.85 (m, 1 H), 4.75 – 4.73 (m, 1 H), 4.52 (d, *J* = 15.2 Hz, 1 H), 4.29 – 4.26 (m, 1 H), 4.22 (d, *J* = 15.2 Hz, 1 H), 3.66 (s, 3 H), 2.49 (ddt, *J* = 17.2, 6.3, 2.1 Hz, 1 H), 2.11 – 2.04 (m, 2 H), 1.89 (dtt, *J* = 17.3, 2.6, 1.2 Hz, 1 H), 1.66 – 1.60 (m, 1 H); **^{13}C NMR** (151 MHz, Chloroform-*d*) δ 158.9, 155.7, 146.9, 138.8, 131.2, 128.7, 127.7, 127.1, 114.3, 107.8, 102.3, 100.1, 63.7, 55.1, 54.6, 31.4, 23.3, 19.0, 17.7; **HRMS** (ESI) *m/z* calcd for $C_{21}H_{22}NO$ $[M + H]^+$, 304.1696; found, 304.1695.

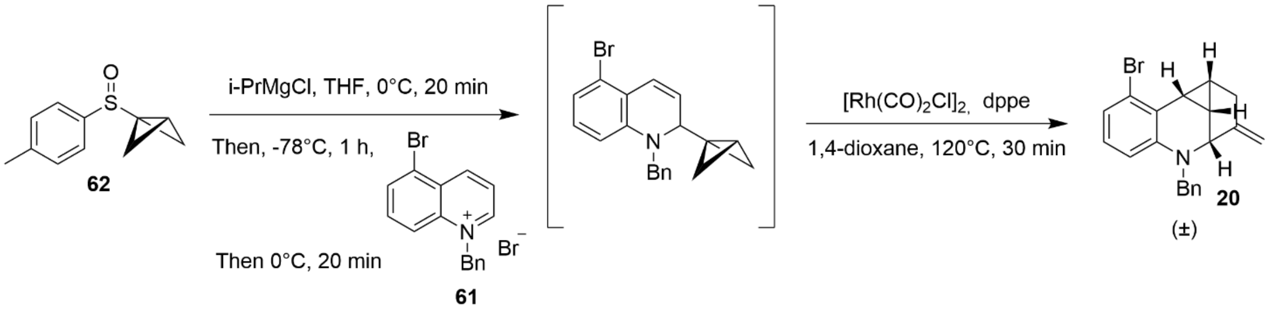

(2a*RS*,2a¹*SR*,7b*RS*,7c*SR*)-3-Benzyl-7-bromo-2-methylene-2,2a,2a¹,3,7b,7c-hexahydro-1*H*-3-azabenzo[*f*]cyclopropa[*cd*]indene (**20**). Using General Protocol A and **61** (60 mg, 0.16 mmol) as a starting material, purification by reverse phase chromatography on C18-SiO_2 (100% H_2O to 100% MeCN) followed by chromatography on SiO_2 (2% ethyl acetate in hexanes) gave **20** (20.1 mg, 0.06 mmol, 36%) as a colorless oil: **IR** (ATR, CH_2Cl_2) 3053, 2916, 2849, 1587, 1558, 1471, 1447, 1264 cm^{-1}; **^{1}H NMR** (700 MHz, Benzene-d_6) δ 7.25 – 7.20 (m, 2 H), 7.19 – 7.16 (m, 4 H), 7.10 – 7.07 (m, 1 H), 7.02 (dd, *J* = 8.4, 3.8 Hz, 1 H), 6.59 (q, *J* = 7.4 Hz, 1 H), 6.35 (dd, *J* = 9.3, 3.7 Hz, 1 H), 4.71 (s, 1 H), 4.55 (d, *J* = 3.7 Hz, 1 H), 4.27 (dd, *J* = 15.4, 3.9 Hz, 1 H), 4.00 (t, *J* = 5.3 Hz, 1 H), 3.94 (dd, *J* = 15.3, 3.9 Hz, 1 H), 2.26 (ddt, *J* = 17.6, 6.1, 2.9 Hz, 1 H), 2.08 (dt, *J* = 8.1, 5.3 Hz, 1 H), 1.83 (d, *J* = 17.5 Hz, 1 H), 1.74 (dq, *J* = 12.9, 6.8, 6.0 Hz, 1 H), 1.43 (dq, *J* = 10.4, 5.9 Hz, 1 H); **^{13}C NMR** (176 MHz, Benzene-d_6) δ 155.4, 148.0, 138.8, 128.9, 127.6, 127.4, 122.6, 121.9, 112.9, 108.5, 64.6, 55.3, 31.8, 23.0, 20.6, 20.3; **HRMS** (ESI) *m/z* calcd for $C_{20}H_{19}NBr$ $[M + H]^+$, 352.0695; found, 352.0699.

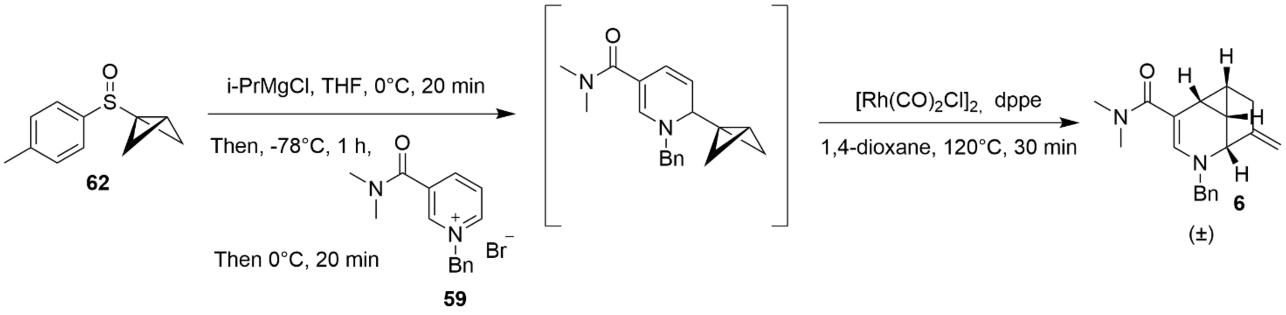

(2a*SR*,2a^1*SR*,2b*R*,5a*RS*)-5-Benzyl-N,N-dimethyl-1-methylene-2,2a,2a^1,2b,5,5a-hexahydro-1*H*-5-azacyclopropa[*cd*]indene-3-carboxamide (**6**). Using General Protocol A and **59** (50 mg, 0.16 mmol) as a starting material, purification by chromatography on SiO_2 (45% hexanes in ethyl acetate) gave **6** (30.2 mg, 0.10 mmol, 66%) as a brownish oil. A sample was repurified on RP-C18 silica with a MeCN/H_2O (10-100%) gradient and dried by lyophilization: **IR** (ATR, CH_2Cl_2) 3048, 2975, 2934, 1629, 1592, 1376, 1493, 1232, 1163, 1128, 1077 cm^{-1}; **^{1}H NMR** (601 MHz, Chloroform-*d*) δ 7.34 (dd, *J* = 7.9, 6.5 Hz, 1 H), 7.31 – 7.26 (m, 2 H), 6.66 (s, 1 H), 4.81 (dd, *J* = 2.9, 1.5 Hz, 1 H), 4.55 (dd, *J* = 2.5, 1.3 Hz, 1 H), 4.41, 4.37 (ABq, *J* = 15.1 Hz, 2H), 3.71 (d, *J* = 6.6 Hz, 1 H), 2.97 (s, 3 H), 2.66 (dd, *J* = 8.0, 6.7 Hz, 1 H), 2.36 (ddd, *J* = 16.7, 6.7, 1.4 Hz, 1 H), 1.79 (dp, *J* = 17.0, 2.3 Hz, 1 H), 1.73 (q, *J* = 7.4 Hz, 1 H), 1.35 (qd, *J* = 6.9, 1.8 Hz, 1 H); **^{13}C NMR** (151 MHz, Chloroform-*d*) δ 173.3, 159.5, 139.2, 138.0, 128.8, 127.9, 127.8, 104.6, 104.2, 59.1, 39.7, 38.8, 38.4, 29.1, 21.5, 16.3; **HRMS** (ESI) *m/z* calcd for $C_{19}H_{23}N_2O$ [M + H]$^+$, 295.1805; found, 295.1807.

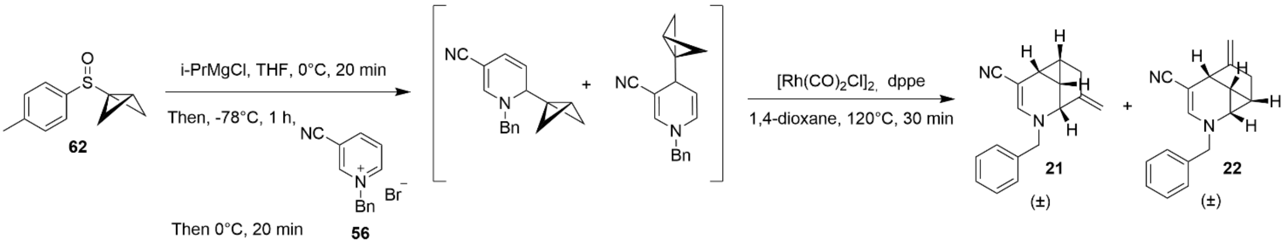

(2a*SR*,2a^1*SR*,2b*RS*,5a*RS*)-5-Benzyl-1-methylene-2,2a,2a^1,2b,5,5a-hexahydro-1*H*-5-azacyclopropa[*cd*]indene-3-carbonitrile (**21**) and **(2a*SR*,2a^1*RS*,2b*SR*,5a*SR*)-3-benzyl-1-methylene-2,2a,2a^1,2b,3,5a-hexahydro-1*H*-3-azacyclopropa[*cd*]indene-5-carbonitrile** (**22**). Using General Protocol A and **56** (50 mg, 0.18 mmol) as a starting material, purification by chromatography on SiO_2 (15% ethyl acetate in hexanes) gave **21** (29.1 mg, 0.12 mmol, 64%) as a pale-yellow oil and **22** (7.2 mg, 0.03 mmol, 16%) as a pale-yellow solid.

21: R_f = 0.33 (15% ethyl acetate in hexanes); **IR** (ATR, CH_2Cl_2) 3028, 2927, 2184, 1618, 1420, 1365, 1273, 1193, 1128, 796 cm^{-1}; **^{1}H NMR** (500 MHz, Chloroform-*d*) δ 7.38 (dd, *J* = 8.0, 6.5 Hz, 2 H), 7.33 (t, *J* = 7.3 Hz, 1 H), 7.25 – 7.20 (m, 2H), 6.89 (s, 1 H), 4.86 – 4.79 (m, 2 H), 4.30 (d, *J* = 15.0 Hz, 2 H), 4.26 (d, *J* = 15.0 Hz, 2 H), 3.87 (d, *J* = 5.6 Hz, 1 H), 2.54 – 2.46 (m, 1 H), 1.93 (dq, *J* = 17.5, 2.4 Hz, 1 H), 1.81 – 1.68 (m, 3 H); **^{13}C NMR** (126 MHz, Chloroform-*d*) δ 154.4, 147.2, 136.2, 129.1, 128.2, 128.1, 123.4, 108.2, 72.2, 59.7, 57.4, 29.6, 25.0, 17.5, 14.6; **HRMS** (ESI) *m/z* calcd for $C_{17}H_{17}N_2$ [M + H]$^+$, 249.1386; found, 249.1388.

22: Rf = 0.22 (15% ethyl acetate in hexanes); **Mp** 94.0 - 96.5 °C; **IR** (ATR, CH_2Cl_2) 3053, 2934, 2855, 2278, 2184, 1625, 1450, 1391, 1278, 1260, 1163, 1009, 917, 839 cm^{-1}; **^{1}H NMR** (500 MHz, Chloroform-*d*) δ 7.40 – 7.31 (m, 3 H), 7.28 – 7.26 (m, 1 H), 7.26 – 7.24 (m, 1 H), 6.94 (s, 1 H), 5.19 – 4.75 (m, 1 H), 4.75 – 4.56 (m, 1 H), 4.43 (d, *J* = 15.0 Hz, 1 H), 4.40 (d, *J* = 10.0 Hz, 1 H) 3.27 (d, *J* = 6.2 Hz, 1 H), 2.73 – 2.68 (m, 1 H), 2.34 (ddd, *J* = 16.9, 6.9, 1.4 Hz, 1 H), 1.75 (td, *J* = 7.8, 6.6 Hz, 1 H), 1.71 – 1.62 (m, 1 H), 1.48 (qd, *J* = 7.0, 2.1 Hz, 1 H); **^{13}C NMR** (126 MHz, Chloroform-*d*) δ 157.5, 144.7, 136.4, 129.0, 128.4, 128.1, 122.7, 105.2, 79.3, 59.6, 39.1, 38.9, 28.0, 23.3, 15.4; **HRMS** (ESI) *m/z* calcd for $C_{17}H_{17}N_2$ [M + H]$^+$, 249.1386; found, 249.1390.

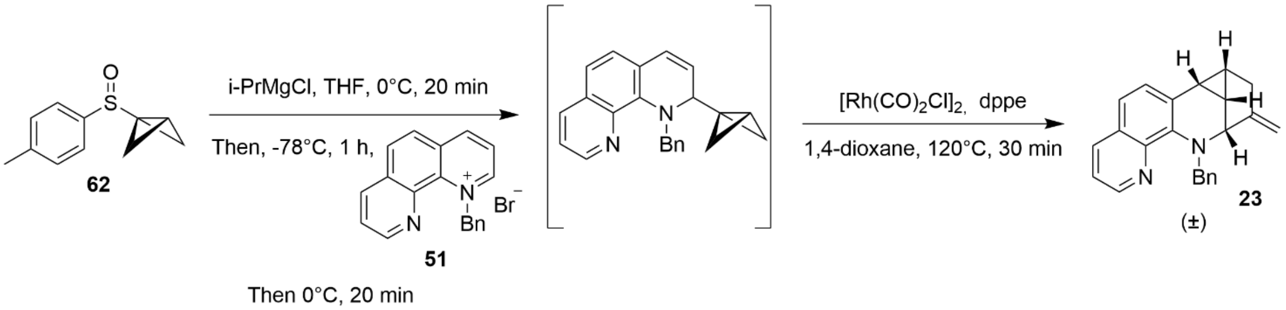

(2a*RS*,2a1*SR*,9b*RS*,9c*SR*)-3-Benzyl-2-methylene-2,2a,2a1,3,9b,9c-hexahydro-1*H*-3,4-diazacyclopropa[7,1]indeno[5,6-*a*]naphthalene (**23**). Using General Protocol A and **51** (60 mg, 0.17 mmol) as a starting material, purification by chromatography on SiO_2 (5% ethyl acetate in hexanes) gave **23** (22.1 mg, 0.07 mmol, 41%) as a yellow solid: **Mp** 81.3 – 83.4 °C; **IR** (ATR, CH_2Cl_2) 3027, 2975, 2930, 1496, 1450, 1358, 1215, 1193 cm^{-1}; **^{1}H NMR** (601 MHz, Chloroform-*d*) δ 8.95 – 8.87 (m, 1 H), 8.07 (d, *J* = 8.1 Hz, 1 H), 7.51 (d, *J* = 7.4 Hz, 2 H), 7.45 (d, *J* = 8.2 Hz, 1 H), 7.38 (d, *J* = 8.3 Hz, 1 H), 7.34 – 7.28 (m, 3 H), 7.28 – 7.24 (m, 1 H), 5.12 – 5.04 (m, 1 H), 4.88 (d, *J* = 14.2 Hz, 1 H), 4.75 (dd, *J* = 14.1, 1.4 Hz, 1 H), 4.62 (d, *J* = 2.6 Hz, 1 H), 4.45 – 4.38 (m, 1 H), 2.56 – 2.49 (m, 1 H), 2.06 (dt, *J* = 17.6, 2.1 Hz, 1 H), 1.89 (t, *J* = 8.0 Hz, 1 H), 1.61 – 1.51 (m, 2 H); **^{13}C NMR** (151 MHz, Chloroform-*d*) δ 154.7, 148.7, 148.5, 143.9, 142.7, 140.7, 136.4, 129.6, 129.5, 128.2, 128.1, 127.1, 120.8, 120.3, 110.1, 62.7, 59.9, 31.0, 22.1, 18.5, 14.8; **HRMS** (ESI) *m/z* calcd for $C_{23}H_{21}N_2$ $[M + H]^+$, 325.1699; found, 325.1707.

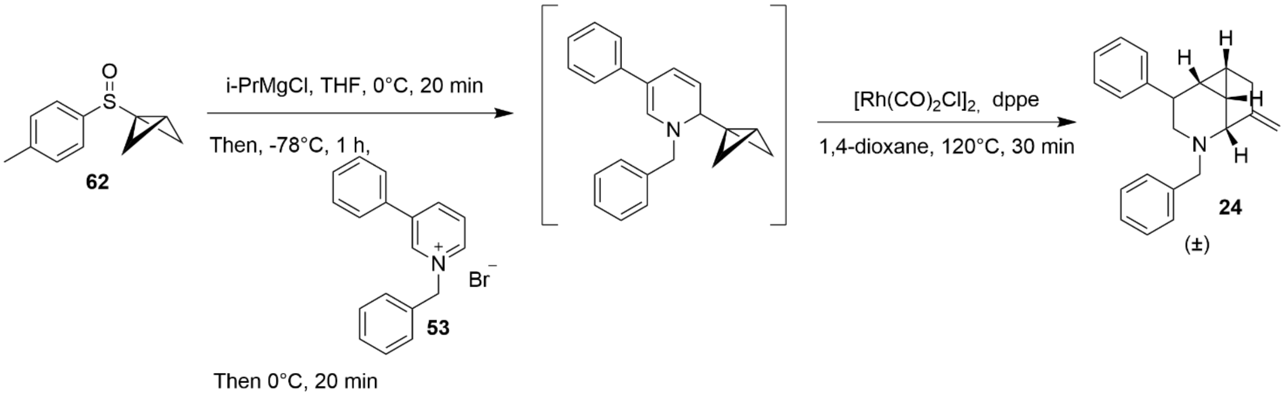

(2a*SR*,2a1*SR*,2b*RS*,5a*RS*)-5-Benzyl-1-methylene-3-phenyl-2,2a,2a1,2b,5,5a-hexahydro-1*H*-5-azacyclopropa[*cd*]indene (**24**). Using General Protocol A and **53** (50 mg, 0.15 mmol) as a starting material, purification by reverse phase chromatography on C18-SiO_2 (100% H_2O to 100% MeCN) gave **24** (7.2 mg, 0.02 mmol, 16%) as a colorless oil: **IR** (ATR, CH_2Cl_2) 3027, 2910, 2854, 1631, 1617, 1450, 1211, 1164, 894 cm^{-1}; **^{1}H NMR** (601 MHz, Chloroform-*d*) δ 7.41 – 7.31 (m, 7 H), 7.30 – 7.28 (m, 1 H), 7.27 – 7.26 (m, 1 H), 7.06 – 7.01 (m, 1 H), 6.77 – 6.71 (m, 1 H), 4.77 (s, 1 H), 4.56 (s, 1 H), 4.44 (s, 2 H), 3.89 – 3.84 (m, 1 H), 2.74 – 2.66 (m, 1H), 2.42 (dd, *J* = 16.6, 6.3 Hz, 1H), 1.90 (d, *J* = 16.6 Hz, 1H), 1.84 – 1.76 (m, 1H), 1.38 – 1.32 (m, 1H); **^{13}C NMR** (151 MHz, Chloroform-*d*) δ 160.2, 160.0, 138.8, 132.5, 128.8, 128.75, 128.67, 128.6, 128.5, 128.0, 127.5, 123.9, 123.4, 103.7, 59.0, 39.9, 39.3, 30.2, 21.2, 18.1; **HRMS** (ESI) *m/z* calcd for $C_{22}H_{22}N$ $[M + H]^+$, 300.1747; found, 300.1757.

Derivatization of rearranged products.

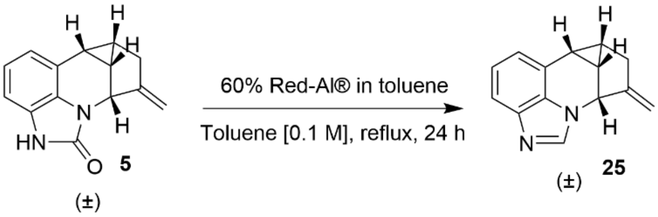

(2a*SR*,2a^1*SR*,2b*RS*,7b*RS*)-1-Methylene-1,2,2a,2a^1,2b,7b-hexahydro-6,7a-diazabenzo[*cd*]cyclopropa[*fg*]-as-indacene (**25**). A toluene solution (1 mL, 0.1 M) of **5** (25 mg, 0.11 mmol, 1 eq) was treated dropwise with a 60% toluene solution of Red-Al (181 uL, 0.56 mmol, 5 eq). The reaction mixture was stirred at reflux for 8 h under nitrogen, diluted three times with Et_2O, cooled to 0 °C, and quenched with 30 % aq. Rochelle salt. The mixture was stirred for 30 min at room temperature. The layers were separated, and the aqueous layer was extracted with ethyl acetate (3x). The combined organic layers were washed with brine, dried (Na_2SO_4), filtered, and concentrated under reduced pressure. The crude residue was purified by chromatography on SiO_2 (30 % hexane in ethyl acetate) to give **25** (6.3 mg, 0.03 mmol, 27%) as a white solid: **Mp** 154.0 - 157.4 °C; **IR** (ATR, CH_2Cl_2) 3358, 3052, 2922, 2850, 1732, 1698, 1732, 1698, 1657, 1632, 1472, 1446, 1372, 1345, 1272, 1198, 904, 748, 730 cm^{-1}; **^{1}H NMR** (601 MHz, Chloroform-*d*) δ 7.92 (s, 1 H), 7.62 (d, *J* = 8.1 Hz, 1 H), 7.28 (d, *J* = 7.2 Hz, 1 H), 7.24 (t, *J* = 7.5 Hz, 1 H), 5.30 (d, *J* = 6.3 Hz, 1 H), 4.95 (t, *J* = 2.2 Hz, 1 H), 4.67 (d, *J* = 2.1 Hz, 1 H), 2.60 – 2.48 (m, 2 H), 2.39 (q, *J* = 7.2 Hz, 1 H), 2.23 (td, *J* = 7.2, 5.0 Hz, 1 H), 1.74 (dq, *J* = 17.3, 2.7 Hz, 1 H); **^{13}C NMR** (151 MHz, Chloroform-*d*) δ 156.6, 141.0, 140.2, 131.7, 122.7, 122.4, 117.8, 108.2, 105.0, 59.1, 29.1, 28.0, 21.5, 20.6. **HRMS** (ESI) *m/z* calcd for $C_{14}H_{13}N_2$ $[M + H]^+$, 209.1073; found, 209.1074.

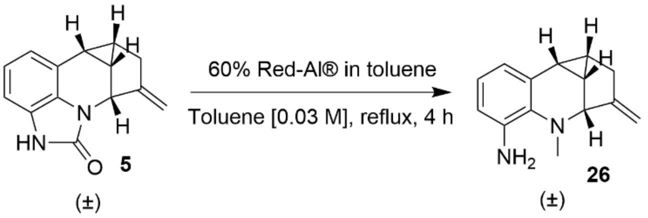

(2a*RS*,2a^1*SR*,7b*RS*,7c*SR*)-3-Methyl-2-methylene-2,2a,2a^1,3,7b,7c-hexahydro-1*H*-3-azabenzo[*f*]cyclopropa[*cd*]inden-4-amine (**26**). A toluene solution (3.8 mL, 0.03 M) of **5** (25 mg, 0.11 mmol, 1 eq) was treated dropwise with a 60% toluene solution of Red-Al® (181 uL, 0.56 mmol, 5 eq). The reaction mixture was stirred at reflux for 4 h under nitrogen, diluted three times with Et_2O, cooled to 0 °C, and quenched with 30 % aq. Rochelle salt. The resulting mixture was stirred for 30 min at room temperature. The layers were separated, and the aqueous layer was extracted with ethyl acetate (3x). The combined organic layers were washed with brine, dried (Na_2SO_4), filtered, and concentrated under reduced pressure. The crude residue was purified by chromatography on SiO_2 (20 % ethyl acetate in hexanes) to give **26** (9.2 mg, 0.04 mmol, 39%) as a colorless oil: **IR** (ATR, CH_2Cl_2) 3334, 3041, 2928, 1590, 1499, 1430, 1272, 1075 cm^{-1}; **^{1}H NMR** (300 MHz, Chloroform-*d*) δ 6.83 (t, *J* = 7.7 Hz, 1 H), 6.69 (dd, *J* = 7.5, 1.5 Hz, 1 H), 6.51 (dd, *J* = 7.8, 1.5 Hz, 1 H), 5.05 – 4.95 (m, 1 H), 4.76 – 4.68 (m, 1 H), 4.23 – 4.14 (m, 1 H), 3.89 (br s, 2 H), 2.70 (s, 3H), 2.56 (ddt, *J* = 17.7, 5.6, 2.7 Hz, 1H), 2.07 (dd, *J* = 17.7, 2.1 Hz, 1H), 2.00 (dd, *J* = 7.4, 3.2 Hz, 2H), 1.61 (q, *J* = 6.9 Hz, 1 H); **^{13}C NMR** (76 MHz, Chloroform-*d*) δ 155.2, 141.7, 135.2, 129.0, 123.9, 120.5, 112.6, 109.2, 66.8, 41.4, 31.1, 23.0, 16.8, 15.4. **HRMS** (ESI) *m/z* calcd for $C_{14}H_{17}N_2$ $[M + H]^+$, 213.1386; found, 213.1384.

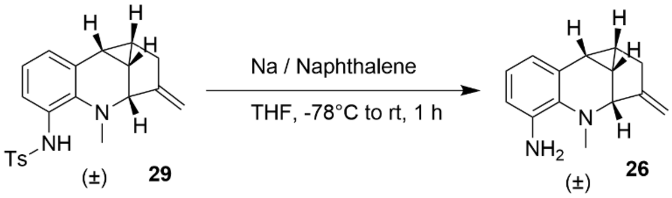

(2a*RS*,2a^1*SR*,7b*RS*,7c*SR*)-3-Methyl-2-methylene-2,2a,2a^1,3,7b,7c-hexahydro-1*H*-3-azabenzo[*f*]cyclopropa[*cd*]inden-4-amine (**26**). A solution of sodium naphthalenide was prepared by dissolving sodium (32 mg) and naphthalene (0.20 g) in dry THF (4.7 mL) at room temperature. A THF solution (1.0 mL, 0.05 M) of **29** (20 mg, 0.05 mmol, 1 eq) was cooled to -78 °C and treated dropwise with the solution of sodium naphthalenide until the mixture remained dark brown/green. The reaction mixture was allowed to warm to room temperature and stirred for an additional 1 h, cooled to -78 °C, quenched with sat. NH_4Cl, warmed up to rt and extracted with ethyl acetate (3x). The combined organic layers were washed with brine, dried ($MgSO_4$), and concentrated in vacuo. The crude residue was purified by chromatography on SiO_2 (5% ethyl acetate in CH_2Cl_2 to 20% ethyl acetate in CH_2Cl_2) to give **26** (9.2 mg, 0.04 mmol, 39%) as a colorless oil.

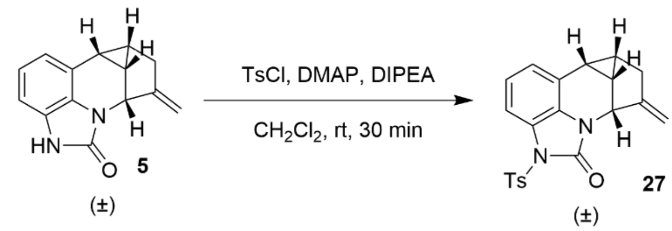

(2a*SR*,2a^1*SR*,2b*RS*,7b*RS*)-1-Methylene-6-tosyl-1,2,2a,2a^1,2b,7b-hexahydro-6,7a-diazabenzo[*cd*]cyclopropa[*fg*]-*as*-indacen-7(6*H*)-one (**27**). A CH_2Cl_2 solution (5 mL, 0.04 mL) of **5** (50 mg, 0.22 mmol, 1 eq) was treated with DMAP (3 mg, 0.02 mmol, 0.1 eq) and DIPEA (78 uL, 0.45 mmol, 2 eq), TsCl (51 mg, 0.27 mmol, 1.2 eq) was added. The reaction mixture was stirred at room temperature for 30 min, treated with sat. $NaHCO_3$ and extracted with CH_2Cl_2 (3x). The combined organic phases were washed with brine, dried ($MgSO_4$), filtered, and concentrated. The crude residue was purified by chromatography on SiO_2 (20% ethyl acetate in hexanes) to give **27** (47.8 mg, 0.13 mmol, 57%) as a white solid: **Mp** 154.2 - 157.2 °C; **IR** (ATR, CH_2Cl_2) 3392, 3056, 2920, 2850, 1734, 1472, 1389, 1372, 1340, 1316, 1265, 1189, 1180, 1170, 1091, 1065, 1022 cm^{-1}; **^{1}H NMR** (601 MHz, Chloroform-*d*) δ 7.96 (d, *J* = 8.4 Hz, 1 H), 7.63 (d, *J* = 0.9 Hz, 1 H), 7.32 – 7.27 (m, 1 H), 7.21 – 7.14 (m, 1 H), 7.09 (t, *J* = 7.9 Hz, 1 H), 5.15 – 5.13 (m, 1 H), 5.11 (dt, *J* = 6.3, 1.1 Hz, 1 H), 4.65 (dt, *J* = 2.5, 1.3 Hz, 1 H), 2.54 – 2.47 (m, 1 H), 2.40 (s, 3 H), 2.30 (t, *J* = 8.2 Hz, 1 H), 2.16 – 2.08 (m, 2 H), 1.79 – 1.73 (m, 1 H); **^{13}C NMR** (151 MHz, Chloroform-*d*) δ 154.6, 150.1, 145.6, 135.4, 129.8, 128.1, 126.0, 124.8, 124.4, 122.5, 117.1, 111.2, 110.2, 55.3, 29.3, 28.0, 21.8, 20.3, 18.4; **HRMS** (ESI) *m/z* calcd for $C_{21}H_{19}N_2O_3S$ [M + H]$^+$, 379.1111; found, 379.1110.

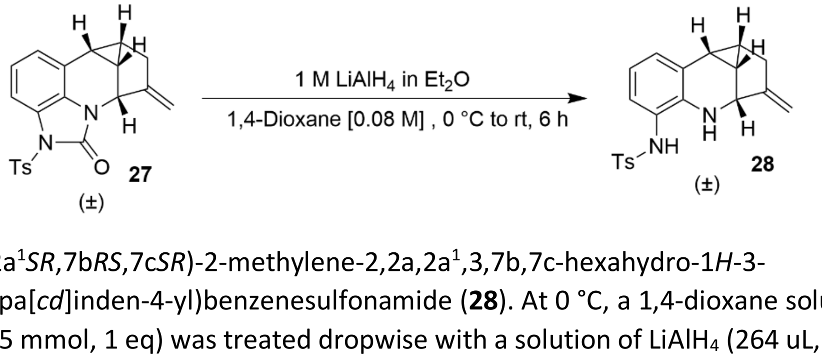

4-Methyl-N-((2a*RS*,2a^1*SR*,7b*RS*,7c*SR*)-2-methylene-2,2a,2a^1,3,7b,7c-hexahydro-1*H*-3-azabenzo[*f*]cyclopropa[*cd*]inden-4-yl)benzenesulfonamide (**28**). At 0 °C, a 1,4-dioxane solution (0.6 mL, 0.08 M) of **27** (20 mg, 0.05 mmol, 1 eq) was treated dropwise with a solution of $LiAlH_4$ (264 uL, 0.26 mmol, 1 M in

Et_2O, 5 eq), stirred for 1 h, warmed up to room temperature and stirred for an additional 5 h. The reaction was quenched with water, and the mixture was partitioned between ethyl acetate and brine. The water layer was extracted with ethyl acetate (2x), and the combined organic layers were dried ($MgSO_4$), filtered, and concentrated. The crude residue was purified by chromatography on SiO_2 (20% ethyl acetate in hexanes) to give **28** (10 mg, 0.03 mmol, 54%) as a white solid: **Mp** 124.0 - 126.8 °C; **IR** (ATR, CH_2Cl_2) 3385, 3254, 1599, 1480, 1330, 1312, 1305, 1157, 1091 cm^{-1}; **^{1}H NMR** (601 MHz, Chloroform-*d*) δ 7.62 (d, *J* = 7.9 Hz, 2 H), 7.24 (d, *J* = 8.0 Hz, 2 H), 7.10 (d, *J* = 7.4 Hz, 1 H), 6.43 (t, *J* = 7.6 Hz, 1 H), 6.22 (d, *J* = 7.8 Hz, 1 H), 5.96 (br s, 1 H), 4.94 (s, 1 H), 4.75 (s, 1 H), 4.41 (d, *J* = 6.5 Hz, 1 H), 2.58 (dd, *J* = 17.6, 6.2 Hz, 1 H), 2.41 (s, 3 H), 2.10 (q, *J* = 7.0 Hz, 1 H), 2.01 (t, *J* = 8.2 Hz, 1 H), 1.92 (d, *J* = 17.6 Hz, 1 H), 1.71 (q, *J* = 7.0 Hz, 1 H); **^{13}C NMR** (151 MHz, Chloroform-*d*) δ 157.3, 143.9, 142.7, 136.0, 130.8, 129.7, 127.9, 125.8, 122.9, 121.3, 117.6, 109.1, 58.0, 31.1, 24.6, 21.7, 18.6, 18.1; **HRMS** (ESI) *m/z* calcd for $C_{20}H_{21}N_2O_2S$ $[M + H]^+$, 353.1318; found, 353.1321.

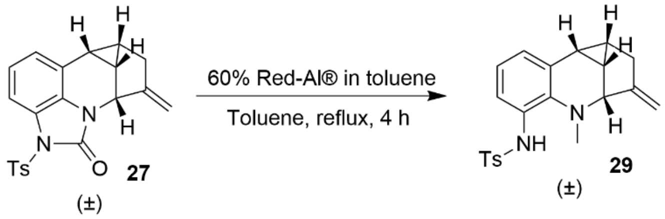

4-Methyl-N-((2a*RS*,2a^1*SR*,7b*RS*,7c*SR*)-3-methyl-2-methylene-2,2a,2a^1,3,7b,7c-hexahydro-1*H*-3-azabenzo[*f*]cyclopropa[*cd*]inden-4-yl)benzenesulfonamide (**29**). A toluene solution (1.8 mL, 0.03 M) of **27** (20 mg, 0.05 mmol, 1 eq) was treated dropwise with a 60% toluene solution of Red-Al (86 uL, 0.26 mmol, 5 eq), stirred at reflux for 4 h under nitrogen, diluted three times with Et_2O, cooled down at 0 °C, and quenched with 30 % Rochelle salt. The resulting mixture was stirred for 30 min at room temperature. Then, the layers were separated and the aqueous layer was extracted with ethyl acetate (3x). The combined organic layers were washed with brine, dried (Na_2SO_4), filtered, and concentrated under reduced pressure. The crude residue was purified by chromatography on SiO_2 (15% ethyl acetate in hexanes) to give **29** (11.1 mg, 0.03 mmol, 57%) as a white solid: **Mp** 144.0 - 145.9 °C; **IR** (ATR, CH_2Cl_2) 3234, 3040, 2905, 1598, 1583, 1473, 1369, 1339, 1314, 1294, 1164, 1091 cm^{-1}; **^{1}H NMR** (500 MHz, Chloroform-*d*) δ 7.77 (br s, 1 H), 7.72 (d, *J* = 8.1 Hz, 2 H), 7.29 (dd, *J* = 6.3, 3.2 Hz, 1 H), 7.20 (d, *J* = 8.1 Hz, 2 H), 6.97 – 6.86 (m, 2 H), 4.92 – 4.81 (m, 1 H), 4.56 (d, *J* = 2.7 Hz, 1 H), 4.15 (d, *J* = 5.2 Hz, 1 H), 2.51 – 2.44 (m, 4 H), 2.36 (s, 3 H), 2.03 – 1.95 (m, 2 H), 1.87 (dq, *J* = 17.9, 2.1 Hz, 1 H), 1.63 (q, *J* = 6.8 Hz, 1 H); **^{13}C NMR** (151 MHz, Chloroform-*d*) δ 154.0, 143.7, 137.4, 136.9, 132.6, 129.7, 127.3, 125.5, 124.6, 114.4, 110.0, 66.8, 42.9, 31.0, 23.9, 21.6, 16.8, 16.0; **HRMS** (ESI) *m/z* calcd for $C_{21}H_{23}N_2O_2S$ $[M + H]^+$, 367.1475; found, 367.1475.

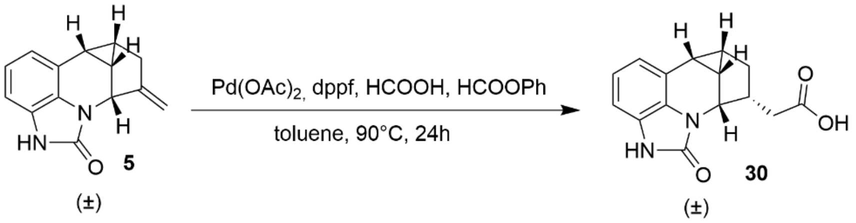

2-((1*SR*,2a*SR*,2a^1*S*,2b*RS*,7b*SR*)-7-Oxo-1,2,2a,2a^1,2b,6,7,7b-octahydro-6,7a-diazabenzo[*cd*]cyclopropa[*fg*]-as-indacen-1-yl)acetic acid (**30**). A stirred mixture of compound **5** (25 mg g, 0.11 mmol, 1 eq), $Pd(OAc)_2$ (1.3 mg, 5.6 µmol, 5 mol%), and 1,1'-bis(diphenylphosphino)ferrocene (6.2 mg, 0.01 mmol, 10 mol%) in toluene (0.22 mL, 0.5 M) in a microwave vial was treated successively via syringe with HCOOH (8.8 uL, 0.22 mmol, 2 eq) and HCOOPh (14.8 uL, 0.13 mmol, 1.2 eq). The vial was purged with argon and tightly sealed with a silicon/PTFE crimp cap. The reaction mixture was stirred at 90 °C for 24 h, cooled to room temperature,

diluted with CH_2Cl_2, and poured into sat. aq. $NaHCO_3$ in a separatory funnel. After vigorous shaking, the mixture was washed with CH_2Cl_2 (3x). The aqueous layer was acidified with 2N HCl, extracted with CH_2Cl_2 (3x), dried (Na_2SO_4), filtered, and concentrated to give carboxylic acid **30** (13.8 mg, 0.05 mmol, 46%) as a white solid: **Mp** 185.1 – 188.4 °C; **IR** (ATR, neat) 3220, 3048, 2923, 2855, 1671, 1647, 1481, 1410, 1343, 1264, 1163, 753, 738 cm^{-1}; **^{1}H NMR** (601 MHz, DMSO-d_6) δ 12.00 (br s, 1 H), 10.74 (s, 1 H), 7.00 (d, *J* = 7.6 Hz, 1 H), 6.92 (t, *J* = 7.7 Hz, 1 H), 6.82 (d, *J* = 7.7 Hz, 1 H), 4.74 (t, *J* = 6.0 Hz, 1 H), 2.83 – 2.74 (m, 1 H), 2.42 – 2.35 (m, 2 H), 2.17 (q, *J* = 6.8 Hz, 1 H), 2.08 – 2.01 (m, 2 H), 1.49 – 1.38 (m, 1 H), 0.41 (td, *J* = 10.9, 5.8 Hz, 1 H); **^{13}C NMR** (126 MHz, DMSO-d_6) δ 172.8, 154.0, 126.8, 125.7, 120.6, 120.0, 116.0, 106.4, 52.1, 51.1, 33.9, 28.2, 27.2, 21.6, 19.5; **HRMS** (ESI) *m/z* calcd for $C_{15}H_{15}N_2O_3$ $[M + H]^+$, 271.1077; found, 271.1076. For the solved X-ray structure of this compound, please see CCDC deposition 2341591.

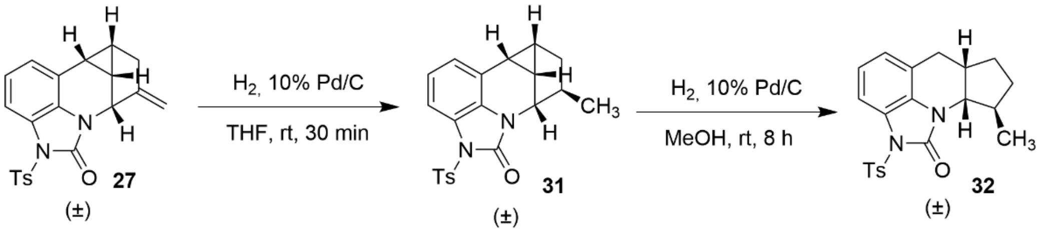

(1*RS*,2a*SR*,2a^1*SR*,2b*RS*,7b*SR*)-1-Methyl-6-tosyl-1,2,2a,2a^1,2b,7b-hexahydro-6,7a-diazabenzo[*cd*]cyclopropa[*fg*]-*as*-indacen-7(6*H*)-one (**31**). A THF (2 mL, 0.02 M) solution of **27** (20 mg, 0.05 mmol, 1 eq) was treated with Pd/C (5.7 mg, 0.005 mmol, 10 mol%). The reaction mixture was purged three times with H_2 and then stirred at room temperature for 30 min under a hydrogen balloon. The mixture was filtered through a pad of celite (30% ethyl acetate in hexanes to rinse) and concentrated *in vacuo*. The crude residue was purified by chromatography on SiO_2 (20% ethyl acetate in hexanes) to give **31** (16.3 mg, 0.047 mmol, 81%) as a colorless solid: **Mp** 215.1 – 218.4 °C; **IR** (ATR, CH_2Cl_2) 3047, 2958, 2928, 2862, 1738, 1476, 1371, 1265, 1169, 1067 cm^{-1}; **^{1}H NMR** (500 MHz, Chloroform-*d*) δ 7.89 (d, *J* = 8.3 Hz, 2 H), 7.60 (d, *J* = 7.9 Hz, 1 H), 7.20 (d, *J* = 8.3 Hz, 2 H), 7.08 (d, *J* = 7.7 Hz, 1 H), 7.01 (t, *J* = 7.9 Hz, 1 H), 4.59 (td, *J* = 6.3, 1.3 Hz, 1 H), 2.49 (ddt, *J* = 11.3, 8.1, 6.4 Hz, 1 H), 2.31 (s, 3 H), 2.21 (t, *J* = 8.1 Hz, 1 H), 2.03 – 1.96 (m, 1 H), 1.96 – 1.89 (m, 1 H), 1.85 (dtd, *J* = 13.7, 7.7, 6.9, 1.3 Hz, 1 H), 0.47 – 0.39 (m, 1 H), 0.38 (d, *J* = 6.8 Hz, 3 H); **^{13}C NMR** (151 MHz, Chloroform-*d*) δ 150.5, 145.6, 135.2, 129.8, 128.0, 126.4, 124.2, 124.1, 122.4, 117.9, 111.0, 54.7, 50.2, 31.0, 28.2, 21.9, 21.8, 20.1, 13.6; **HRMS** (ESI) *m/z* calcd for $C_{21}H_{21}N_2O_3S$ $[M + H]^+$, 381.1273; found, 381.1263.

(6a*SR*,9*RS*,9a*SR*)-9-Methyl-2-tosyl-6,6a,7,8,9,9a-hexahydrocyclopenta[*b*]imidazo[4,5,1-*ij*]quinolin-1(2*H*)-one (**32**). A MeOH (1.5 mL, 0.03 M) solution of **31** (15 mg, 0.04 mmol, 1 eq) was treated with Pd/C (4.2 mg, 0.004 mmol, 10 mol%), purged with H_2 three times and stirred for 8 h at room temperature under a H_2 balloon. The reaction mixture was filtered through a pad of celite (30% ethyl acetate in hexanes to rinse) and concentrated *in vacuo*. The crude residue was purified by chromatography on SiO_2 (20% ethyl acetate in hexanes) to give **32** (13.3 mg, 0.03 mmol, 88%) as a colorless oil: **IR** (ATR, CH_2Cl_2) 3057, 2972, 2968, 1726, 1449, 1351, 1264, 1179cm^{-1}; **^{1}H NMR** (500 MHz, Chloroform-*d*) δ 7.98 (d, *J* = 8.1 Hz, 2 H), 7.86 (d, *J* = 8.0 Hz, 1 H), 7.28 (d, *J* = 8.4 Hz, 2 H), 7.07 (t, *J* = 7.8 Hz, 1 H), 6.98 (d, *J* = 7.6 Hz, 1 H), 4.72 (t, *J* = 5.0 Hz, 1 H), 3.02 (d, *J* = 3.8 Hz, 2 H), 2.73 (dh, *J* = 8.1, 3.9 Hz, 1 H), 2.39 (s, 3 H), 2.29 – 2.12 (m, 3 H), 1.72 (d, *J* = 13.8 Hz, 1 H), 0.83 – 0.74 (m, 1 H), 0.50 (d, *J* = 6.0 Hz, 3 H); **^{13}C NMR** (151 MHz, Chloroform-*d*) δ 151.2, 145.6, 135.2, 129.7, 128.6, 128.1, 127.4, 126.5, 122.4, 122.3, 110.4, 59.5, 41.6, 40.1, 38.4, 35.8, 34.0, 21.8, 14.5; **HRMS** (ESI) *m/z* calcd for $C_{21}H_{23}N_2O_3S$ $[M + H]^+$, 383.1429; found, 383.1420.

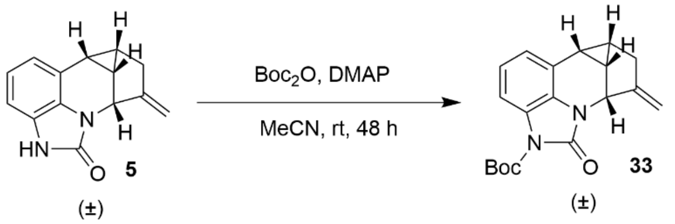

***tert*-Butyl (2a*SR*,2a[1]*SR*,2b*RS*,7b*RS*)-1-methylene-7-oxo-1,2,2a,2a[1],2b,7b-hexahydro-6,7a-diazabenzo[*cd*]cyclopropa[*fg*]-*as*-indacene-6(7*H*)-carboxylate** (**33**). A MeCN solution (1 mL, 0.1 M) of **5** (25 mg, 0.11 mmol, 1 eq) was treated with Boc_2O (49 mg, 0.22 mmol, 2 eq) and DMAP (27 mg, 0.22 mmol, 2 eq), stirred for 48 h at room temperature under nitrogen, and concentrated *in vacuo*. The crude residue was purified by chromatography on SiO_2 (1% MeOH in CH_2Cl_2) to give **33** (14.9 mg, 0.05 mmol, 41%) as a colorless oil: **IR** (ATR, CH_2Cl_2) 2982, 1786, 1747, 1478, 1354, 1311, 1300, 1154, 1137 cm^{-1}; **^{1}H NMR** (400 MHz, Chloroform-*d*) δ 7.47 (dd, *J* = 7.9, 0.9 Hz, 1 H), 7.18 (d, *J* = 6.9 Hz, 1 H), 7.04 (t, *J* = 7.9 Hz, 1 H), 5.30 (p, *J* = 1.2 Hz, 1 H), 5.23 (dt, *J* = 6.4, 1.1 Hz, 1 H), 4.73 (dt, *J* = 2.5, 1.3 Hz, 1 H), 2.53 (ddq, *J* = 17.3, 6.8, 1.5 Hz, 1 H), 2.35 (t, *J* = 8.1 Hz, 1 H), 2.20 (td, *J* = 7.6, 6.4 Hz, 1 H), 2.13 (dtd, *J* = 9.1, 7.1, 2.0 Hz, 1 H), 1.81 – 1.73 (m, 1 H), 1.66 (s, 9 H); **^{13}C NMR** (126 MHz, Chloroform-*d*) δ 154.8, 150.6, 149.1, 126.0, 124.8, 124.0, 122.0, 116.4, 112.4, 110.1, 84.5, 55.0, 29.4, 28.3, 27.9, 20.4, 18.5; **HRMS** (ESI) *m/z* calcd for $C_{19}H_{21}N_2O_3$ [M + H]$^+$, 325.1547; found, 325.1543.

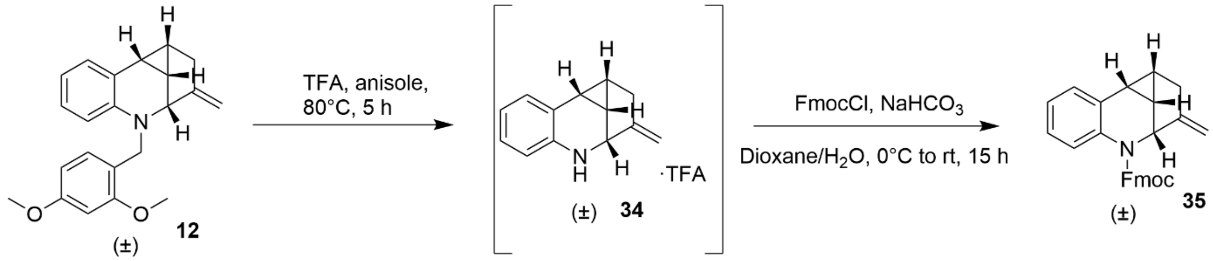

(9*H*-Fluoren-9-yl)methyl (2a*RS*,2a[1]*SR*,7b*RS*,7c*SR*)-2-methylene-1,2,2a,2a[1],7b,7c-hexahydro-3*H*-3-azabenzo[*f*]cyclopropa[*cd*]indene-3-carboxylate (**35**). A mixture of trifluoroacetic acid (1 mL, 0.12 M), **12** (40 mg, 0.12 mmol, 1 eq) and anisole (65 uL, 0.6 mmol, 5 eq) was heated at 80 °C for 5 h under nitrogen. The solvent was evaporated under high vacuum and the crude residue was used in the next step without further purification.

A 1,4-dioxane / water (2:1, 2 mL, 1.3 mL dioxane / 0.7 mL H_2O) solution of crude **35** was treated with $NaHCO_3$ (20.2 mg, 0.24 mmol, 2 eq), stirred for 5 min at room temperature and then cooled to 0 °C. At this point, a 1,4-dioxane solution (0.5 mL) of Fmoc-Cl (57.7 mg, 0.18 mmol, 1.5 eq) was added dropwise. The reaction mixture was warmed up to room temperature and stirred for 15 h under nitrogen. The mixture was partitioned between brine and ethyl acetate, the organic layer was washed with brine, dried ($MgSO_4$), filtered, and concentrated *in vacuo*. The residue was purified by chromatography on SiO_2 (5% ethyl acetate in hexanes) to give **35** (36.6 mg, 0.09 mmol, 75%) as a colorless oil: **IR** (ATR, CH_2Cl_2) 3027, 2898, 1694, 1094, 1493, 1450, 1396, 1314, 1292, 1264, 1247 cm^{-1}; **^{1}H NMR** (601 MHz, Chloroform-*d*) δ 7.81 – 7.73 (m, 2 H), 7.66 – 7.46 (m, 2 H), 7.40 (t, *J* = 7.5 Hz, 2 H), 7.34 – 7.26 (m, 3 H), 7.05 – 7.00 (m, 2 H), 7.00 (br s, 1 H), 5.71 (br s, 1 H), 4.89 – 4.81 (m, 1 H), 4.79 (d, *J* = 2.2 Hz, 1 H), 4.71 – 4.63 (m, 1 H), 4.58 (dd, *J* = 10.8, 6.6 Hz, 1 H), 4.30 (br s, 1 H), 2.60 (ddt, *J* = 17.7, 5.6, 2.7 Hz, 1 H), 2.27 – 2.12 (m, 1 H), 2.11 – 2.04 (m, 1 H), 2.02 (t, *J* = 8.1 Hz, 1 H), 1.65 (dt, *J* = 8.4, 6.0 Hz, 1 H); **HRMS** (ESI) *m/z* calcd for $C_{28}H_{24}NO_2$ [M + H]$^+$, 406.1802; found, 406.1800.

Computational Details

Density Functional Theory (DFT) calculations were primarily performed using the Gaussian 16 software package.[6] The PBE0-D3(BJ)[7,8] functional was utilized consistently, chosen for its reliable performance in transition-metal chemistry and its comparatively lower computational demands. Geometry optimizations and vibrational frequency analyses were executed with the def2-SVP basis set.[9] For the calculation of single-point energies, the more extensive def2-TZVPP basis set was employed,[9] along with the Truhlar-Cramer universal solvation model based on density (SMD) for the solvent 1,4-dioxane.[10] All thermodynamic properties were assessed using the GoodVibes software,[11] applying quasi-rigid-rotor-harmonic-oscillator approximations with a frequency cutoff of 100 cm^{-1}. Molecular visualizations were generated using CYLview[7].[12]

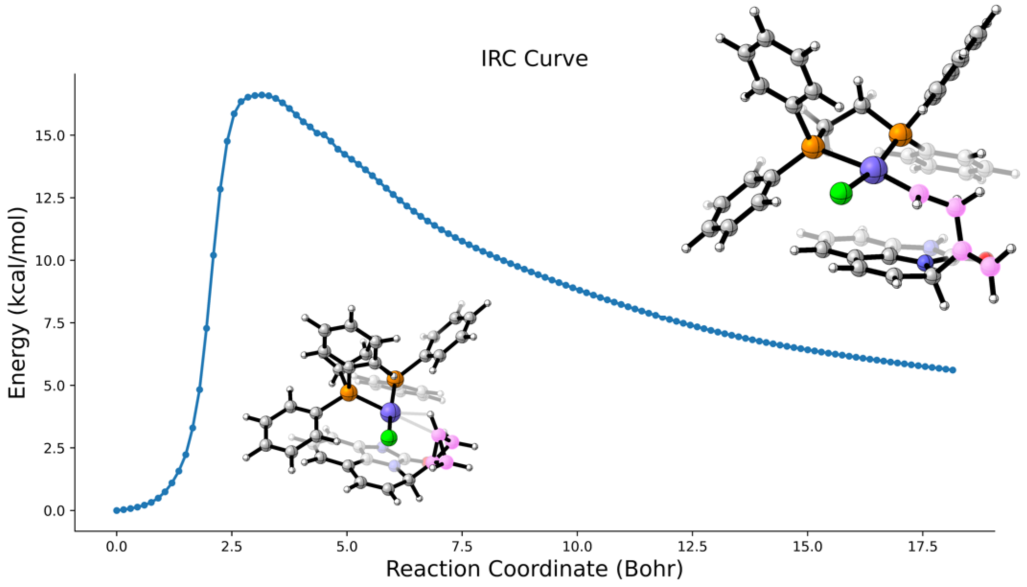

Figure S8. Intrinsic Reaction Coordinate (IRC) Analysis Curve for Double σ-Bond Insertion Reaction

We conducted a distortion/interaction analysis on the transition states associated with Double σ-Bond Insertion Reaction (TS1 and TS1'). The optimized structure of TS1 is dissected into two segments: the Rh(dppe) catalyst fragment and the BCB substrate fragment. The energies of these distorted fragments were computed using the PBE0 level of theory with the def2-TZVPP basis set, incorporating Grimme's D3 (BJ-damping) dispersion corrections. By comparing the energies of the distorted fragments in the transition states with those at the reference point, we derived the distortion energies of the catalyst and substrate ($\Delta E_{dist\text{-}cat}$ and $\Delta E_{dist\text{-}sub}$). The interaction energy, ΔE_{int}, is defined as the disparity between the total distortion energy and the electronic reaction barrier, $\Delta E_{int} = \Delta E^{‡} - (\Delta E_{dist\text{-}cat} + \Delta E_{dist\text{-}sub})$.

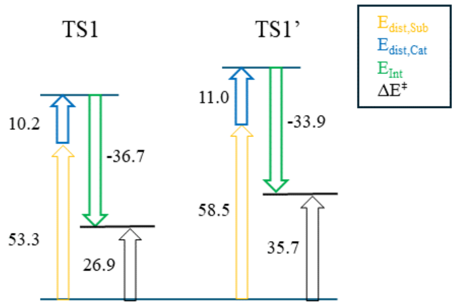

Figure S9. Distortion/interaction Analysis for Transition States **TS1** and **TS1**'

The diastereoselectivity origins were further explored, as depicted in Supplementary Figure S10. To elucidate the specific component of the complex responsible for the observed selectivity, we initiated the removal of chloride and phosphine ligands from rhodium, resulting in the construction of TS2-model-I and TS2'-model-I, respectively. The electronic energy difference (ΔΔE = 2.3 kcal mol^{-1}) between these two models has the same qualitative results as real transition states, indicating that the ligands make a negligible contribution to the selectivity. Subsequently, we eliminated the rhodium to form TS2-model-II and TS2'-model-II. The energy difference for model II (ΔΔE = 24.5 kcal mol^{-1}) aligns with that of real transition states (ΔΔE = 22.3 kcal mol^{-1}), suggesting that the ring strain is the primary factor determining the stereoselectivity.

a) Model-I based on real transition states

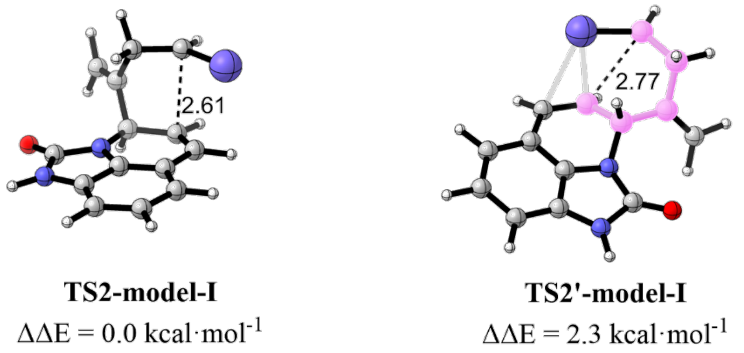

b) Model-II based on real transition states

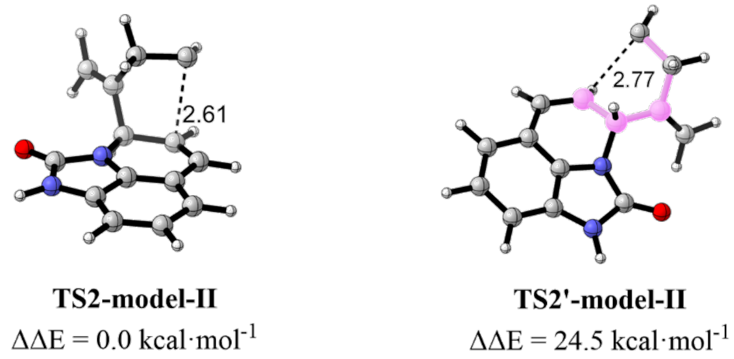

Figure S10. Origins of diastereoselectivity in cycloisomerizations. (a) **Model I**, (b) **Model II**

Supplementary Table 2. Zero-point vibrational energy (ZPVE), thermal correction to enthalpy (TCH), thermal correction to Gibbs free energy (TCG), energies (E), enthalpies (H), and Gibbs free energies (G) (in Hartree) of the structures calculated at the PBE0-D3(BJ)/def2-TZVPP - SMD(1,4-Dioxane) // PBE0-D3(BJ)/def2-SVP level of theory.

Structure	*E_SPC*	E	ZPE	H_SPC	qh-H_SPC	T.S	T.qh-S	G(T)_SPC	qh-G(T)_SPC
**BCB1a**	-2982.6233	-2980.35	0.665189	-2981.92	-2981.92	0.121785	0.110579	-2982.04	-2982.03
**BCB1b**	-2982.6177	-2980.35	0.665685	-2981.91	-2981.92	0.118629	0.109071	-2982.03	-2982.03
**BCB1c**	-2982.6135	-2980.35	0.666116	-2981.9	-2981.91	0.118558	0.109067	-2982.02	-2982.02
**BCB_mo**	-2982.617	-2980.35	0.665307	-2981.91	-2981.92	0.122434	0.110787	-2982.03	-2982.03
**BCB_mph2**	-2982.6096	-2980.34	0.664524	-2981.9	-2981.91	0.125719	0.112695	-2982.03	-2982.02
**BCB_mph**	-2982.6071	-2980.33	0.665056	-2981.9	-2981.91	0.12461	0.112057	-2982.02	-2982.02
**2**	-724.87335	-724.091	0.234084	-724.626	-724.627	0.053263	0.051715	-724.679	-724.679
**37**	-2982.5906	-2980.31	0.663138	-2981.88	-2981.89	0.126261	0.113373	-2982.01	-2982.01
**CO**	-113.22623	-113.096	0.005182	-113.218	-113.218	0.022427	0.022427	-113.24	-113.24
**$[Rh(CO)_2Cl]_2$**	-1594.5448	-1593.63	0.038508	-1594.49	-1594.49	0.061848	0.05883	-1594.55	-1594.55
**dppe**	-1686.9278	-1685.63	0.425681	-1686.48	-1686.48	0.085849	0.078255	-1686.56	-1686.56
**38**	-2982.6034	-2980.33	0.663142	-2981.9	-2981.91	0.121208	0.11086	-2982.02	-2982.02
**5**	-724.91989	-724.14	0.235957	-724.671	-724.672	0.050565	0.049724	-724.722	-724.722
**39’**	-2982.6349	-2980.37	0.66535	-2981.93	-2981.94	0.116961	0.107361	-2982.04	-2982.04
**39**	-2982.6577	-2980.39	0.667084	-2981.95	-2981.96	0.115487	0.106861	-2982.06	-2982.06
**37’**	-2982.6025	-2980.33	0.663577	-2981.9	-2981.9	0.122879	0.111904	-2982.02	-2982.02
**TS1’**	-2982.5635	-2980.29	0.663382	-2981.86	-2981.87	0.117577	0.108419	-2981.98	-2981.97
**TS1**	-2982.5777	-2980.31	0.66285	-2981.87	-2981.88	0.116092	0.107627	-2981.99	-2981.99
**TS2**	-2982.6004	-2980.33	0.662914	-2981.89	-2981.9	0.118624	0.108943	-2982.01	-2982.01
**TS2’**	-2982.5648	-2980.29	0.663159	-2981.86	-2981.87	0.117953	0.10851	-2981.98	-2981.98
**TS3**	-2982.6347	-2980.37	0.665924	-2981.93	-2981.93	0.115439	0.106828	-2982.04	-2982.04

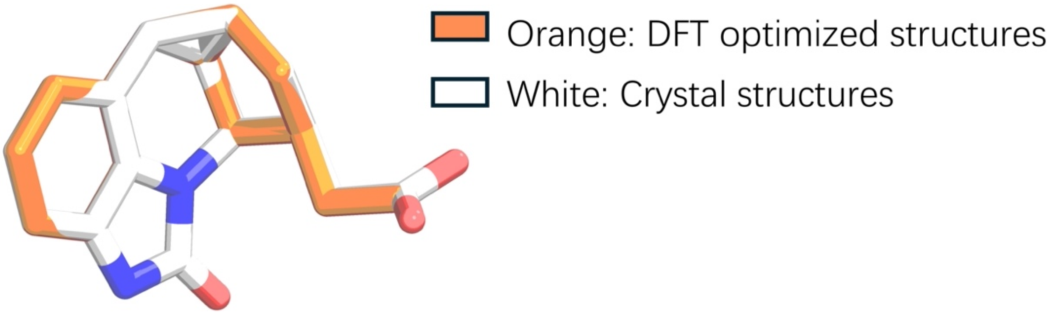

30
RMSD = 0.041
ΔE = 2.3 kcal/mol

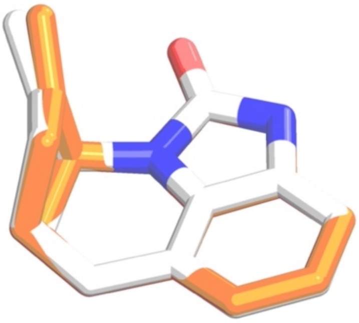

5
RMSD = 0.067
ΔE = 1.0 kcal/mol

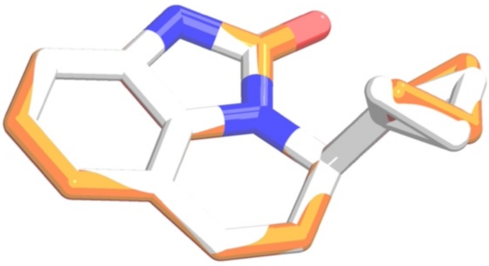

2
RMSD = 0.021
ΔE = 1.1 kcal/mol

Figure S11. Comparative analysis of experimental crystal structures and DFT-optimized structures with RMSD and energy discrepancies.

To validate the appropriateness of the computational methods, we compared the crystal structures obtained experimentally with those optimized using Density Functional Theory (DFT). Initially, we froze the coordinates of the heavy atoms in the crystal structure and optimized the hydrogen atoms. Subsequently, single-point energy calculations were conducted at the PBE0-D3(BJ)/def2-TZVPP level. The results were then compared with those from the DFT optimization.

For structural comparison, we used the 'align' function in PyMOL to align the structures and compute the Root Mean Square Deviation (RMSD). The alignment showed a high degree of congruence between the DFT-optimized structures and the experimental crystal structures, with the highest RMSD being only 0.067. Additionally, the difference in electronic energy was within a reasonable range, at approximately 2.3 kcal/mol. These findings confirm that our choice of computational methods is appropriate.

Supplementary Table 3. Key Bond Lengths, Bond Angles, and Dihedral Angles for Species **2**, **5**, and **30** in DFT-Optimized Structures and Experimental Crystal Structures

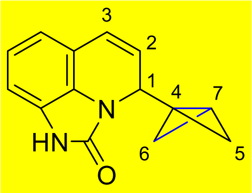

2

Atom	Atom	Atom	Atom		DFT	Crystal
3	2			Distance:	1.346577	1.332283
2	1			Distance:	1.514754	1.518505
1	4			Distance:	1.500606	1.496034
4	7			Distance:	1.473578	1.494345
4	5			Distance:	1.492908	1.4982
4	6			Distance:	1.491045	1.485531
7	5			Distance:	1.49506	1.484932
7	6			Distance:	1.4938	1.482605
3	2	1		Angle:	125.2239	125.6102
2	1	4		Angle:	111.3066	109.7883
1	4	7		Angle:	132.636	130.3563
1	4	5		Angle:	131.0156	128.7499
1	4	6		Angle:	130.0485	132.2003
4	7	5		Angle:	60.37804	60.37959
4	7	6		Angle:	60.32373	59.86765
4	5	7		Angle:	59.09833	60.12137
4	6	7		Angle:	59.16627	60.4586
7	4	5		Angle:	60.52363	59.49904
7	4	6		Angle:	60.51	59.67375
5	4	6		Angle:	98.35746	97.58368
5	7	6		Angle:	98.13994	98.30123
3	2	1	4	Dihedral:	110.2158	106.999
2	1	4	7	Dihedral:	56.91618	44.37911
2	1	4	5	Dihedral:	-28.1696	-35.7144
2	1	4	6	Dihedral:	141.1635	127.2607
1	4	7	5	Dihedral:	-120.284	-116.92
1	4	7	6	Dihedral:	118.9538	121.6113
1	4	5	7	Dihedral:	122.6534	119.398
1	4	6	7	Dihedral:	-122.763	-118.833
4	5	7	6	Dihedral:	48.95464	48.20005
4	6	7	5	Dihedral:	-48.9902	-48.5309
7	5	4	6	Dihedral:	-49.1127	-47.9627
7	6	4	5	Dihedral:	49.12165	47.84918
5	4	7	6	Dihedral:	-120.762	-121.469
5	7	4	6	Dihedral:	120.7619	121.469

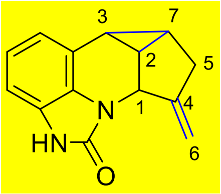

5

Atom	Atom	Atom	Atom		DFT	Crystal
3	2			Distance:	1.514125	1.513225
3	7			Distance:	1.513591	1.517438
2	1			Distance:	1.533665	1.533946
2	7			Distance:	1.507502	1.512775
1	4			Distance:	1.524172	1.517735
7	5			Distance:	1.521177	1.520283
5	4			Distance:	1.511719	1.511244
4	6			Distance:	1.332437	1.311585
3	2	1		Angle:	117.0295	118.4156
3	2	7		Angle:	60.12117	60.19415
3	7	2		Angle:	60.15639	59.91763
3	7	5		Angle:	119.784	121.1147
2	3	7		Angle:	59.72244	59.88822
2	1	4		Angle:	104.3011	103.8005
2	7	5		Angle:	108.8618	108.3957
1	2	7		Angle:	107.4811	106.9034
1	4	5		Angle:	108.3104	107.2902
1	4	6		Angle:	124.0595	124.5637
7	5	4		Angle:	104.7038	104.0957
5	4	6		Angle:	127.6133	127.8665
3	2	1	4	Dihedral:	-81.6104	-84.7709
3	2	7	5	Dihedral:	114.1529	115.8937
3	7	2	1	Dihedral:	-111.626	-113.522
3	7	5	4	Dihedral:	78.71355	81.72387
2	3	7	5	Dihedral:	-95.8016	-94.3785
2	1	4	5	Dihedral:	25.39073	30.82107
2	1	4	6	Dihedral:	-153.212	-143.51
2	7	5	4	Dihedral:	12.94247	16.32298
1	2	3	7	Dihedral:	95.49784	94.07445
1	2	7	5	Dihedral:	2.526667	2.372108
1	4	5	7	Dihedral:	-23.8817	-29.3706
7	2	1	4	Dihedral:	-16.8029	-20.0009
7	5	4	6	Dihedral:	154.6566	144.715

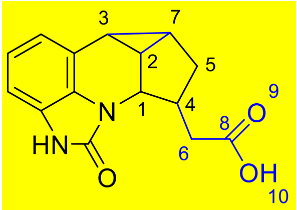

30

Atom	Atom	Atom	Atom		DFT	Crystal
9	8			Distance:	1.202216	1.20375
10	8			Distance:	1.344352	1.306098
1	2			Distance:	1.535252	1.535145
1	4			Distance:	1.548964	1.540628
2	3			Distance:	1.514845	1.518507
2	7			Distance:	1.508026	1.506583
3	7			Distance:	1.512515	1.511892
4	5			Distance:	1.536731	1.534439
4	6			Distance:	1.52053	1.528369
5	7			Distance:	1.518271	1.514655
6	8			Distance:	1.503318	1.49816
9	8	10		Angle:	122.076	122.5545
9	8	6		Angle:	125.4139	124.1242
1	2	3		Angle:	117.4368	117.976
1	2	7		Angle:	107.4017	107.102
1	4	5		Angle:	104.658	104.3118
1	4	6		Angle:	115.0305	114.9091
2	1	4		Angle:	103.5781	103.3671
2	3	7		Angle:	59.75298	59.62365
2	7	3		Angle:	60.20027	60.40646
2	7	5		Angle:	108.2212	108.0622
3	2	7		Angle:	60.04675	59.96989
3	7	5		Angle:	119.3059	120.0787
4	5	7		Angle:	104.474	104.2505
4	6	8		Angle:	114.136	113.6299
5	4	6		Angle:	114.977	115.572
10	8	6		Angle:	112.503	113.3162
9	8	6	4	Dihedral:	136.7864	125.282
10	8	6	4	Dihedral:	-44.1696	-55.521
1	2	3	7	Dihedral:	95.14878	94.44887
1	2	7	3	Dihedral:	-112.129	-112.895
1	2	7	5	Dihedral:	1.780913	2.004816
1	4	5	7	Dihedral:	-32.4387	-34.0292
1	4	6	8	Dihedral:	174.1841	174.1
2	1	4	5	Dihedral:	33.31185	35.01864
2	1	4	6	Dihedral:	160.457	162.5813
2	3	7	5	Dihedral:	-95.2648	-94.7676
2	7	5	4	Dihedral:	19.08842	19.867
3	2	1	4	Dihedral:	-86.4111	-87.4651
3	2	7	5	Dihedral:	113.9103	114.9002
3	7	5	4	Dihedral:	84.55566	85.57462
4	1	2	7	Dihedral:	-21.6783	-22.8984

5	4	6	8	Dihedral:	-64.1067	-64.2746
7	5	4	6	Dihedral:	-159.617	-161.183

Supplementary Table 4. Electronic Energy (in a.u.) for Distortion/Interaction Analysis, Model Calculations, and Comparative Studies of DFT-Optimized and Crystal Structures

Energy (Distortion-Interaction Analysis)		Energy (Model calculation)		Energy (Crystal and DFT structures)	
$E_{sub\text{-}ref}$	-724.857	$E_{ts2\text{-}model\text{-}I}$	-835.221	$E_{2\text{-}crystal}$	-724.855
$E_{cat\text{-}ref}$	-2257.67	$E_{ts2\text{-}model\text{-}II}$	-724.768	$E_{2\text{-}DFT}$	-724.857
$E_{sub\text{-}ts1}$	-724.772	$E_{ts2'\text{-}model\text{-}I}$	-835.217	$E_{30\text{-}crystal}$	-914.598
$E_{cat\text{-}ts1}$	-2257.67	$E_{ts2'\text{-}model\text{-}II}$	-724.729	$E_{30\text{-}DFT}$	-914.602
$E_{sub\text{-}ts1'}$	-724.764			$E_{5\text{-}crystal}$	-724.903
$E_{cat\text{-}ts1'}$	-2257.67			$E_{5\text{-}DFT}$	-724.905
E_{ts1}	-2982.55				
$E_{ts1'}$	-2257.69				

BCB 1a

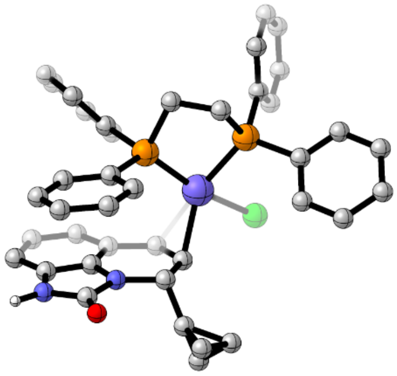

Energy: -1871624.3669123

C	-3.91591	2.34839	-2.19712
C	-4.84423	1.74435	-1.33546
C	-4.48034	0.53266	-0.75612
C	-3.22155	-0.00829	-1.02856
C	-2.29366	0.55902	-1.88877
C	-2.67406	1.77092	-2.48326
N	-3.08648	-1.18380	-0.33488
C	-1.91505	-2.04047	-0.44115
C	-0.91086	-1.43479	-1.40784
C	-1.08492	-0.23799	-2.11266
N	-5.08558	-0.36719	0.10161

C	-4.23672	-1.43295	0.40463
O	-4.45877	-2.35788	1.15196
C	-2.28491	-3.42538	-0.87860
C	-1.45980	-4.61557	-1.15470
C	-2.48224	-4.65750	-0.06606
C	-2.32596	-4.04962	-2.23385
H	-4.17856	3.29754	-2.66908
H	-5.81380	2.20654	-1.14115
H	-1.99561	2.26460	-3.18172
H	-1.45879	-2.12826	0.56105
H	-0.23828	-2.17771	-1.84318
H	-0.52658	-0.09483	-3.04296
H	-5.98982	-0.28035	0.54048
H	-0.37827	-4.74844	-1.12559
H	-3.39929	-5.24919	-0.19783
H	-2.12223	-4.61040	0.96759
H	-1.82149	-3.50707	-3.04119
H	-3.22636	-4.59891	-2.54610
Rh	0.68887	-0.13053	-0.74932
P	-0.18887	1.13348	0.87126
C	1.08334	1.37905	2.23270
C	2.03868	0.20068	2.28106
P	2.54921	-0.18692	0.54910
Cl	1.97277	-1.12739	-2.49750
C	-0.45093	2.86384	0.33863
C	-1.66944	0.69687	1.85570
C	3.89033	0.99369	0.16432
C	3.35729	-1.81263	0.68861
C	4.49061	1.81174	1.12900
C	5.49984	2.70321	0.76681
C	5.92129	2.78033	-0.55882
C	5.32663	1.96713	-1.52397

C	4.31186	1.08211	-1.17100
C	4.72769	-1.94884	0.92600
C	5.28336	-3.21855	1.07667
C	4.47519	-4.35123	0.99306
C	3.10728	-4.21700	0.75240
C	2.54976	-2.95217	0.59309
C	-0.12631	3.20117	-0.97863
C	-0.22015	4.52268	-1.41148
C	-0.63731	5.51679	-0.52945
C	-0.94872	5.19079	0.79150
C	-0.85095	3.87193	1.22671
C	-2.85004	1.44461	1.84687
C	-3.91891	1.06895	2.66146
C	-3.82765	-0.05778	3.47349
C	-2.66218	-0.82390	3.46542
C	-1.59074	-0.44784	2.66324
H	1.62558	2.30204	1.97229
H	0.57233	1.54971	3.19212
H	2.90155	0.37128	2.94323
H	1.53949	-0.71222	2.64115
H	4.17861	1.76371	2.17420
H	5.95850	3.33950	1.52747
H	6.71284	3.47871	-0.84119
H	5.65042	2.02586	-2.56581
H	3.82898	0.45071	-1.92434
H	5.36332	-1.06150	0.98153
H	6.35618	-3.32292	1.25541
H	4.91476	-5.34509	1.10682
H	2.47440	-5.10421	0.67339
H	1.48539	-2.83047	0.37228
H	0.20919	2.41046	-1.65715
H	0.03553	4.77486	-2.44326

H	-0.71417	6.55275	-0.86844
H	-1.26657	5.97041	1.48798
H	-1.08988	3.62692	2.26491
H	-2.94353	2.31661	1.19759
H	-4.83557	1.66395	2.64981
H	-4.67276	-0.35785	4.09648
H	-2.59622	-1.72725	4.07489
H	-0.68362	-1.05883	2.65402

BCB 1b

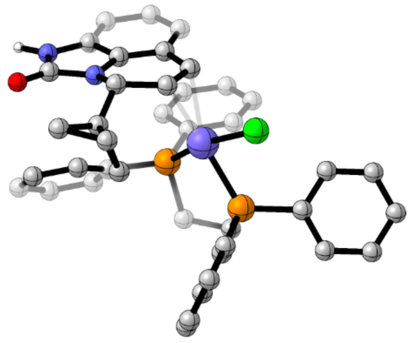

Energy: -1871620.8663257

C	3.82667	2.94336	-1.34404
C	4.73975	2.00941	-0.83312
C	4.35448	0.67155	-0.83533
C	3.09816	0.31983	-1.33256
C	2.16785	1.23092	-1.81020
C	2.56531	2.57382	-1.82137
N	2.97440	-1.05082	-1.31081
C	1.89923	-1.72385	-2.03642
C	0.79201	-0.73984	-2.34706
C	0.91360	0.64559	-2.28648
N	4.96472	-0.52682	-0.51186
C	4.16058	-1.61467	-0.84734

O	4.44707	-2.78654	-0.75364
C	1.38602	-2.97498	-1.39147
C	0.46831	-3.98714	-1.96139
C	1.85688	-4.37602	-1.58311
C	0.08404	-3.27829	-0.71093
H	4.10696	3.99834	-1.35692
H	5.71933	2.32315	-0.46791
H	1.87705	3.33648	-2.19064
H	2.31602	-2.04223	-3.01606
H	0.02865	-1.15484	-3.00906
H	0.23259	1.26613	-2.87528
H	5.89254	-0.66064	-0.13786
H	-0.02985	-3.99535	-2.93036
H	2.02550	-5.00599	-0.69831
H	2.57938	-4.53514	-2.39049
H	-0.69576	-2.50888	-0.79099
H	0.09301	-3.81941	0.24754
Rh	-0.64231	-0.00088	-0.79666
P	0.48206	0.60015	1.07513
C	-0.71117	0.62921	2.52041
C	-2.09529	1.05593	2.08441
P	-2.49786	0.19031	0.50649
Cl	-2.20745	-0.33322	-2.58280
C	1.83150	-0.28414	1.95569
C	0.98827	2.36849	0.99845
C	-3.13074	-1.45511	0.99059
C	-3.96296	1.10528	-0.07422
C	-3.21651	-1.87682	2.32202
C	-3.66756	-3.16157	2.62501
C	-4.04079	-4.03084	1.60289
C	-3.96072	-3.61309	0.27369
C	-3.50411	-2.33602	-0.03590

C	-5.22668	0.85329	0.46940
C	-6.31748	1.63251	0.08827
C	-6.15010	2.66460	-0.83396
C	-4.89184	2.91324	-1.38209
C	-3.80064	2.13283	-1.00952
C	2.19395	-1.56547	1.53771
C	3.14461	-2.30593	2.24123
C	3.72592	-1.77905	3.39036
C	3.33421	-0.51997	3.84883
C	2.38933	0.21937	3.14353
C	2.20400	2.88067	1.45872
C	2.46779	4.24829	1.38740
C	1.52775	5.12156	0.84788
C	0.32500	4.61632	0.35300
C	0.06324	3.25145	0.41883
H	-0.71523	-0.40614	2.89800
H	-0.30493	1.27137	3.31650
H	-2.86673	0.88244	2.85109
H	-2.12333	2.13013	1.84428
H	-2.93145	-1.21040	3.13848
H	-3.72586	-3.48199	3.66794
H	-4.39161	-5.03768	1.84160
H	-4.24778	-4.29179	-0.53285
H	-3.41539	-2.00971	-1.07717
H	-5.36073	0.03749	1.18481
H	-7.30423	1.42862	0.51084
H	-7.00711	3.27203	-1.13501
H	-4.76224	3.70961	-2.11862
H	-2.82037	2.28859	-1.46605
H	1.72443	-1.99465	0.65534
H	3.43586	-3.28904	1.86673
H	4.47465	-2.35390	3.94098

H	3.76452	-0.11107	4.76640
H	2.08111	1.19178	3.53257
H	2.97349	2.21131	1.84277
H	3.42846	4.62825	1.74331
H	1.73806	6.19245	0.79407
H	-0.40999	5.28697	-0.09868
H	-0.85328	2.84842	-0.02099

BCB 1c

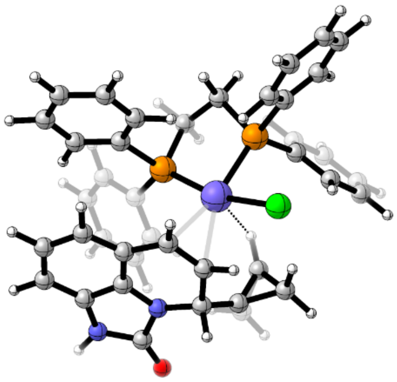

Energy: -1871618.2042109

C	4.24119	2.44536	-1.03375
C	5.08077	1.37951	-0.68303
C	4.57551	0.09229	-0.83626
C	3.26888	-0.08900	-1.29157
C	2.41952	0.95192	-1.63558
C	2.94147	2.24546	-1.51113
N	3.01122	-1.43899	-1.39058
C	1.80474	-1.94448	-2.03838
C	0.84944	-0.80345	-2.34298
C	1.11765	0.54490	-2.17159
N	5.08946	-1.18176	-0.68588
C	4.17137	-2.15203	-1.08327
O	4.35824	-3.34471	-1.14365

C	1.12105	-3.08111	-1.32473
C	0.22472	-3.25823	-0.16953
C	-0.26582	-3.60096	-1.53565
C	1.51637	-4.01177	-0.23066
H	4.61633	3.46635	-0.93572
H	6.09732	1.55685	-0.32763
H	2.32209	3.10303	-1.78116
H	2.11168	-2.34800	-3.02500
H	0.05745	-1.08440	-3.03859
H	0.52528	1.28409	-2.71974
H	6.02935	-1.43374	-0.41778
H	-0.18112	-2.55844	0.56030
H	-0.39915	-4.66062	-1.80431
H	-0.99281	-2.91085	-1.97938
H	2.32931	-3.68769	0.42427
H	1.50541	-5.09717	-0.41501
Rh	-0.61729	0.02390	-0.78681
P	0.41658	0.95389	0.98632
C	-0.74120	0.96803	2.45438
C	-2.17281	1.16111	2.00511
P	-2.47293	0.13112	0.50049
Cl	-2.17154	-0.58178	-2.51922
C	1.89203	0.32749	1.87840
C	0.67340	2.75612	0.69828
C	-2.92394	-1.52906	1.11392
C	-4.03580	0.83762	-0.11941
C	-2.83494	-1.88955	2.46318
C	-3.12553	-3.19403	2.86238
C	-3.51281	-4.14367	1.92010
C	-3.60988	-3.78629	0.57474
C	-3.31190	-2.49005	0.16862
C	-5.25284	0.50990	0.48731

C	-6.42814	1.13709	0.07799
C	-6.39246	2.09338	-0.93584
C	-5.18103	2.41750	-1.54633
C	-4.00542	1.78855	-1.14487
C	2.35227	-0.94923	1.55341
C	3.39810	-1.53599	2.26376
C	3.98583	-0.85269	3.32508
C	3.50399	0.40491	3.68966
C	2.45695	0.98795	2.98051
C	1.79434	3.49179	1.09740
C	1.88305	4.85513	0.81962
C	0.85884	5.50396	0.13334
C	-0.25378	4.77668	-0.28825
C	-0.34024	3.41397	-0.01493
H	-0.59913	-0.01542	2.93049
H	-0.41708	1.73612	3.17334
H	-2.91106	0.94203	2.79261
H	-2.34912	2.19963	1.68349
H	-2.53709	-1.15936	3.21820
H	-3.04743	-3.46654	3.91753
H	-3.73590	-5.16618	2.23368
H	-3.90756	-4.52820	-0.16976
H	-3.35721	-2.21080	-0.88806
H	-5.28359	-0.24766	1.27505
H	-7.37720	0.87326	0.55075
H	-7.31527	2.58126	-1.25905
H	-5.15339	3.15296	-2.35368
H	-3.05986	2.00034	-1.64913
H	1.87322	-1.48343	0.73565
H	3.75000	-2.53225	1.98526
H	4.80780	-1.30692	3.88366
H	3.94023	0.93340	4.54080

H	2.07279	1.95664	3.30541
H	2.63189	2.99740	1.58775
H	2.77197	5.40969	1.13017
H	0.93385	6.57161	-0.08643
H	-1.05463	5.26775	-0.84609
H	-1.18624	2.83355	-0.39320

BCB_m-o

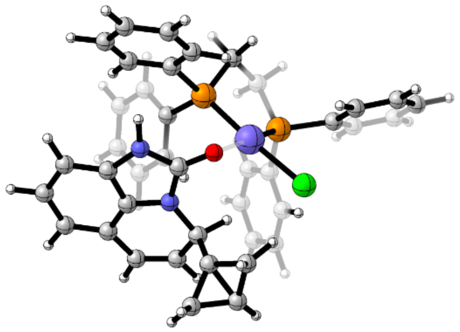

Energy: -1871620.4261152

C	-5.59960	0.55511	1.04934
C	-5.14320	-0.32012	0.05310
C	-3.94573	0.00788	-0.57695
C	-3.28296	1.17644	-0.20759
C	-3.68207	2.03384	0.80647
C	-4.88936	1.70107	1.43503
N	-2.10218	1.25260	-0.89601
C	-1.24938	2.43805	-0.83594
C	-1.57239	3.20573	0.42552
C	-2.71116	3.07360	1.13502
N	-3.12056	-0.59229	-1.51712
C	-1.95279	0.12835	-1.67065
O	-0.97117	-0.20389	-2.33805
C	-1.44558	3.29406	-2.05125

C	-0.80351	4.54429	-2.49035
C	-0.68520	3.32179	-3.33819
C	-2.28120	4.51520	-2.26510
H	-6.53653	0.32097	1.55925
H	-5.69646	-1.22620	-0.19885
H	-5.27028	2.32594	2.24622
H	-0.19796	2.08850	-0.82715
H	-0.83123	3.95502	0.71341
H	-2.89292	3.71277	2.00322
H	-3.21040	-1.52835	-1.89346
H	-0.03134	5.14225	-2.00793
H	-1.25059	3.27392	-4.28070
H	0.27286	2.78977	-3.33233
H	-2.66016	5.01491	-1.36645
H	-2.95812	4.55733	-3.13165
Rh	0.82926	-0.40126	-1.00985
P	-0.07783	-1.93322	0.27437
C	1.30488	-3.02090	0.85825
C	2.33548	-2.07719	1.47170
P	2.46478	-0.53084	0.41716
Cl	1.98394	1.20361	-2.32888
C	-0.79449	-1.39013	1.87621
C	-1.34844	-3.02584	-0.46335
C	2.45835	0.89067	1.58076
C	4.19113	-0.58941	-0.17928
C	2.29329	0.79119	2.96501
C	2.19696	1.94108	3.75052
C	2.26964	3.20005	3.16145
C	2.44096	3.30682	1.77931
C	2.52971	2.16399	0.99143
C	5.25835	-0.30266	0.68004
C	6.57018	-0.42481	0.22852

C	6.82135	-0.83115	-1.08250
C	5.75978	-1.10933	-1.94197
C	4.44522	-0.98590	-1.49535
C	-0.97310	-0.01979	2.08488
C	-1.46766	0.45497	3.29822
C	-1.77720	-0.43780	4.32066
C	-1.58597	-1.80814	4.13051
C	-1.09540	-2.28213	2.91664
C	-2.56680	-3.34336	0.14888
C	-3.53649	-4.06497	-0.54758
C	-3.29896	-4.47606	-1.85937
C	-2.08582	-4.16236	-2.47601
C	-1.11927	-3.43516	-1.78528
H	0.97226	-3.79350	1.56871
H	1.70236	-3.52312	-0.03702
H	2.01429	-1.79579	2.48443
H	3.32968	-2.53903	1.55454
H	2.22316	-0.18182	3.45398
H	2.06060	1.84567	4.83045
H	2.19281	4.09937	3.77759
H	2.50033	4.29130	1.30806
H	2.62538	2.24181	-0.09753
H	5.06187	0.03042	1.70311
H	7.40063	-0.19478	0.90052
H	7.85092	-0.92140	-1.43780
H	5.95422	-1.41131	-2.97379
H	3.60233	-1.16435	-2.16710
H	-0.69179	0.67386	1.28912
H	-1.59739	1.52999	3.43910
H	-2.16169	-0.06838	5.27454
H	-1.81878	-2.51054	4.93467
H	-0.94625	-3.35710	2.78191

H	-2.77864	-2.99544	1.16147
H	-4.48723	-4.30027	-0.06269
H	-4.06233	-5.03558	-2.40546
H	-1.89898	-4.46918	-3.50771
H	-0.19298	-3.13410	-2.28343

BCB_m-ph_2

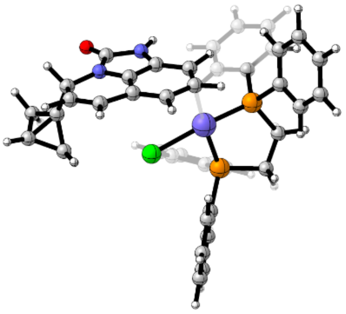

Energy: -1871615.7924974

C	0.01878	-2.00216	-1.99506
C	-0.00152	-2.30561	-0.60674
C	-1.26000	-2.39460	0.00881
C	-2.40105	-2.17965	-0.75095
C	-2.41077	-1.92487	-2.11745
C	-1.16499	-1.83447	-2.73918
N	-3.49880	-2.28309	0.05695
C	-4.83191	-1.91210	-0.39530
C	-4.83738	-1.81187	-1.90586
C	-3.74268	-1.77586	-2.68822
N	-1.70760	-2.63862	1.29122
C	-3.10163	-2.55138	1.36313
O	-3.80079	-2.69282	2.33866
C	-5.28329	-0.62912	0.23652
C	-6.47990	0.21243	0.05038
C	-6.19836	-0.39208	1.38835

C	-5.12208	0.78261	-0.21227
H	0.97736	-2.02379	-2.51722
H	0.88569	-2.68302	-0.09988
H	-1.10067	-1.61688	-3.80699
H	-5.53430	-2.70309	-0.07563
H	-5.82911	-1.70470	-2.35211
H	-3.84674	-1.64617	-3.76840
H	-1.14119	-2.82599	2.10611
H	-7.33715	0.09205	-0.61142
H	-5.86383	0.25077	2.21574
H	-6.81466	-1.24298	1.69971
H	-4.82020	0.92530	-1.25497
H	-4.69518	1.51865	0.48321
Rh	0.45994	0.05935	-0.60972
P	2.49148	-0.51329	0.01010
C	3.55648	0.97808	0.39416
C	2.91300	2.22895	-0.17917
P	1.06980	2.08940	-0.04084
Cl	-1.72000	0.95771	-0.98050
C	2.57430	-1.42332	1.60437
C	3.48399	-1.51421	-1.15831
C	0.68339	2.45301	1.71127
C	0.50445	3.55056	-0.97239
C	1.61446	2.97572	2.61527
C	1.26894	3.15979	3.95466
C	-0.01036	2.83197	4.39717
C	-0.94631	2.31862	3.49729
C	-0.60389	2.12232	2.16287
C	0.41099	4.81038	-0.37371
C	0.04870	5.91900	-1.13730
C	-0.21940	5.77259	-2.49738
C	-0.13241	4.51464	-3.09452

C	0.22178	3.40339	-2.33472
C	1.46107	-1.31260	2.44867
C	1.47564	-1.89533	3.71458
C	2.60155	-2.59686	4.14556
C	3.71693	-2.70203	3.31429
C	3.70798	-2.11298	2.05074
C	3.52135	-2.91345	-1.10458
C	4.16035	-3.64356	-2.10637
C	4.77002	-2.98744	-3.17312
C	4.73324	-1.59397	-3.23871
C	4.08925	-0.86460	-2.24402
H	3.60052	1.03209	1.49260
H	4.58369	0.81853	0.03285
H	3.29117	3.15625	0.27928
H	3.09035	2.31316	-1.26309
H	2.62147	3.24158	2.28580
H	2.00624	3.56101	4.65425
H	-0.27908	2.97428	5.44676
H	-1.95140	2.05933	3.83884
H	-1.32270	1.70191	1.45077
H	0.60867	4.92376	0.69541
H	-0.03046	6.90109	-0.66485
H	-0.50823	6.64179	-3.09346
H	-0.35923	4.39542	-4.15661
H	0.25572	2.40543	-2.77904
H	0.59065	-0.74897	2.09316
H	0.60671	-1.79280	4.36981
H	2.61332	-3.05839	5.13593
H	4.60264	-3.24453	3.65384
H	4.58855	-2.19426	1.40800
H	3.05517	-3.44368	-0.27088
H	4.18093	-4.73445	-2.04926

H	5.27108	-3.56067	-3.95650
H	5.20361	-1.07181	-4.07527
H	4.04283	0.22503	-2.32246

BCB_m-ph_1

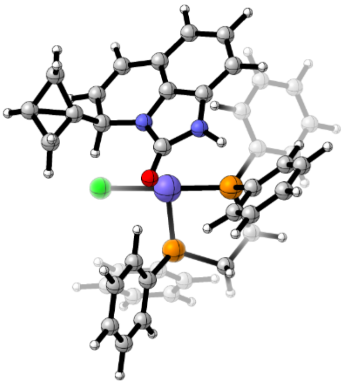

Energy: -1871614.2083812

C	5.13152	-1.08540	-0.76015
C	4.61380	-0.84570	0.52213
C	3.63862	0.14081	0.64248
C	3.22299	0.82629	-0.49911
C	3.69991	0.59761	-1.78047
C	4.69186	-0.38899	-1.89379
N	2.23180	1.70827	-0.15620
C	1.77446	2.73937	-1.08130
C	2.13584	2.32925	-2.49124
C	3.04379	1.38929	-2.81686
N	2.89299	0.67350	1.67644
C	1.99881	1.63722	1.21300
O	1.19707	2.27154	1.86491
C	2.33829	4.08112	-0.72729
C	2.19219	5.40640	-1.35698
C	1.71080	5.19591	0.04030
C	3.55240	4.80421	-1.20946

H	5.90020	-1.85245	-0.87681
H	4.96118	-1.41578	1.38502
H	5.11792	-0.61933	-2.87335
H	0.67498	2.79705	-1.01699
H	1.61240	2.88010	-3.27512
H	3.26503	1.18118	-3.86680
H	2.84603	0.32906	2.62711
H	1.64862	5.70174	-2.25356
H	2.26018	5.64905	0.87850
H	0.62877	5.11945	0.19337
H	4.02153	4.40975	-2.11795
H	4.25509	5.23011	-0.47772
Rh	-0.65951	-0.15287	-0.74233
P	-0.08652	-1.93929	0.40826
C	-1.62552	-2.83914	0.93529
C	-2.62409	-1.78552	1.38898
P	-2.58122	-0.35296	0.20208
Cl	-1.26313	1.67261	-2.07098
C	0.94583	-3.19337	-0.42077
C	0.75838	-1.70284	2.02777
C	-3.98811	-0.63097	-0.92917
C	-3.09404	1.07325	1.21745
C	-4.58560	-1.88763	-1.07817
C	-5.63491	-2.06671	-1.97857
C	-6.09474	-0.99263	-2.73693
C	-5.49606	0.26031	-2.59887
C	-4.44473	0.44260	-1.70658
C	-4.41577	1.20651	1.66194
C	-4.76450	2.26530	2.49652
C	-3.79861	3.19341	2.89007
C	-2.48434	3.06475	2.44460
C	-2.12953	2.01022	1.60492

C	2.00483	-2.73746	-1.21605
C	2.83055	-3.64442	-1.87475
C	2.60233	-5.01449	-1.75313
C	1.54860	-5.47622	-0.96535
C	0.72450	-4.57081	-0.29918
C	1.82495	-2.50461	2.45156
C	2.42870	-2.27400	3.68762
C	1.96456	-1.25186	4.51799
C	0.90092	-0.44964	4.10112
C	0.31160	-0.66590	2.85759
H	-1.98923	-3.37519	0.04348
H	-1.41253	-3.57364	1.72727
H	-3.64699	-2.17592	1.49879
H	-2.33418	-1.37114	2.36665
H	-4.23677	-2.74304	-0.49570
H	-6.09381	-3.05241	-2.08609
H	-6.91772	-1.13266	-3.44188
H	-5.84499	1.10375	-3.19917
H	-3.95193	1.41408	-1.62546
H	-5.17632	0.48808	1.34373
H	-5.79703	2.36996	2.83859
H	-4.07674	4.02414	3.54353
H	-1.71965	3.78571	2.74261
H	-1.09841	1.92082	1.25060
H	2.19363	-1.66483	-1.31877
H	3.65500	-3.27076	-2.48524
H	3.24658	-5.72586	-2.27544
H	1.36589	-6.54907	-0.86771
H	-0.09253	-4.94774	0.31979
H	2.19737	-3.30235	1.80492
H	3.26760	-2.89839	4.00521
H	2.43877	-1.07499	5.48651

H	0.54862	0.37383	4.72578
H	-0.46244	0.01830	2.50504

2

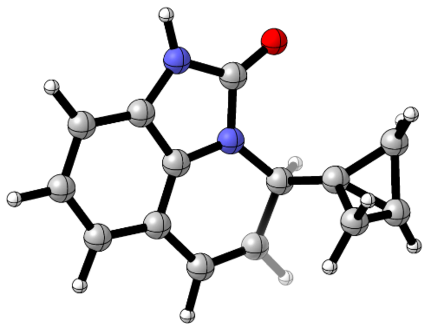

Energy: -454864.8932845

C	-3.65827	-0.66238	0.57384
C	-3.29205	0.69272	0.60542
C	-1.99332	1.01524	0.22272
C	-1.12797	-0.00933	-0.16181
C	-1.46792	-1.35084	-0.22227
C	-2.77731	-1.67059	0.16541
N	0.07798	0.53270	-0.51519
C	1.24371	-0.28541	-0.81497
C	0.80432	-1.71750	-1.03978
C	-0.40856	-2.21508	-0.73219
N	-1.25532	2.17981	0.09223
C	0.03949	1.91510	-0.36027
O	0.92572	2.70765	-0.57427
C	2.27057	-0.19507	0.27570
C	3.58802	-0.82896	0.46006
C	3.48820	0.65925	0.37864
C	2.50962	-1.05872	1.46973
H	-4.67246	-0.93560	0.87268
H	-4.00670	1.45715	0.91565

H	-3.11285	-2.71004	0.14270
H	1.71116	0.10831	-1.73578
H	1.57752	-2.37709	-1.44152
H	-0.61867	-3.27578	-0.89330
H	-1.56356	3.12348	0.27596
H	4.13016	-1.53917	-0.16339
H	3.64458	1.26530	1.28258
H	3.81831	1.13964	-0.54875
H	2.02404	-2.04108	1.46321
H	2.59295	-0.59448	2.46346

CO

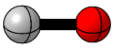

Energy: -71050.5305430

O	0.00000	0.00000	0.48347
C	0.00000	0.00000	-0.64463

37

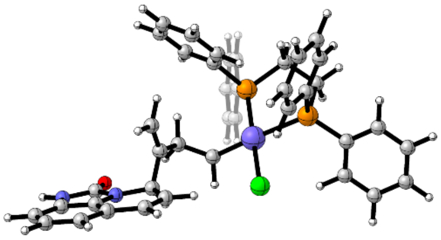

Energy: -1871603.8513499

C	-7.91057	-2.64499	0.56584
C	-8.14704	-1.33419	0.12410
C	-7.05579	-0.61902	-0.36285
C	-5.80328	-1.23068	-0.38667
C	-5.54007	-2.52469	0.03209
C	-6.64210	-3.23861	0.52364
N	-4.89042	-0.35513	-0.91176
C	-3.46994	-0.64858	-0.98497
C	-3.21963	-2.09433	-0.61356
C	-4.14918	-2.94009	-0.12952
N	-6.85008	0.64758	-0.88887
C	-5.51259	0.84177	-1.23398
O	-5.00422	1.83297	-1.71025
C	-0.69148	0.50295	-1.34992
C	-1.79364	1.32289	-0.78591
C	-2.66642	0.29658	-0.09145
C	-2.72026	0.19034	1.23853
Rh	0.97452	-0.08854	-0.74551
P	1.50529	1.80256	0.27011
C	3.26557	1.77368	0.90748
C	4.12530	0.82866	0.08128
P	3.19279	-0.74821	-0.16031
C	0.55719	2.06933	1.82022
C	1.40833	3.40216	-0.61704
C	3.22083	-1.55915	1.47957
C	4.26427	-1.76702	-1.22188
C	0.97138	3.42061	-1.94470
C	0.90975	4.62131	-2.65150
C	1.29093	5.81223	-2.03819
C	1.73762	5.80277	-0.71560
C	1.79754	4.60513	-0.00905
C	-0.29316	3.15715	2.04257

C	-1.04526	3.23456	3.21646
C	-0.95134	2.23162	4.17785
C	-0.10998	1.13873	3.95853
C	0.63072	1.05186	2.78481
C	3.81381	-2.09084	-2.50591
C	4.62669	-2.83923	-3.35659
C	5.88151	-3.26822	-2.92896
C	6.32756	-2.95670	-1.64326
C	5.52020	-2.21095	-0.78943
C	4.11564	-1.22374	2.50264
C	4.02903	-1.84847	3.74636
C	3.05230	-2.81628	3.97498
C	2.16121	-3.15863	2.95734
C	2.23701	-2.53183	1.71596
H	-8.75175	-3.22441	0.95258
H	-9.14888	-0.90267	0.16169
H	-6.51136	-4.26588	0.87196
H	-3.14200	-0.49128	-2.02780
H	-2.17859	-2.41082	-0.74270
H	-3.86352	-3.96119	0.13563
H	-7.53971	1.37106	-1.02981
H	-0.98851	0.00211	-2.29355
H	-2.40035	1.82727	-1.55873
H	-1.44956	2.05689	-0.04636
H	-3.34489	-0.57297	1.71233
H	-2.15062	0.85825	1.88808
H	3.21317	1.43360	1.95303
H	3.66980	2.79673	0.91635
H	4.28866	1.22390	-0.93433
H	5.11900	0.67078	0.53021
H	0.68971	2.47553	-2.41771
H	0.56457	4.62308	-3.68793

H	1.24320	6.75319	-2.59155
H	2.03960	6.73498	-0.23222
H	2.14068	4.60995	1.02941
H	-0.37919	3.94842	1.29482
H	-1.71154	4.08582	3.37447
H	-1.54142	2.29434	5.09513
H	-0.04098	0.33877	4.69926
H	1.25395	0.17085	2.60425
H	2.81329	-1.77755	-2.81489
H	4.27023	-3.09655	-4.35670
H	6.51443	-3.85793	-3.59676
H	7.30656	-3.30305	-1.30331
H	5.86305	-1.98514	0.22421
H	4.88529	-0.46507	2.34089
H	4.72844	-1.57593	4.54045
H	2.98383	-3.30322	4.95084
H	1.39202	-3.91446	3.13270
H	1.52614	-2.77815	0.91881
Cl	0.42316	-2.32232	-1.43772

$[Rh(CO)_2Cl]_2$

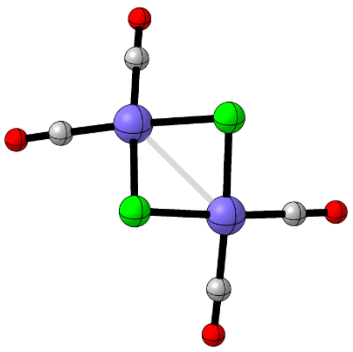

Energy: -1000591.9826448

Rh	-1.58208	-0.00001	0.10597

C	-2.70534	1.31790	-0.51602
C	-2.70529	-1.31794	-0.51605
Cl	-0.00021	-1.62054	0.92949
Cl	-0.00023	1.62063	0.92942
O	-3.38616	2.14756	-0.89880
O	-3.38605	-2.14765	-0.89884
Rh	1.58195	-0.00001	0.10607
C	2.70551	1.31764	-0.51588
C	2.70559	-1.31757	-0.51586
O	3.38677	2.14692	-0.89873
O	3.38676	-2.14695	-0.89868

dppe

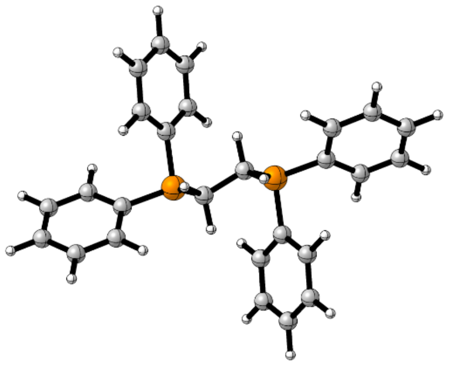

Energy: -1058563.1807515

P	1.63836	0.15615	-0.51855
C	0.64807	0.39994	1.04503
C	-0.64799	-0.39963	1.04500
P	-1.63831	-0.15591	-0.51857
C	3.28751	0.76480	0.00874
C	1.87940	-1.66708	-0.42614
C	-1.87958	1.66728	-0.42606
C	-3.28732	-0.76485	0.00881
C	1.19413	-2.46576	-1.34949
C	1.28420	-3.85663	-1.28670

C	2.06721	-4.46113	-0.30563
C	2.75931	-3.67268	0.61554
C	2.66469	-2.28480	0.55725
C	4.42611	0.30414	-0.67152
C	5.68945	0.79870	-0.36401
C	5.84108	1.77674	0.62018
C	4.71791	2.25248	1.29180
C	3.45124	1.75133	0.98995
C	-3.45083	-1.75159	0.98983
C	-4.71739	-2.25304	1.29163
C	-5.84068	-1.77737	0.62016
C	-5.68927	-0.79909	-0.36383
C	-4.42604	-0.30423	-0.67129
C	-2.66511	2.28491	0.55718
C	-2.75990	3.67278	0.61546
C	-2.06769	4.46132	-0.30555
C	-1.28442	3.85691	-1.28647
C	-1.19419	2.46605	-1.34926
H	0.41923	1.47629	1.11516
H	1.26006	0.12856	1.92142
H	-0.41911	-1.47597	1.11519
H	-1.26007	-0.12825	1.92133
H	0.57490	-1.98718	-2.11284
H	0.74172	-4.46940	-2.01080
H	2.14203	-5.55037	-0.25824
H	3.37671	-4.14393	1.38451
H	3.21304	-1.67068	1.27706
H	4.31957	-0.46166	-1.44513
H	6.56383	0.41890	-0.89845
H	6.83287	2.16700	0.86029
H	4.82449	3.01982	2.06273
H	2.58610	2.14081	1.53079

H	-2.58559	-2.14102	1.53056
H	-4.82379	-3.02056	2.06241
H	-6.83239	-2.16787	0.86022
H	-6.56375	-0.41932	-0.89813
H	-4.31967	0.46178	-1.44472
H	-3.21354	1.67073	1.27688
H	-3.37751	4.14394	1.38431
H	-2.14264	5.55055	-0.25817
H	-0.74186	4.46974	-2.01045
H	-0.57477	1.98754	-2.11250

38

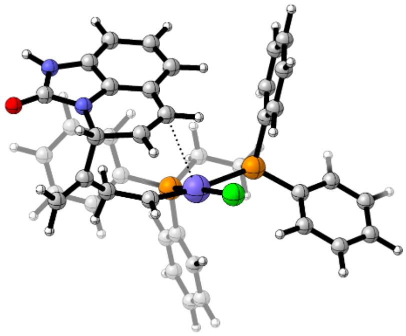

Energy: -1871611.8497488

C	2.32635	-2.45951	-2.51463
C	3.64728	-1.97799	-2.52566
C	4.18455	-1.55727	-1.31229
C	3.39570	-1.62599	-0.16340
C	2.10731	-2.13171	-0.11721
C	1.56526	-2.54697	-1.34253
N	4.12078	-1.17155	0.90634
C	3.55315	-1.04235	2.23927
C	2.23566	-1.78317	2.29736
C	1.53910	-2.21495	1.22189
N	5.39609	-1.03765	-0.88846

C	5.39335	-0.79215	0.48903
O	6.29652	-0.35977	1.16275
C	0.86100	0.59653	2.05942
C	2.24244	1.08020	1.77667
C	3.31455	0.41875	2.59701
C	3.99848	1.01556	3.57451
Rh	-0.65157	0.18433	1.01073
P	-0.25332	1.56427	-0.68094
C	-0.99324	0.85856	-2.23625
C	-2.42437	0.44249	-1.92205
P	-2.42479	-0.59604	-0.38982
C	1.40250	2.12462	-1.21176
C	-1.20214	3.13247	-0.55000
C	-2.10742	-2.30127	-0.97179
C	-4.17233	-0.62917	0.12836
C	-1.99233	3.34592	0.58404
C	-2.78244	4.49133	0.68639
C	-2.78522	5.43051	-0.34224
C	-1.99654	5.22576	-1.47647
C	-1.20982	4.08233	-1.58104
C	1.90318	3.36895	-0.80681
C	3.21158	3.73564	-1.11228
C	4.03216	2.86792	-1.83148
C	3.53857	1.62978	-2.23921
C	2.23546	1.25173	-1.92373
C	-4.49188	-0.24846	1.43553
C	-5.82507	-0.23519	1.84577
C	-6.83570	-0.59985	0.95888
C	-6.51714	-0.99145	-0.34298
C	-5.18867	-1.01104	-0.75684
C	-1.97656	-2.65256	-2.31934
C	-1.69855	-3.97221	-2.67842

C	-1.55209	-4.94862	-1.69554
C	-1.68399	-4.60253	-0.34942
C	-1.95556	-3.28707	0.01530
H	1.88410	-2.79692	-3.45475
H	4.22332	-1.94620	-3.45256
H	0.55429	-2.95350	-1.37712
H	4.26814	-1.46091	2.96663
H	1.79429	-1.88734	3.29074
H	0.55229	-2.65512	1.37051
H	6.22421	-0.88145	-1.44400
H	0.71988	0.39332	3.13885
H	2.21186	2.16125	2.01939
H	2.48364	1.02063	0.70525
H	4.77200	0.47974	4.13079
H	3.82005	2.06110	3.84023
H	-0.38026	-0.01263	-2.52044
H	-0.94598	1.59214	-3.05611
H	-3.03496	1.32736	-1.67784
H	-2.91466	-0.05832	-2.77063
H	-1.97699	2.59175	1.37866
H	-3.39848	4.64739	1.57502
H	-3.40353	6.32805	-0.26287
H	-1.99562	5.96281	-2.28322
H	-0.58727	3.93293	-2.46787
H	1.26633	4.05266	-0.24061
H	3.59306	4.70548	-0.78421
H	5.05896	3.15519	-2.06926
H	4.17357	0.94069	-2.79888
H	1.88342	0.25867	-2.21532
H	-3.68428	-0.00215	2.13074
H	-6.07215	0.05575	2.86947
H	-7.87905	-0.58857	1.28391

H	-7.30838	-1.28866	-1.03552
H	-4.94034	-1.33639	-1.77113
H	-2.07912	-1.90081	-3.10447
H	-1.59304	-4.23534	-3.73372
H	-1.32912	-5.98023	-1.97768
H	-1.56355	-5.36265	0.42610
H	-2.02688	-3.00063	1.07089
Cl	-1.39100	-1.21883	2.81976

5

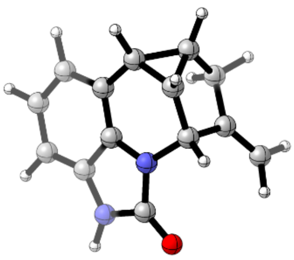

Energy: -454894.0971359

C	-3.21575	-0.99786	0.53735
C	-3.08478	0.40007	0.55101
C	-1.86266	0.92747	0.15320
C	-0.83920	0.05878	-0.24183
C	-0.94295	-1.31989	-0.26936
C	-2.17692	-1.84604	0.13843
N	0.25746	0.80325	-0.59984
C	1.56330	0.17823	-0.71461
C	1.43633	-1.25314	-1.25043
C	0.23341	-2.05320	-0.79756
N	-1.32717	2.19913	0.03209
C	0.00296	2.15325	-0.38877
O	0.76642	3.07931	-0.53332

C	1.55634	-2.18916	-0.07491
C	1.81910	-1.37083	1.18020
C	2.17272	0.00830	0.67225
C	2.91086	0.93530	1.28154
H	-4.16674	-1.43659	0.84721
H	-3.91272	1.03756	0.86626
H	-2.33303	-2.92740	0.13277
H	2.18365	0.81157	-1.36217
H	1.88316	-1.47611	-2.22122
H	-0.01531	-2.91034	-1.43080
H	-1.78144	3.07561	0.24374
H	2.07457	-3.14441	-0.18829
H	2.63278	-1.80229	1.78086
H	0.92537	-1.33314	1.82885
H	3.32413	0.76762	2.28022
H	3.10554	1.89915	0.80431

39’

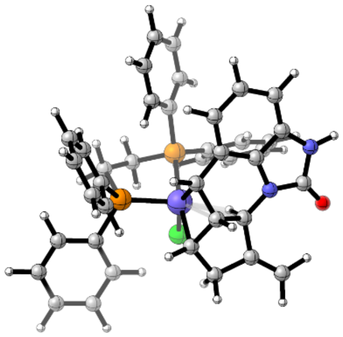

Energy: -1871631.6261176

C	-2.83703	2.77460	-2.27982
C	-3.97633	1.95741	-2.20834
C	-3.75933	0.59020	-2.10525
C	-2.45637	0.08581	-2.07335
C	-1.30691	0.87725	-2.07073

C	-1.54336	2.25748	-2.21648
N	-2.51783	-1.29713	-1.96629
C	-1.28005	-1.92901	-1.62546
C	-0.09670	-1.25219	-2.39368
C	-0.00982	0.19165	-1.94878
N	-4.58388	-0.51511	-1.97690
C	-3.85601	-1.69274	-1.83803
O	-4.31370	-2.79568	-1.65900
C	1.02405	-1.96918	-1.67366
C	0.57887	-3.42452	-1.54631
C	-0.92976	-3.38639	-1.73545
C	-1.73219	-4.41824	-1.98977
Rh	0.37365	-0.72874	-0.09792
P	2.28022	0.22196	0.48113
C	2.04141	1.10959	2.10866
C	0.76287	0.67128	2.82097
P	-0.60017	0.59430	1.57496
C	2.98612	1.45943	-0.67448
C	3.73303	-0.86148	0.73057
C	-1.03126	2.35983	1.36728
C	-2.07022	-0.12304	2.35905
C	3.61862	-2.25061	0.64648
C	4.74779	-3.04995	0.82515
C	5.98663	-2.47042	1.08793
C	6.10327	-1.08183	1.17284
C	4.98189	-0.27886	0.99047
C	3.43693	0.96505	-1.90846
C	3.94067	1.82519	-2.87776
C	4.00585	3.19730	-2.63106
C	3.57267	3.69695	-1.40620
C	3.06655	2.83448	-0.43247
C	-3.22181	-0.20416	1.56232

C	-4.36640	-0.82961	2.04408
C	-4.37148	-1.38096	3.32616
C	-3.22966	-1.30258	4.12047
C	-2.07830	-0.67980	3.63966
C	-2.03937	2.98876	2.10813
C	-2.26761	4.35522	1.96064
C	-1.48513	5.10928	1.08531
C	-0.48238	4.48857	0.34305
C	-0.26962	3.11828	0.47316
H	-2.97018	3.85433	-2.37714
H	-4.98106	2.38219	-2.23378
H	-0.69078	2.93492	-2.28236
H	-1.18067	-1.75285	-0.48473
H	-0.23921	-1.43864	-3.48219
H	0.83810	0.75488	-2.35679
H	-5.59335	-0.52900	-1.98131
H	0.83374	-3.86686	-0.56932
H	1.03453	-4.05699	-2.32854
H	-2.81042	-4.29079	-2.09401
H	-1.30801	-5.42285	-2.07601
H	1.99490	2.18788	1.89937
H	2.92989	0.93523	2.73340
H	0.86662	-0.35838	3.19590
H	0.51954	1.35282	3.65039
H	2.63626	-2.69344	0.47479
H	4.65188	-4.13634	0.76215
H	6.86819	-3.10112	1.22685
H	7.07396	-0.62262	1.37445
H	5.08290	0.80941	1.03438
H	3.39410	-0.10868	-2.10613
H	4.28415	1.42160	-3.83295
H	4.39947	3.87438	-3.39260

H	3.63100	4.76795	-1.19788
H	2.73850	3.25752	0.51807
H	-3.21513	0.22231	0.55454
H	-5.25260	-0.90089	1.40946
H	-5.26628	-1.88216	3.70245
H	-3.22782	-1.74186	5.12074
H	-1.18291	-0.64633	4.26237
H	-2.65038	2.40406	2.80010
H	-3.06084	4.83657	2.53782
H	-1.66473	6.18162	0.97647
H	0.12402	5.06699	-0.35882
H	0.47922	2.61959	-0.14498
Cl	0.45560	-2.40396	1.69147
H	2.03420	-1.81470	-2.07071

39

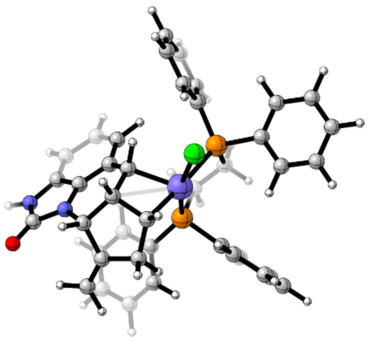

Energy: -1871645.9263116

C	1.89109	-3.17721	-1.92638
C	3.29024	-3.07010	-1.83973
C	3.80150	-2.36889	-0.75737
C	2.93011	-1.77293	0.16884
C	1.54813	-1.88715	0.12154
C	1.04161	-2.62314	-0.96686
N	3.67982	-1.15396	1.13278

C	3.07967	-0.34439	2.17042
C	1.57662	-0.67973	2.39053
C	0.78804	-1.35597	1.26569
N	5.07296	-2.07464	-0.30498
C	5.03217	-1.33681	0.87859
O	5.97570	-0.95094	1.53032
C	0.82951	0.67160	2.46116
C	1.84741	1.79378	2.28850
C	3.15260	1.15307	1.90369
C	4.24072	1.78900	1.46816
Rh	-0.57925	0.16877	1.03802
P	0.22848	1.18921	-0.78994
C	-0.47736	0.38033	-2.30457
C	-1.97156	0.16689	-2.12212
P	-2.29944	-0.58216	-0.45686
C	1.92844	1.57435	-1.32682
C	-0.59653	2.82843	-0.77483
C	-2.43135	-2.38492	-0.69888
C	-4.01209	-0.05204	-0.11558
C	-1.06038	3.32832	0.44910
C	-1.74572	4.54193	0.50362
C	-1.97182	5.26582	-0.66495
C	-1.50461	4.77982	-1.88745
C	-0.81817	3.56982	-1.94247
C	2.46599	2.85689	-1.16792
C	3.77485	3.12186	-1.56161
C	4.56170	2.10977	-2.10799
C	4.02651	0.83465	-2.28197
C	2.71430	0.56738	-1.90272
C	-4.20742	1.27408	0.29107
C	-5.48959	1.74803	0.54855
C	-6.58765	0.89703	0.41926

C	-6.39707	-0.42675	0.02836
C	-5.11416	-0.90229	-0.24039
C	-2.46254	-3.00620	-1.95232
C	-2.48880	-4.39733	-2.04637
C	-2.49440	-5.17653	-0.89095
C	-2.47747	-4.56141	0.36129
C	-2.44042	-3.17353	0.46205
H	1.45503	-3.73874	-2.75573
H	3.93965	-3.53653	-2.58275
H	-0.03281	-2.78333	-1.04629
H	3.63267	-0.55552	3.10034
H	1.46367	-1.26667	3.31405
H	0.08950	-2.10990	1.65379
H	5.94925	-2.38591	-0.69764
H	0.23804	0.77411	3.37958
H	1.97527	2.32427	3.25109
H	1.56218	2.57568	1.56506
H	5.17568	1.26190	1.26954
H	4.22659	2.87134	1.31906
H	0.05303	-0.58058	-2.40658
H	-0.24490	0.97957	-3.19849
H	-2.49598	1.13582	-2.10511
H	-2.41682	-0.41447	-2.94281
H	-0.87974	2.75795	1.36773
H	-2.10371	4.91861	1.46442
H	-2.51184	6.21480	-0.62552
H	-1.67668	5.34885	-2.80413
H	-0.44774	3.20336	-2.90319
H	1.85920	3.65449	-0.73397
H	4.18602	4.12555	-1.43062
H	5.59273	2.31701	-2.40379
H	4.63126	0.03576	-2.71681

H	2.30677	-0.43229	-2.06050
H	-3.34724	1.93776	0.41325
H	-5.63040	2.78407	0.86541
H	-7.59406	1.26558	0.63174
H	-7.25358	-1.09841	-0.06742
H	-4.96940	-1.94183	-0.54312
H	-2.45409	-2.41248	-2.86841
H	-2.50441	-4.87344	-3.02971
H	-2.50995	-6.26639	-0.96655
H	-2.48121	-5.16732	1.27036
H	-2.40720	-2.68097	1.44074
Cl	-1.93833	-0.59222	2.84329

37’

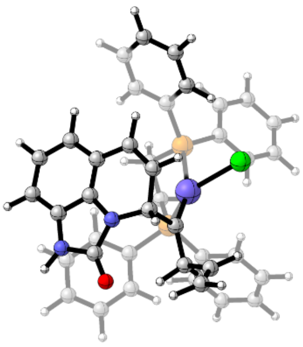

Energy: -1871611.3254458

C	1.78584	-4.11749	-1.99106
C	3.03414	-3.48114	-1.92256
C	3.25934	-2.63438	-0.83971
C	2.25038	-2.44996	0.10272
C	1.03412	-3.11645	0.09564
C	0.80495	-3.95828	-1.00253
N	2.69715	-1.59333	1.07792
C	1.79457	-1.09168	2.11182
C	0.59163	-2.01391	2.22476

C	0.21521	-2.90199	1.28128
N	4.32127	-1.87368	-0.38533
C	4.00278	-1.20687	0.79348
O	4.71929	-0.45850	1.42483
C	1.31526	0.31275	1.74095
C	2.26385	1.61990	3.53414
C	3.28819	1.30405	4.32934
C	2.27247	1.42354	2.03934
H	1.58468	-4.78416	-2.83273
H	3.79575	-3.65470	-2.68475
H	-0.13670	-4.50739	-1.06958
H	2.34479	-1.05441	3.06905
H	-0.01977	-1.86692	3.11706
H	-0.70339	-3.47812	1.40806
H	5.19815	-1.70479	-0.85571
H	1.33293	2.01412	3.95756
H	4.21030	0.88188	3.91657
H	3.23422	1.45662	5.41077
H	1.93963	2.35331	1.55461
H	3.29727	1.17588	1.71755
Rh	-0.37011	0.25761	0.94629
P	0.36609	1.30245	-0.86574
C	-0.78471	1.01344	-2.30958
C	-1.47841	-0.33622	-2.18534
P	-2.14991	-0.41502	-0.46490
Cl	-1.94126	0.41377	2.74449
C	0.32419	3.12544	-0.68430
C	2.02110	1.00635	-1.60590
C	-3.65810	0.60981	-0.58580
C	-2.78138	-2.10506	-0.23505
C	-4.83749	0.13335	-1.17089
C	-5.94254	0.97138	-1.29544

C	-5.87671	2.28808	-0.83688
C	-4.70737	2.76336	-0.24631
C	-3.60181	1.92514	-0.11309
C	-3.27596	-2.43060	1.03715
C	-3.73026	-3.72006	1.29657
C	-3.68985	-4.69572	0.29823
C	-3.19914	-4.37541	-0.96583
C	-2.74749	-3.08257	-1.23460
C	-0.19745	3.66906	0.49464
C	-0.30867	5.05177	0.63797
C	0.09682	5.89515	-0.39461
C	0.61751	5.35716	-1.57295
C	0.73338	3.97732	-1.71857
C	3.12961	1.77729	-1.22987
C	4.38107	1.53968	-1.79241
C	4.53960	0.54231	-2.75401
C	3.44397	-0.23305	-3.12962
C	2.19774	-0.01546	-2.54751
H	-1.52814	1.82567	-2.26125
H	-0.23654	1.12438	-3.25716
H	-2.26751	-0.45981	-2.94398
H	-0.75972	-1.16684	-2.28270
H	-4.89361	-0.90151	-1.52007
H	-6.86265	0.59454	-1.74877
H	-6.74621	2.94300	-0.93305
H	-4.65612	3.78934	0.12547
H	-2.69022	2.27743	0.37433
H	-3.26707	-1.66874	1.82434
H	-4.11032	-3.96861	2.29032
H	-4.04135	-5.70892	0.50749
H	-3.16733	-5.13496	-1.75090
H	-2.36063	-2.84771	-2.22827

H	-0.51153	2.98483	1.29243
H	-0.71326	5.46887	1.56310
H	0.01020	6.97865	-0.28220
H	0.93717	6.01801	-2.38229
H	1.15238	3.56101	-2.63884
H	3.01244	2.58294	-0.50343
H	5.23460	2.14640	-1.48214
H	5.51727	0.37250	-3.21233
H	3.55665	-1.02533	-3.87327
H	1.36159	-0.65747	-2.83316

TS1'

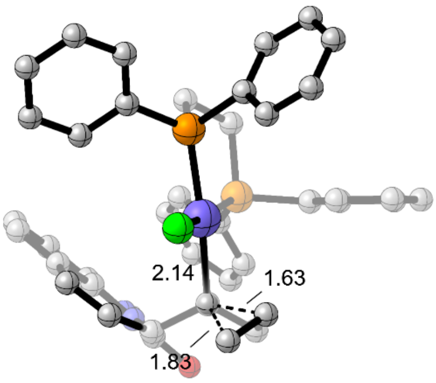

Energy: -1871586.8317495

C	2.84067	-3.75549	-1.78592
C	3.71573	-2.65825	-1.81442
C	3.68317	-1.78810	-0.72817
C	2.77377	-2.02447	0.29955
C	1.95873	-3.13926	0.39489
C	1.99355	-4.01428	-0.70129
N	2.93273	-1.06424	1.26435
C	2.08069	-0.98184	2.42323
C	1.34773	-2.29048	2.59714
C	1.26091	-3.25799	1.66680

N	4.41137	-0.68326	-0.32281
C	3.95904	-0.19918	0.89849
O	4.37072	0.76140	1.51255
C	1.10613	0.24023	2.28862
C	0.40569	1.41149	3.18116
C	0.51112	0.29908	4.01721
C	1.70991	1.62722	2.55435
H	2.84491	-4.44944	-2.62932
H	4.40276	-2.50930	-2.64899
H	1.36925	-4.91073	-0.70253
H	2.74228	-0.79741	3.29014
H	0.79879	-2.39968	3.53240
H	0.65632	-4.14469	1.87005
H	5.05516	-0.14458	-0.88612
H	-0.54071	1.83085	2.83556
H	1.42433	0.14764	4.59793
H	-0.38117	-0.28736	4.25007
H	1.74648	2.31626	1.71013
H	2.59040	1.65587	3.20725
Rh	-0.40269	-0.06211	0.80291
P	0.34102	1.34244	-0.73522
C	-0.67820	1.19038	-2.31137
C	-1.32308	-0.18101	-2.38884
P	-1.98818	-0.55562	-0.70524
Cl	-1.70181	-1.28628	2.41413
C	-0.03565	3.10951	-0.34221
C	2.04688	1.44254	-1.43973
C	-3.52136	0.44611	-0.58908
C	-2.57862	-2.27434	-0.85973
C	-4.13532	1.05213	-1.69116
C	-5.27268	1.84211	-1.52210
C	-5.81320	2.02550	-0.25150

C	-5.21197	1.41626	0.85072
C	-4.06983	0.63692	0.68803
C	-3.87782	-2.60242	-1.25701
C	-4.24616	-3.93984	-1.39738
C	-3.32094	-4.95151	-1.14391
C	-2.02494	-4.62538	-0.74307
C	-1.65631	-3.29179	-0.59364
C	-1.08318	3.33324	0.56169
C	-1.52127	4.62629	0.83859
C	-0.91533	5.71563	0.21447
C	0.11473	5.50285	-0.70122
C	0.54749	4.20849	-0.98564
C	3.03219	2.28645	-0.91115
C	4.28675	2.38444	-1.51052
C	4.58288	1.63223	-2.64698
C	3.62391	0.75687	-3.15988
C	2.37373	0.65715	-2.55531
H	-1.45259	1.96879	-2.22948
H	-0.06612	1.43593	-3.19155
H	-2.08842	-0.25445	-3.17715
H	-0.58039	-0.97311	-2.57806
H	-3.73066	0.91625	-2.69643
H	-5.73737	2.31570	-2.39041
H	-6.70398	2.64466	-0.11963
H	-5.63368	1.55300	1.84965
H	-3.58315	0.16288	1.54740
H	-4.60727	-1.81087	-1.44634
H	-5.26462	-4.19288	-1.70215
H	-3.61370	-5.99894	-1.25110
H	-1.30142	-5.41708	-0.53238
H	-0.65389	-3.01826	-0.25688
H	-1.56567	2.46208	1.01919

H	-2.34387	4.78062	1.54105
H	-1.25186	6.73187	0.43379
H	0.58312	6.35213	-1.20490
H	1.34343	4.05766	-1.71809
H	2.82475	2.89184	-0.02794
H	5.03538	3.05284	-1.07982
H	5.55879	1.72540	-3.13040
H	3.84855	0.14711	-4.03845
H	1.64355	-0.04490	-2.96419

TS1

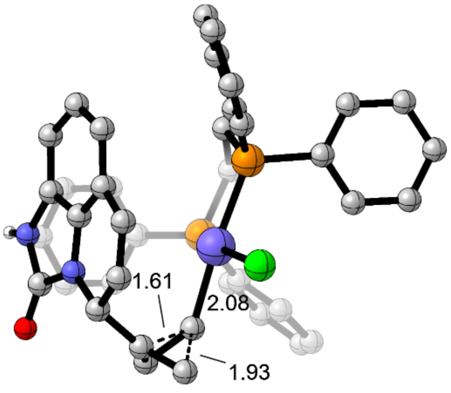

Energy: -1871595.7357072

C	-0.21845	-3.59439	-2.02475
C	1.13071	-3.25676	-2.20916
C	1.84908	-2.86054	-1.08379
C	1.19522	-2.77375	0.14165
C	-0.11217	-3.17107	0.36431
C	-0.83010	-3.58293	-0.76505
N	2.09981	-2.38936	1.10201
C	1.72766	-2.22716	2.48777
C	0.35326	-2.80954	2.72945
C	-0.50134	-3.19369	1.76629
N	3.17369	-2.56239	-0.81441

C	3.36568	-2.27539	0.52838
O	4.40379	-1.98534	1.08393
H	-0.80585	-3.90824	-2.89049
H	1.60017	-3.32737	-3.19173
H	-1.86966	-3.89884	-0.66252
H	2.47088	-2.77344	3.10281
H	0.04398	-2.86765	3.77404
H	-1.49720	-3.54971	2.03664
H	3.90663	-2.39368	-1.49066
Rh	-0.24443	0.31201	0.90009
Cl	-1.73604	-0.06531	2.74425
C	1.86537	-0.79594	3.00760
C	1.13950	-0.26654	4.07629
C	2.74165	0.25730	2.45304
C	1.26064	0.52840	2.32415
H	1.57289	0.51381	4.70417
H	0.16929	-0.68144	4.36027
H	3.32427	0.84831	3.17093
H	3.28320	-0.00842	1.54716
H	0.88669	1.39947	2.88482
P	0.90156	1.27113	-0.71954
P	-1.94463	0.23154	-0.58356
C	-0.23171	1.60454	-2.16645
C	2.41032	0.61286	-1.55706
C	1.45730	2.98847	-0.33962
C	-1.26012	0.49300	-2.28447
C	-3.05678	-1.21214	-0.76216
C	-3.15142	1.61148	-0.44726
H	-0.73377	2.55469	-1.92130
H	0.34091	1.76795	-3.09203
C	2.32775	-0.07528	-2.77443
C	3.67413	0.78897	-0.97611

C	0.90344	3.63494	0.77047
C	2.32938	3.69960	-1.17459
H	-0.79662	-0.46258	-2.58240
H	-2.04804	0.74436	-3.01167
C	-3.37576	-1.77358	-2.00380
C	-3.60045	-1.77058	0.40261
C	-4.21899	1.74538	-1.34380
C	-2.98061	2.56061	0.56446
H	1.35867	-0.24133	-3.24813
C	3.47636	-0.55767	-3.40048
C	4.82168	0.29424	-1.59013
H	3.76714	1.34768	-0.04274
H	0.20453	3.07612	1.39964
C	1.22415	4.96196	1.05201
C	2.64989	5.02576	-0.89320
H	2.76755	3.21223	-2.04917
H	-2.95634	-1.36226	-2.92381
C	-4.21908	-2.88171	-2.08188
C	-4.44386	-2.87574	0.31950
H	-3.33233	-1.34018	1.37329
H	-4.38156	0.99011	-2.11831
C	-5.08908	2.82735	-1.24348
C	-3.85528	3.64217	0.66585
H	-2.16451	2.41790	1.27749
H	3.39174	-1.07708	-4.35862
C	4.72806	-0.37688	-2.81020
H	5.79334	0.43556	-1.11238
H	0.78478	5.45385	1.92329
C	2.10091	5.65856	0.22247
H	3.33331	5.56956	-1.55009
H	-4.45673	-3.31463	-3.05681
C	-4.75326	-3.43697	-0.92053

H	-4.86056	-3.30573	1.23383
H	-5.91963	2.92571	-1.94692
C	-4.90526	3.77985	-0.23941
H	-3.71730	4.37808	1.46171
H	5.62914	-0.74989	-3.30338
H	2.35591	6.69813	0.44259
H	-5.41169	-4.30694	-0.98173
H	-5.59049	4.62751	-0.15941

TS2

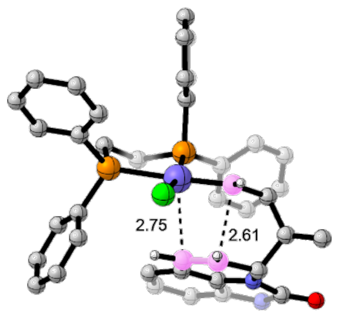

Energy: -1871609.9776872

C	2.36586	-2.45304	-2.67785
C	3.70286	-2.03933	-2.54827
C	4.12146	-1.61538	-1.29055
C	3.20623	-1.60475	-0.23605
C	1.89226	-2.03233	-0.33069
C	1.47350	-2.46363	-1.59904
N	3.83744	-1.16798	0.89950
C	3.18691	-1.11571	2.20051
C	1.77732	-1.65128	2.08726
C	1.16011	-2.03083	0.93028
N	5.30973	-1.16441	-0.74471
C	5.17800	-0.90819	0.62382

O	6.03944	-0.55936	1.39319
C	0.80078	0.76824	2.18198
C	2.21613	1.21039	2.01807
C	3.15354	0.29846	2.76180
C	3.85865	0.63976	3.84084
Rh	-0.53365	0.13438	0.98972
P	-0.09206	1.48215	-0.71422
C	-0.78307	0.73041	-2.26750
C	-2.23002	0.35211	-1.98018
P	-2.30332	-0.61683	-0.39996
C	1.57745	2.04558	-1.19408
C	-1.04567	3.05197	-0.65371
C	-2.21165	-2.37378	-0.90169
C	-4.03589	-0.41665	0.13326
C	-1.91732	3.27595	0.41610
C	-2.71425	4.42056	0.44753
C	-2.64266	5.34870	-0.58852
C	-1.77170	5.13360	-1.65883
C	-0.97763	3.99100	-1.69262
C	2.07475	3.27075	-0.72777
C	3.38577	3.64980	-1.00233
C	4.21475	2.81299	-1.74909
C	3.72519	1.59540	-2.21750
C	2.41650	1.20711	-1.93758
C	-4.30485	0.21971	1.34866
C	-5.62608	0.43164	1.74139
C	-6.67622	0.00975	0.92853
C	-6.40947	-0.63738	-0.27933
C	-5.09273	-0.85416	-0.67501
C	-2.10408	-2.80898	-2.22664
C	-1.97603	-4.16899	-2.51298
C	-1.95664	-5.10255	-1.47939

C	-2.06676	-4.67360	-0.15546
C	-2.19038	-3.31854	0.13674
H	2.01757	-2.79837	-3.65382
H	4.38225	-2.06538	-3.40233
H	0.45291	-2.82482	-1.73574
H	3.76377	-1.73813	2.90760
H	1.22640	-1.75644	3.02501
H	0.16496	-2.47526	0.98453
H	6.20388	-1.08346	-1.20612
H	0.53070	0.66881	3.25112
H	2.29183	2.22900	2.44215
H	2.51300	1.26924	0.96119
H	4.52304	-0.07704	4.32975
H	3.81004	1.64889	4.25795
H	-0.17543	-0.16103	-2.49611
H	-0.69603	1.43233	-3.11162
H	-2.82970	1.25782	-1.79393
H	-2.70653	-0.17560	-2.81980
H	-1.96165	2.52911	1.21531
H	-3.39532	4.58342	1.28605
H	-3.26693	6.24530	-0.56515
H	-1.71216	5.86179	-2.47140
H	-0.29119	3.83311	-2.52941
H	1.43118	3.92896	-0.13916
H	3.76341	4.60438	-0.62839
H	5.24411	3.11003	-1.96268
H	4.36536	0.93200	-2.80255
H	2.06002	0.23727	-2.29196
H	-3.47022	0.50529	1.99405
H	-5.83389	0.92084	2.69597
H	-7.71035	0.17544	1.24081
H	-7.23209	-0.97989	-0.91179

H	-4.88473	-1.37739	-1.61280
H	-2.10677	-2.09205	-3.05027
H	-1.88847	-4.49780	-3.55140
H	-1.85228	-6.16652	-1.70464
H	-2.04758	-5.40080	0.65975
H	-2.24735	-2.97130	1.17546
Cl	-1.49212	-1.10151	2.86329

TS2'

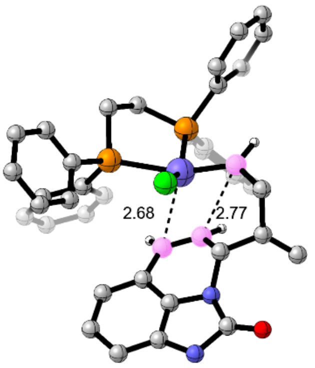

Energy: -1871587.6484593

C	5.06628	0.35853	1.47371
C	5.67941	-0.84914	1.09514
C	4.84842	-1.89641	0.71547
C	3.46511	-1.71248	0.71459
C	2.83317	-0.53184	1.10059
C	3.68037	0.52294	1.49091
N	2.84050	-2.87625	0.30707
C	1.52596	-2.60764	-0.28388
C	0.74842	-1.72744	0.69665
C	1.39045	-0.63882	1.25055
N	5.03578	-3.19742	0.28804
C	3.82404	-3.81619	-0.03033
O	3.69245	-4.92658	-0.48366
C	-1.13234	-1.97572	-1.31640

C	-0.33905	-3.13538	-1.84017
C	0.63397	-3.69091	-0.80599
C	0.62813	-4.96861	-0.42526
Rh	-0.43195	-0.33989	-0.68553
P	-2.49047	0.20410	0.06839
C	-2.69530	2.05915	0.16885
C	-1.67036	2.76601	-0.70596
P	-0.01178	2.00416	-0.38582
C	-3.07097	-0.40220	1.70083
C	-3.88518	-0.25852	-1.03821
C	0.36027	2.67210	1.29262
C	1.16943	2.90340	-1.43094
C	-3.68203	-0.14453	-2.42048
C	-4.72231	-0.41133	-3.30681
C	-5.96898	-0.80945	-2.82330
C	-6.17222	-0.93759	-1.45048
C	-5.13629	-0.66054	-0.55870
C	-2.80427	-1.74364	2.00948
C	-3.19487	-2.28340	3.23134
C	-3.84881	-1.48515	4.17028
C	-4.12005	-0.15103	3.87396
C	-3.73970	0.38703	2.64435
C	2.48863	2.43090	-1.45854
C	3.45412	3.10376	-2.19965
C	3.10907	4.23617	-2.93824
C	1.79481	4.69821	-2.92946
C	0.82647	4.03650	-2.17424
C	1.21371	3.76419	1.49093
C	1.47448	4.23296	2.77853
C	0.87875	3.62779	3.88346
C	0.02223	2.54317	3.69678
C	-0.22571	2.06400	2.41311

H	5.70187	1.19366	1.77663
H	6.76611	-0.95069	1.09969
H	3.24354	1.46789	1.82315
H	1.74772	-1.96216	-1.15317
H	-0.10183	-2.17551	1.21718
H	0.90987	-0.06181	2.04048
H	5.91240	-3.68190	0.16135
H	-2.21147	-2.20712	-1.26412
H	0.26515	-2.74906	-2.68580
H	-0.98634	-3.94640	-2.20611
H	1.37792	-5.35869	0.26445
H	-0.08943	-5.67450	-0.85254
H	-2.55228	2.36048	1.21708
H	-3.72531	2.32102	-0.11486
H	-1.88147	2.60262	-1.77520
H	-1.66729	3.84951	-0.50795
H	-2.68815	0.12646	-2.79103
H	-4.55324	-0.32098	-4.38244
H	-6.78175	-1.02952	-3.51967
H	-7.14401	-1.25889	-1.06781
H	-5.30272	-0.76453	0.51606
H	-2.28268	-2.36938	1.28000
H	-2.97907	-3.33075	3.45474
H	-4.14685	-1.90426	5.13414
H	-4.63663	0.47834	4.60267
H	-3.97573	1.43068	2.42721
H	2.75313	1.52661	-0.90693
H	4.47971	2.72811	-2.21303
H	3.86690	4.75645	-3.52909
H	1.51847	5.58062	-3.51150
H	-0.19754	4.41526	-2.17123
H	1.68606	4.24643	0.63247

H	2.15039	5.08043	2.91650
H	1.08681	3.99641	4.89065
H	-0.44554	2.05406	4.55457
H	-0.86789	1.18826	2.28615
Cl	1.16505	-0.29239	-2.52114

TS3

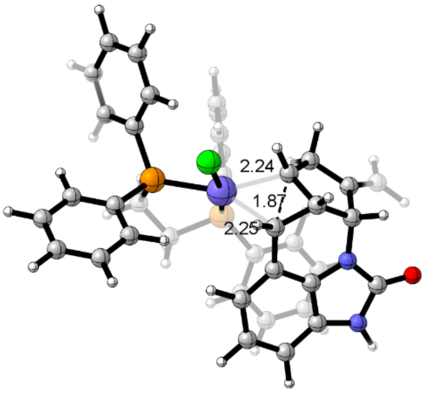

Energy: -1871631.5284018

C	2.54753	-3.10487	-1.58391
C	3.89302	-2.70100	-1.55832
C	4.27883	-1.84639	-0.53550
C	3.33627	-1.43882	0.41696
C	2.01258	-1.83749	0.42551
C	1.62269	-2.68811	-0.62455
N	3.96534	-0.64895	1.34453
C	3.27944	-0.08333	2.49032
C	1.93376	-0.78802	2.74372
C	1.21652	-1.44364	1.59878
N	5.46826	-1.25821	-0.14458
C	5.30613	-0.50587	1.01707
O	6.15191	0.12641	1.61071
C	0.82639	0.18214	2.43408
C	1.43699	1.55744	2.22029

C	2.93218	1.39162	2.31166
C	3.84361	2.36558	2.28079
Rh	-0.60957	-0.39721	0.81649
P	0.19956	0.66821	-0.97595
C	-0.80343	0.09065	-2.43031
C	-2.26567	0.30565	-2.08251
P	-2.56109	-0.34390	-0.36287
C	1.90303	0.69932	-1.65753
C	-0.22204	2.46281	-0.99108
C	-3.33152	-1.97432	-0.67860
C	-3.92574	0.70731	0.26243
C	-1.08680	2.95853	-0.00808
C	-1.51521	4.28479	-0.04713
C	-1.07467	5.12854	-1.06456
C	-0.20442	4.64464	-2.04332
C	0.21966	3.31876	-2.00890
C	2.87588	1.49974	-1.04308
C	4.16563	1.57504	-1.55656
C	4.50070	0.86280	-2.70837
C	3.54117	0.06858	-3.32957
C	2.25151	-0.01909	-2.80574
C	-4.14284	0.77219	1.64552
C	-5.15534	1.58107	2.15614
C	-5.95878	2.33331	1.29977
C	-5.74980	2.27055	-0.07645
C	-4.73901	1.46190	-0.59367
C	-4.60712	-2.07348	-1.24672
C	-5.14382	-3.32251	-1.54808
C	-4.41163	-4.48031	-1.28228
C	-3.14651	-4.38615	-0.70593
C	-2.60820	-3.13740	-0.39980
H	2.21742	-3.77771	-2.37867

H	4.59869	-3.04623	-2.31563
H	0.58884	-3.03566	-0.67097
H	3.94731	-0.20604	3.35593
H	1.89219	-1.32930	3.69435
H	0.51927	-2.20836	1.95521
H	6.36202	-1.32147	-0.60898
H	-0.03299	0.11395	3.10821
H	1.07487	2.20159	3.04097
H	1.12010	2.06734	1.29987
H	4.90906	2.13556	2.36363
H	3.54746	3.41151	2.15477
H	-0.58340	-0.98076	-2.56682
H	-0.51593	0.62914	-3.34698
H	-2.48609	1.38384	-2.07454
H	-2.94163	-0.17999	-2.80159
H	-1.42185	2.28546	0.78808
H	-2.19707	4.65508	0.72190
H	-1.40671	6.16919	-1.09580
H	0.14657	5.30636	-2.83897
H	0.90516	2.94627	-2.77492
H	2.62963	2.07959	-0.15472
H	4.91043	2.19175	-1.04816
H	5.51118	0.92732	-3.11939
H	3.79521	-0.49846	-4.22821
H	1.52374	-0.66270	-3.30036
H	-3.50901	0.17783	2.31123
H	-5.31447	1.62416	3.23630
H	-6.74918	2.96989	1.70499
H	-6.37495	2.85575	-0.75521
H	-4.59028	1.43146	-1.67498
H	-5.19277	-1.17089	-1.44159
H	-6.14228	-3.39324	-1.98629

H	-4.83559	-5.46005	-1.51578
H	-2.57762	-5.29082	-0.47886
H	-1.63698	-3.04849	0.09180
Cl	-1.82781	-1.57396	2.54413

References

[1] Kerner, M. J.; Wipf, P. "Semipinacol-Type Rearrangements of [3-(Arylsulfonyl)bicyclo[1.1.0]butan-1-yl]alkanols." *Org. Lett.* **2021**, *23*(9), 3615-3619.

[2] Zhao, H.; Wu, Y.; Ci, C.; Tan, Z.; Yang, J.; Jiang, H.; Dixneuf, P.H.; Zhang, M. "Intermolecular Diastereoselective Annulation of Azaarenes into Fused N-Heterocycles by Ru(II) Reductive Catalysis." *Nat. Commun*. **2022**, *13*, 2393.

[3] Grozavu, A.; Hepburn, H. B.; Smith, P. J.; Potukuchi, H. K.; Lindsay-Scott, P. J.; Donohoe, T. J. "The Reductive C3 Functionalization of Pyridinium and Quinolinium Salts through Iridium-Catalysed Interrupted Transfer Hydrogenation." *Nat. Chem*. **2019**, *11*, 242–247.

[4] Guan, R.; Zhao, H.; Zhang, M. "Construction of Fused Tetrahydroquinolines by Catalytic Hydride-Transfer-Initiated Tandem Functionalization of Quinolines." *Org. Lett.* **2022**, *24*(16), 3048-3052.

[5] Yadav, S.; Chaudhary, D.; Maurya, N.K.; Kumar, D.; Ishua, K.; Kuram, M.R. "Transfer Hydrogenation of Pyridinium and Quinolinium Species using Ethanol as a Hydrogen Source to Access Saturated N-Heterocycles." *Chem. Commun*. **2022**, *58*, 4255-4258.

[6] Frisch, M. J., Trucks, G. W., Schlegel, H. B., Scuseria, G. E., Robb, M. A., Cheeseman, J. R., Scalmani, G., Barone, V., Petersson, G. A., Nakatsuji, H., Li, X., Caricato, M., Marenich, A. V., Bloino, J., Janesko, B. G., Gomperts, R., Mennucci, B., Hratchian, H. P., Ortiz, J. V., Izmaylov, A. F., Sonnenberg, J. L., Williams-Young, D., Ding, F., Lipparini, F., Egidi, F., Goings, J., Peng, B., Petrone, A., Henderson, T., Ranasinghe, D., Zakrzewski, V. G., Gao, J., Rega, N., Zheng, G., Liang, W., Hada, M., Ehara, M., Toyota, K., Fukuda, R., Hasegawa, J., Ishida, M., Nakajima, T., Honda, Y., Kitao, O., Nakai, H., Vreven, T., Throssell, K., Montgomery Jr. J. A., Peralta, J. E., Ogliaro, F., Bearpark, M. J., Heyd, J. J., Brothers, E. N., Kudin, K. N., Staroverov, V. N., Keith, T. A., Kobayashi, R., Normand, J., Raghavachari, K., Rendell, A. P., Burant, J. C., Iyengar, S. S., Tomasi, J., Cossi, M., Millam, J. M., Klene, M., Adamo, C., Cammi, R., Ochterski, J. W., Martin, R. L., Morokuma, K., Farkas, O., Foresman, J. B., Fox, D. J. Gaussian 16, Rev. A.03, Wallingford, CT, **2016**.

[7] Adamo, C.; Barone, V. "Toward Reliable Density Functional Methods without Adjustable Parameters: The PBE0 Model." *J. Chem. Phys.* **1999**, *110*, 6158–69.

[8] Grimme, S.; Antony, J.; Ehrlich, S.; Krieg, H. A. "Consistent and Accurate Ab Initio Parametrization of Density Func-tional Dispersion Correction (DFT-D) for the 94 Elements H-Pu." *J. Chem. Phys.* **2010**, *132*, 154104.

[9] Weigend, F.; Ahlrichs, R. "Balanced Basis Sets of Split Valence, Triple Zeta Valence, and Quadruple Zeta Valence Quality for H to Rn: Design and Assessment of Accuracy." *Phys. Chem. Chem. Phys.* **2005**, *7 (18)*, 3297-3305.

[10] Marenich, A. V.; Cramer, C. J.; Truhlar, D. G. "Universal Solvation Model Based on Solute Electron Density and on a Continuum Model of the Solvent Defined by the Bulk Dielectric Constant and Atomic Surface Tensions." *J. Phys. Chem. B* **2009**, *113 (18)*, 6378-6396.

[11] Luchini, G.; Alegre-Requena, J. V.; Funes-Ardoiz, I.; Paton, R. S. "GoodVibes: Automated Thermochemistry for Heterogeneous Computational Chemistry Data." *F1000Research* **2020**, 9, 291.

[12] Legault, C. Y. "CYLview, 1.0b." Université de Sherbrooke: Quebec, Montreal, Canada, **2009**.

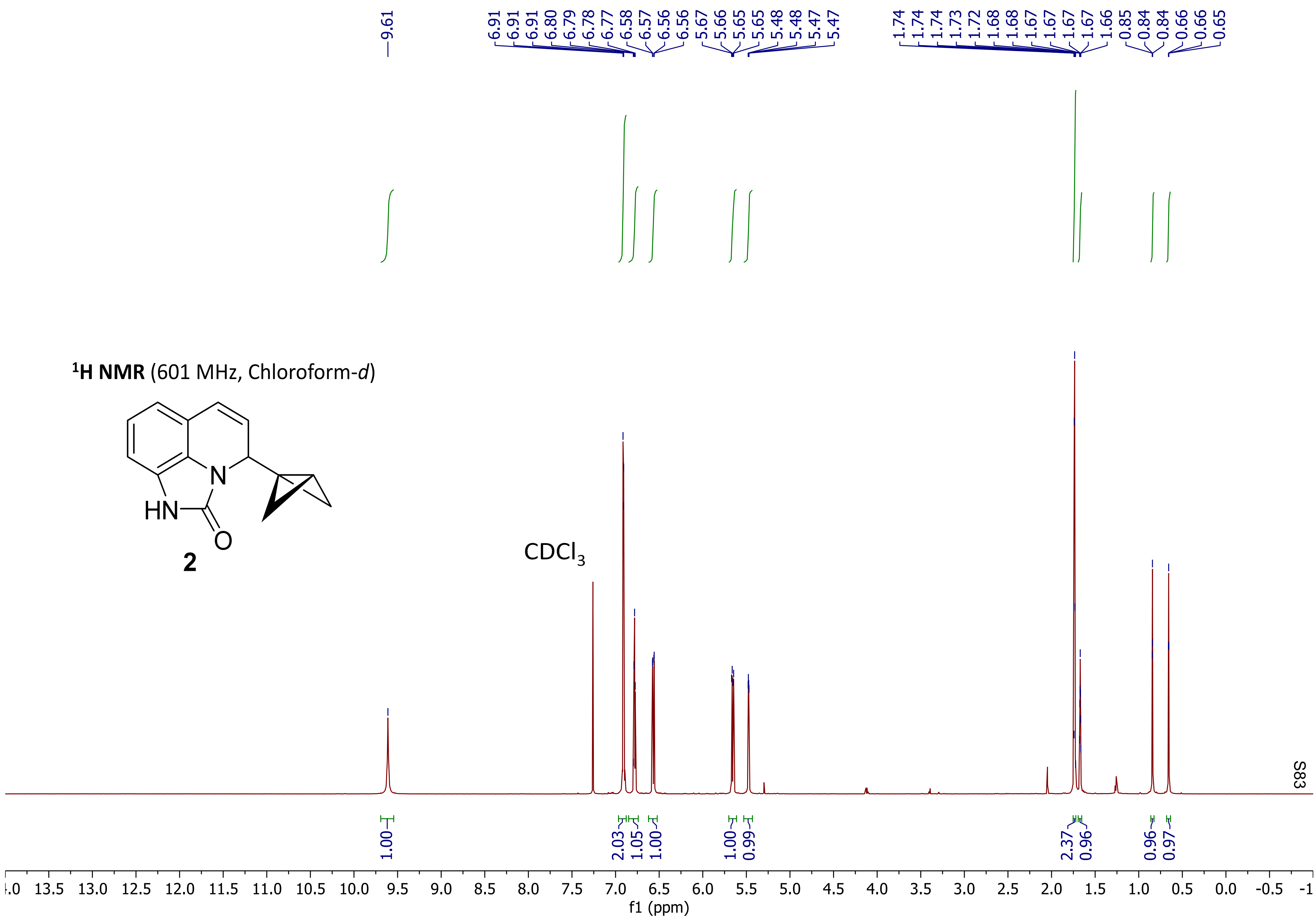

¹H NMR (601 MHz, Chloroform-d)
HN
N
O
2
CDCl3
9.61
6.91 6.91 6.91 6.80 6.79 6.78 6.77 6.58 6.57 6.56 6.56 5.67 5.66 5.65 5.65 5.48 5.48 5.47 5.47
1.74 1.74 1.74 1.73 1.72 1.68 1.68 1.67 1.67 1.67 1.67 1.66 0.85 0.84 0.84 0.66 0.66 0.65
1.00 2.03 1.05 1.00 1.00 0.99 2.37 0.96 0.96 0.97
f1 (ppm)

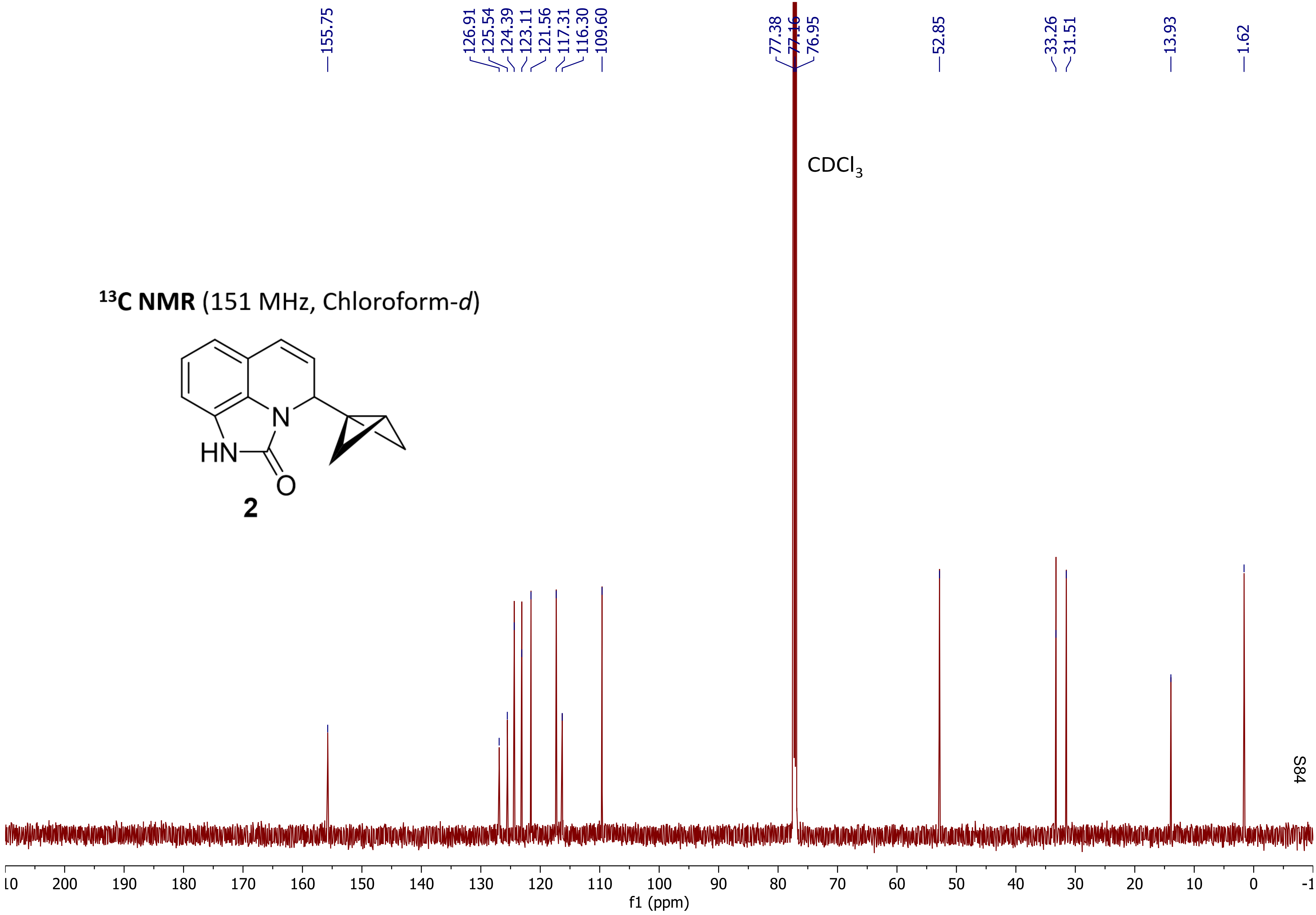

13C NMR (151 MHz, Chloroform-d)
HN
N
O
2
CDCl3
155.75
126.91
125.54
124.39
123.11
121.56
117.31
116.30
109.60
77.38
77.16
76.95
52.85
33.26
31.51
13.93
1.62
f1 (ppm)

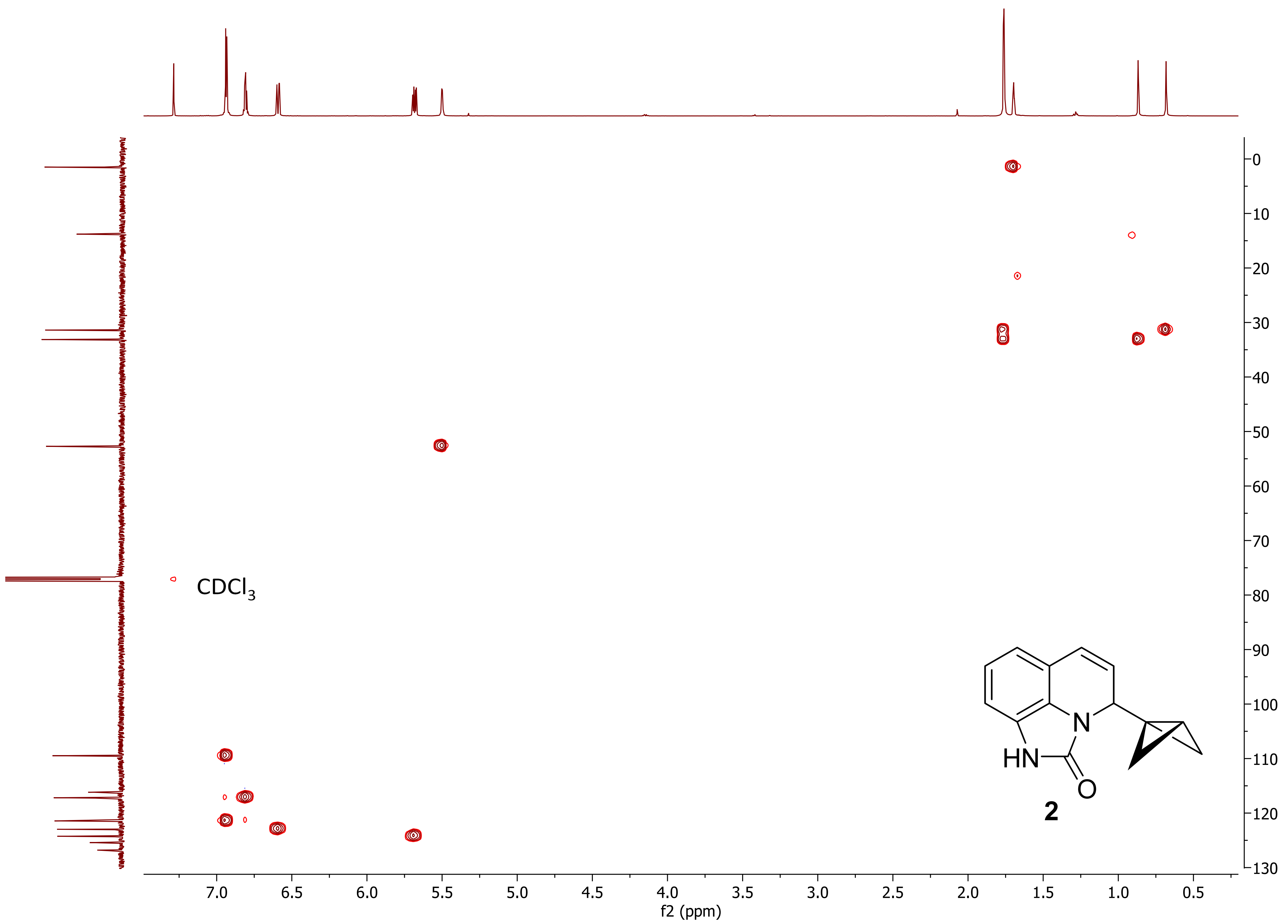

CDCl3
HN
N
O
2
f2 (ppm)
f1 (ppm)

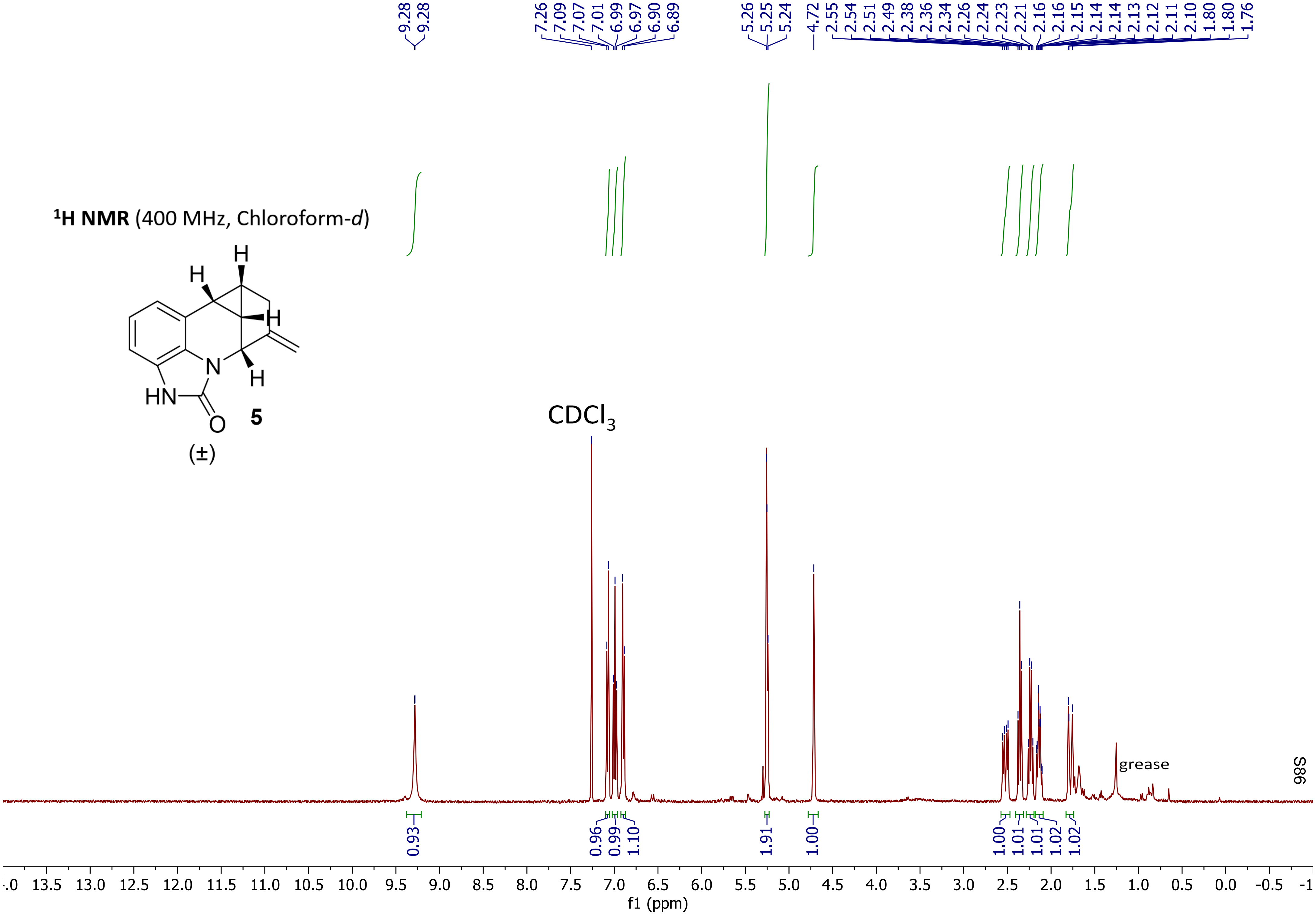

¹H NMR (400 MHz, Chloroform-d)
5
(±)
HN
N
O
H
CDCl3
grease
9.28 9.28
7.26 7.09 7.07 7.01 6.99 6.97 6.90 6.89
5.26 5.25 5.24
4.72
2.55 2.54 2.51 2.49 2.38 2.36 2.34 2.26 2.24 2.23 2.21 2.16 2.16 2.15 2.14 2.14 2.13 2.12 2.11 2.10 1.80 1.80 1.76
0.93
0.96 0.99 1.10
1.91
1.00
1.00 1.01 1.01 1.02 1.02
f1 (ppm)

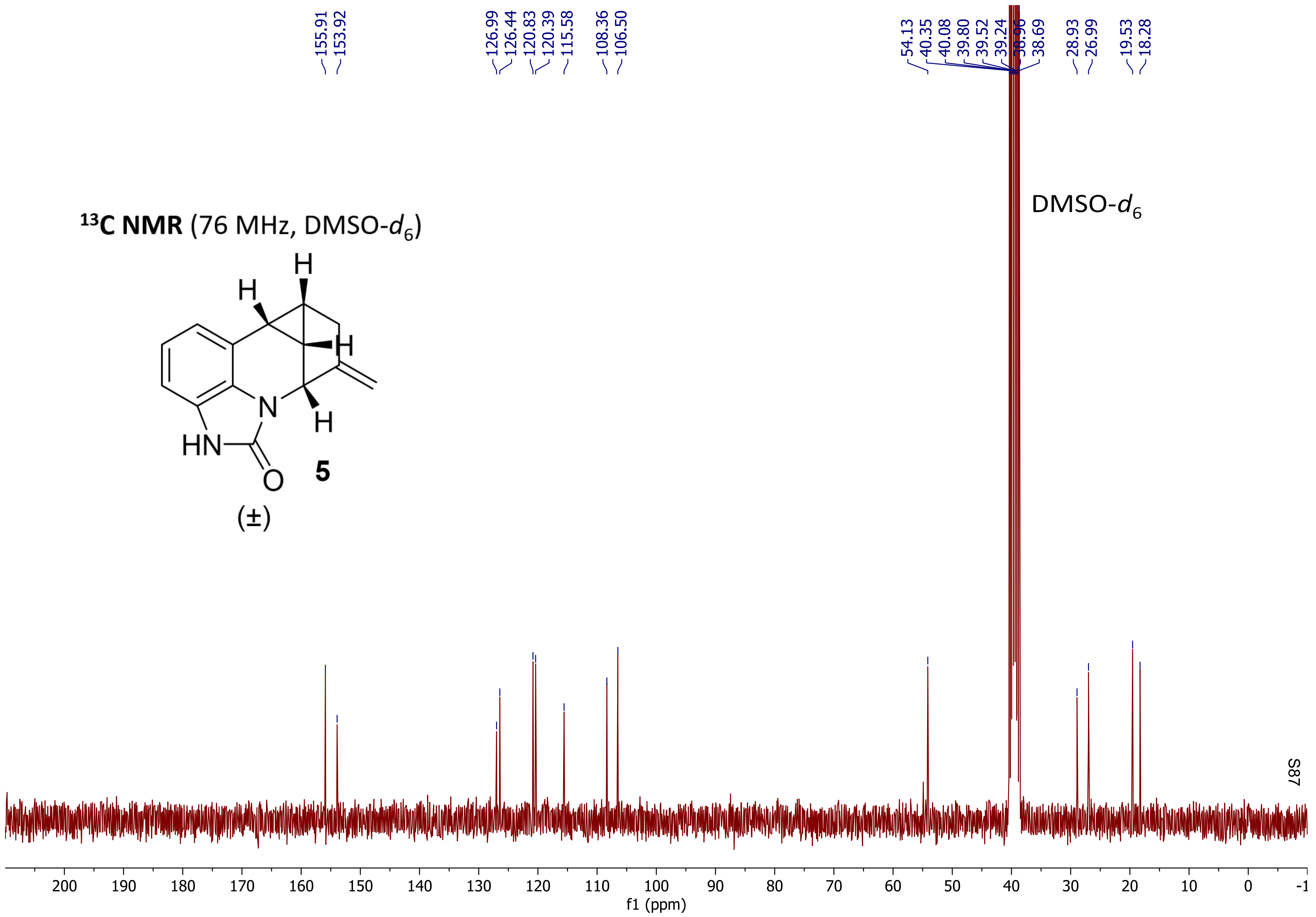

^{13}C NMR (76 MHz, DMSO-d_6)
5
(±)
DMSO-d_6
155.91
153.92
126.99
126.44
120.83
120.39
115.58
108.36
106.50
54.13
40.35
40.08
39.80
39.52
39.24
38.96
38.69
28.93
26.99
19.53
18.28
f1 (ppm)

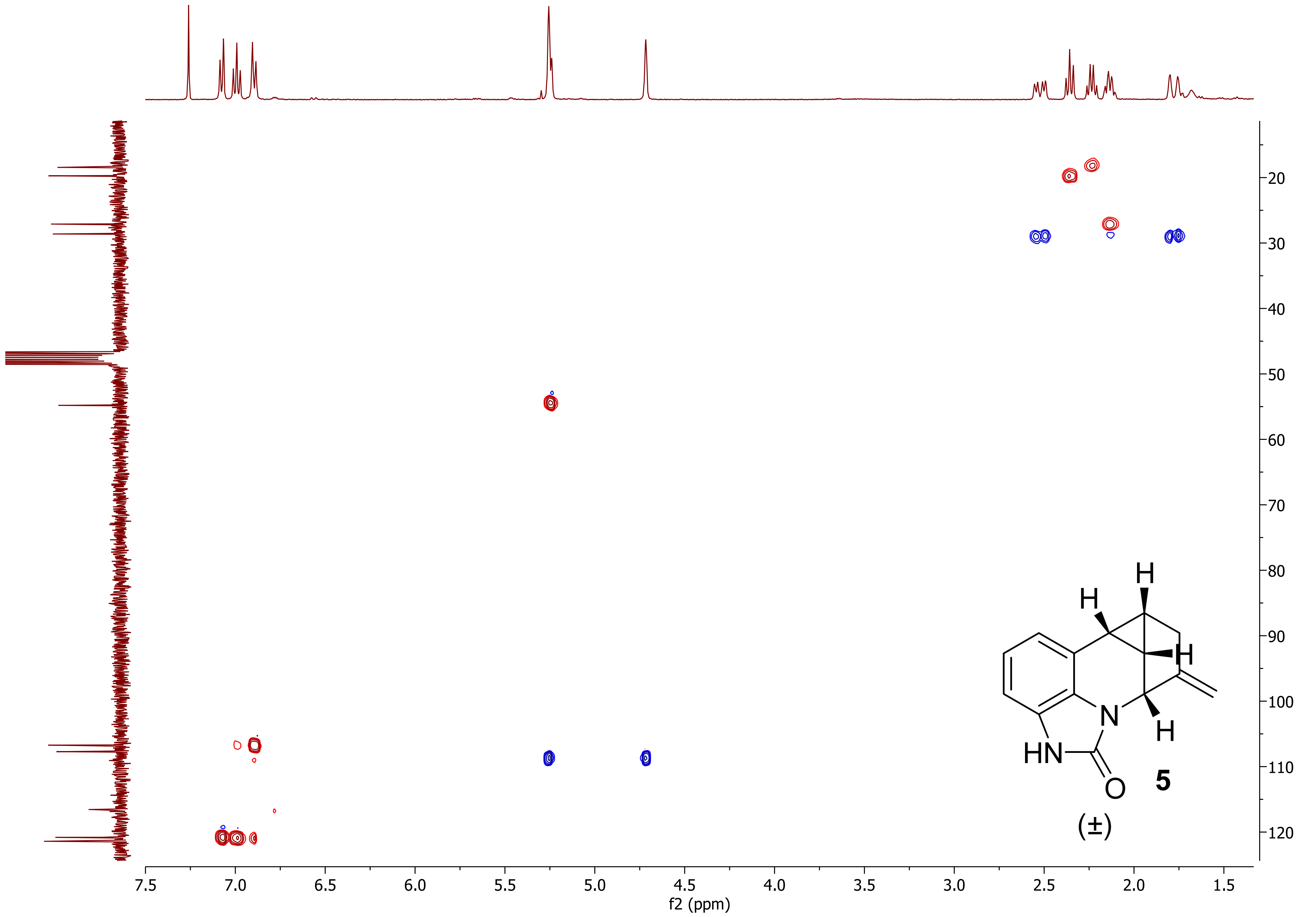

7.5
7.0
6.5
6.0
5.5
5.0
4.5
4.0
3.5
3.0
2.5
2.0
1.5
f2 (ppm)
20
30
40
50
60
70
80
90
100
110
120
f1 (ppm)
H
H
H
H
N
HN
O
5
(±)

1H NMR (601 MHz, Chloroform-*d*)

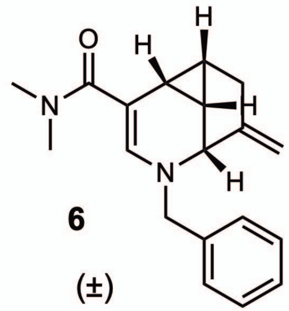

6

(±)

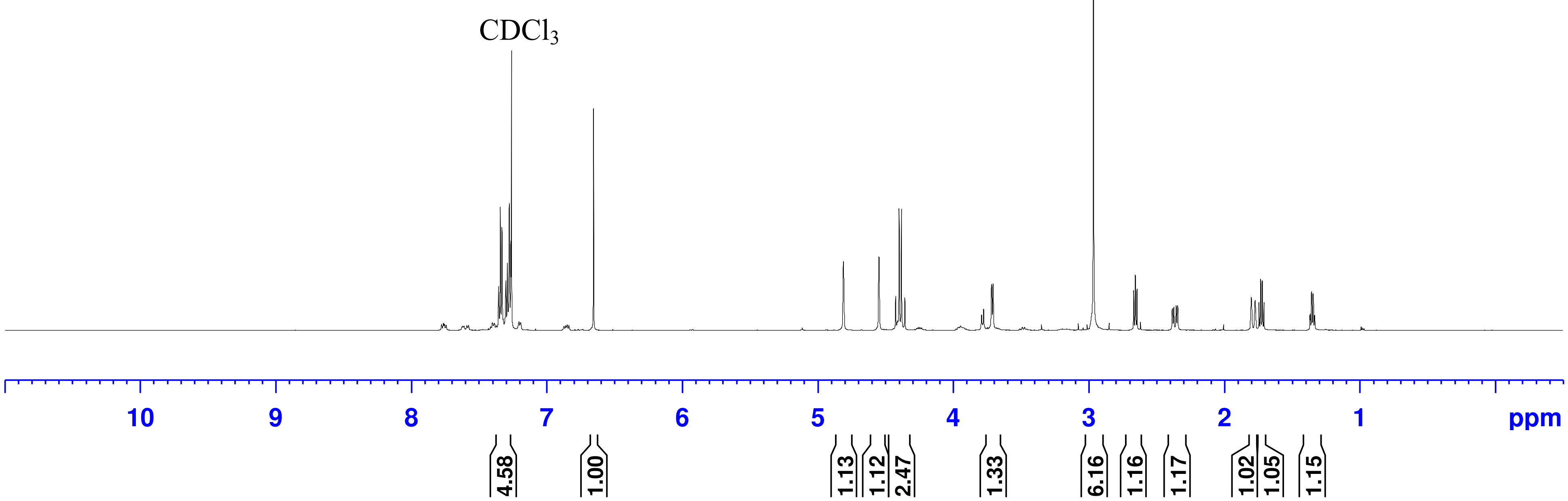

^{13}C **NMR** (151 MHz, Chloroform-*d*)

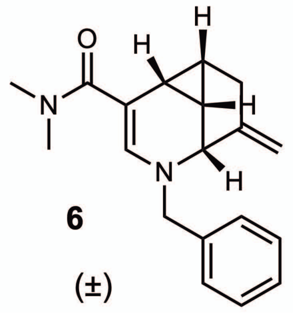

6

(±)

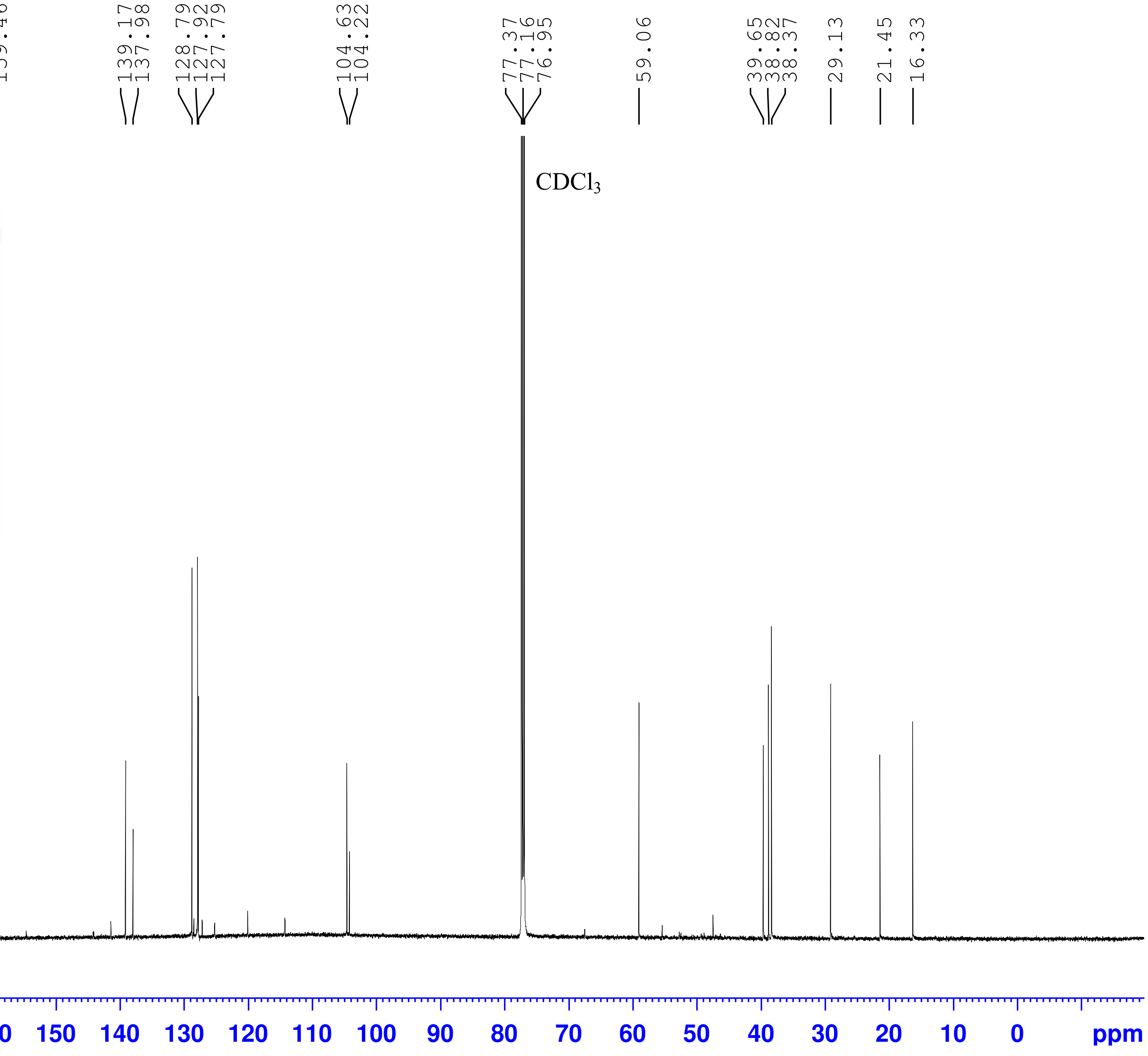

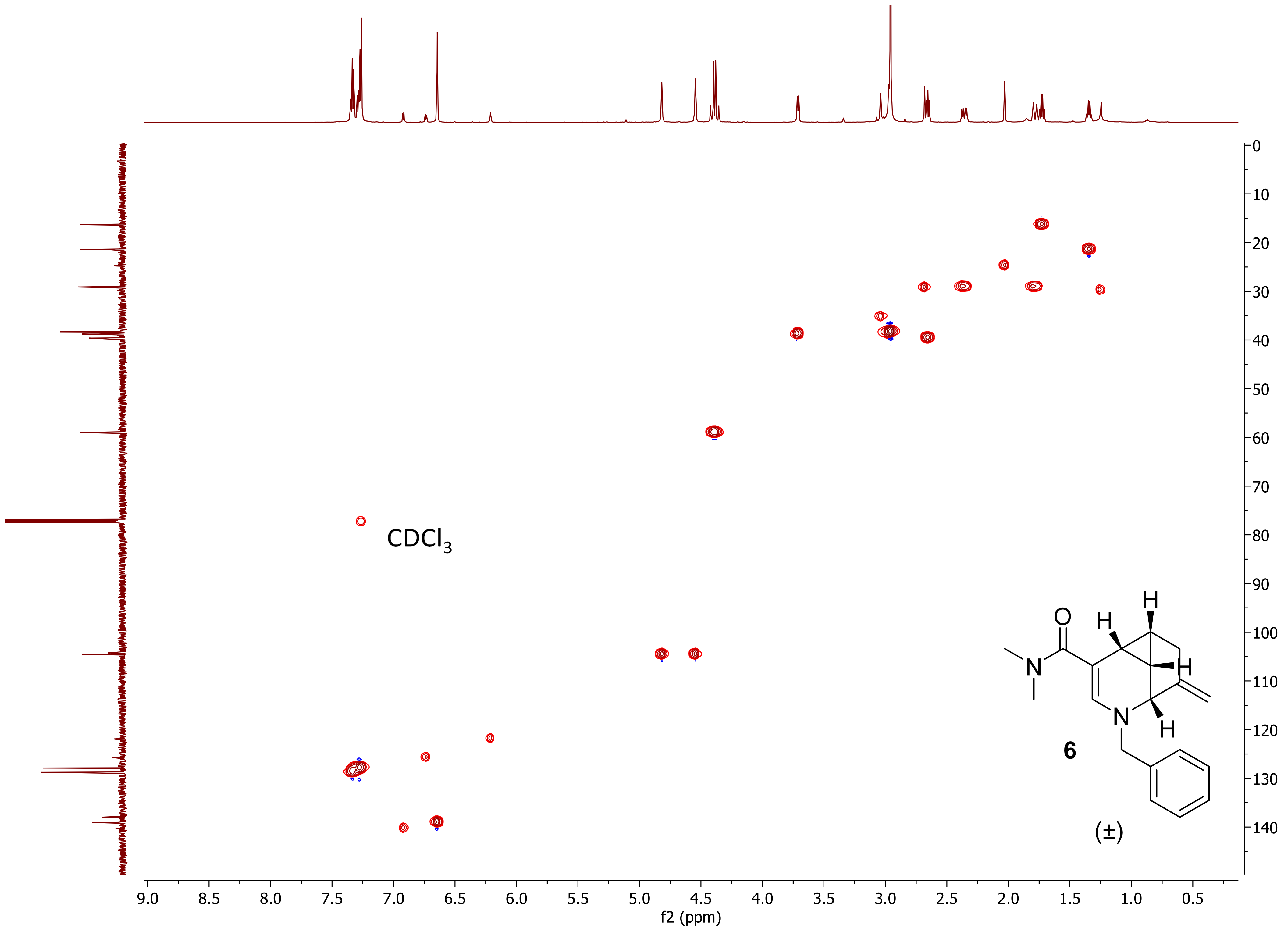
CDCl3
6
(±)
f1 (ppm)
f2 (ppm)

¹H NMR (500 MHz, Chloroform-*d*)

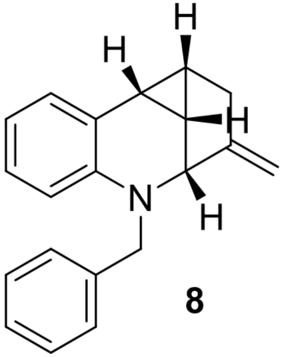

8

(±)

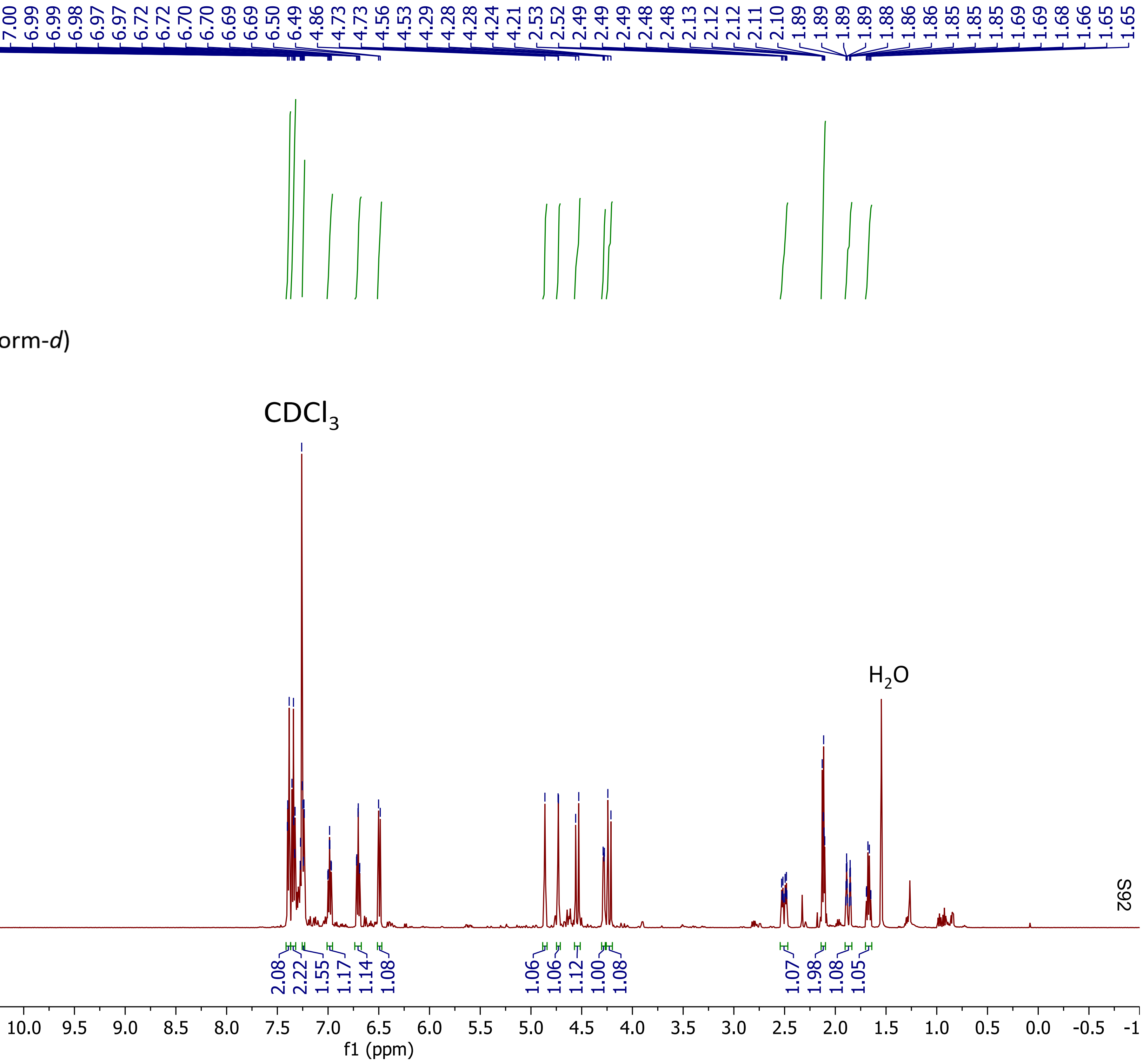

^{13}C NMR (126 MHz, Chloroform-*d*)

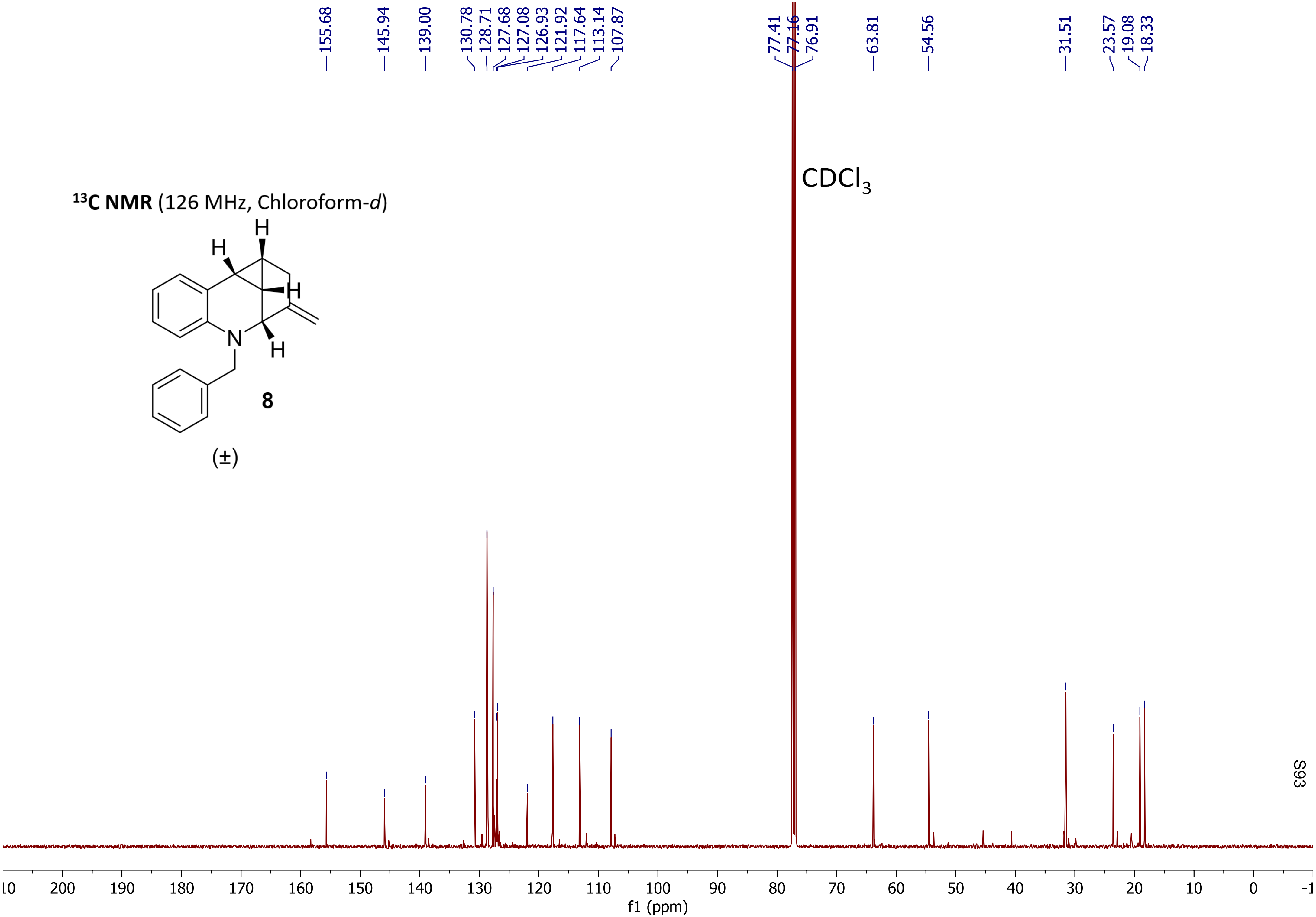

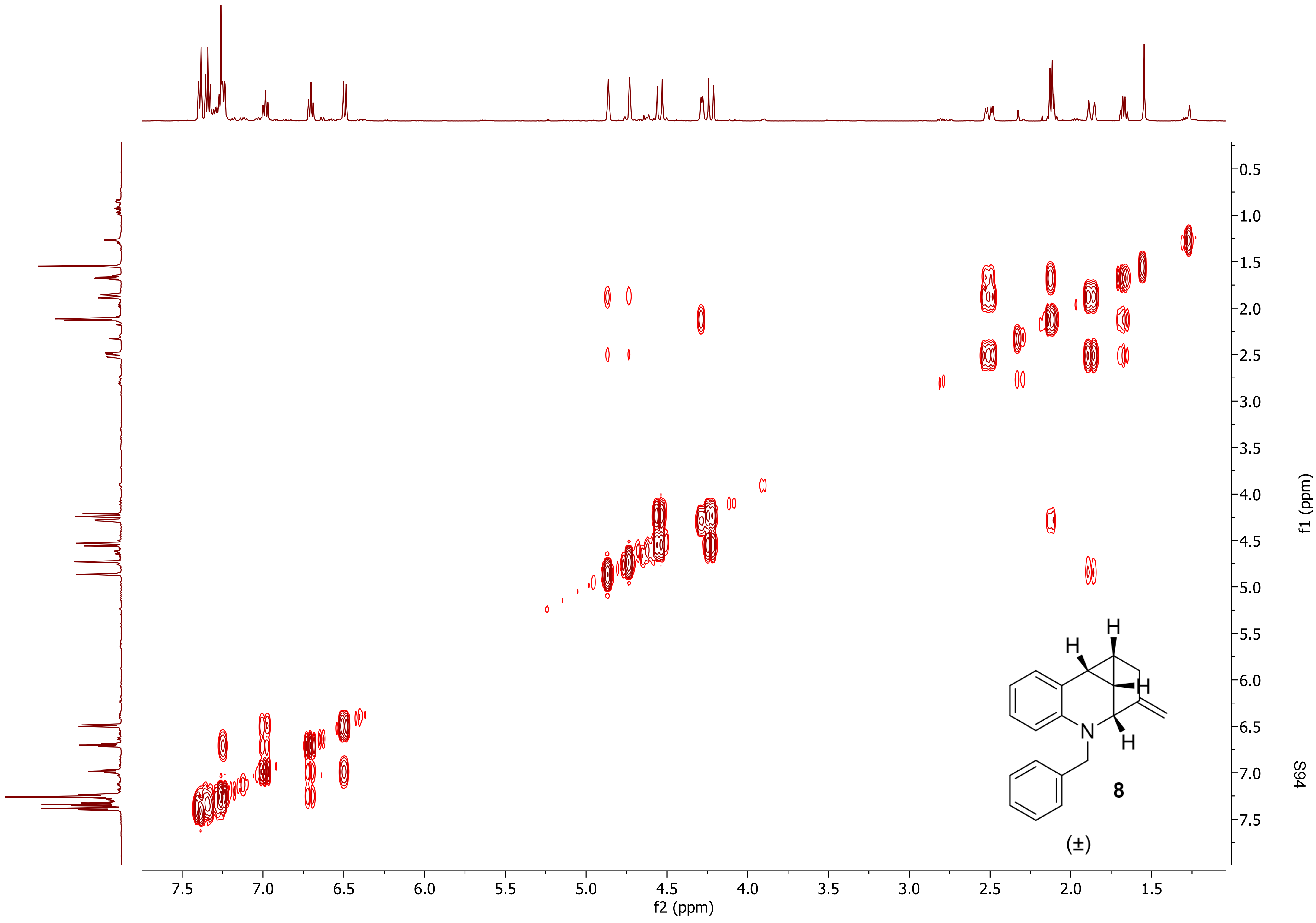

f2 (ppm)
f1 (ppm)
7.5
7.0
6.5
6.0
5.5
5.0
4.5
4.0
3.5
3.0
2.5
2.0
1.5
1.0
0.5
H
N
8
(±)

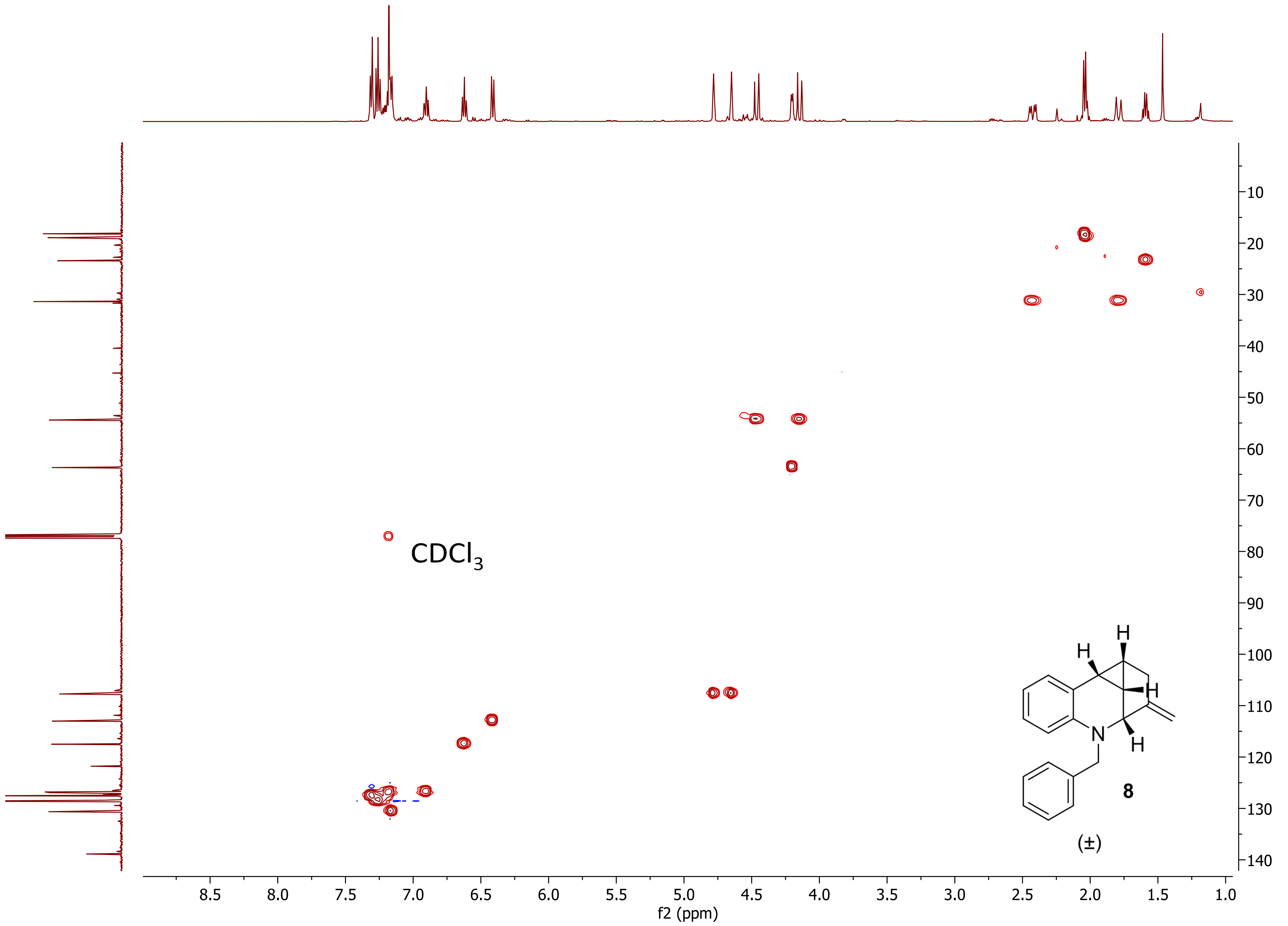
CDCl$_3$
8
(±)
f2 (ppm)
f1 (ppm)

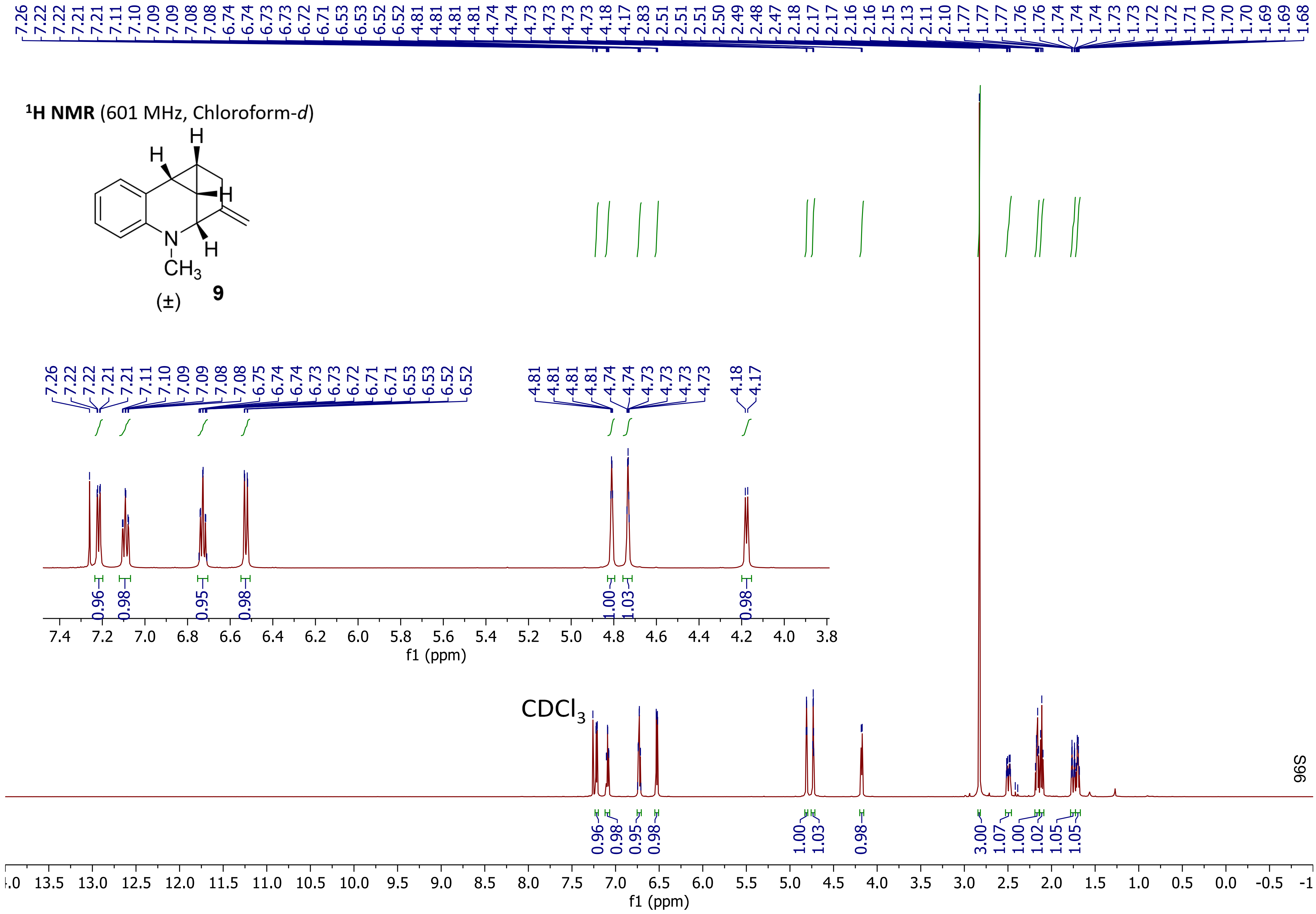
7.26 7.22 7.22 7.21 7.21 7.11 7.10 7.09 7.09 7.08 7.08 6.74 6.74 6.73 6.73 6.72 6.71 6.53 6.53 6.52 6.52 4.81 4.81 4.81 4.81 4.74 4.74 4.73 4.73 4.73 4.73 4.18 4.17 2.83 2.51 2.51 2.51 2.50 2.49 2.48 2.47 2.18 2.17 2.17 2.16 2.16 2.15 2.13 2.11 2.10 1.77 1.77 1.77 1.76 1.76 1.74 1.74 1.74 1.73 1.73 1.72 1.72 1.71 1.70 1.70 1.70 1.69 1.69 1.68
¹H NMR (601 MHz, Chloroform-*d*)
H
H
H
H
N
CH_3
(±)
9
7.26 7.22 7.22 7.21 7.21 7.11 7.10 7.09 7.09 7.08 7.08 6.75 6.74 6.74 6.73 6.73 6.72 6.71 6.71 6.53 6.53 6.52 6.52
4.81 4.81 4.81 4.81 4.74 4.74 4.73 4.73 4.73 4.73
4.18 4.17
0.96 0.98 0.95 0.98 1.00 1.03 0.98
7.4 7.2 7.0 6.8 6.6 6.4 6.2 6.0 5.8 5.6 5.4 5.2 5.0 4.8 4.6 4.4 4.2 4.0 3.8
f1 (ppm)
$CDCl_3$
0.96 0.98 0.95 0.98 1.00 1.03 0.98 3.00 1.07 1.00 1.02 1.05 1.05
14.0 13.5 13.0 12.5 12.0 11.5 11.0 10.5 10.0 9.5 9.0 8.5 8.0 7.5 7.0 6.5 6.0 5.5 5.0 4.5 4.0 3.5 3.0 2.5 2.0 1.5 1.0 0.5 0.0 -0.5 -1
f1 (ppm)

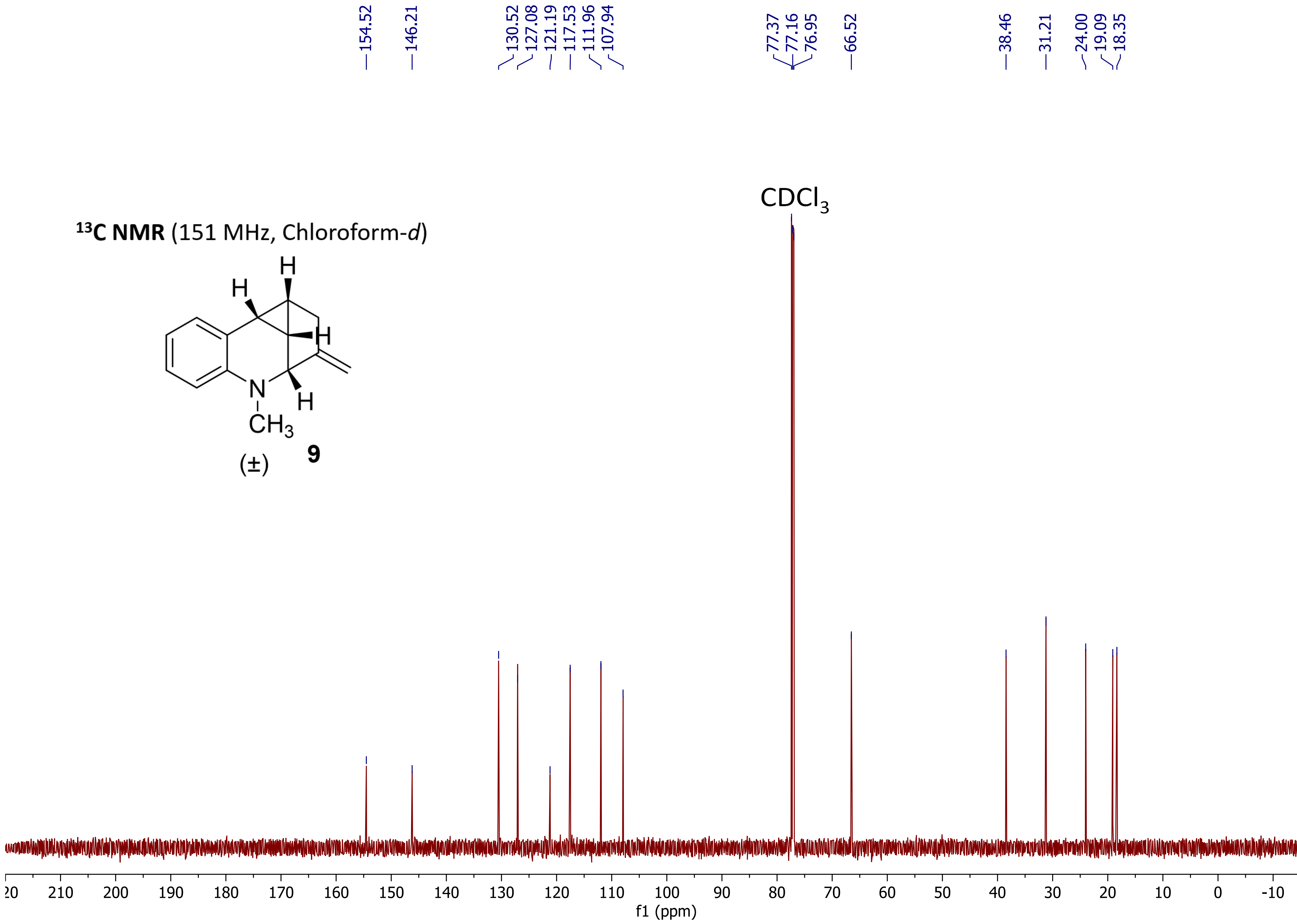
154.52
146.21
130.52
127.08
121.19
117.53
111.96
107.94
77.37
77.16
76.95
66.52
38.46
31.21
24.00
19.09
18.35
CDCl$_3$
^{13}C NMR (151 MHz, Chloroform-d)
H
H
H
H
N
CH$_3$
(±)
9
f1 (ppm)
20 210 200 190 180 170 160 150 140 130 120 110 100 90 80 70 60 50 40 30 20 10 0 -10

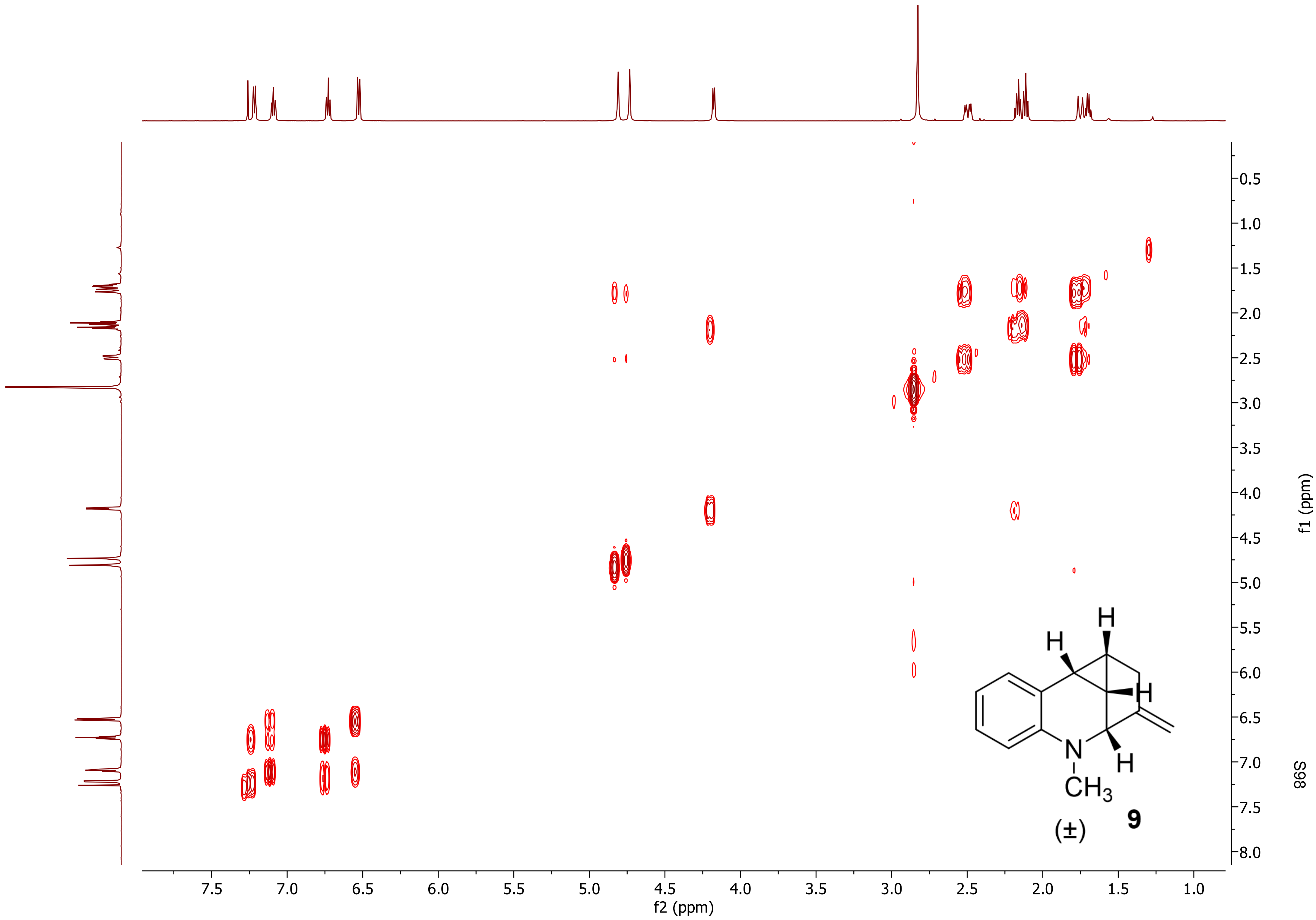

f2 (ppm)
f1 (ppm)
N
CH3
H
(±)
9

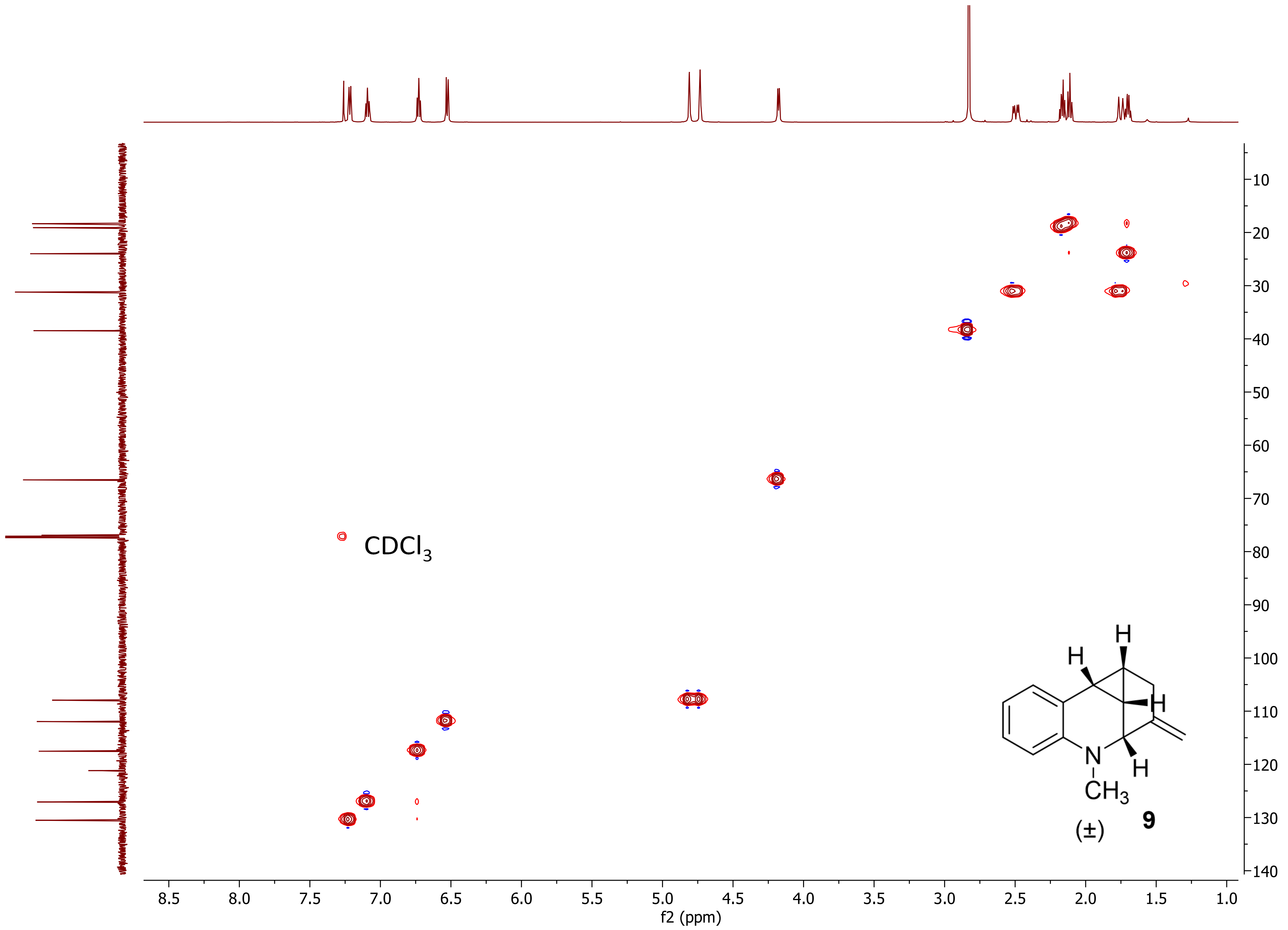
CDCl3
f2 (ppm)
f1 (ppm)
(±)
9

^{1}H NMR (500 MHz, Chloroform-*d*)

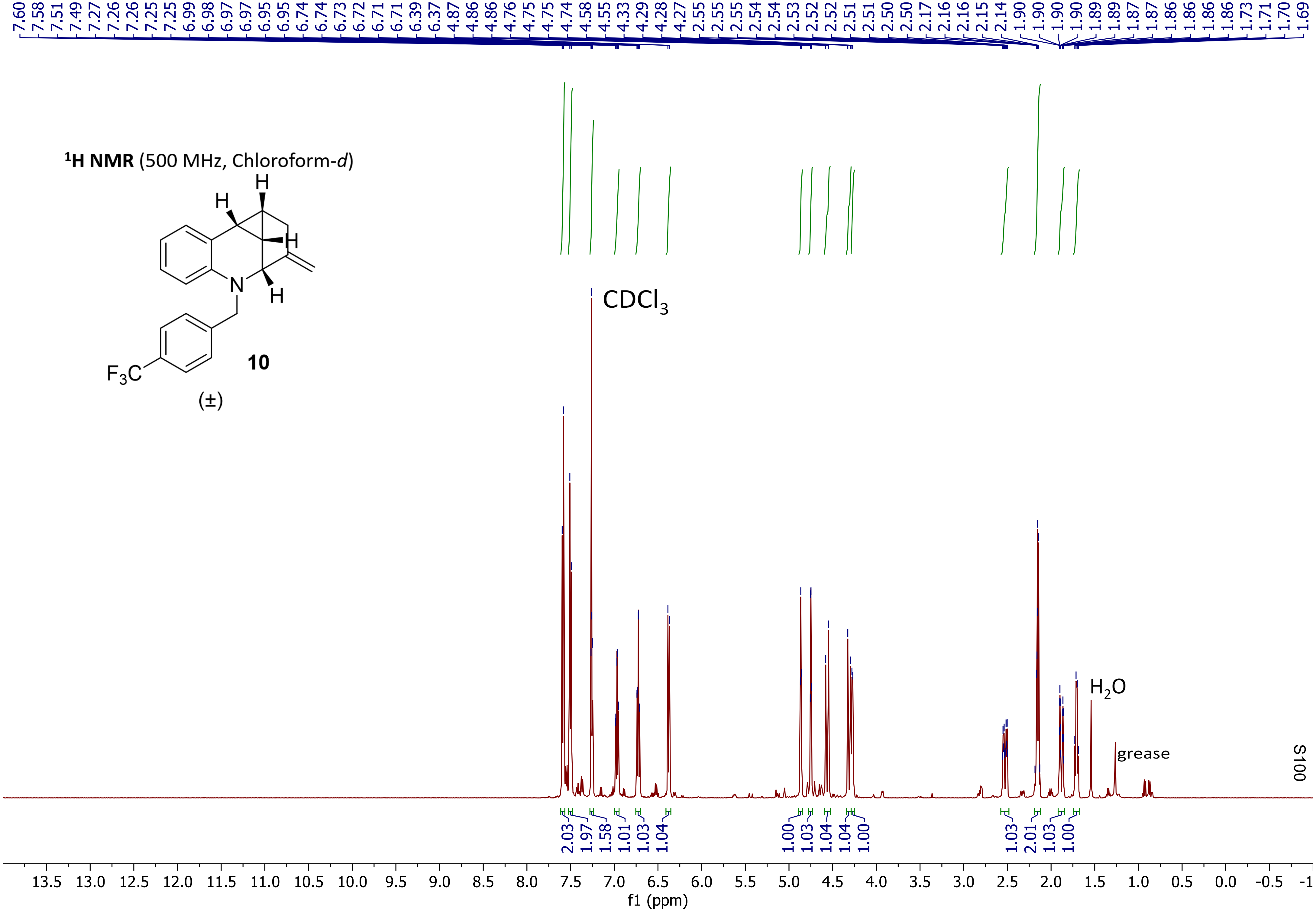

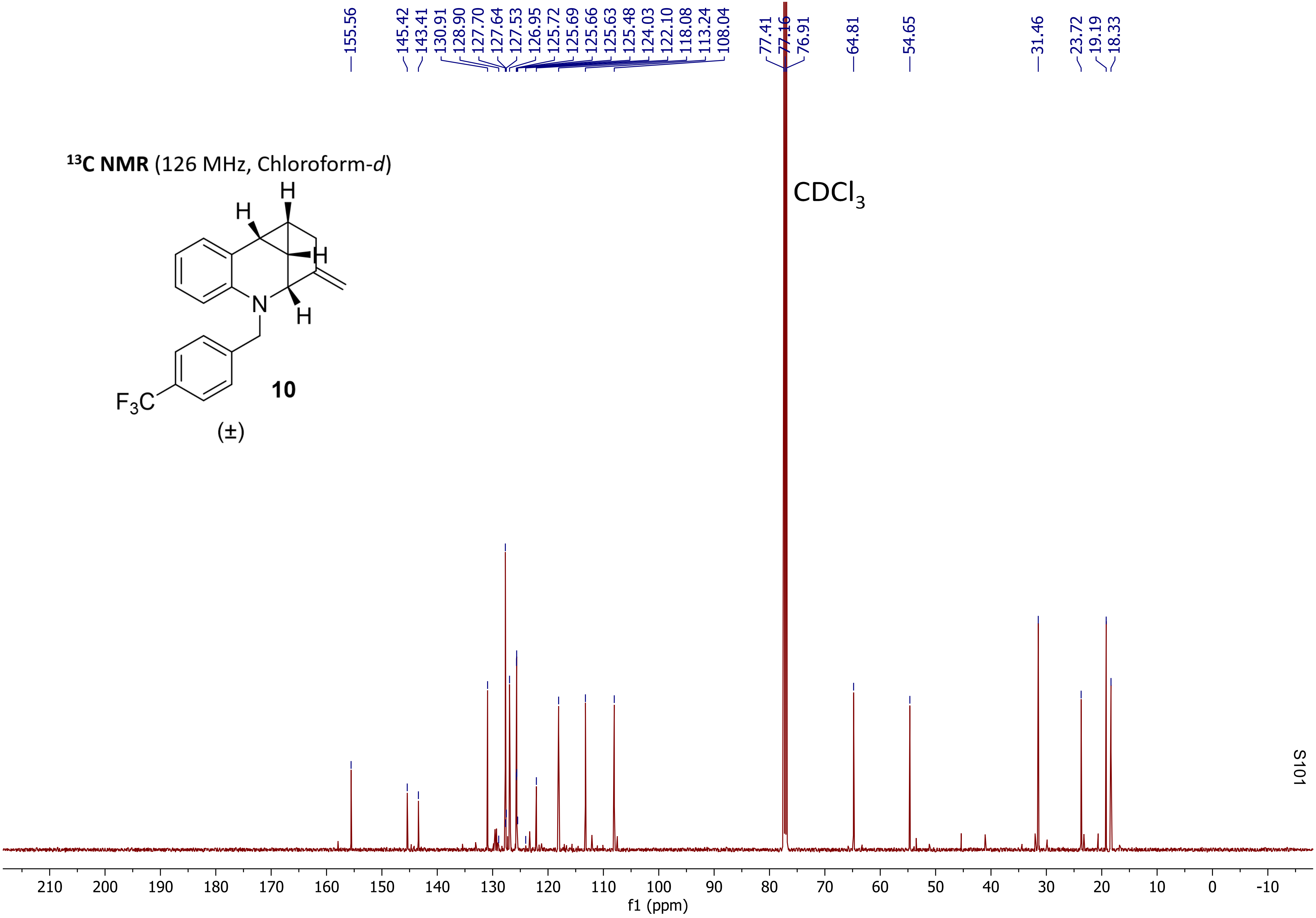

155.56
145.42
143.41
130.91
128.90
127.70
127.64
127.53
126.95
125.72
125.69
125.66
125.63
125.48
124.03
122.10
118.08
113.24
108.04
77.41
77.16
76.91
64.81
54.65
31.46
23.72
19.19
18.33
^{13}C NMR (126 MHz, Chloroform-d)
H
H
H
H
N
F_3C
10
(±)
$CDCl_3$
210 200 190 180 170 160 150 140 130 120 110 100 90 80 70 60 50 40 30 20 10 0 -10
f1 (ppm)

19F NMR (471 MHz, Chloroform-*d*)

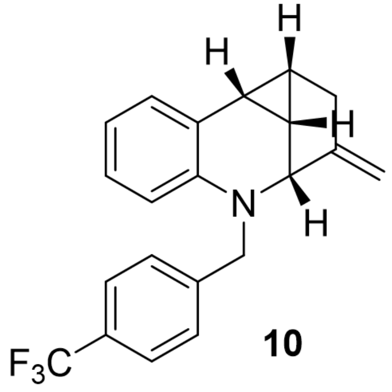

10

(±)

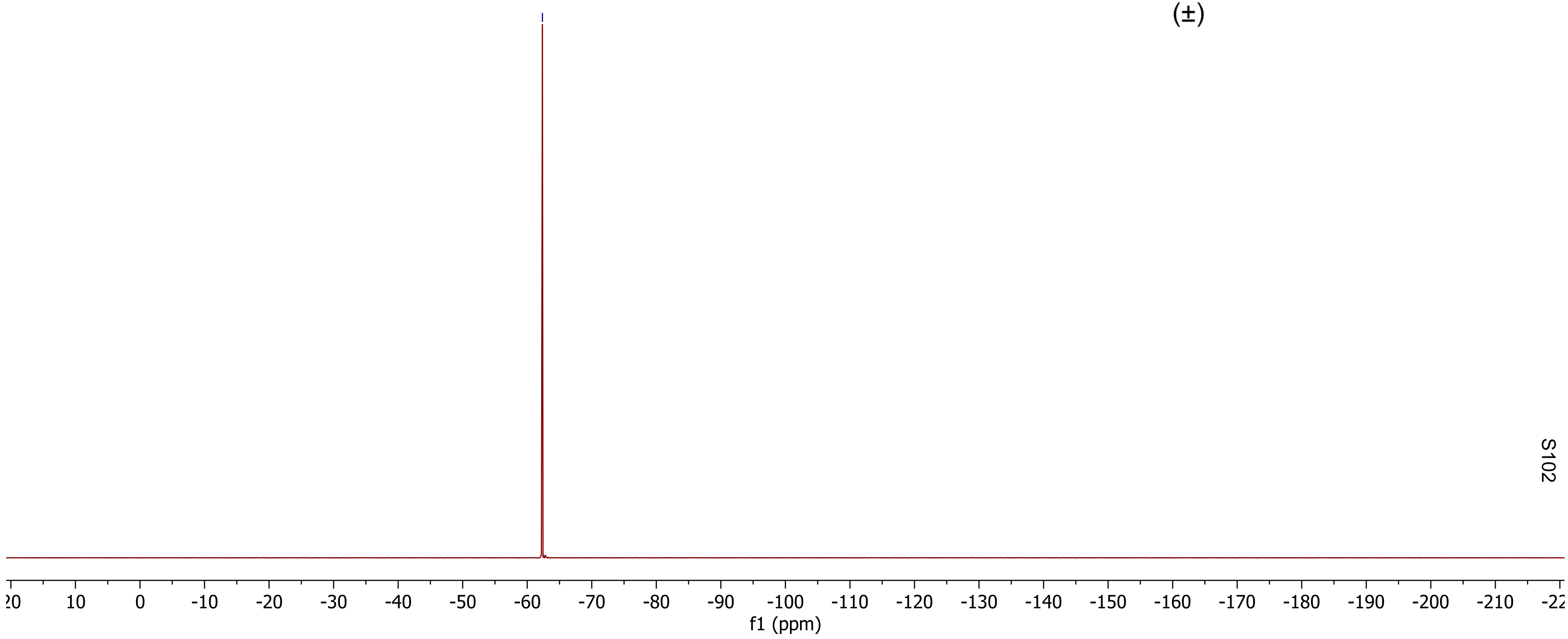

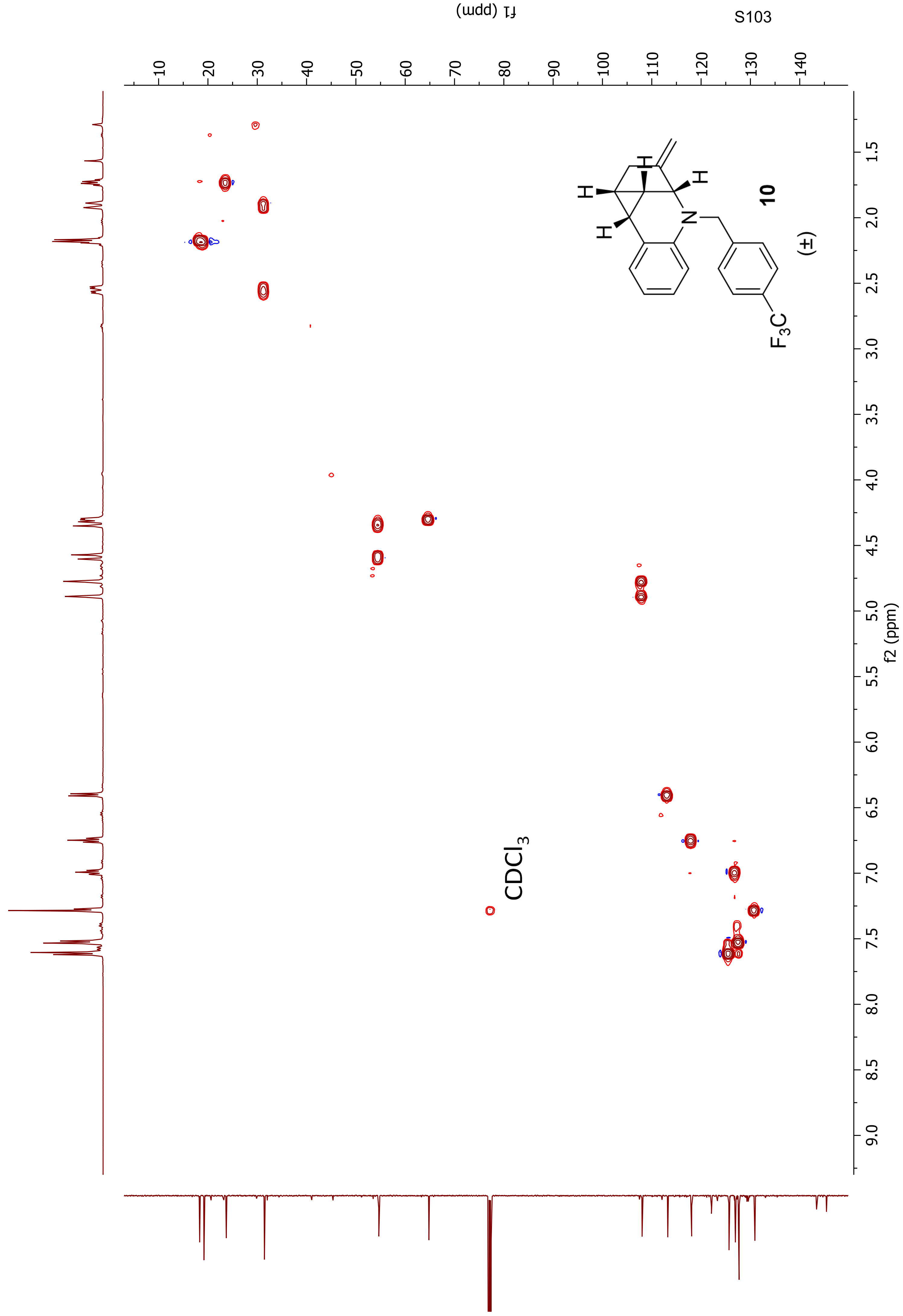
f1 (ppm)
f2 (ppm)
10
(±)
F3C
CDCl3

^{1}H NMR (300 MHz, Chloroform-*d*)

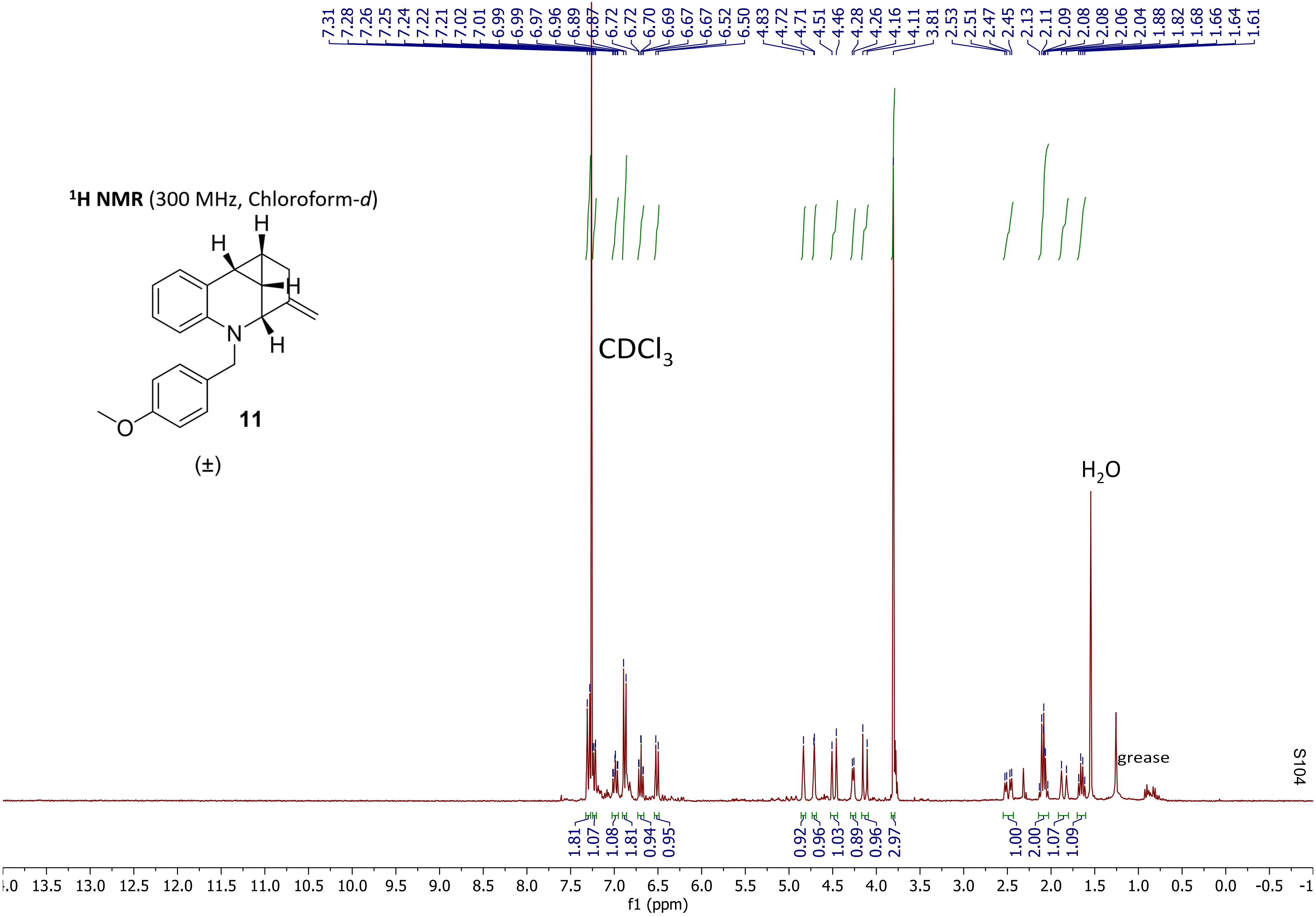

^{13}C NMR (126 MHz, Chloroform-*d*)

11

(±)

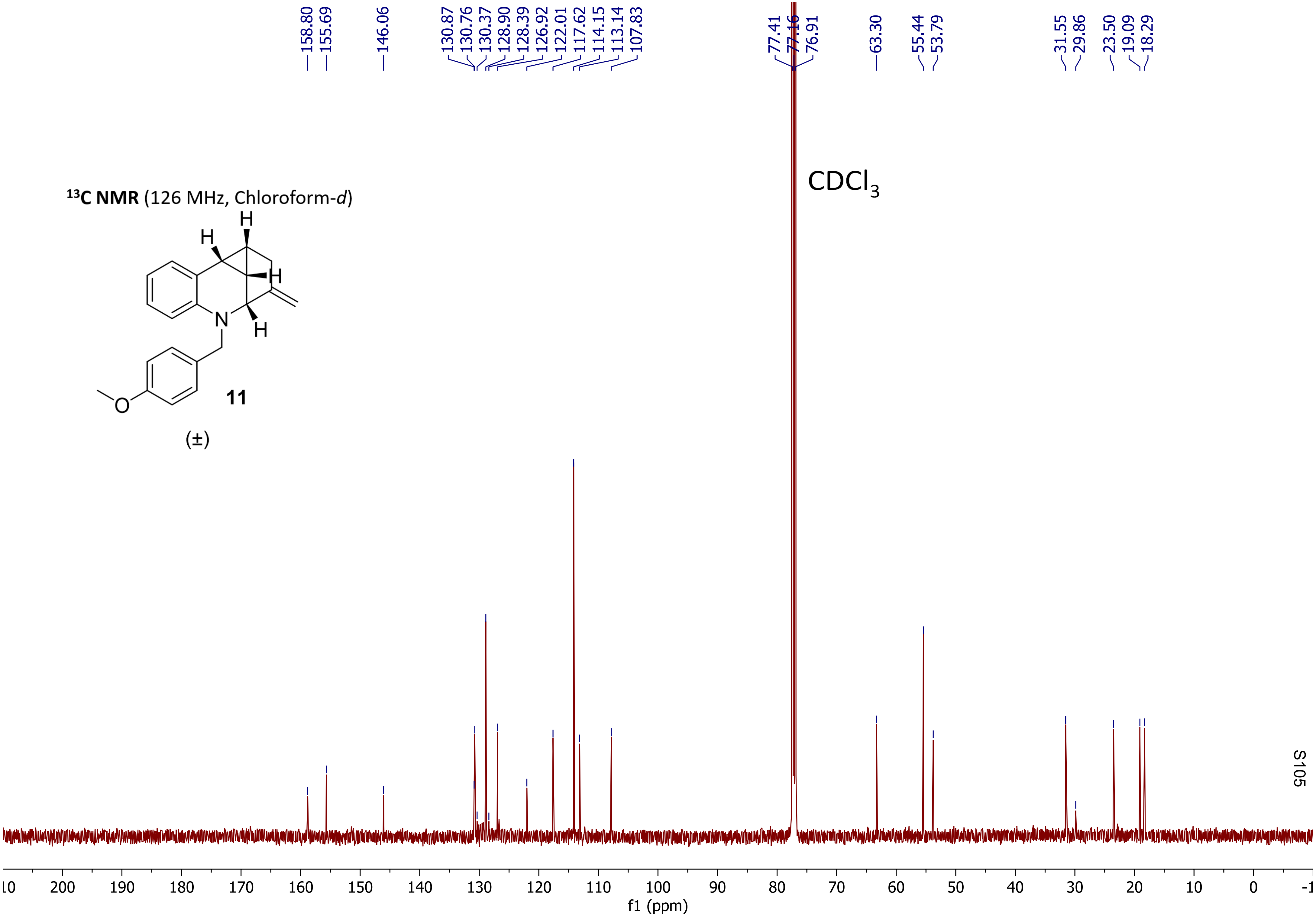

^{1}H NMR (601 MHz, Chloroform-*d*)

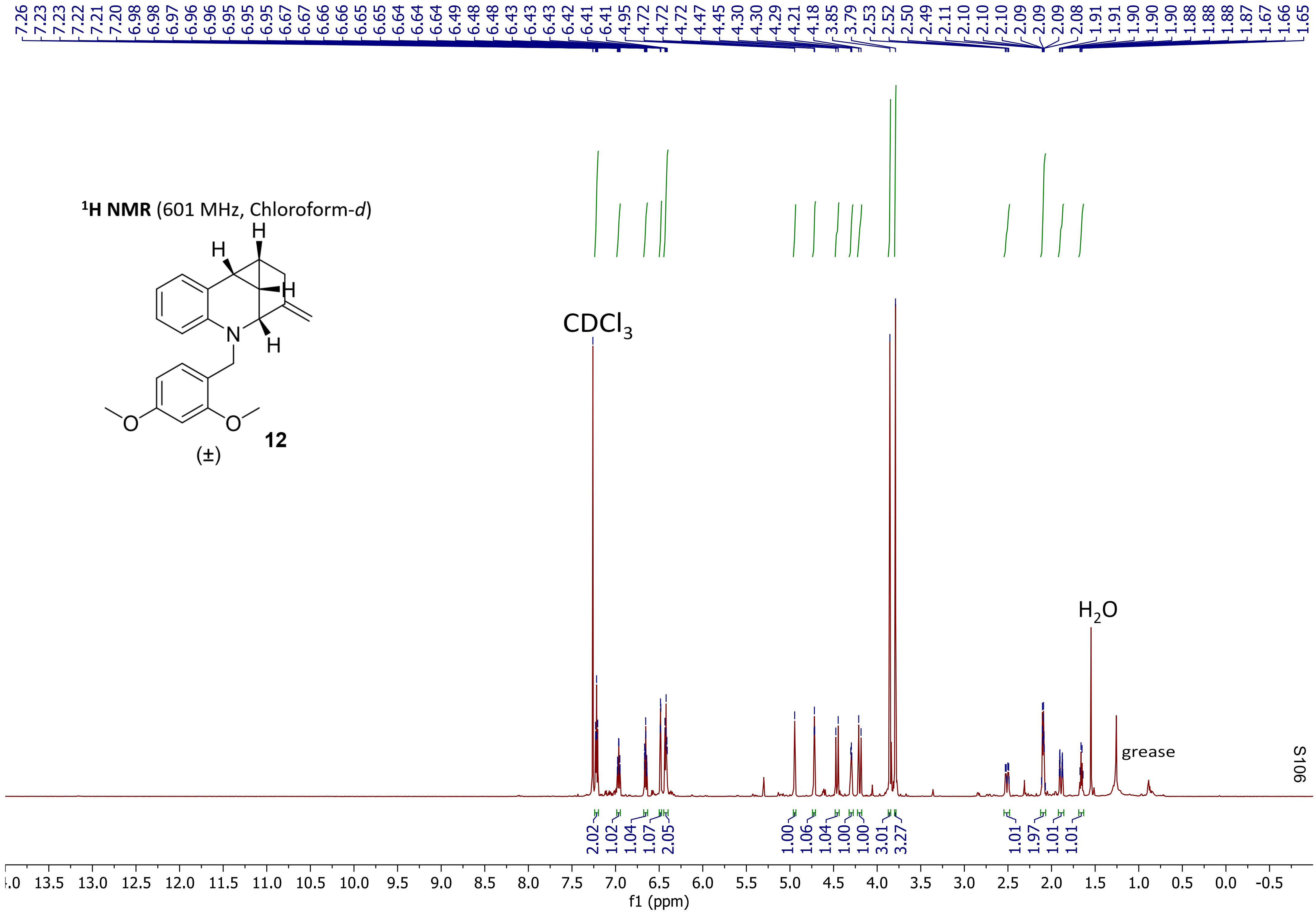

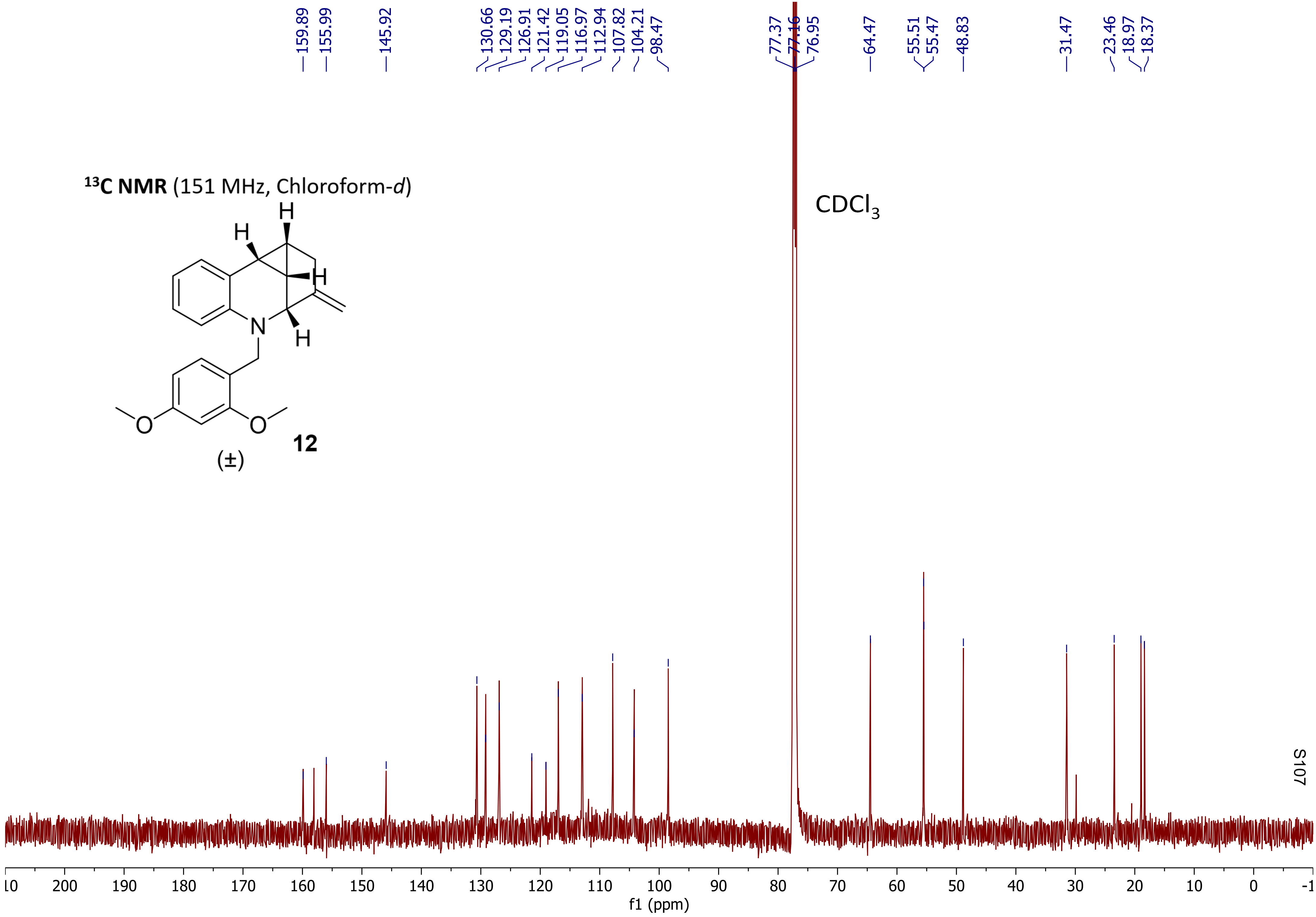
13C NMR (151 MHz, Chloroform-d)
12
(±)
CDCl3
159.89
155.99
145.92
130.66
129.19
126.91
121.42
119.05
116.97
112.94
107.82
104.21
98.47
77.37
77.16
76.95
64.47
55.51
55.47
48.83
31.47
23.46
18.97
18.37
f1 (ppm)

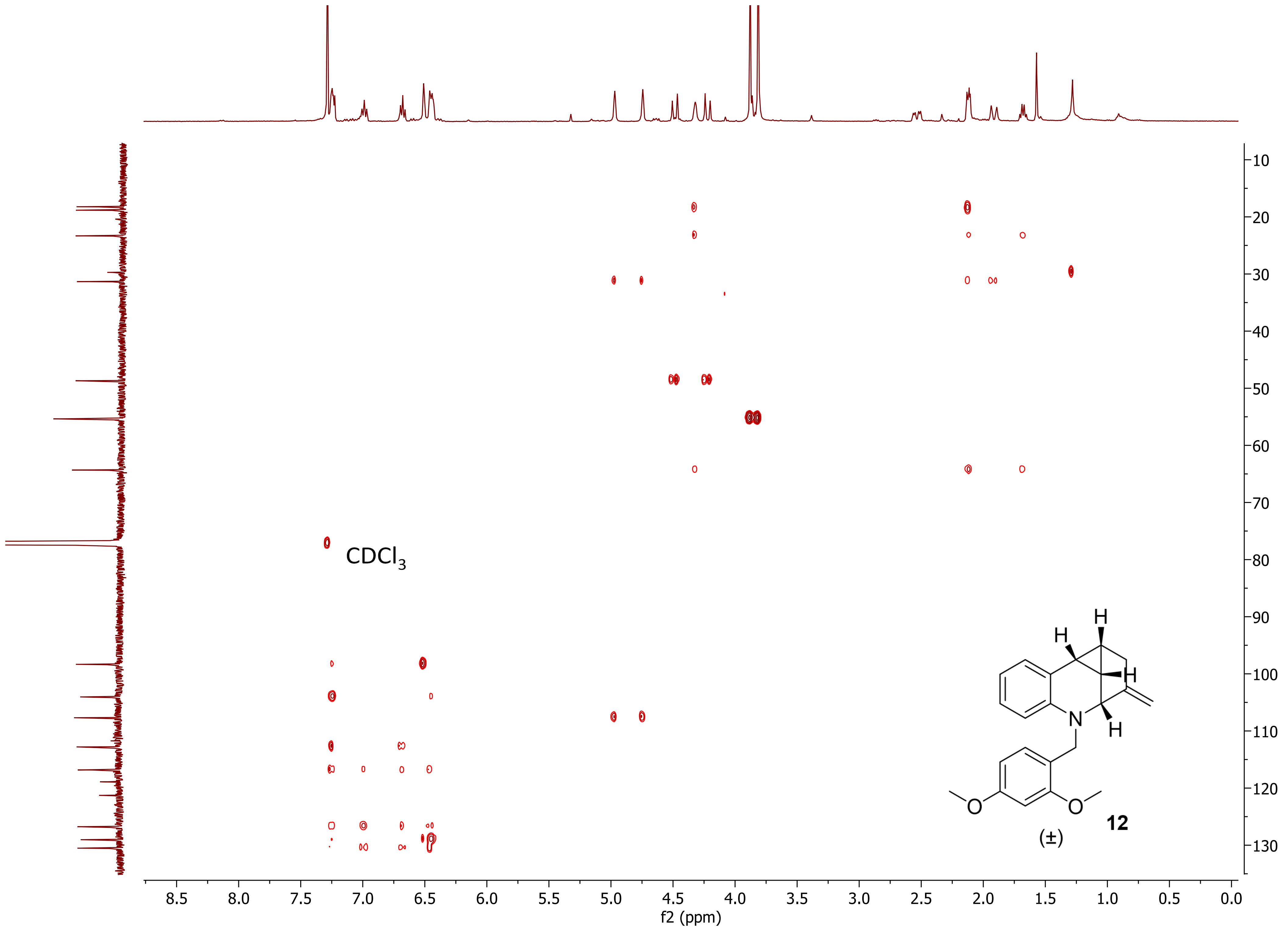

CDCl$_3$
12
(±)
f1 (ppm)
f2 (ppm)

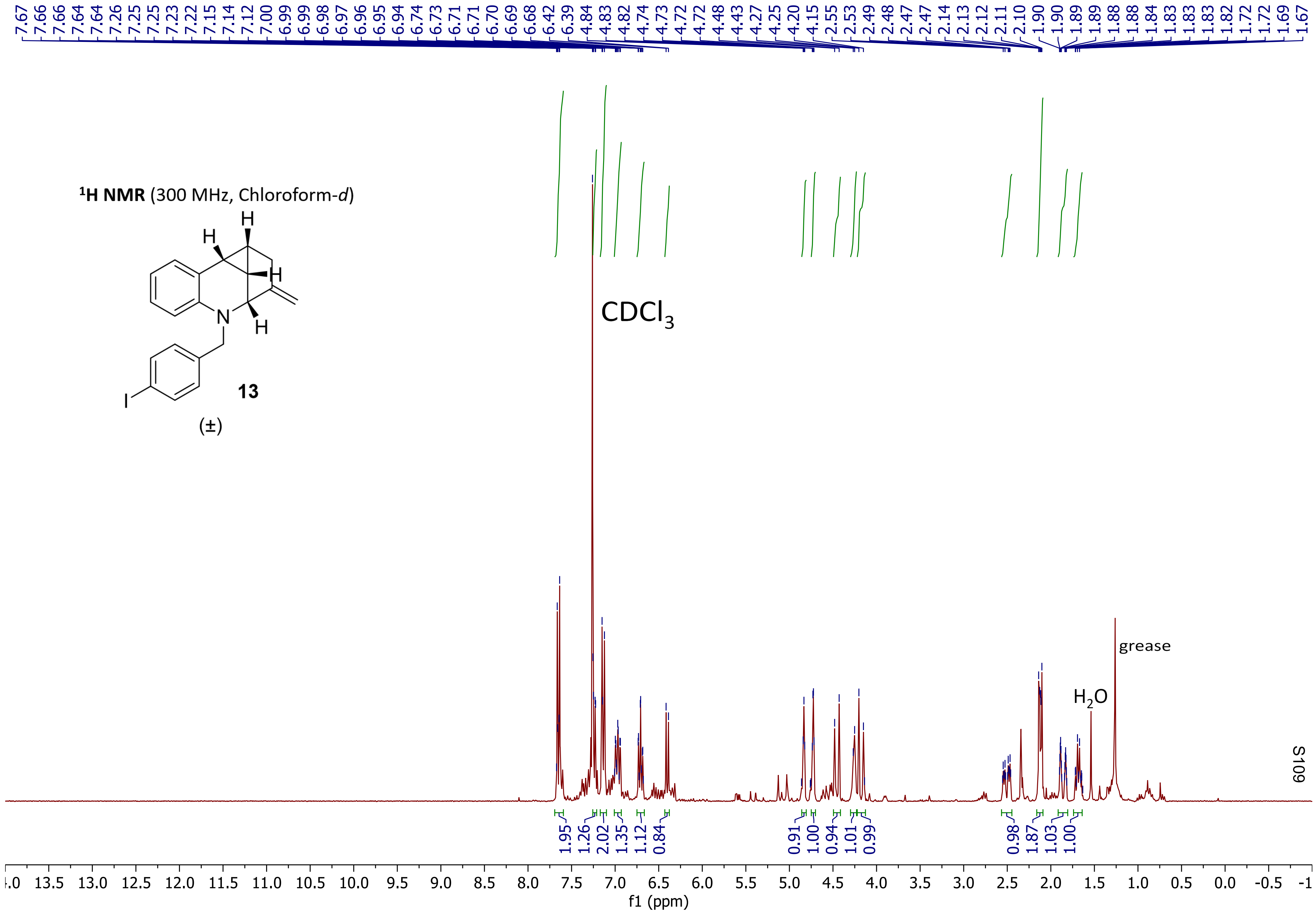

¹H NMR (300 MHz, Chloroform-d)
13
(±)
CDCl3
H2O
grease
7.67 7.66 7.66 7.64 7.64 7.26 7.25 7.25 7.23 7.22 7.15 7.14 7.12 7.00 6.99 6.99 6.98 6.97 6.96 6.95 6.94 6.74 6.73 6.71 6.71 6.70 6.69 6.68 6.42 6.39 4.84 4.83 4.82 4.74 4.73 4.72 4.72 4.48 4.43 4.27 4.25 4.20 4.15 2.55 2.53 2.49 2.48 2.47 2.47 2.14 2.13 2.12 2.11 2.10 1.90 1.90 1.89 1.89 1.88 1.88 1.84 1.83 1.83 1.83 1.82 1.72 1.72 1.69 1.67
1.95 1.26 2.02 1.35 1.12 0.84 0.91 1.00 0.94 1.01 0.99 0.98 1.87 1.03 1.00
f1 (ppm)

^{13}C NMR (151 MHz, Chloroform-*d*)

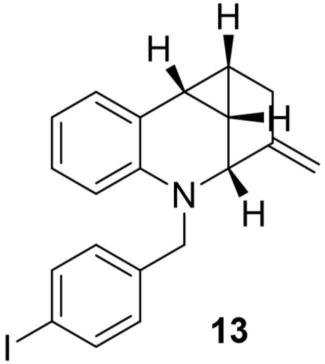

13

(±)

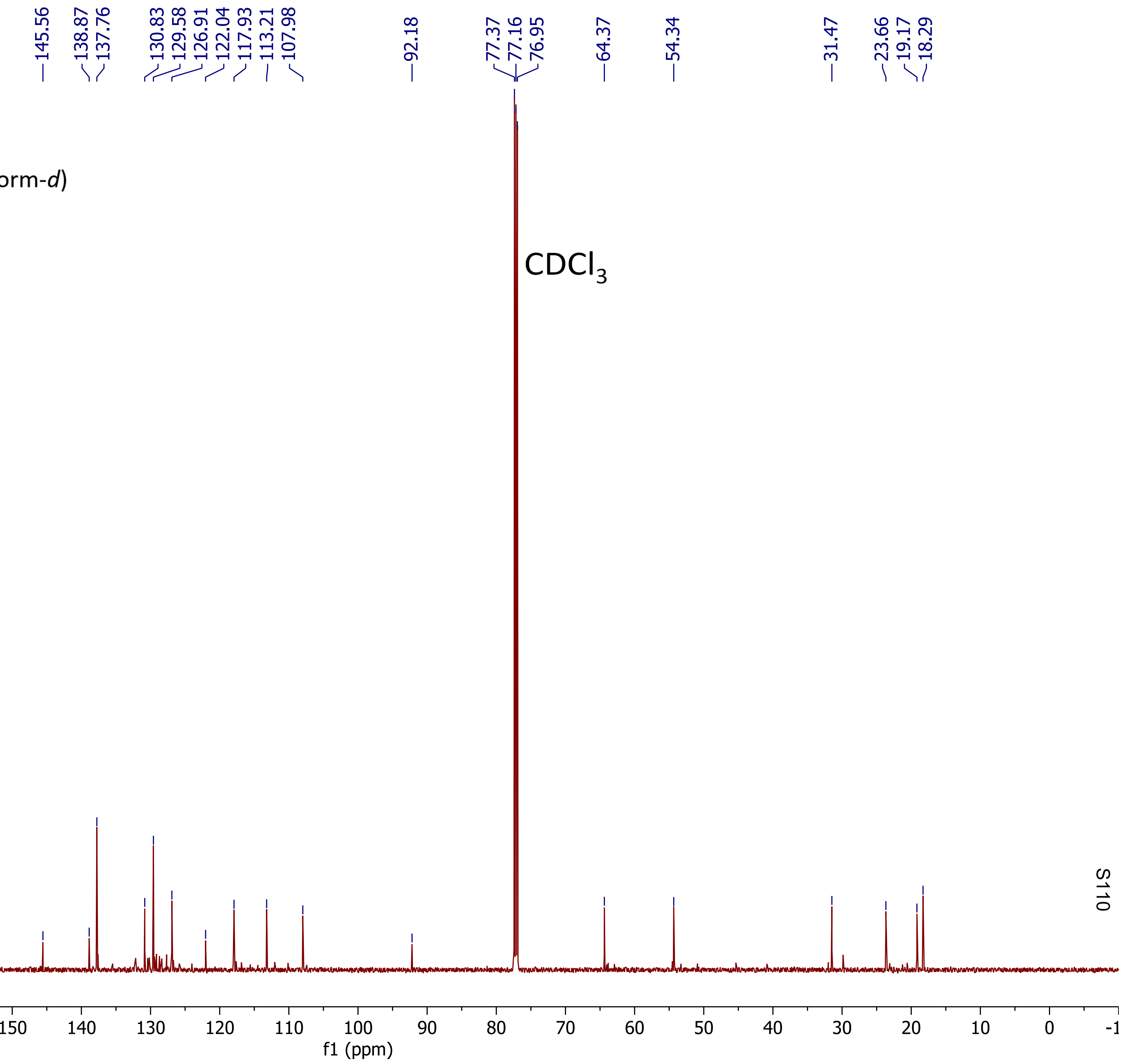

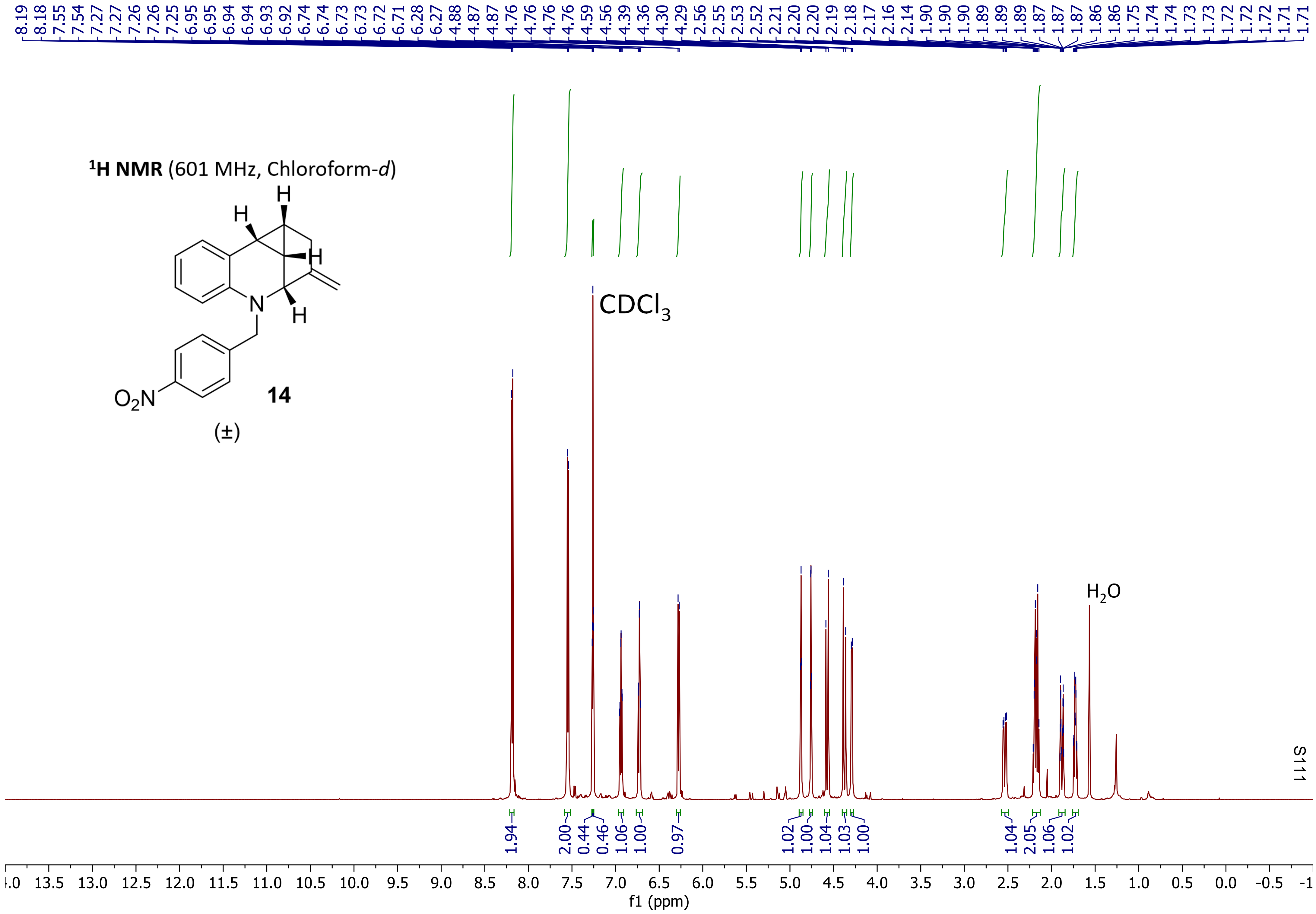

1H NMR (601 MHz, Chloroform-d)
14
(±)
O2N
N
H
CDCl3
H2O
8.19 8.18 7.55 7.54 7.27 7.27 7.26 7.26 7.25 6.95 6.95 6.94 6.94 6.93 6.92 6.74 6.74 6.73 6.73 6.72 6.71 6.28 6.27 4.88 4.87 4.87 4.76 4.76 4.76 4.76 4.59 4.56 4.39 4.36 4.30 4.29 2.56 2.55 2.53 2.52 2.21 2.20 2.20 2.19 2.18 2.17 2.16 2.14 1.90 1.90 1.90 1.89 1.89 1.87 1.87 1.87 1.86 1.86 1.75 1.74 1.74 1.73 1.73 1.72 1.72 1.72 1.71 1.71
1.94 2.00 0.44 0.46 1.06 1.00 0.97 1.02 1.00 1.04 1.03 1.00 1.04 2.05 1.06 1.02
f1 (ppm)

^{13}C NMR (151 MHz, Chloroform-*d*)

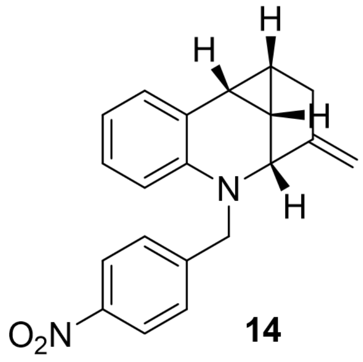

14

(±)

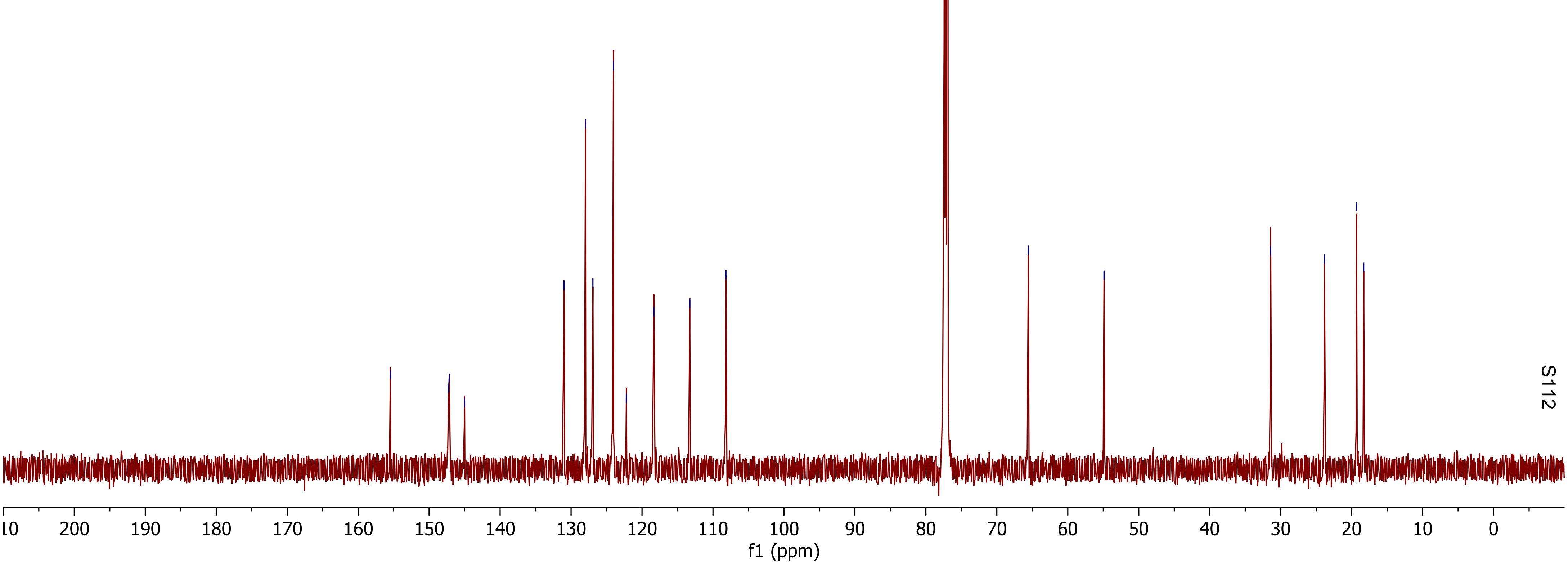

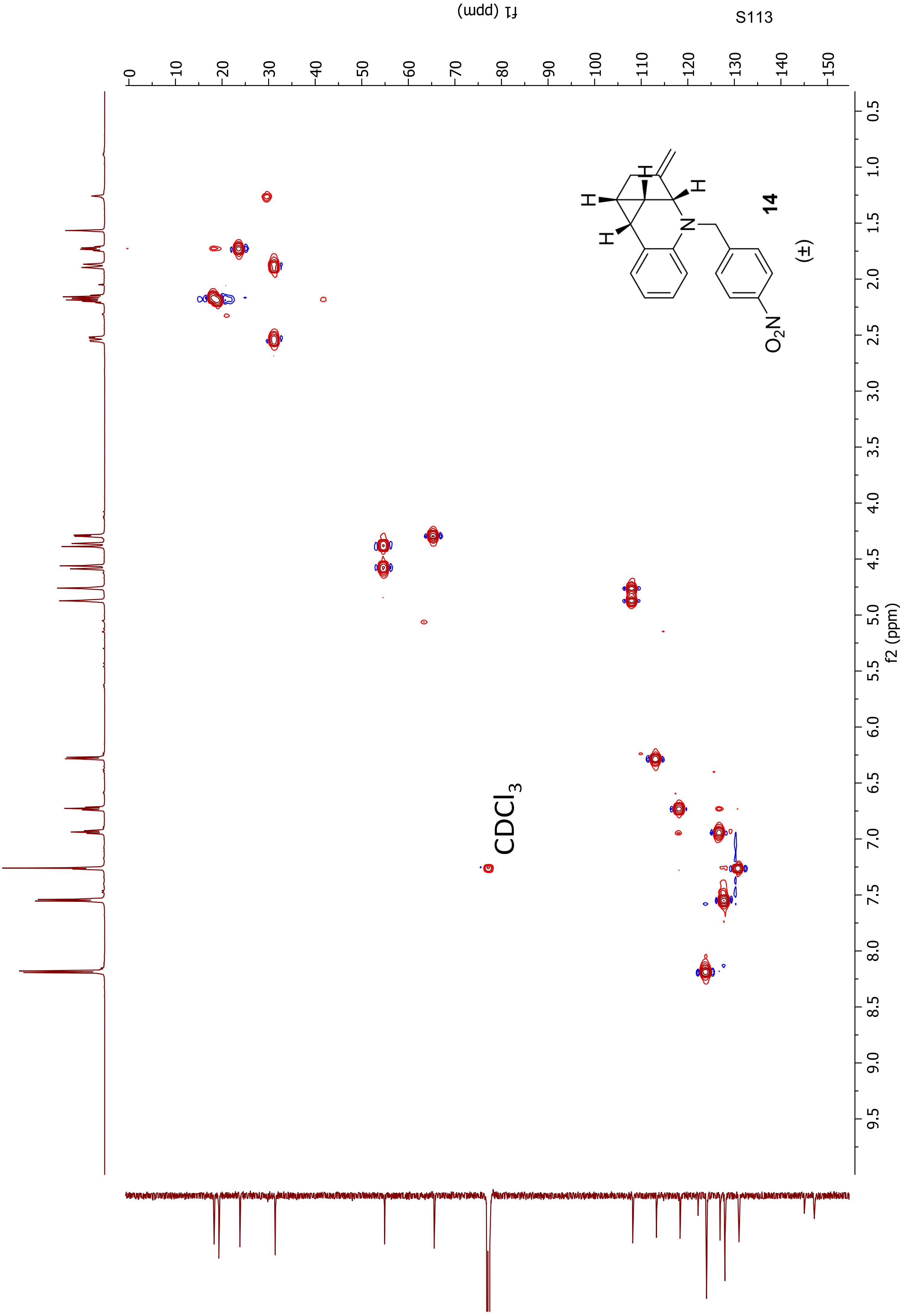
f1 (ppm)
f2 (ppm)
14
(±)
CDCl3
O2N

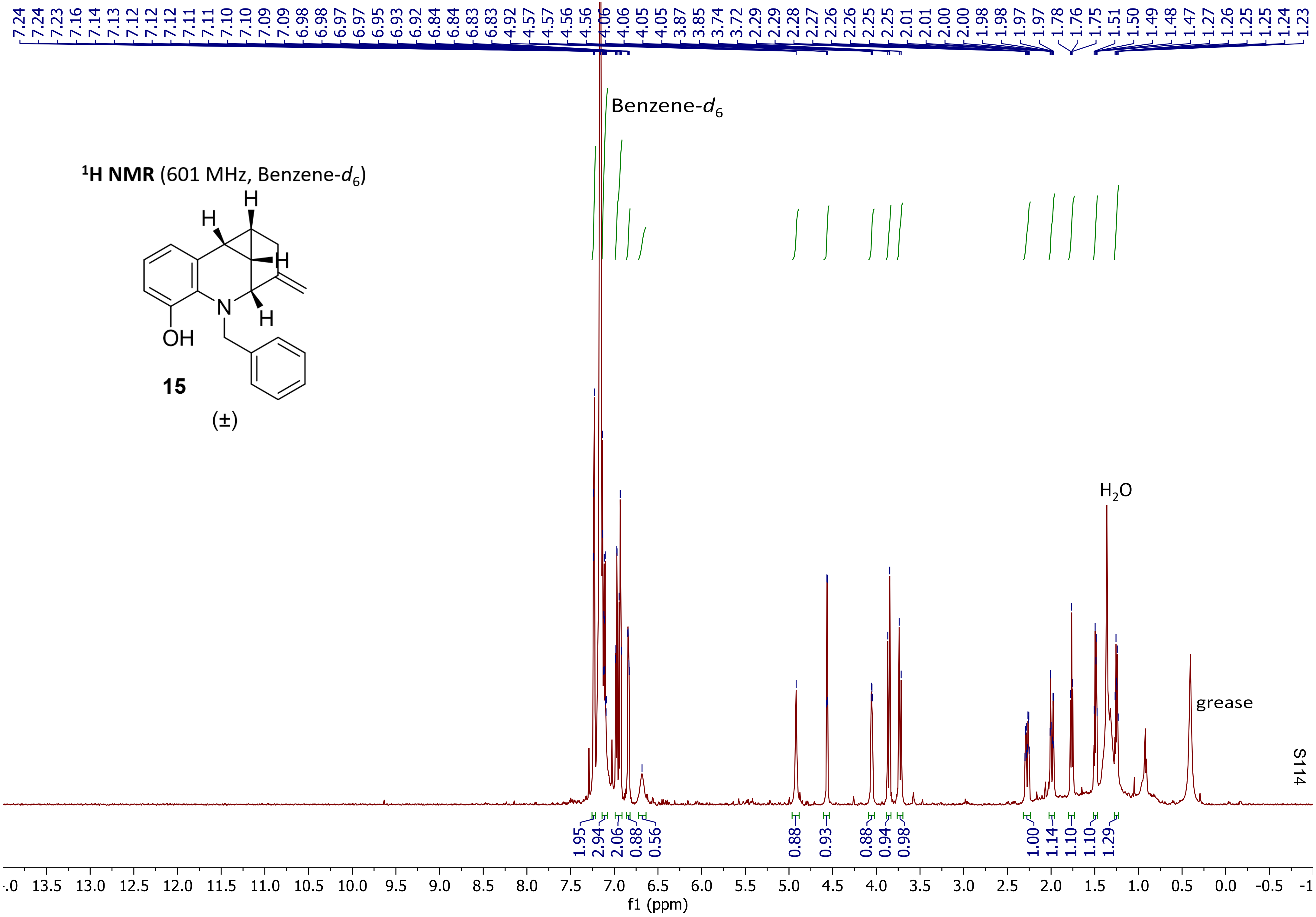

7.24 7.24 7.23 7.16 7.14 7.13 7.12 7.12 7.12 7.11 7.11 7.10 7.10 7.09 7.09 6.98 6.98 6.97 6.97 6.95 6.93 6.92 6.84 6.84 6.83 6.83 4.92 4.57 4.57 4.56 4.56 4.06 4.06 4.06 4.05 4.05 3.87 3.85 3.74 3.72 2.29 2.29 2.28 2.27 2.26 2.26 2.25 2.25 2.01 2.01 2.00 2.00 1.98 1.98 1.97 1.97 1.78 1.76 1.75 1.51 1.50 1.49 1.48 1.47 1.27 1.26 1.25 1.25 1.24 1.23
Benzene-d_6
^{1}H NMR (601 MHz, Benzene-d_6)
H
H
H
H
N
OH
15
(±)
H_2O
grease
1.95 2.94 2.06 0.88 0.56 0.88 0.93 0.88 0.94 0.98 1.00 1.14 1.10 1.10 1.29
14.0 13.5 13.0 12.5 12.0 11.5 11.0 10.5 10.0 9.5 9.0 8.5 8.0 7.5 7.0 6.5 6.0 5.5 5.0 4.5 4.0 3.5 3.0 2.5 2.0 1.5 1.0 0.5 0.0 -0.5 -1
f1 (ppm)

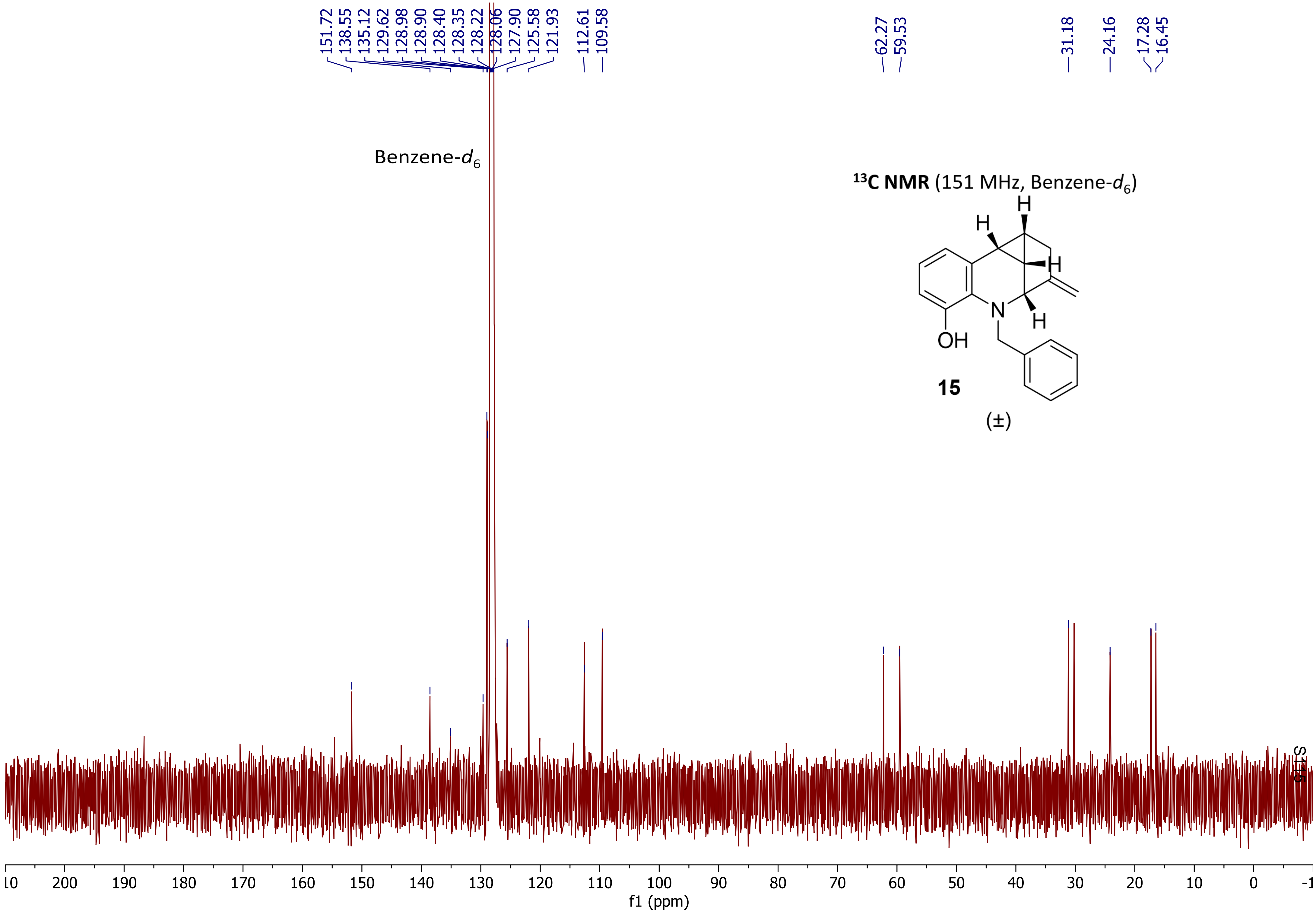

151.72
138.55
135.12
129.62
128.98
128.90
128.40
128.35
128.22
128.06
127.90
125.58
121.93
112.61
109.58
62.27
59.53
31.18
24.16
17.28
16.45
Benzene-d_6
^{13}C NMR (151 MHz, Benzene-d_6)
H
H
H
H
N
OH
15
(±)
f1 (ppm)

^{1}H NMR (601 MHz, Chloroform-*d*)

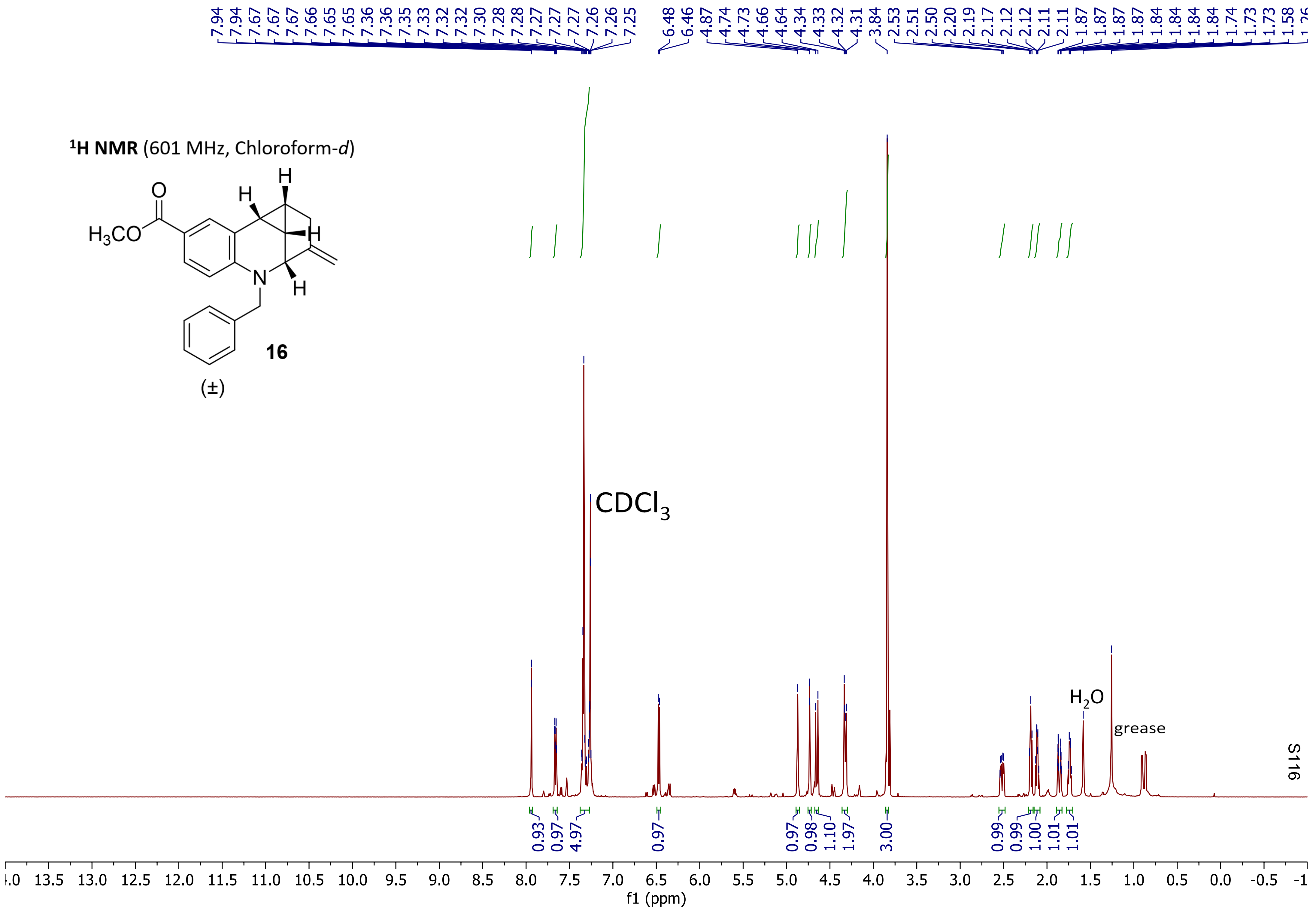

^{13}C **NMR** (151 MHz, Chloroform-*d*)

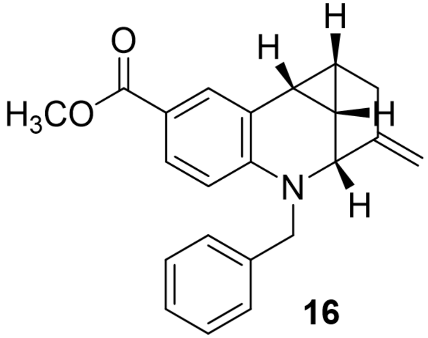

16

(±)

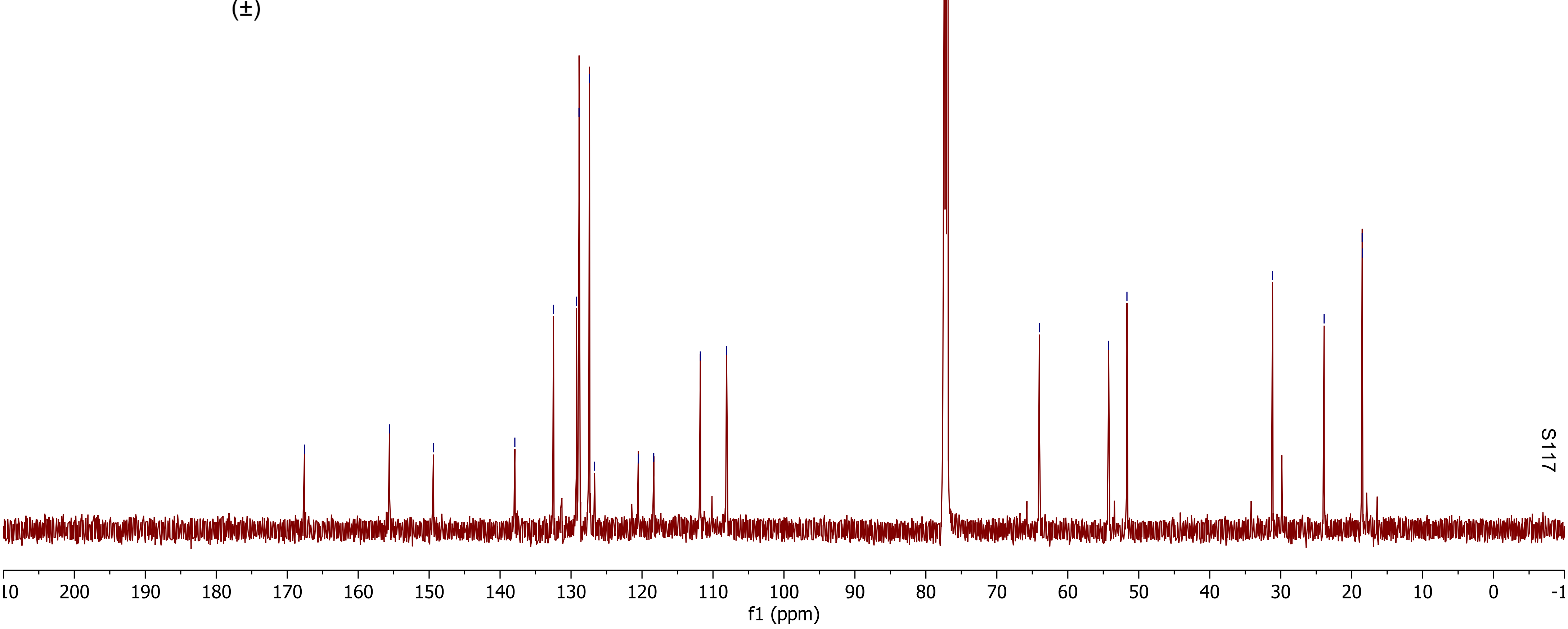

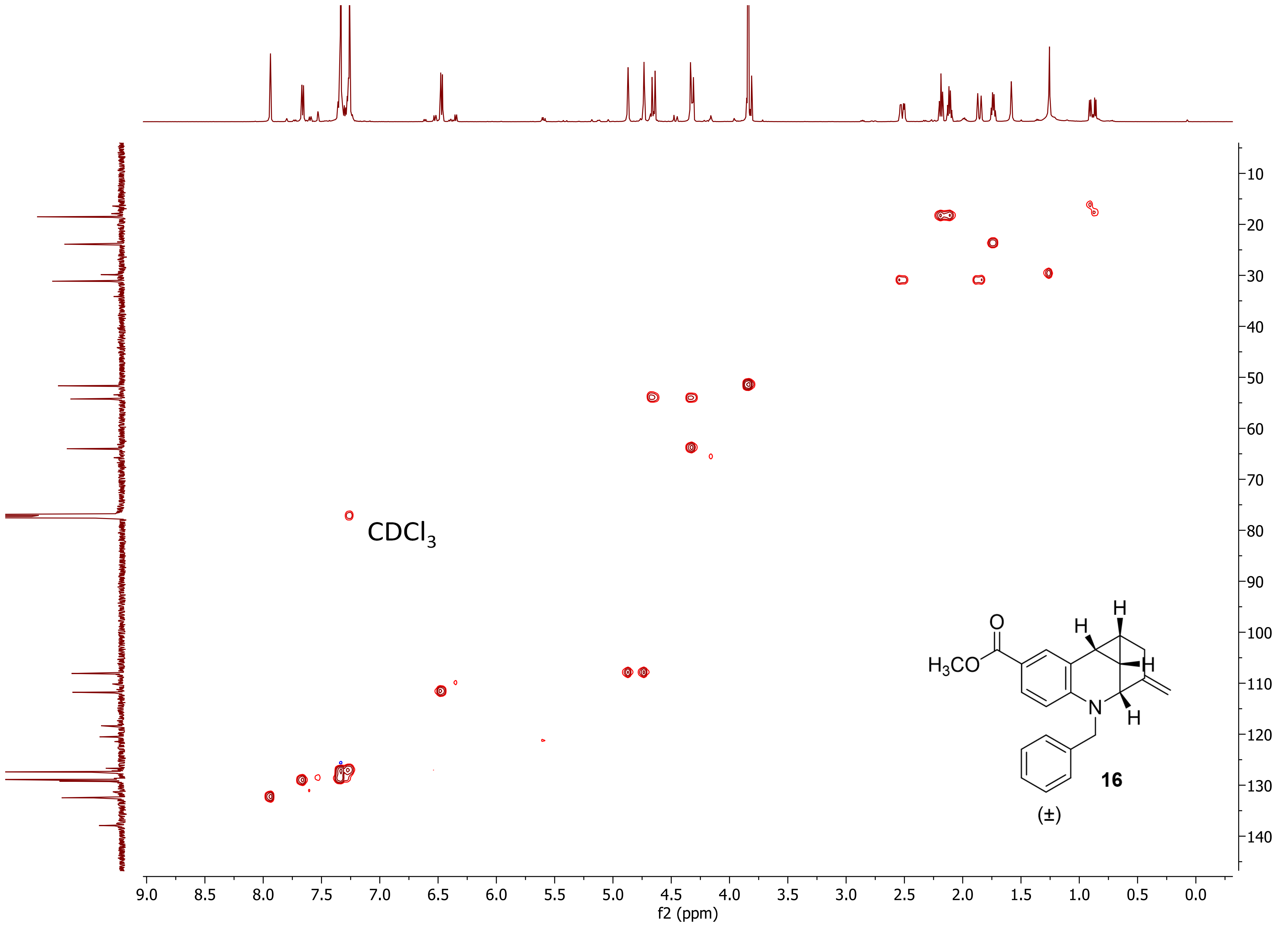
CDCl3
H3CO
O
H
N
16
(±)
f1 (ppm)
f2 (ppm)

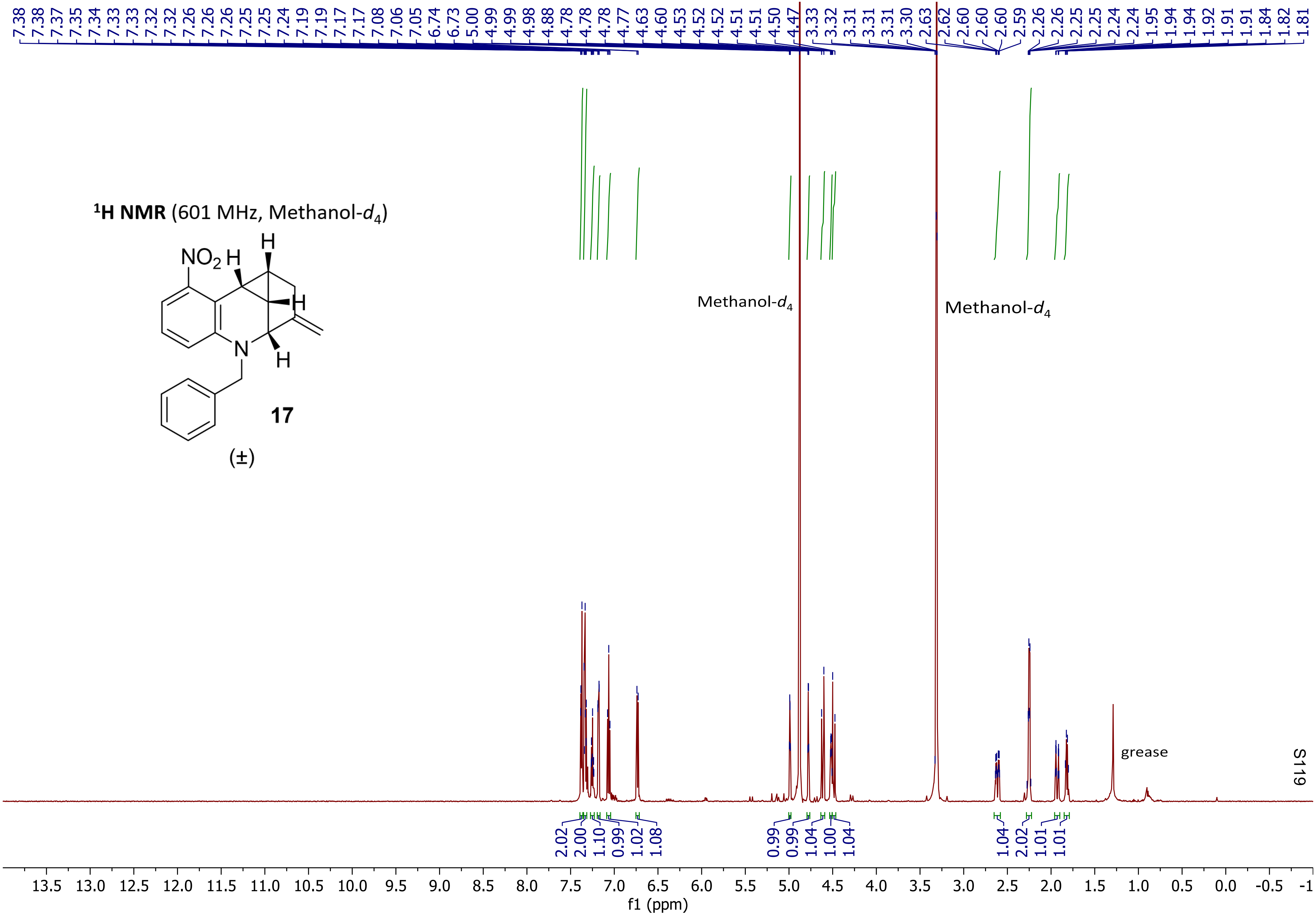

^{1}H NMR (601 MHz, Methanol-d_4)

17

(±)

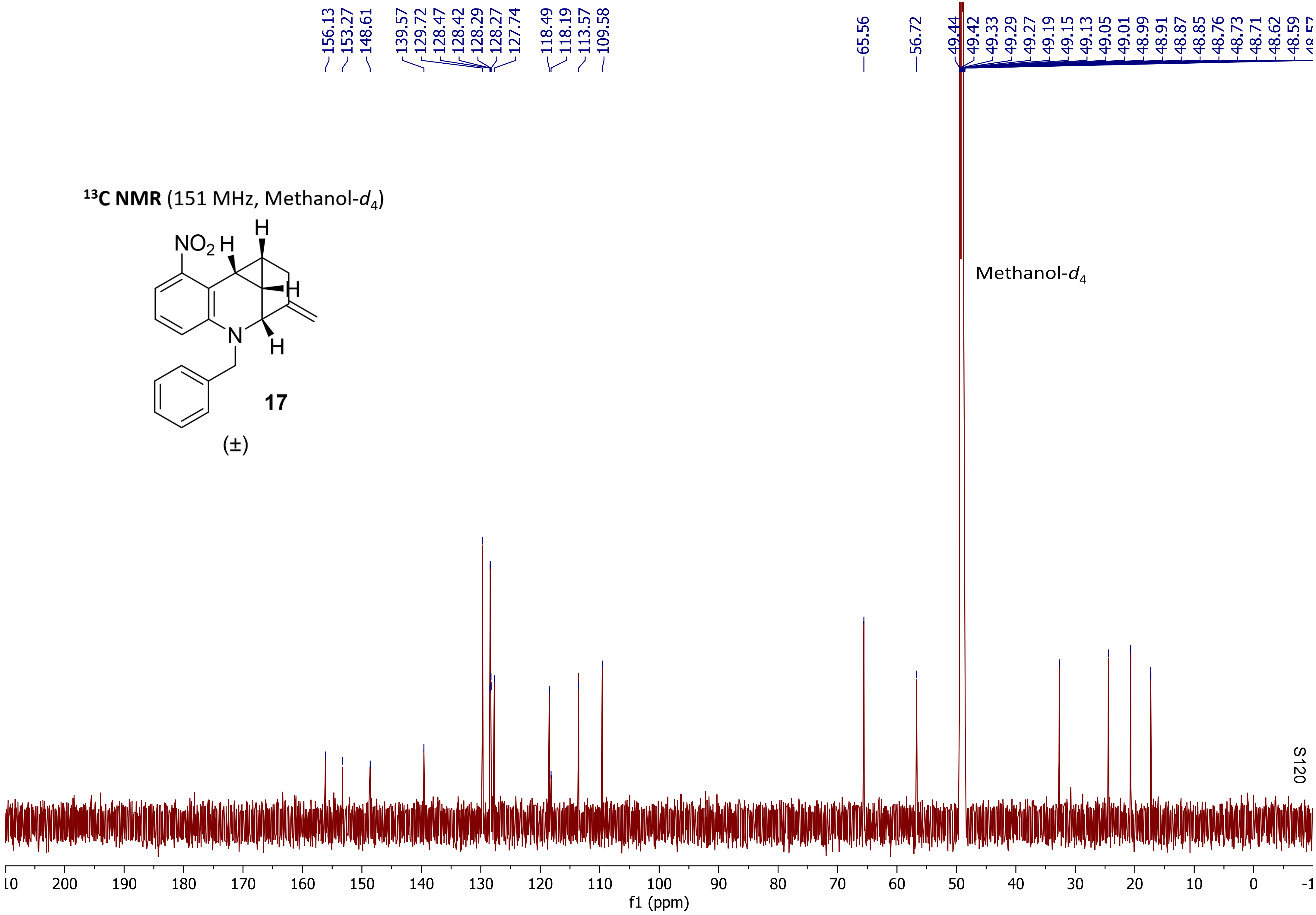

^{13}C NMR (151 MHz, Methanol-d_4)

17

(±)

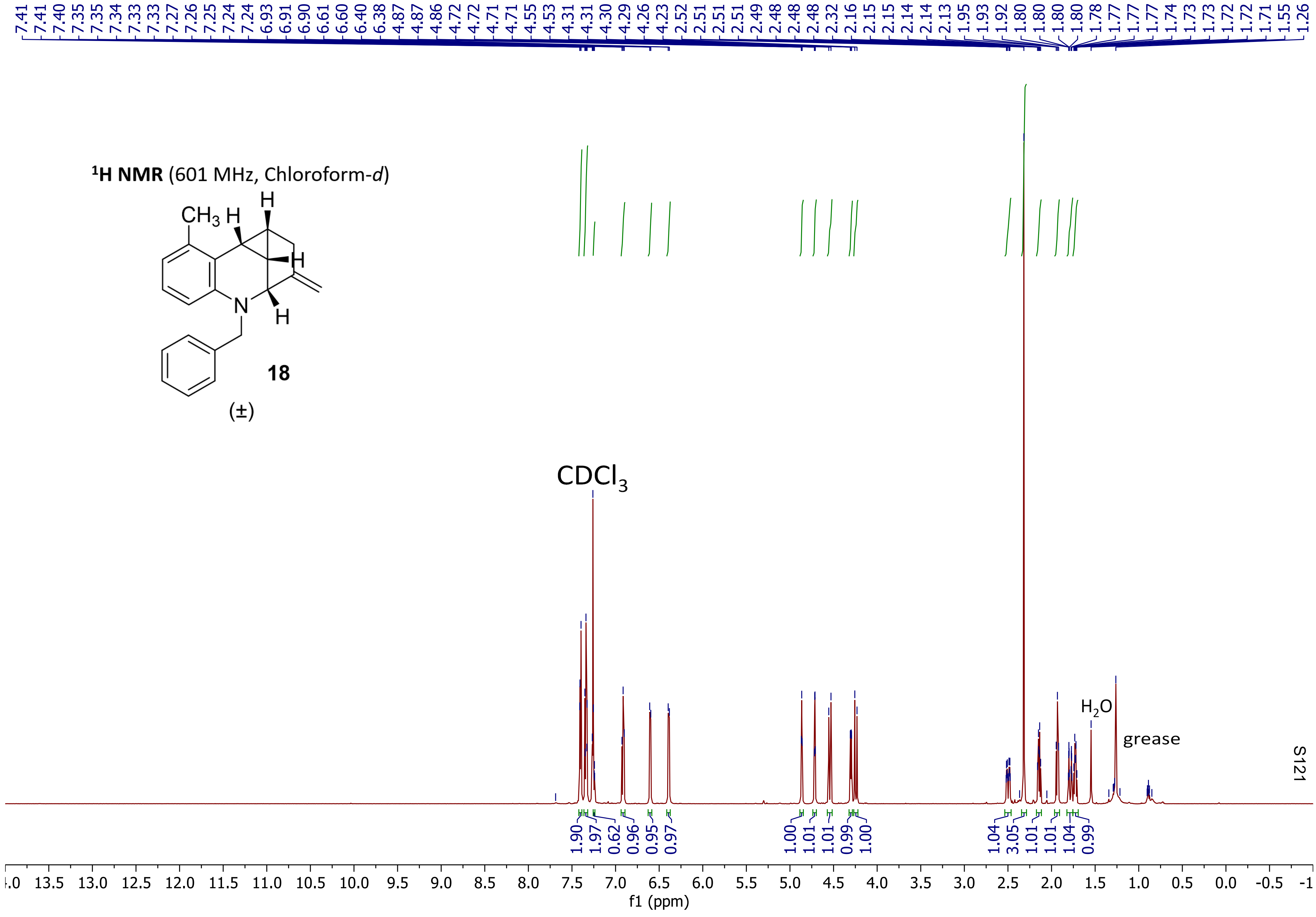

7.41 7.41 7.40 7.35 7.35 7.34 7.33 7.33 7.27 7.26 7.25 7.24 7.24 6.93 6.91 6.90 6.61 6.60 6.40 6.38 4.87 4.87 4.86 4.72 4.72 4.71 4.71 4.55 4.53 4.31 4.31 4.30 4.29 4.26 4.23 2.52 2.51 2.51 2.51 2.49 2.48 2.48 2.48 2.32 2.16 2.15 2.15 2.14 2.14 2.13 1.95 1.93 1.92 1.80 1.80 1.80 1.80 1.78 1.77 1.77 1.77 1.74 1.73 1.73 1.72 1.72 1.71 1.55 1.26
¹H NMR (601 MHz, Chloroform-d)
CH3 H
H
H
N
H
18
(±)
CDCl3
H2O
grease
1.90 1.97 0.62 0.96 0.95 0.97
1.00 1.01 1.01 0.99 1.00
1.04 3.05 1.01 1.01 1.04 0.99
14.0 13.5 13.0 12.5 12.0 11.5 11.0 10.5 10.0 9.5 9.0 8.5 8.0 7.5 7.0 6.5 6.0 5.5 5.0 4.5 4.0 3.5 3.0 2.5 2.0 1.5 1.0 0.5 0.0 -0.5 -1
f1 (ppm)

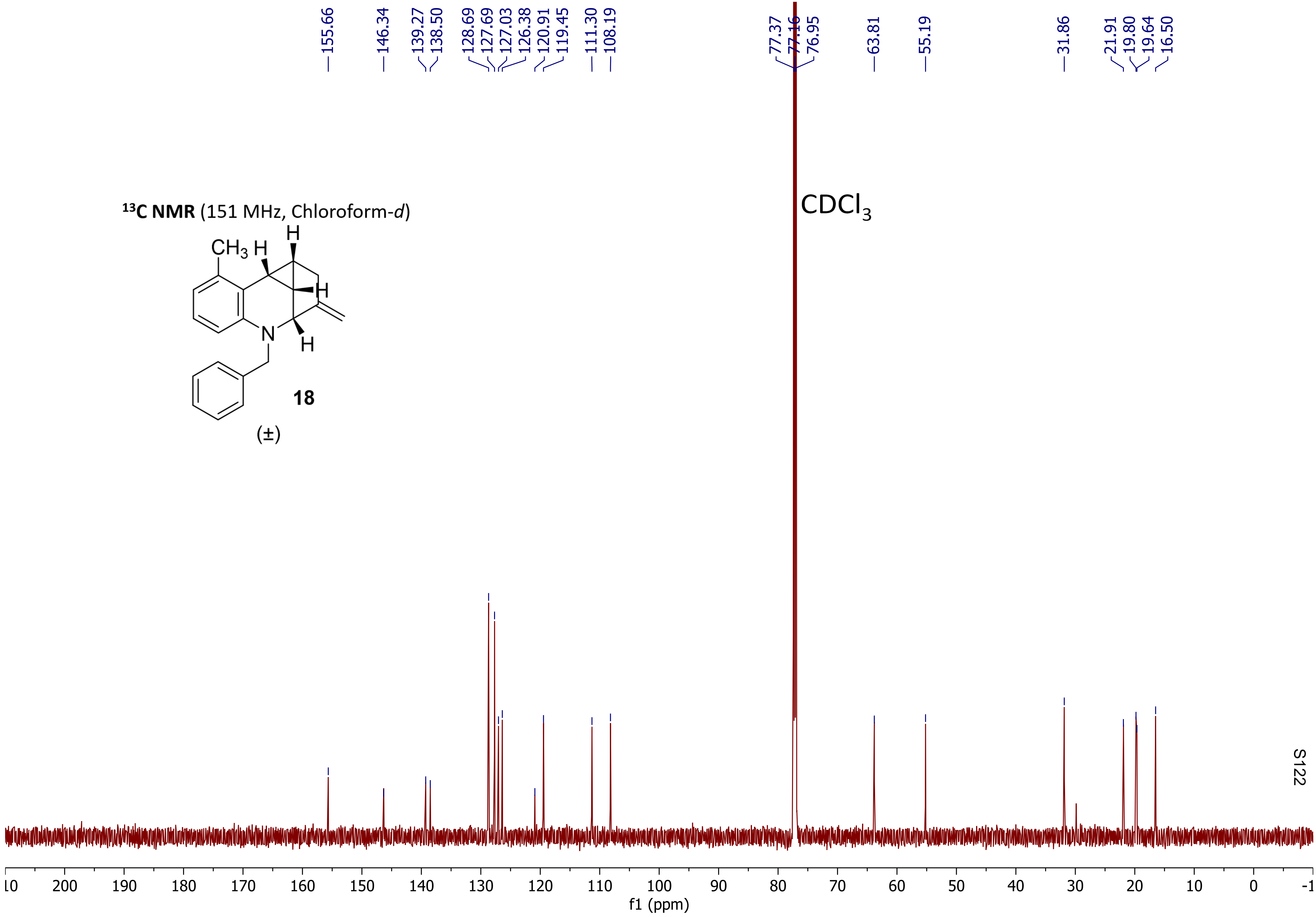

^{13}C NMR (151 MHz, Chloroform-*d*)

18

(±)

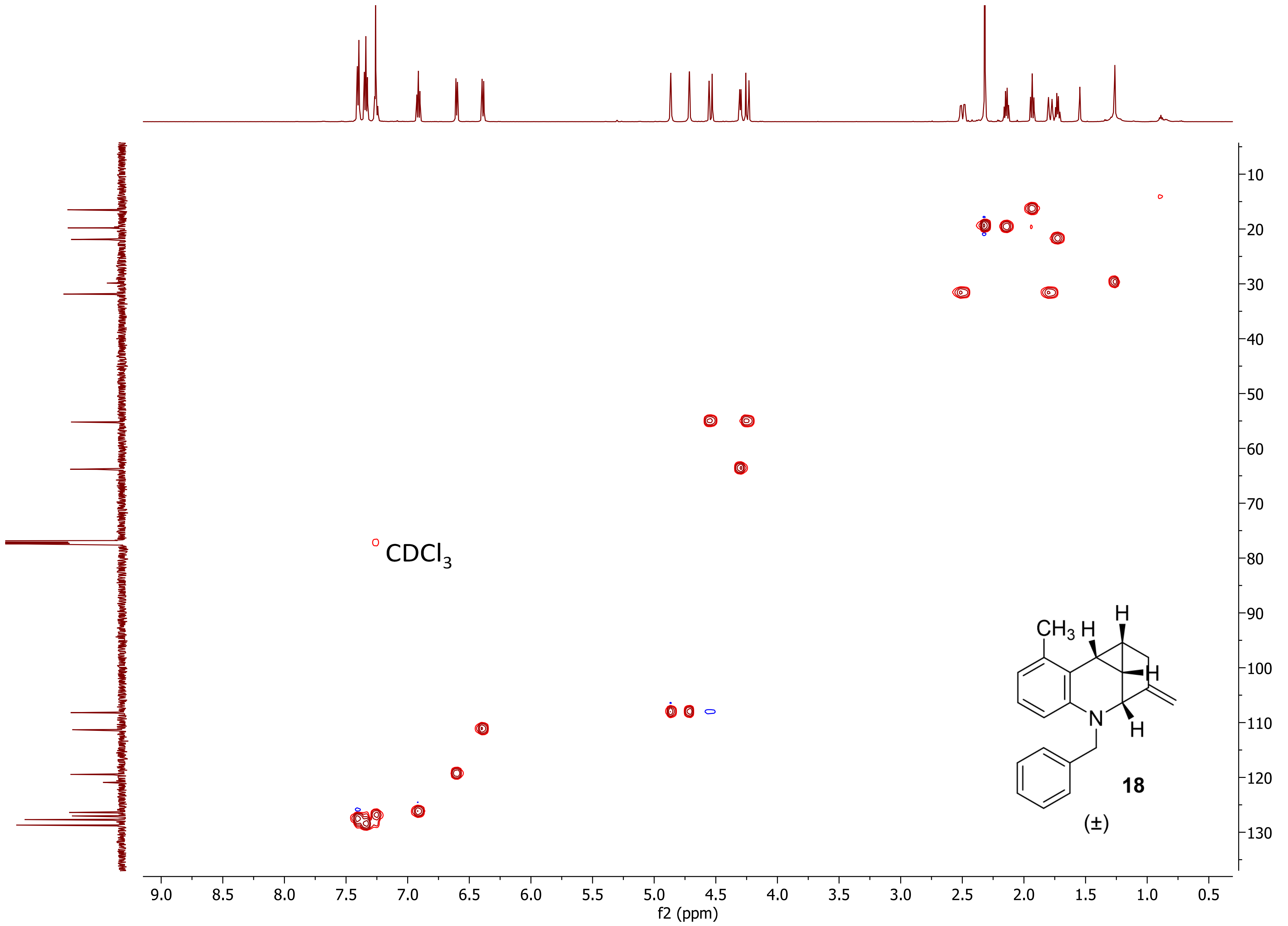
f2 (ppm)
f1 (ppm)
CDCl3
CH3 H
H
H
N
H
18
(±)

^{1}H NMR (601 MHz, Chloroform-d)

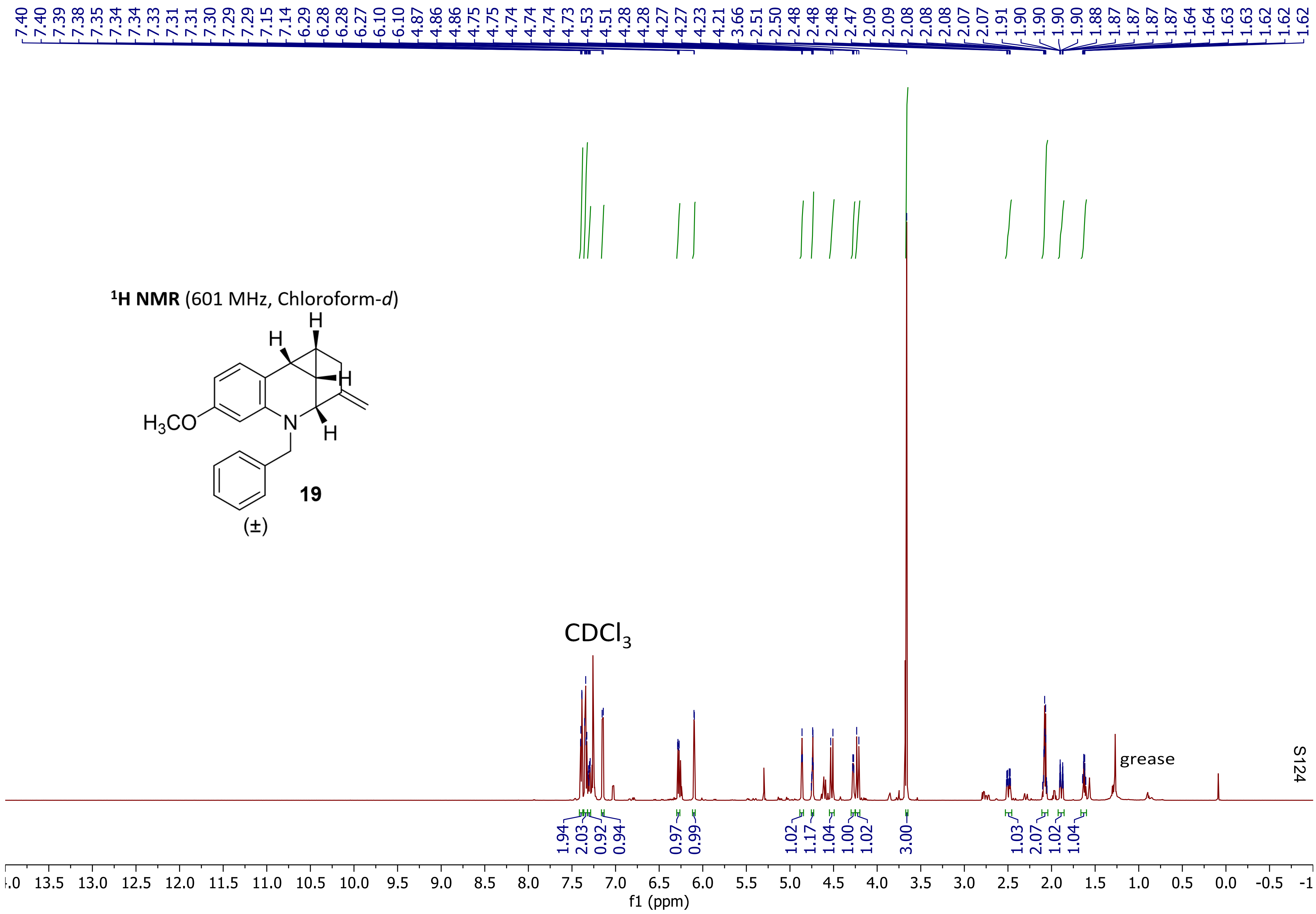

19

^{13}C NMR (151 MHz, Chloroform-*d*)

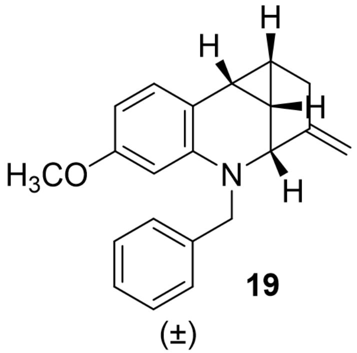

19

(±)

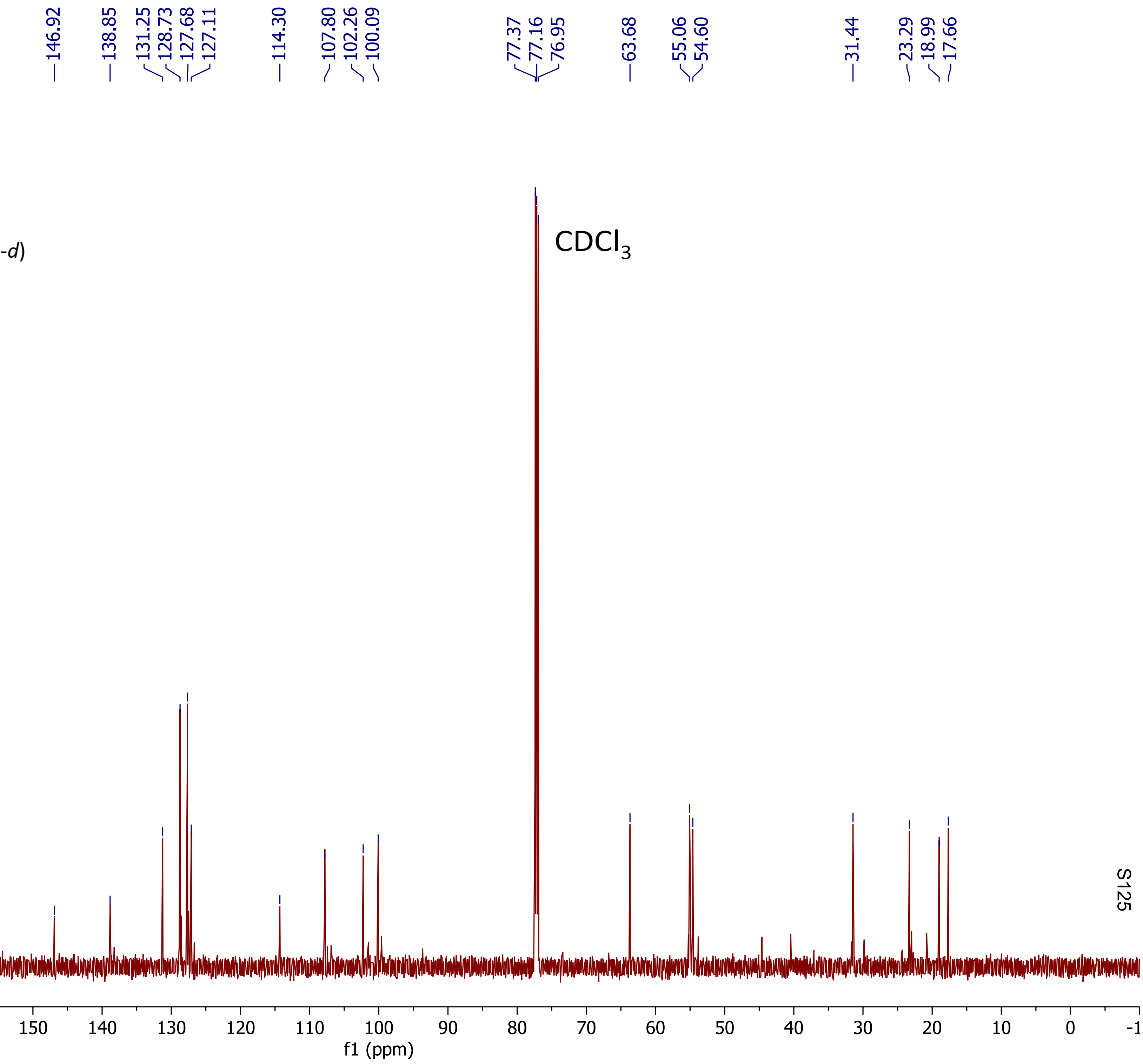

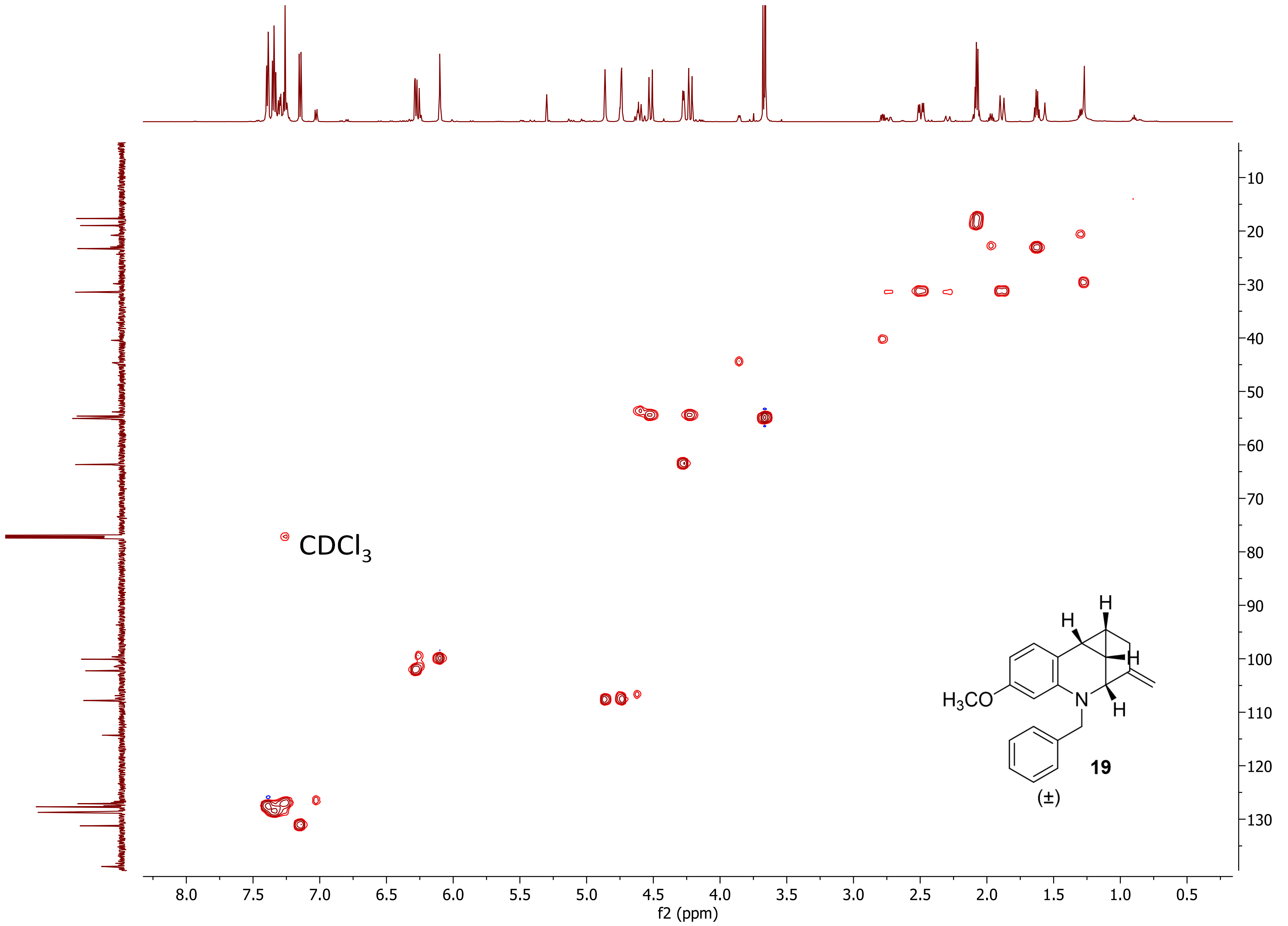
CDCl$_3$
H$_3$CO
N
H
19
(±)
f2 (ppm)
f1 (ppm)

^{1}H NMR (700 MHz, Benzene-d_6)

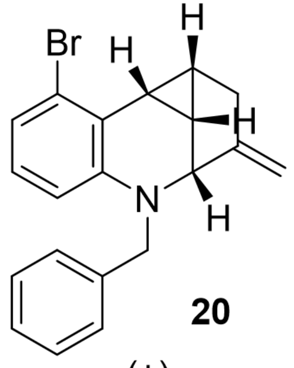

20

(±)

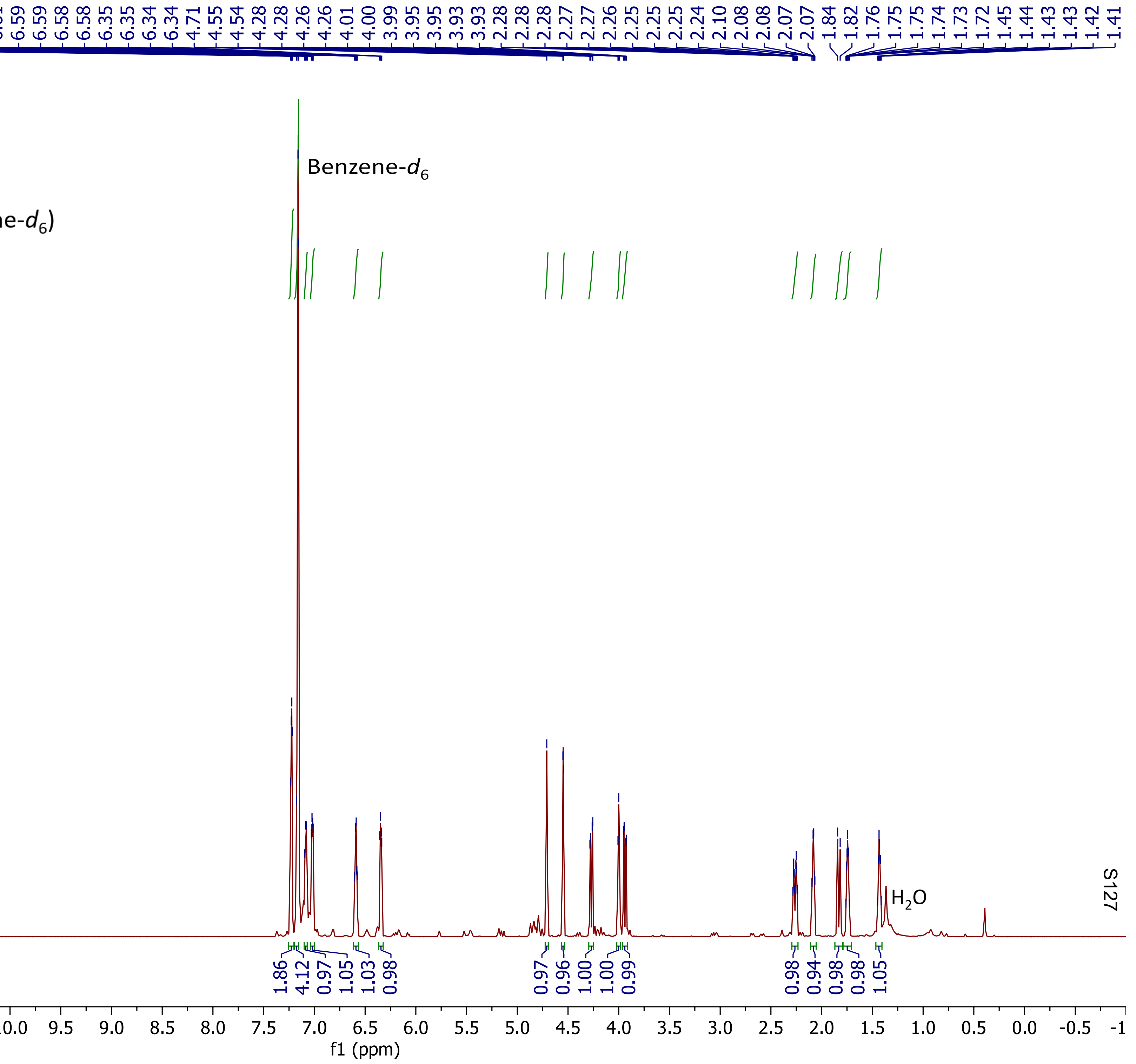

^{13}C NMR (176 MHz, Benzene-d_6)

20

(±)

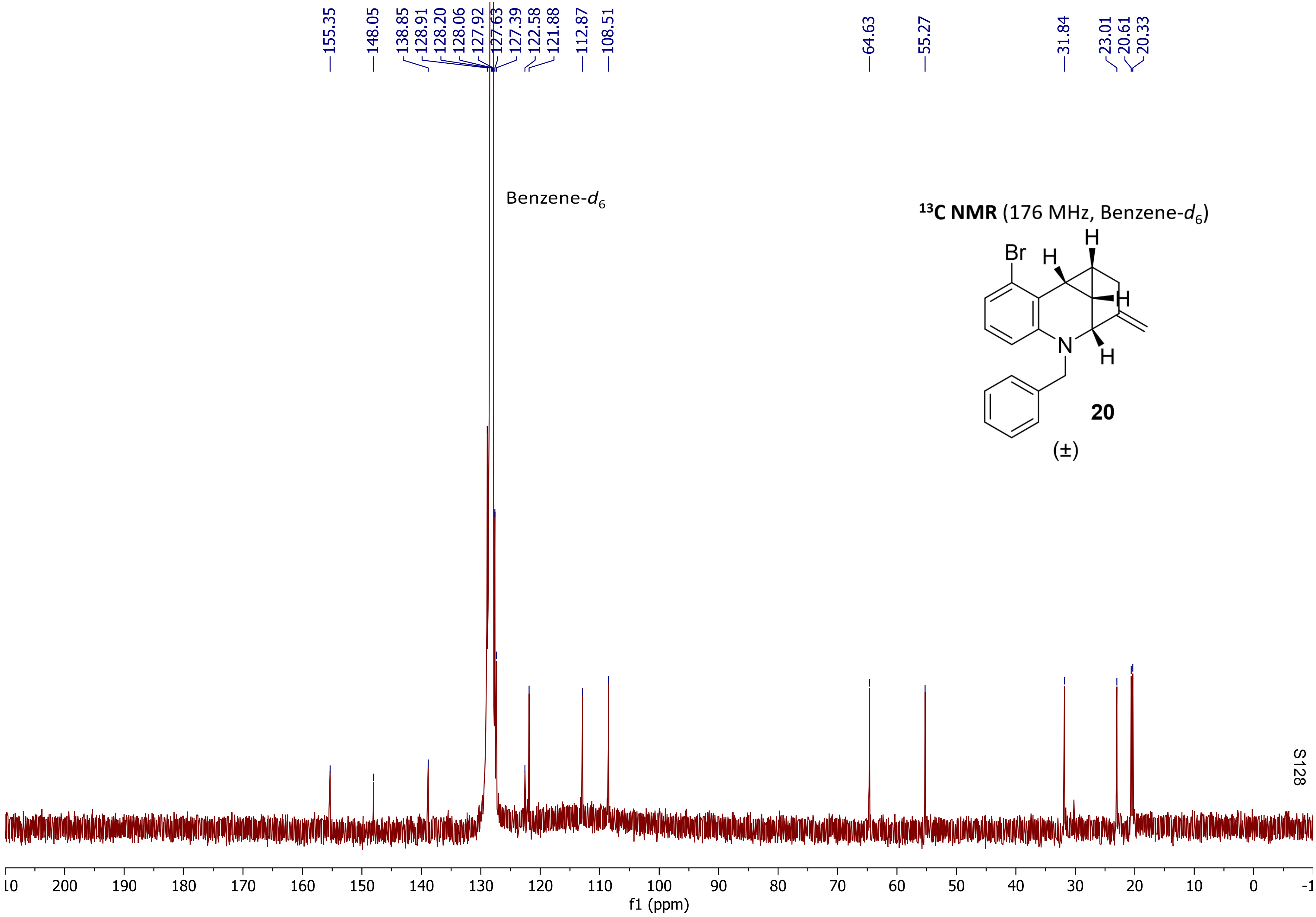

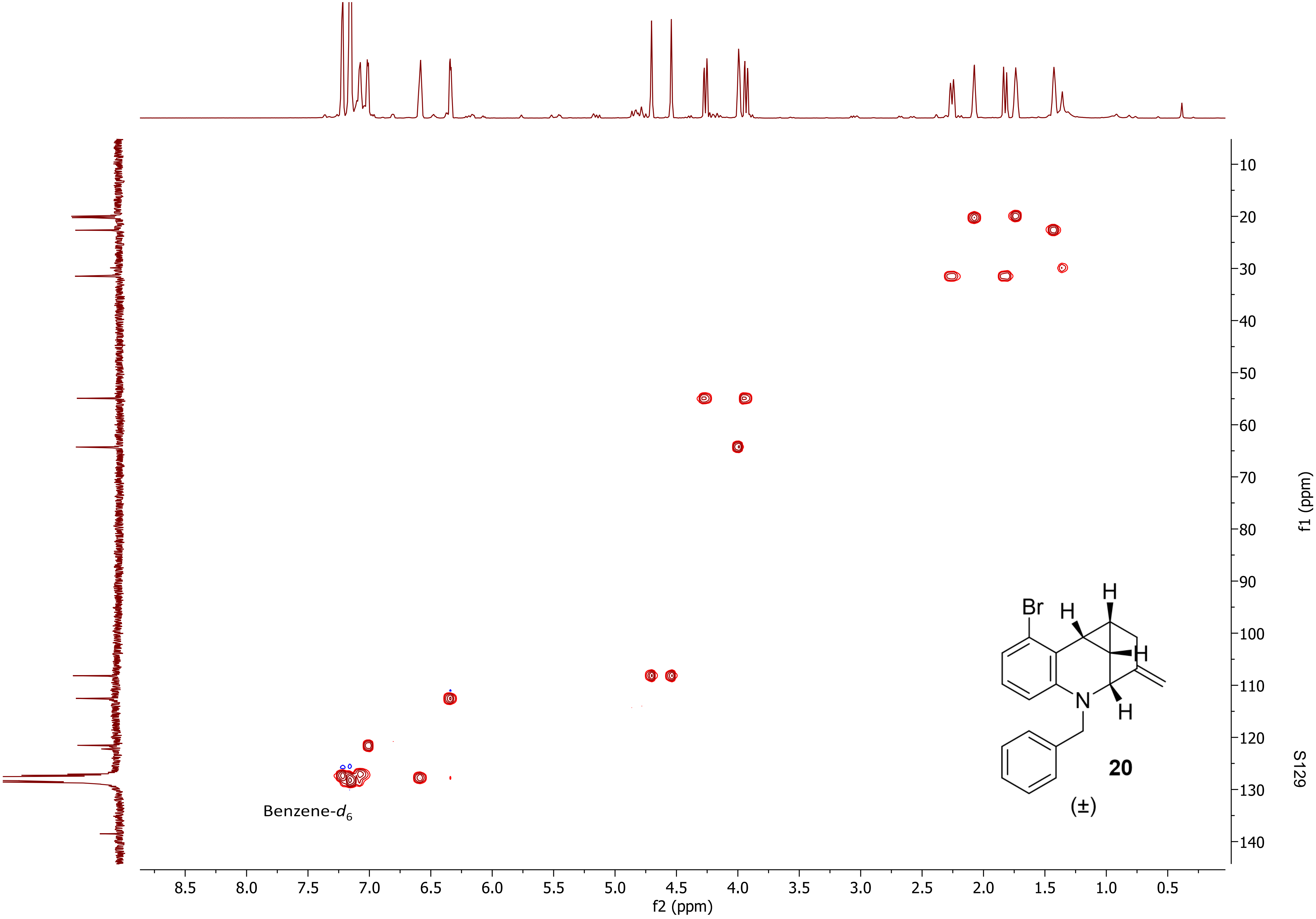
Br
H
H
H
N
H
20
(±)
Benzene-d_6
f2 (ppm)
f1 (ppm)

¹H NMR (500 MHz, Chloroform-*d*)

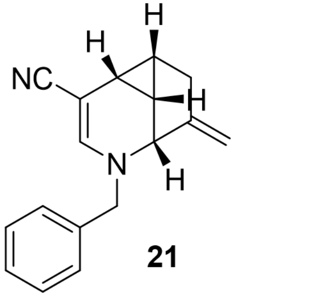

21

(±)

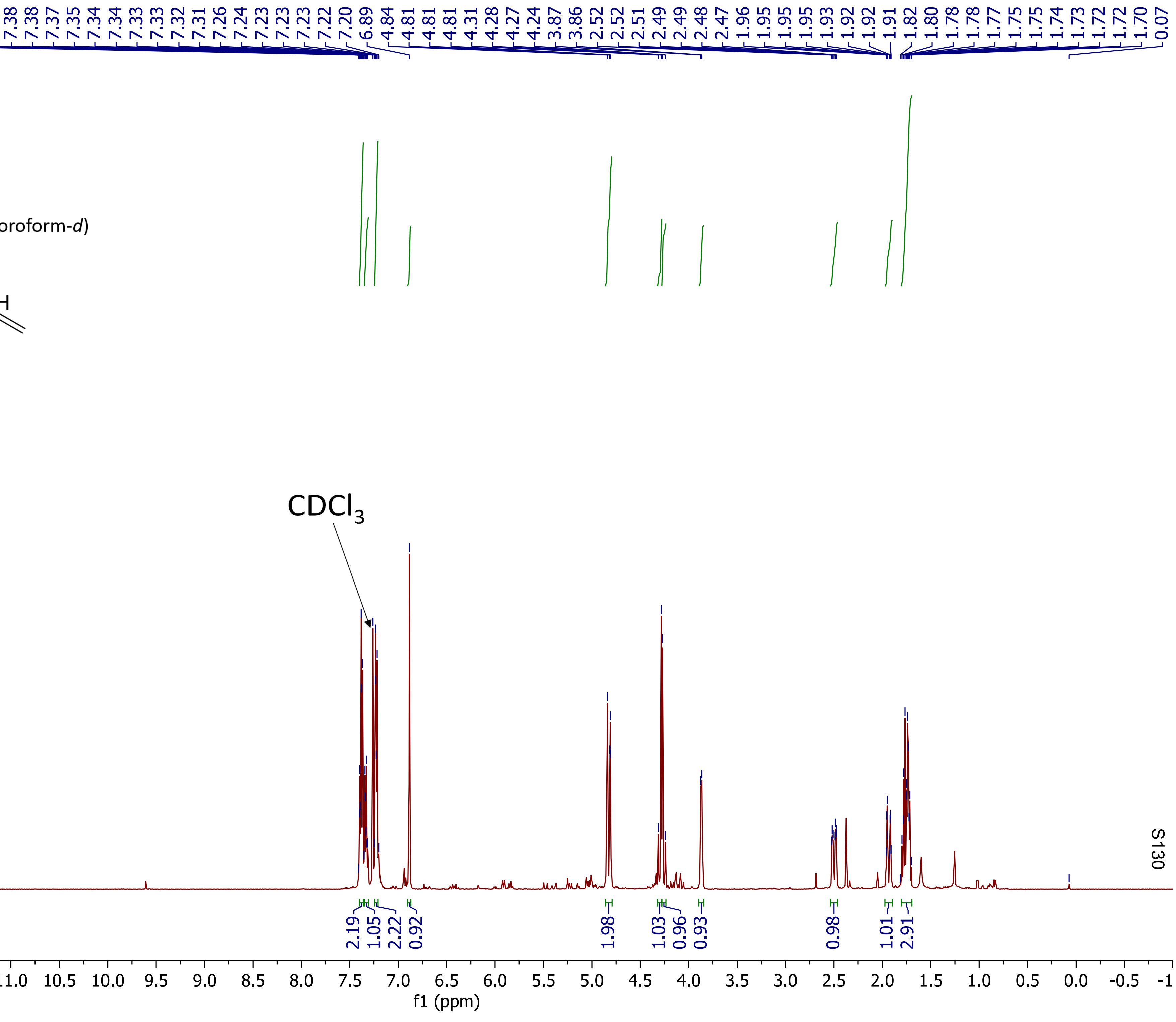

^{13}C NMR (126 MHz, Chloroform-*d*)

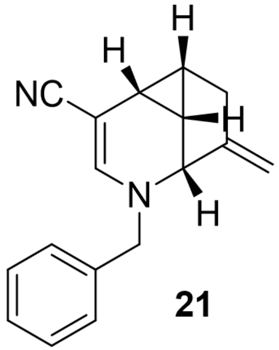

21

(±)

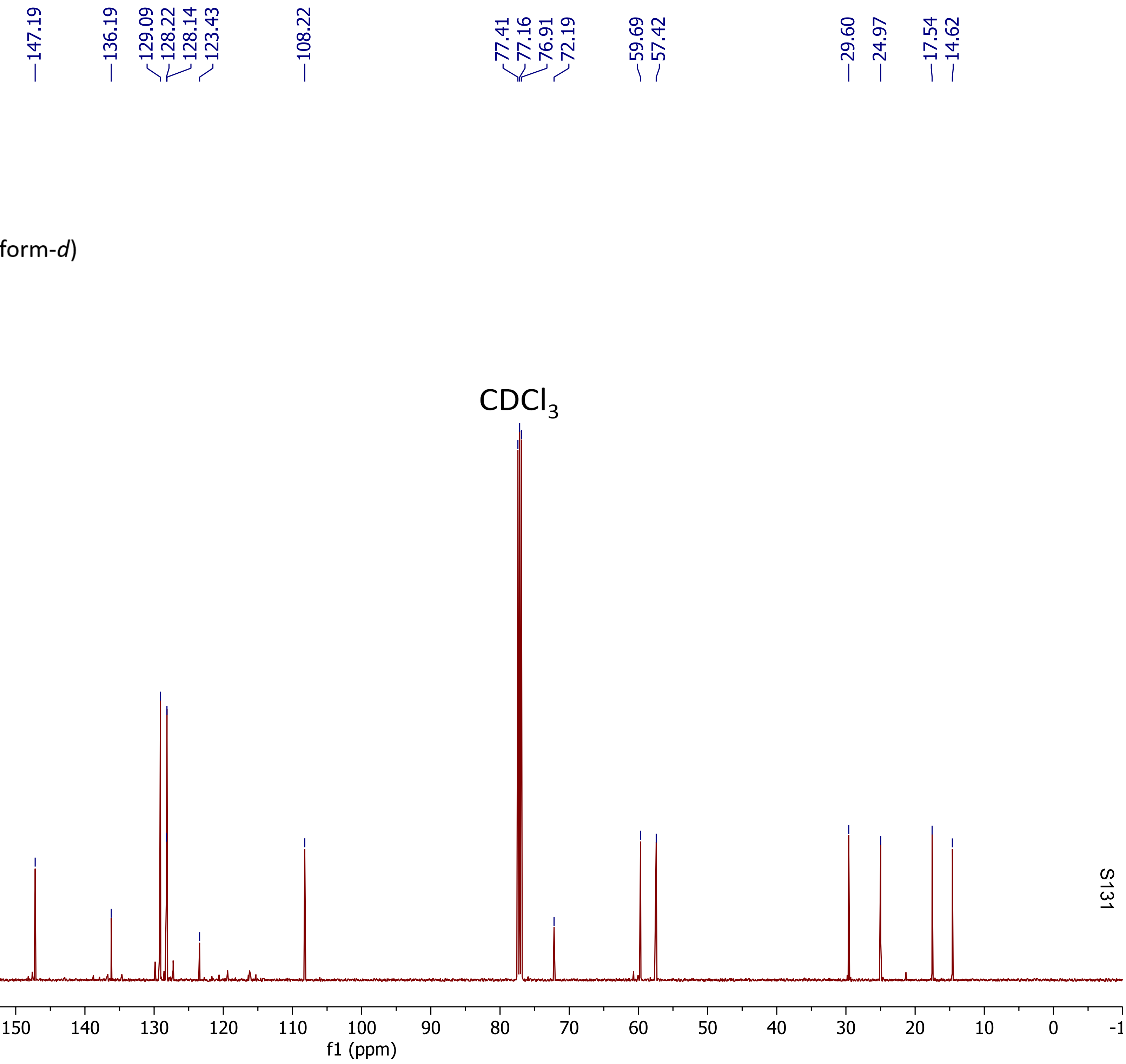

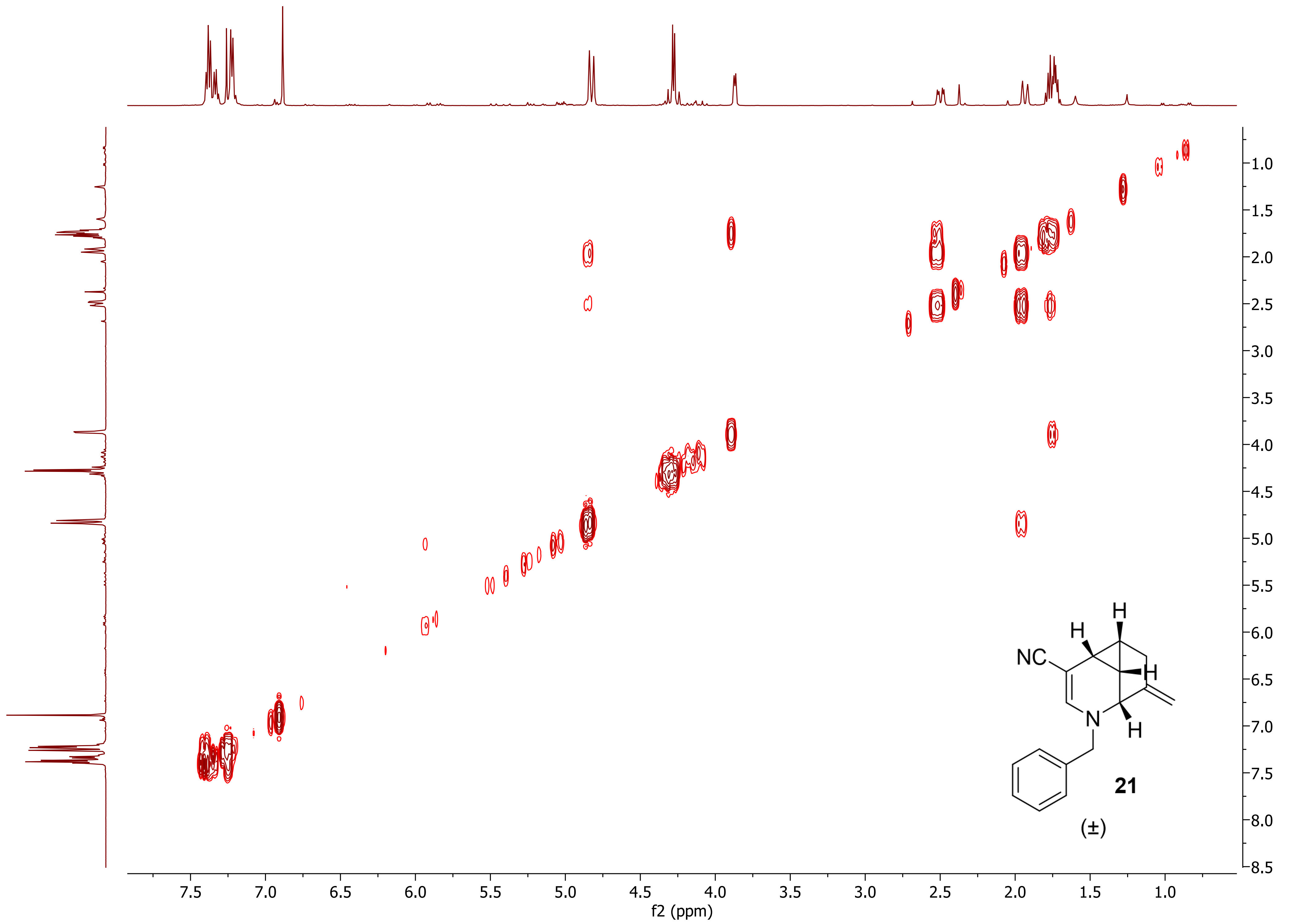
f2 (ppm)
f1 (ppm)
NC
N
H
21
(±)

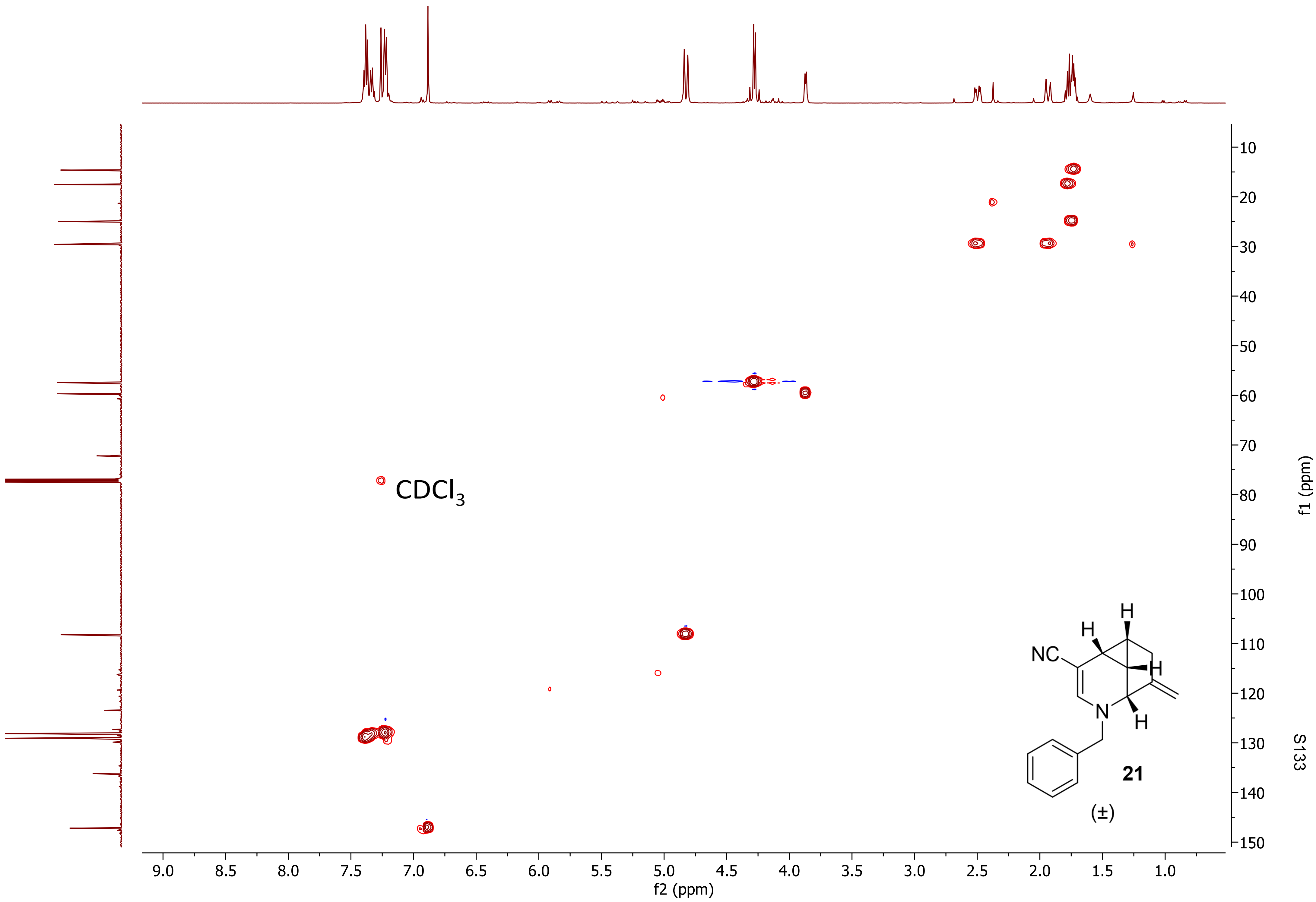
CDCl3
NC
H
H
H
N
H
21
(±)
f2 (ppm)
f1 (ppm)

^{1}H NMR (500 MHz, Chloroform-*d*)

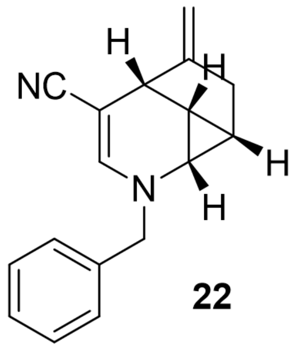

22

(±)

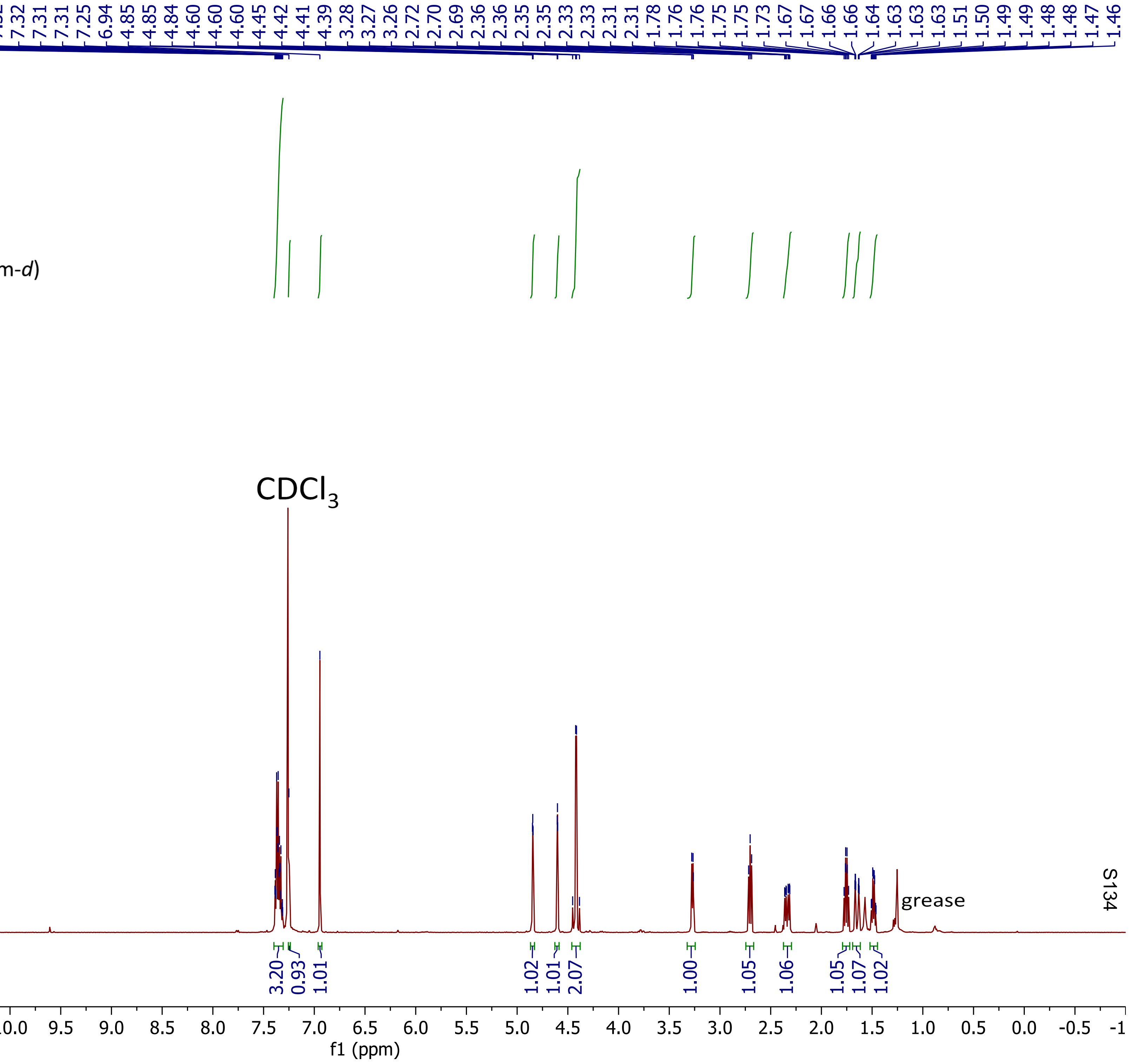

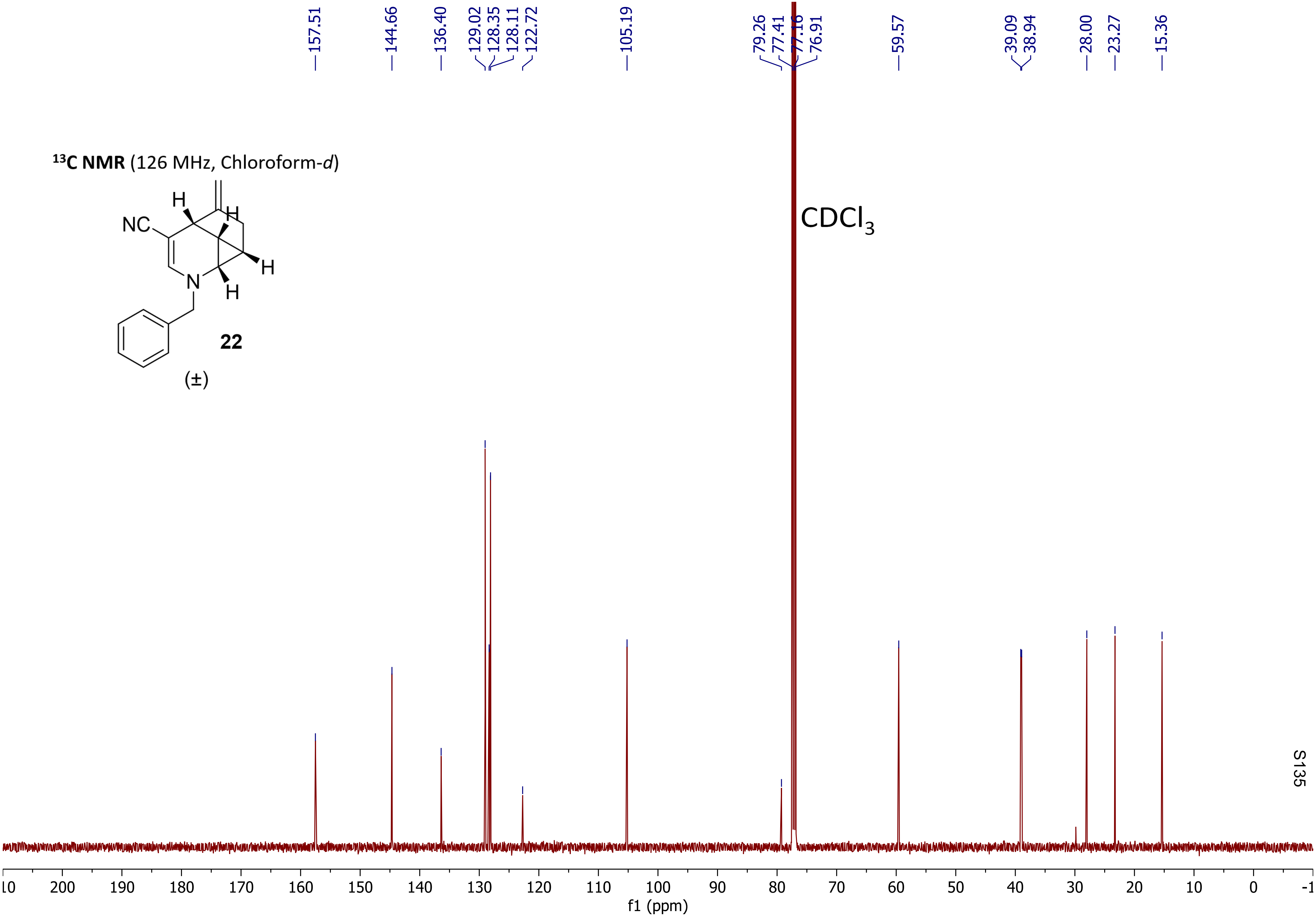
13C NMR (126 MHz, Chloroform-d)
NC
H
H
H
H
N
22
(±)
CDCl3
157.51
144.66
136.40
129.02
128.35
128.11
122.72
105.19
79.26
77.41
77.16
76.91
59.57
39.09
38.94
28.00
23.27
15.36
f1 (ppm)
10
200
190
180
170
160
150
140
130
120
110
100
90
80
70
60
50
40
30
20
10
0
-1

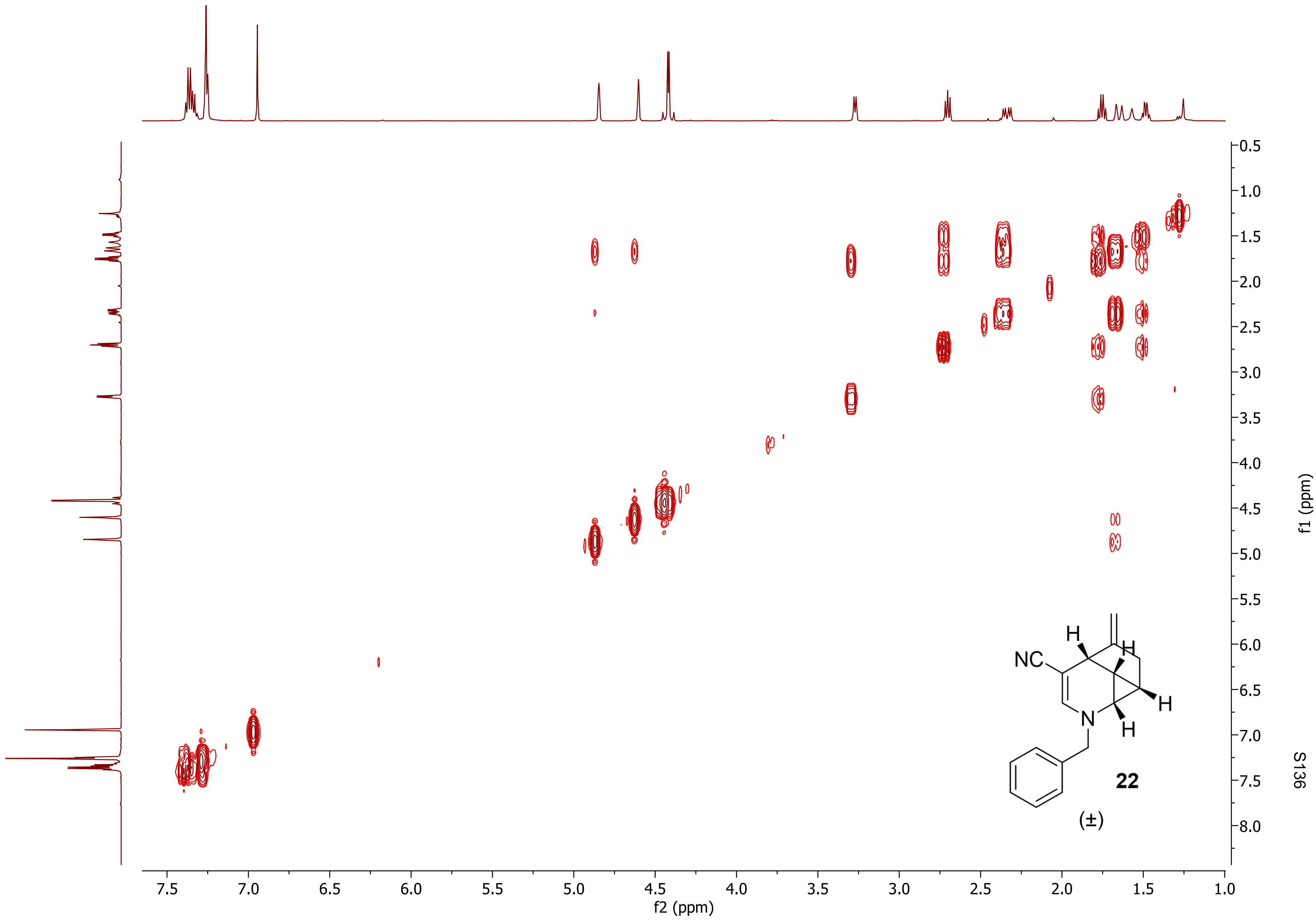

NC
N
H
22
(±)
f1 (ppm)
f2 (ppm)

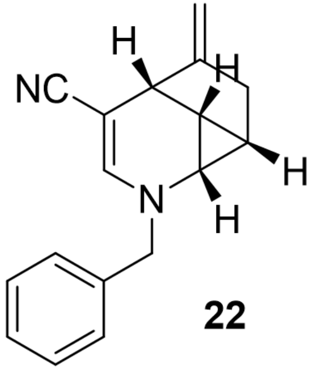

22

(±)

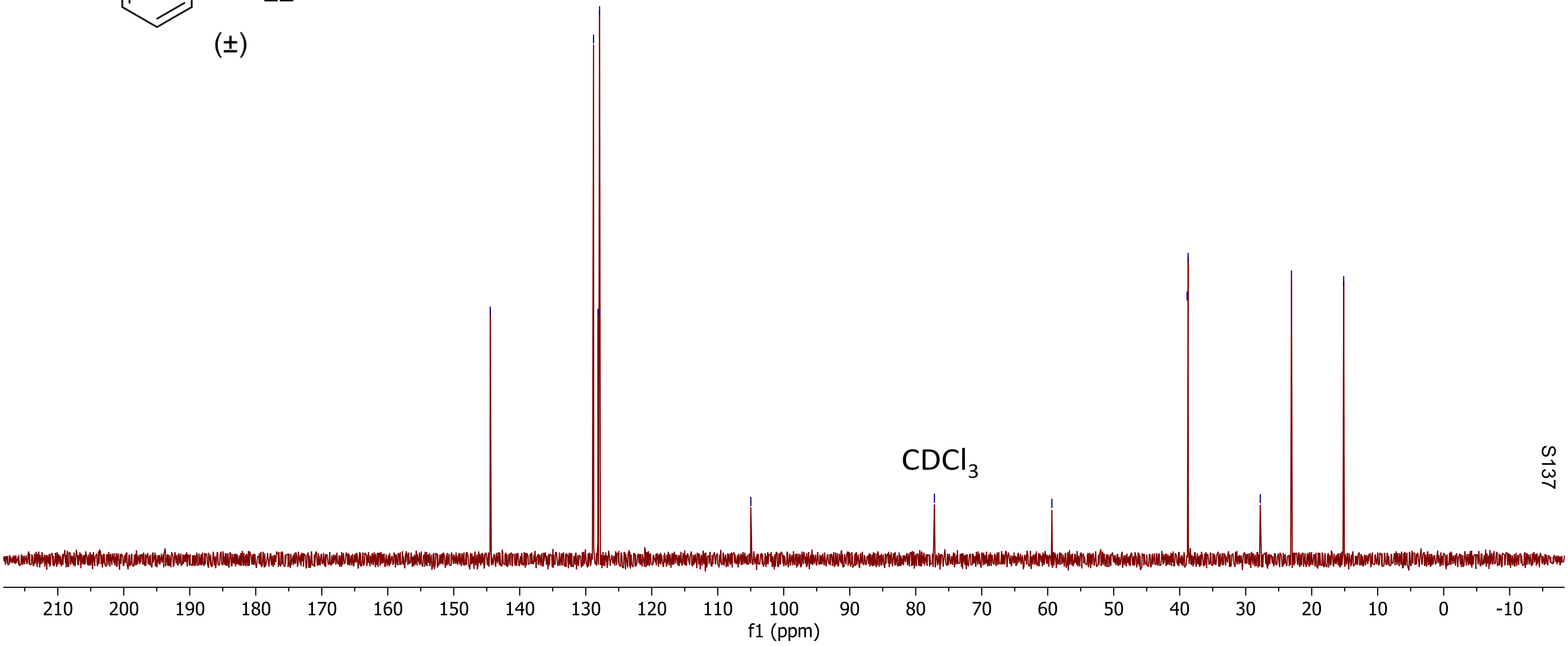
144.46
128.81
128.14
127.90
104.98
77.16
59.36
38.87
38.72
27.79
23.06
15.15
CDCl$_3$
210 200 190 180 170 160 150 140 130 120 110 100 90 80 70 60 50 40 30 20 10 0 -10
f1 (ppm)

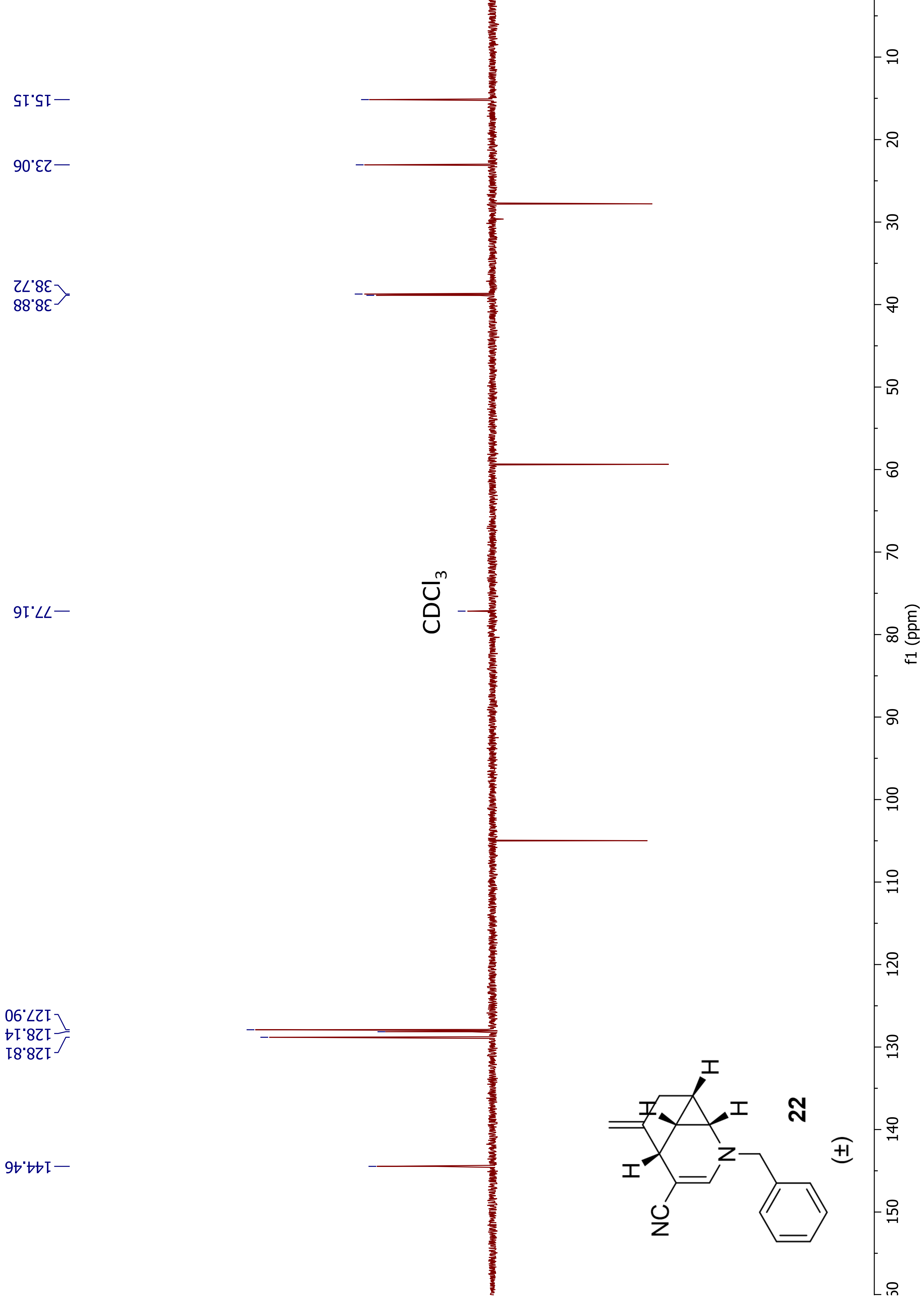
15.15
23.06
38.72
38.88
77.16
127.90
128.14
128.81
144.46
CDCl3
NC
N
H
22
(±)
f1 (ppm)

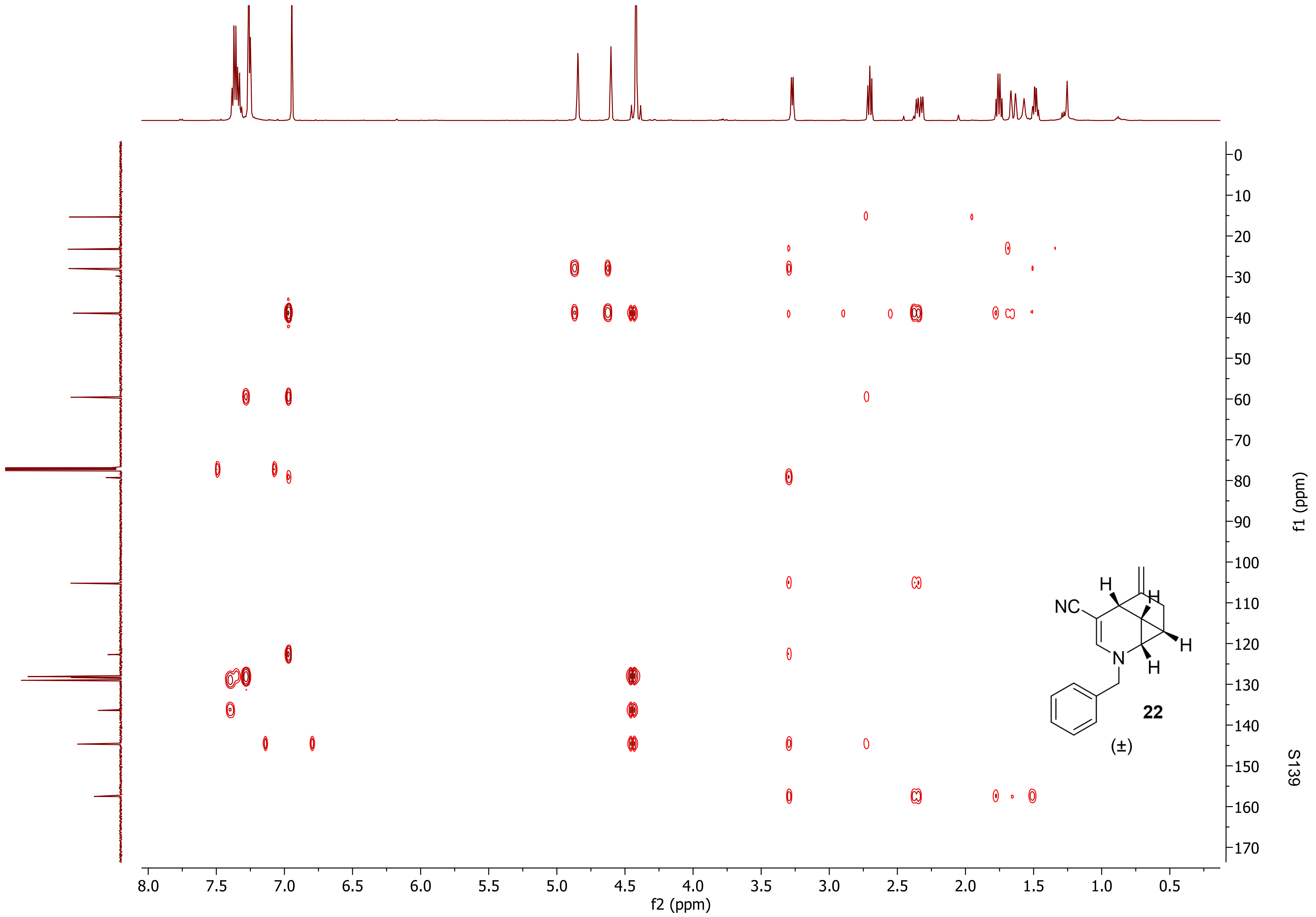

NC
N
H
H
H
H
22
(±)
f1 (ppm)
f2 (ppm)
0
10
20
30
40
50
60
70
80
90
100
110
120
130
140
150
160
170
8.0
7.5
7.0
6.5
6.0
5.5
5.0
4.5
4.0
3.5
3.0
2.5
2.0
1.5
1.0
0.5

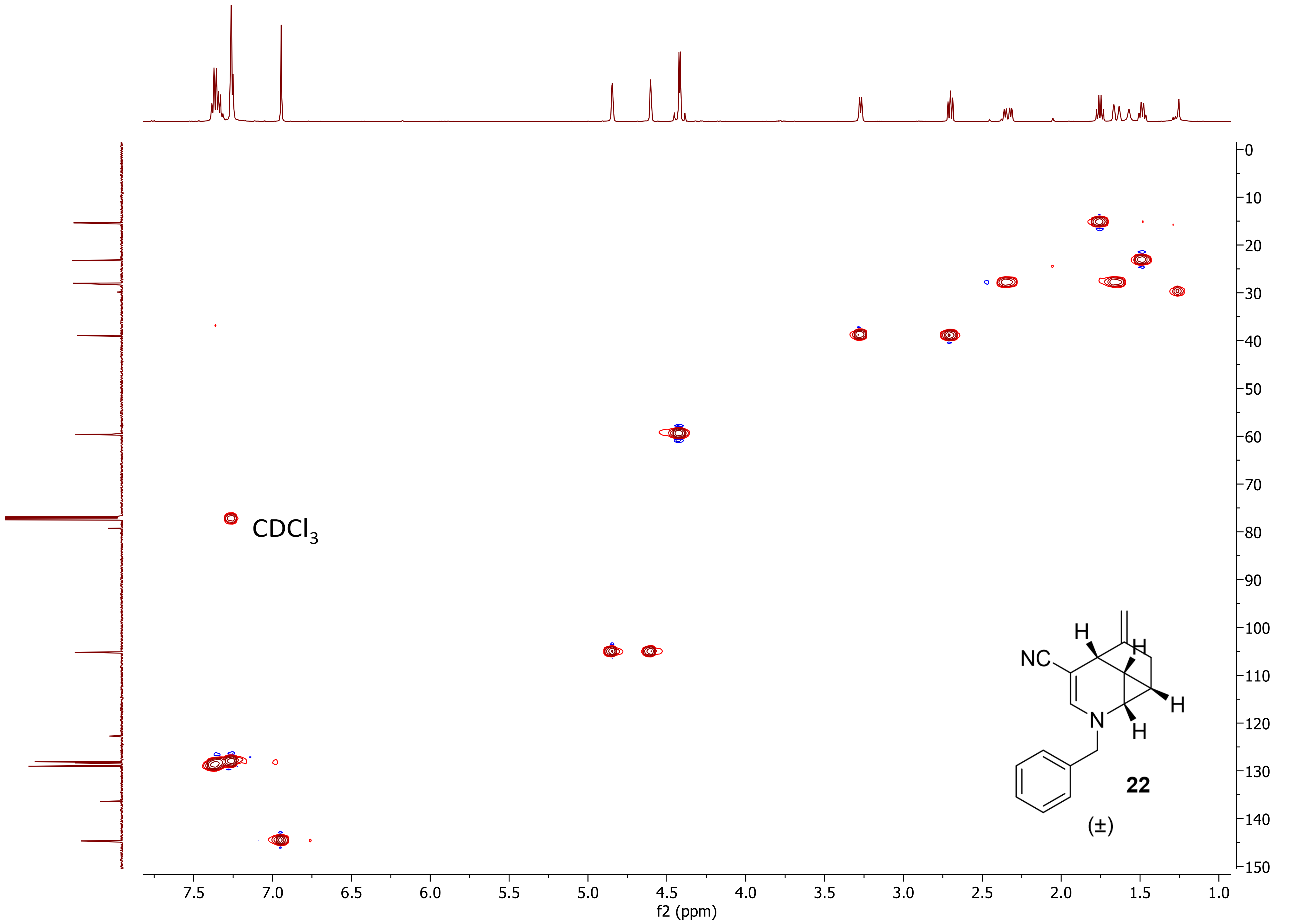
CDCl3
NC
N
H
H
H
H
22
(±)
f2 (ppm)
f1 (ppm)

¹H NMR (601 MHz, Chloroform-*d*)

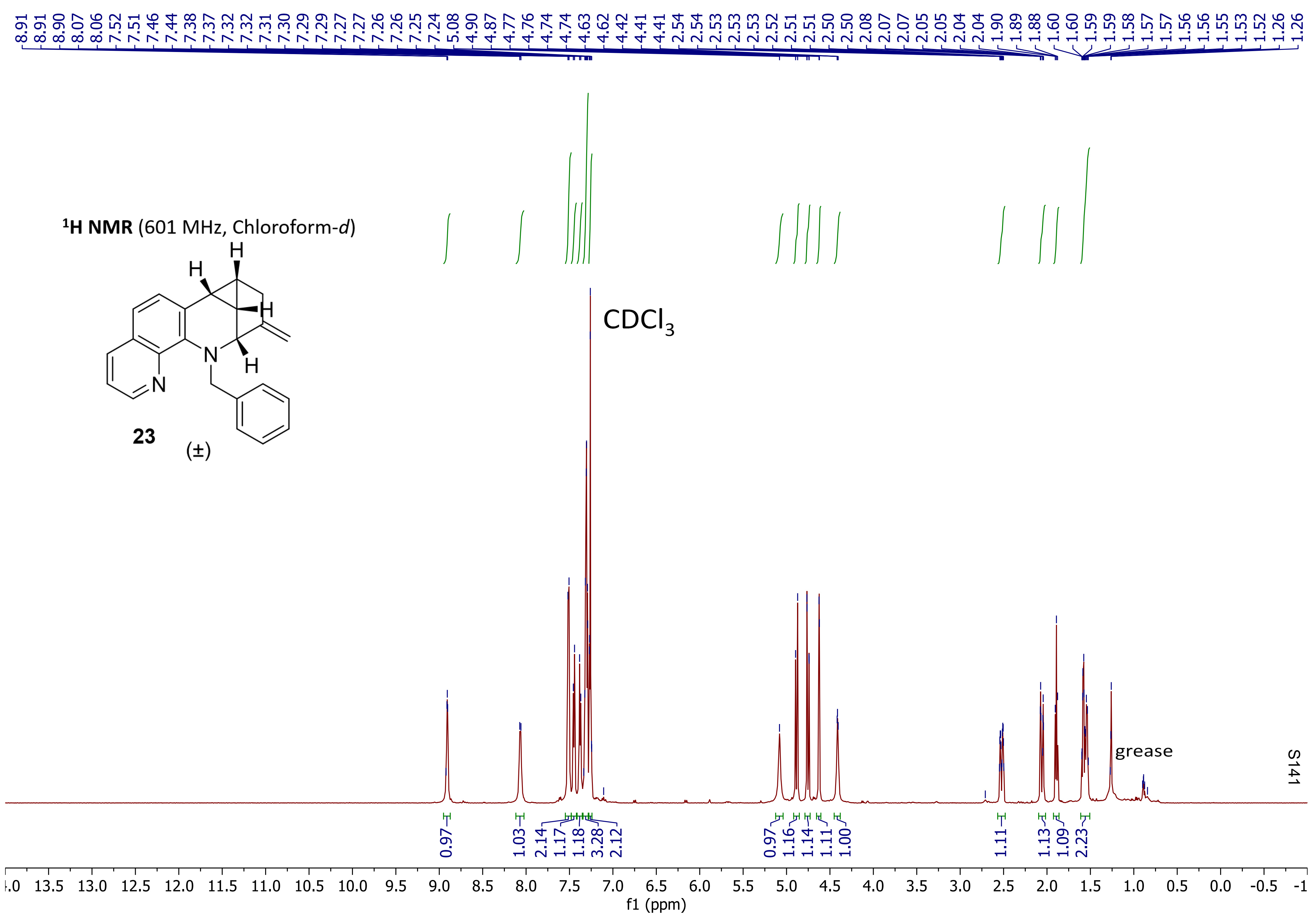

23 (±)

^{13}C NMR (151 MHz, Chloroform-*d*)

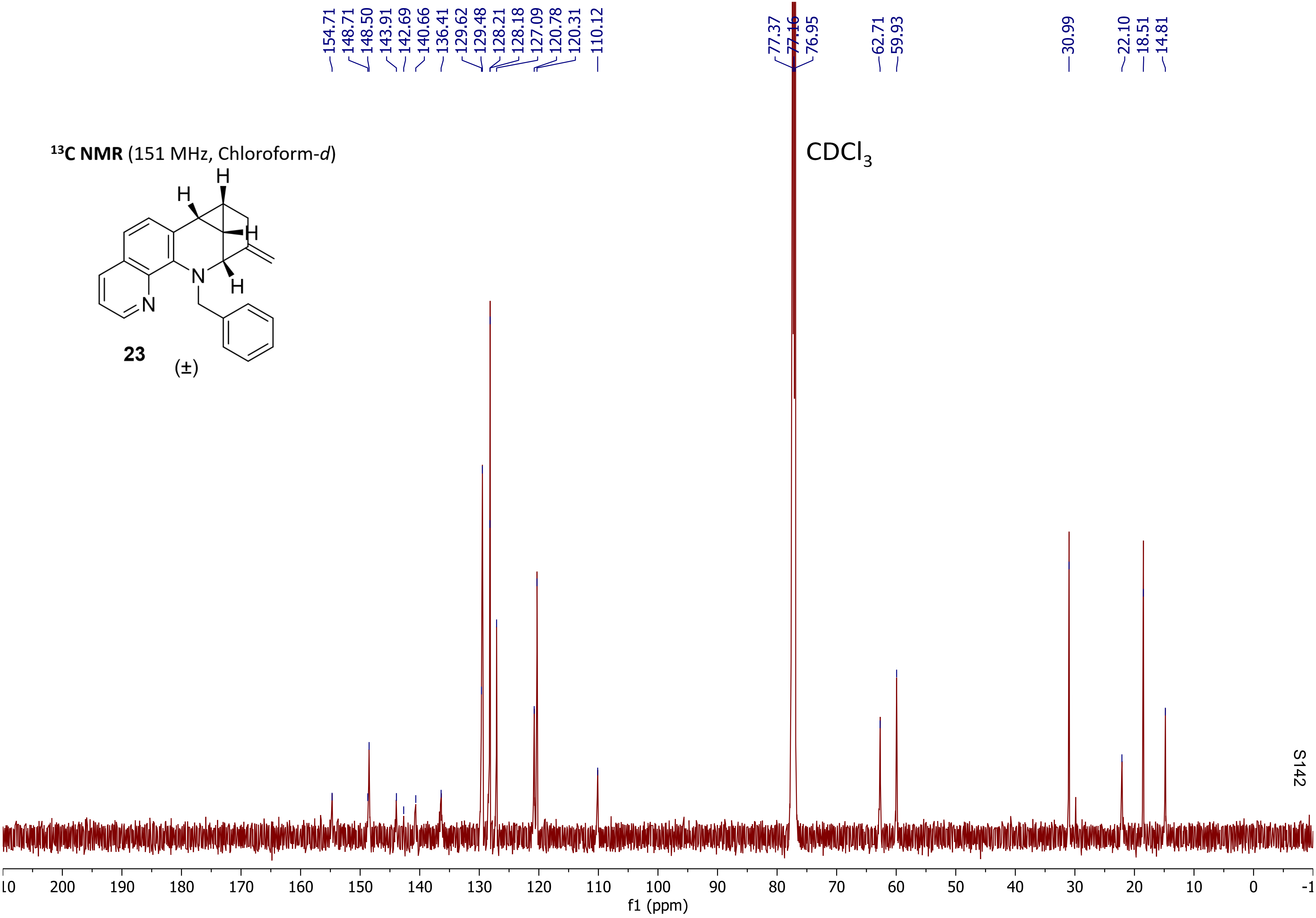

23 (±)

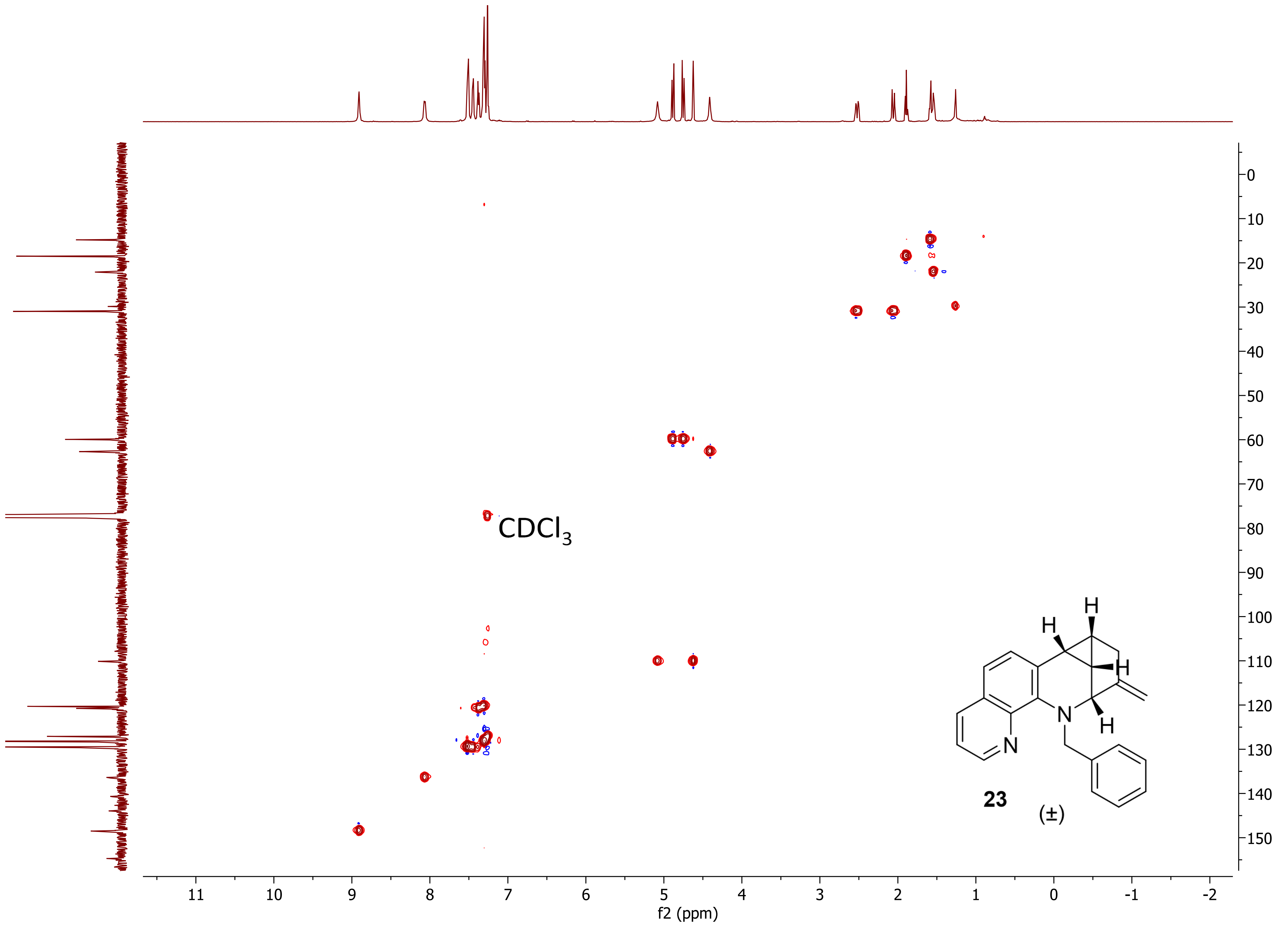
CDCl$_3$
23
(±)
f2 (ppm)
f1 (ppm)

¹H NMR (601 MHz, Chloroform-*d*)

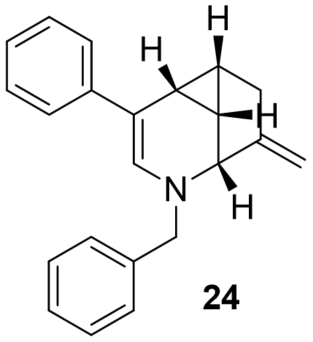

24

(±)

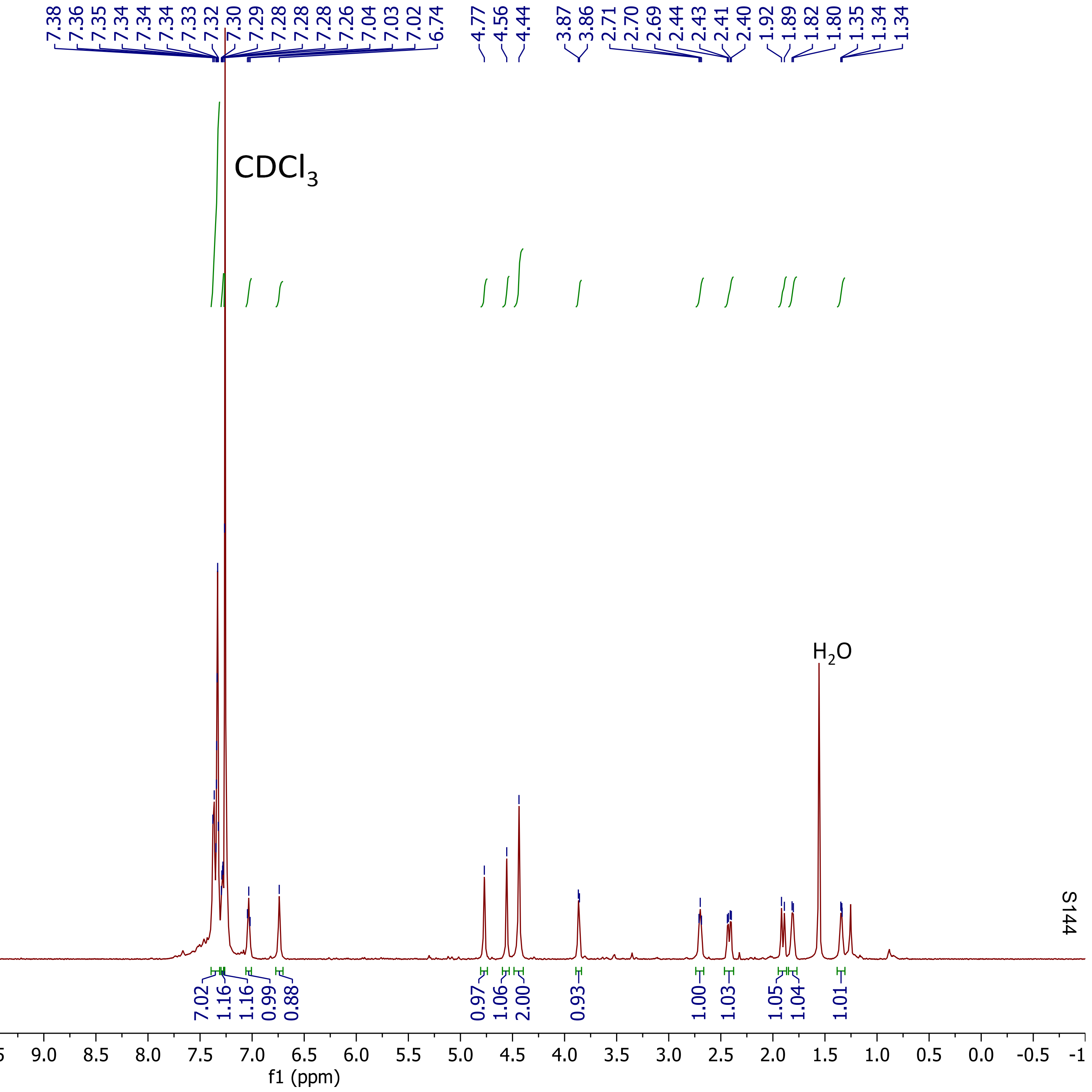

^{13}C NMR (151 MHz, Chloroform-*d*)

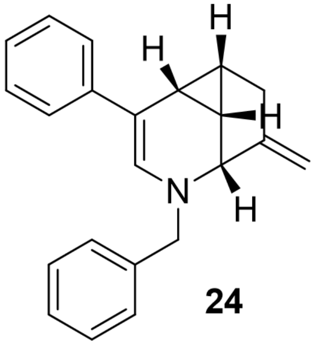

24

(±)

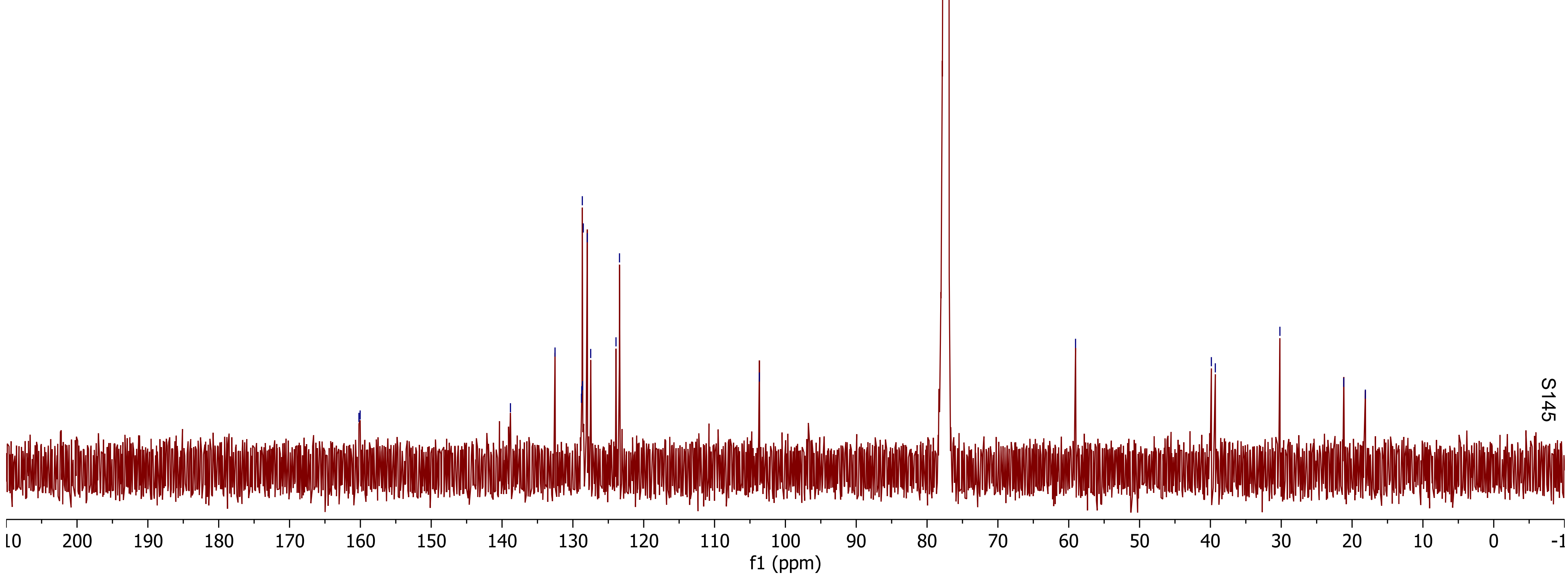

¹H NMR (601 MHz, Chloroform-*d*)

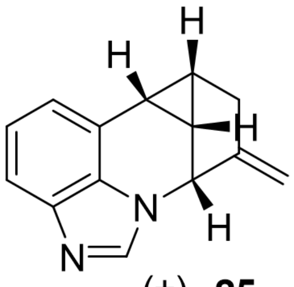

(±) **25**

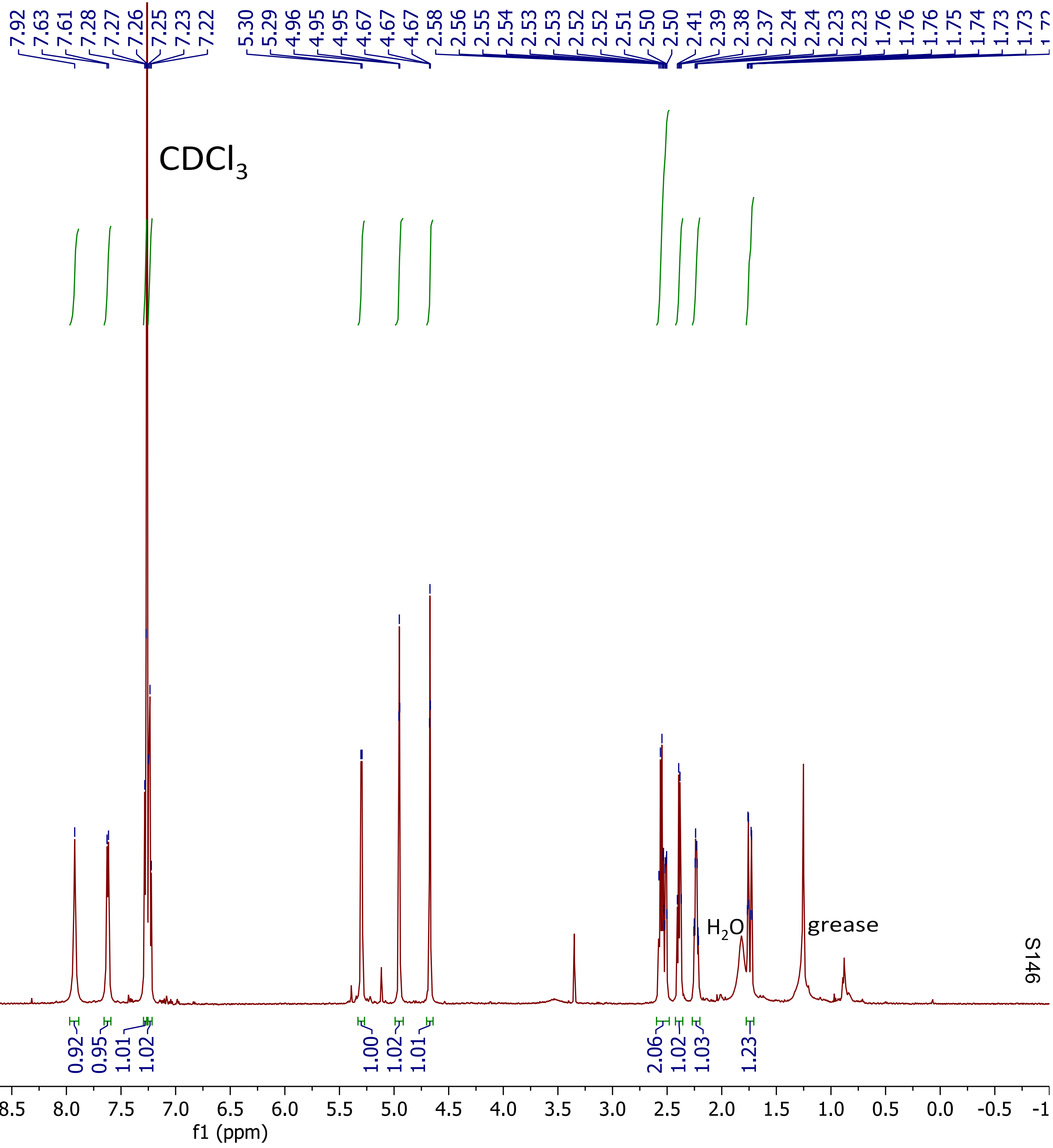

^{13}C NMR (151 MHz, Chloroform-*d*)

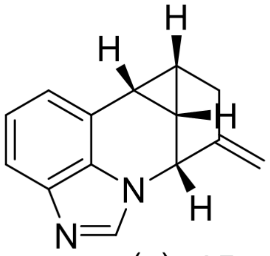

(±) **25**

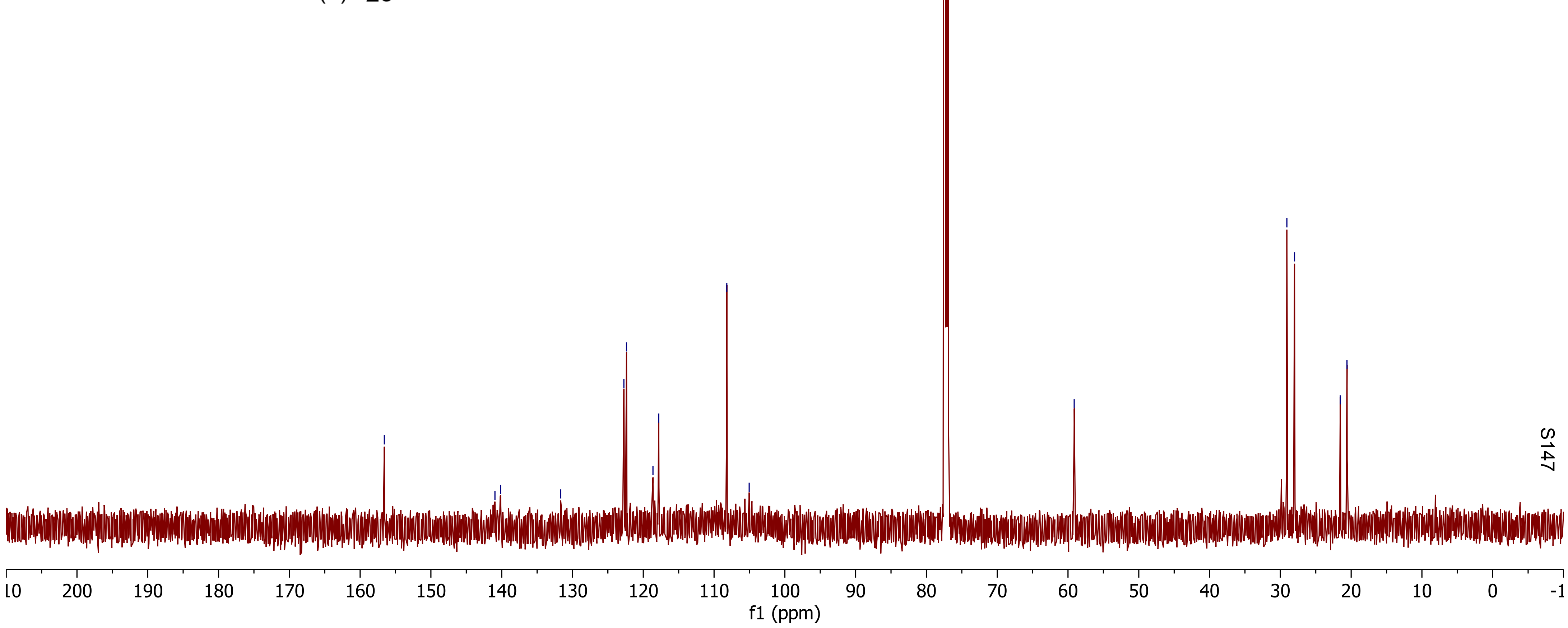

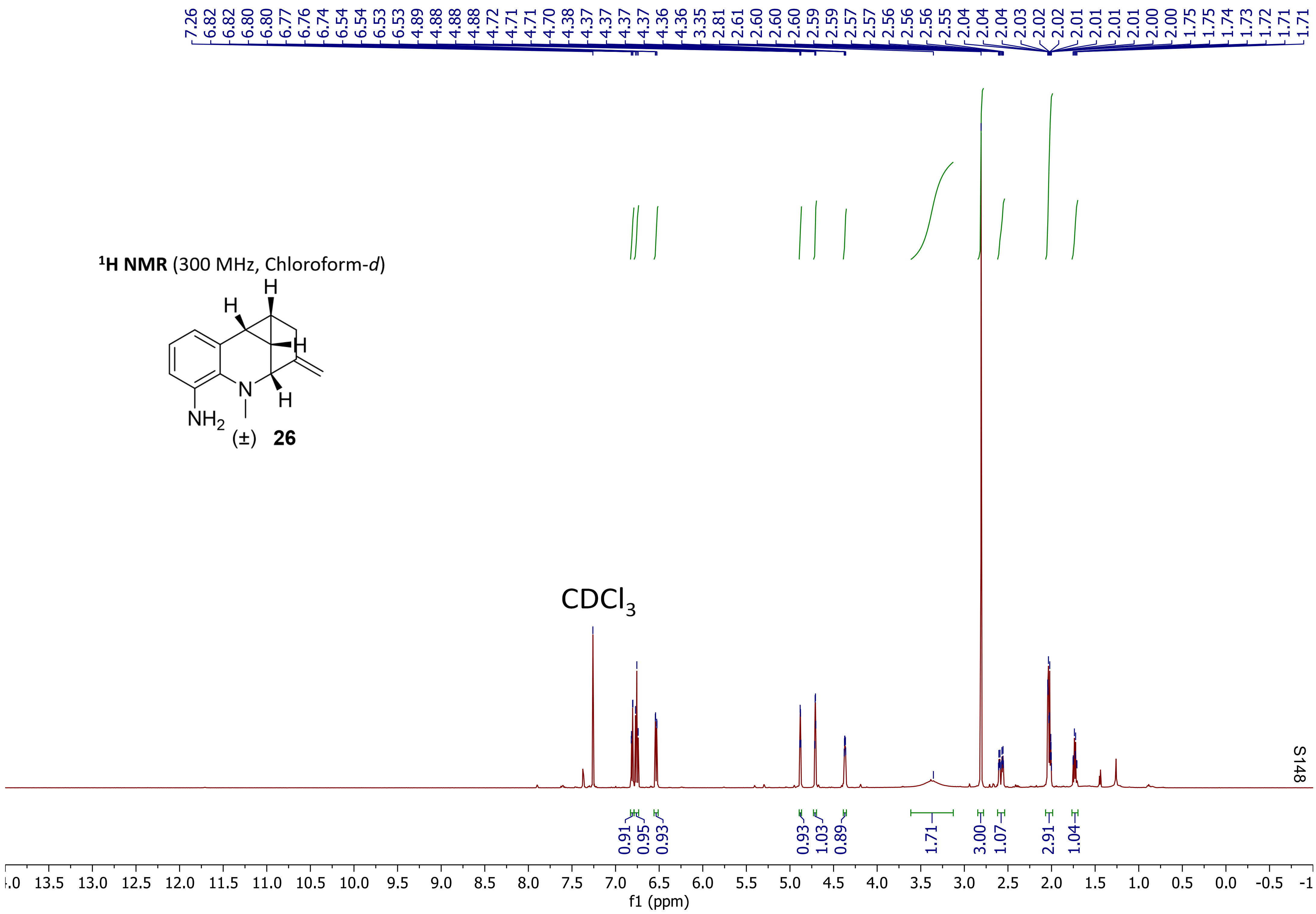
7.26 6.82 6.82 6.80 6.80 6.77 6.76 6.74 6.54 6.54 6.53 6.53 4.89 4.88 4.88 4.88 4.72 4.71 4.71 4.70 4.38 4.37 4.37 4.37 4.37 4.36 4.36 3.35 2.81 2.61 2.60 2.60 2.60 2.59 2.59 2.57 2.57 2.56 2.56 2.56 2.55 2.04 2.04 2.04 2.03 2.02 2.02 2.01 2.01 2.01 2.01 2.00 2.00 1.75 1.75 1.74 1.73 1.72 1.71 1.71
¹H NMR (300 MHz, Chloroform-d)
H
H
H
H
N
NH2
(±) 26
CDCl3
S148
0.91 0.95 0.93 0.93 1.03 0.89 1.71 3.00 1.07 2.91 1.04
14.0 13.5 13.0 12.5 12.0 11.5 11.0 10.5 10.0 9.5 9.0 8.5 8.0 7.5 7.0 6.5 6.0 5.5 5.0 4.5 4.0 3.5 3.0 2.5 2.0 1.5 1.0 0.5 0.0 -0.5 -1
f1 (ppm)

^{13}C NMR (76 MHz, Chloroform-*d*)

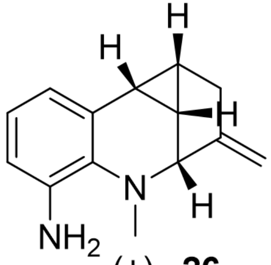

(±) **26**

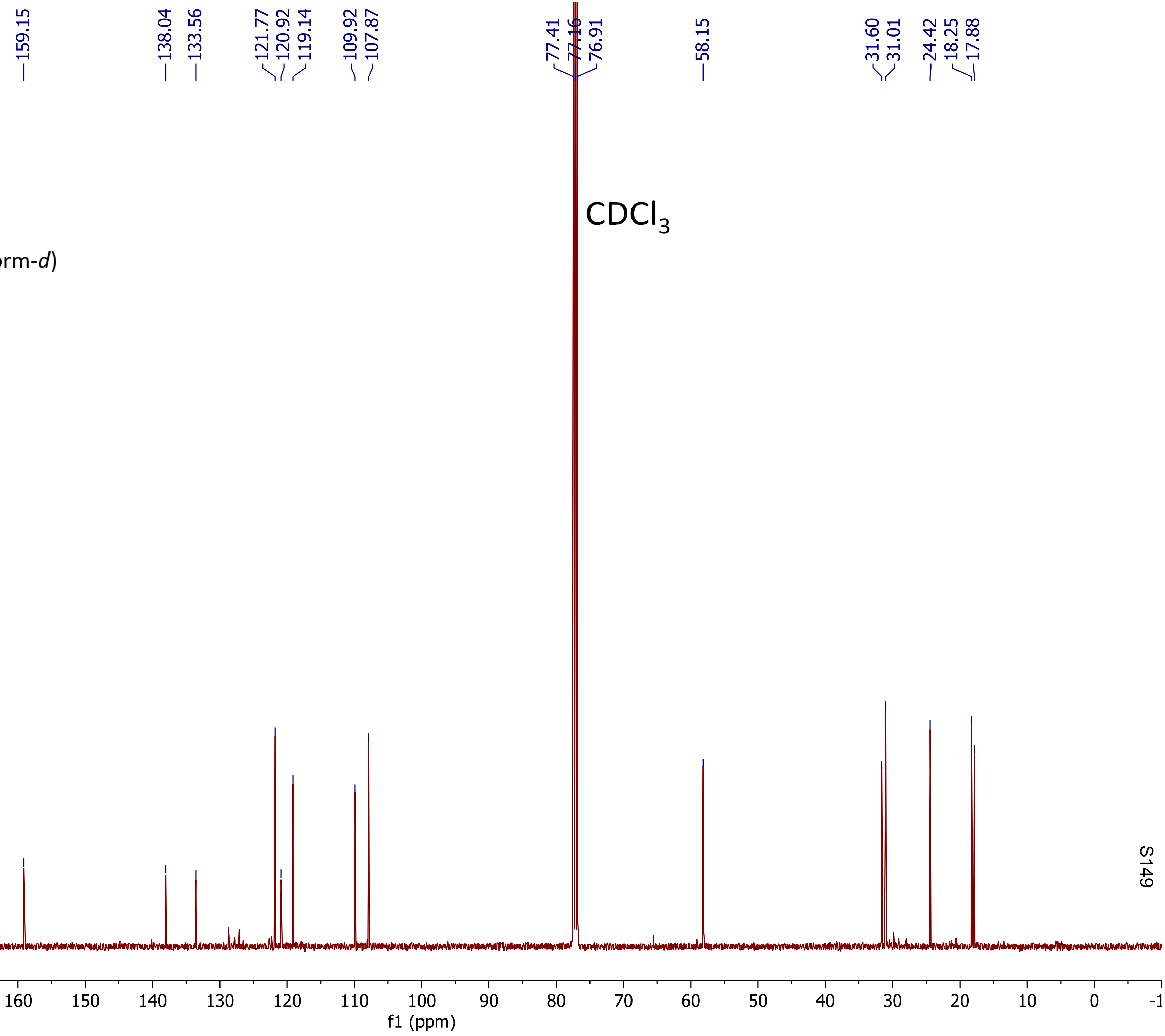

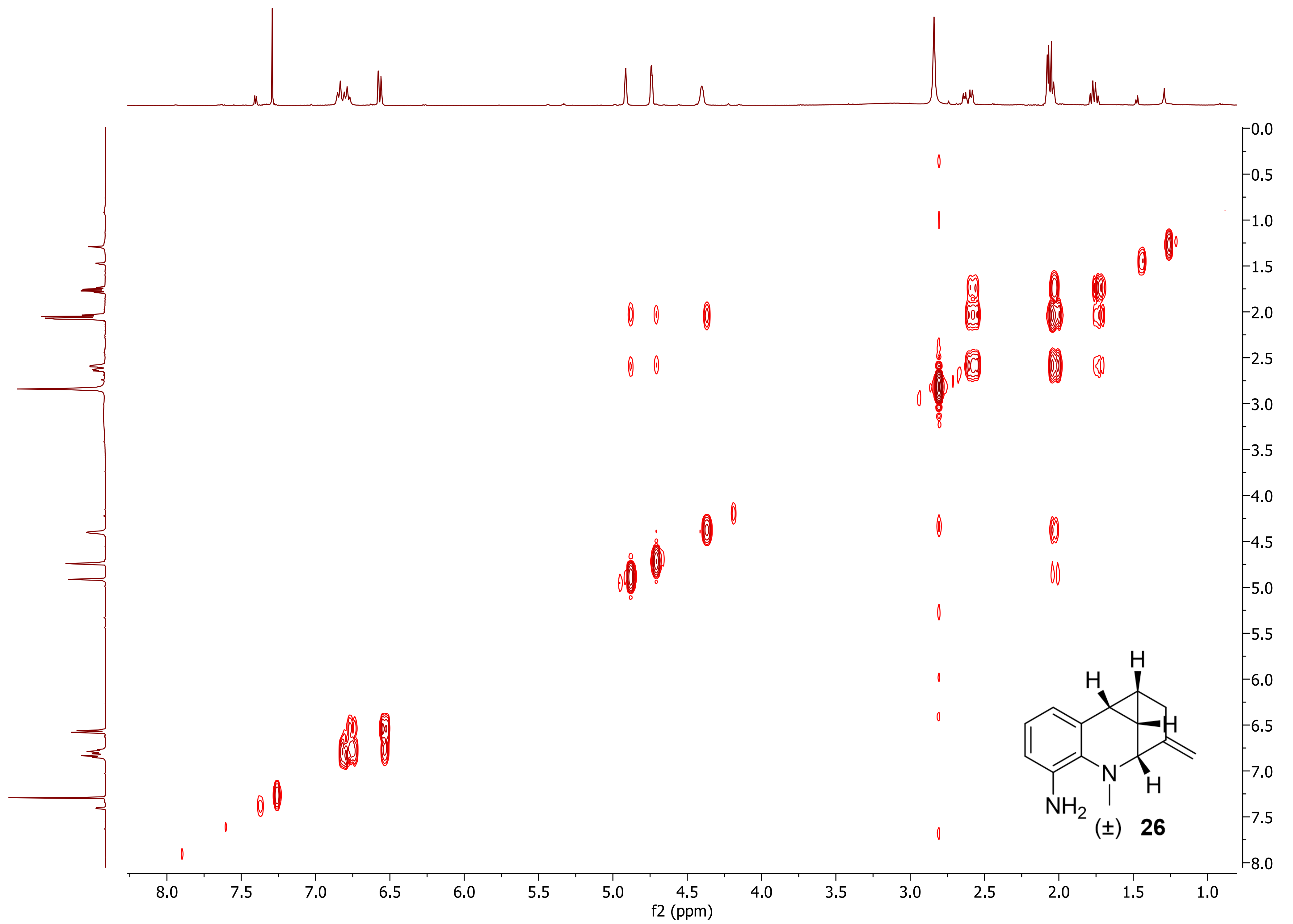

f2 (ppm)
f1 (ppm)
NH2
N
H
(±) 26

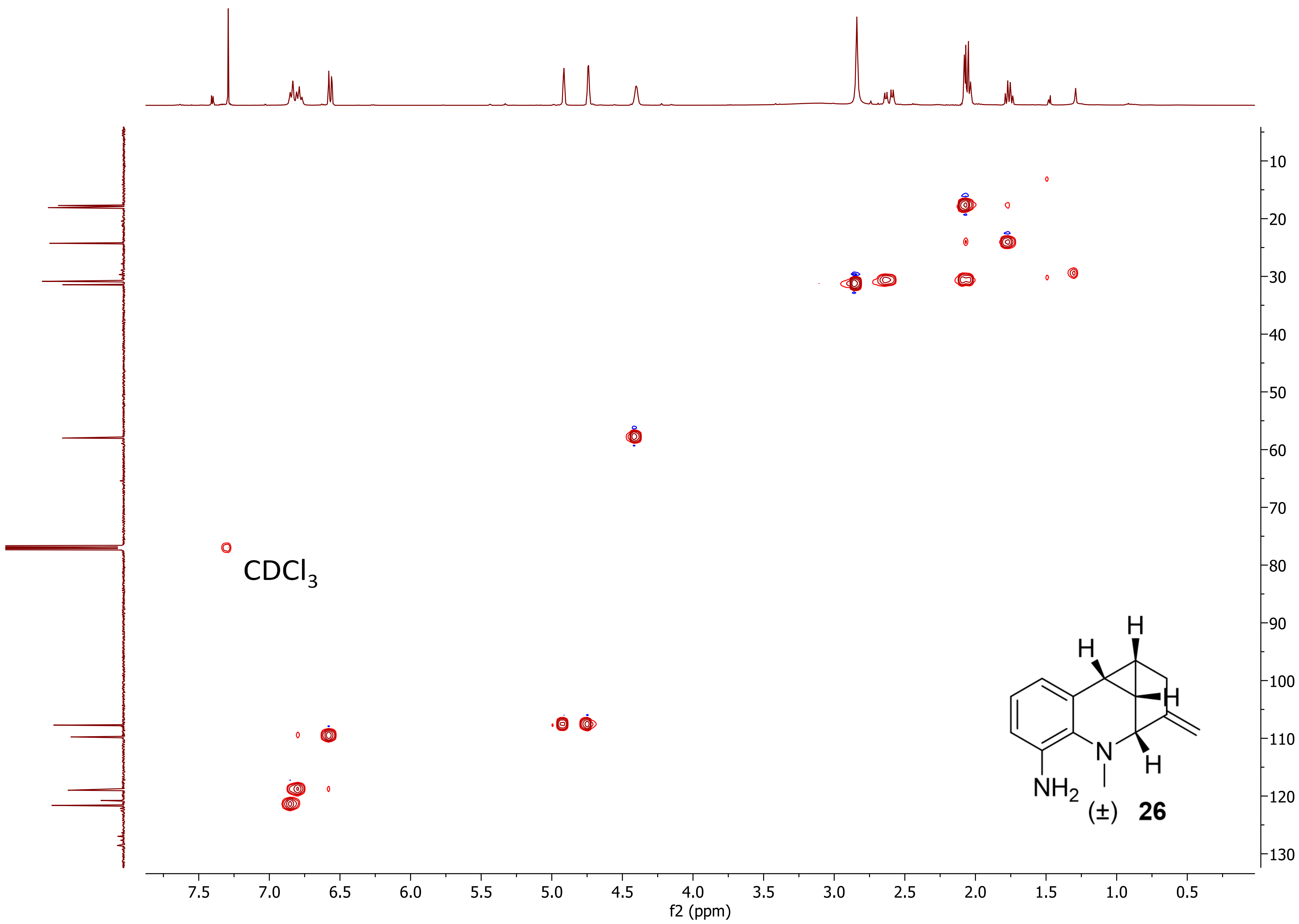

f1 (ppm)
f2 (ppm)
CDCl3
NH2
N
H
(±) 26

¹H NMR (601 MHz, Chloroform-*d*)

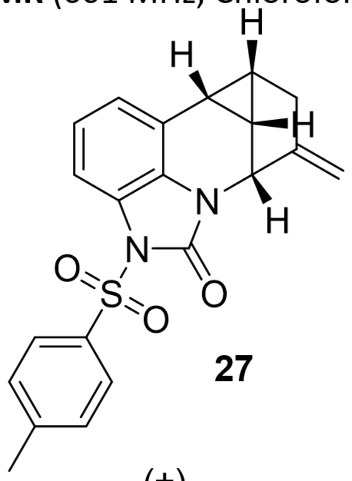

27

(±)

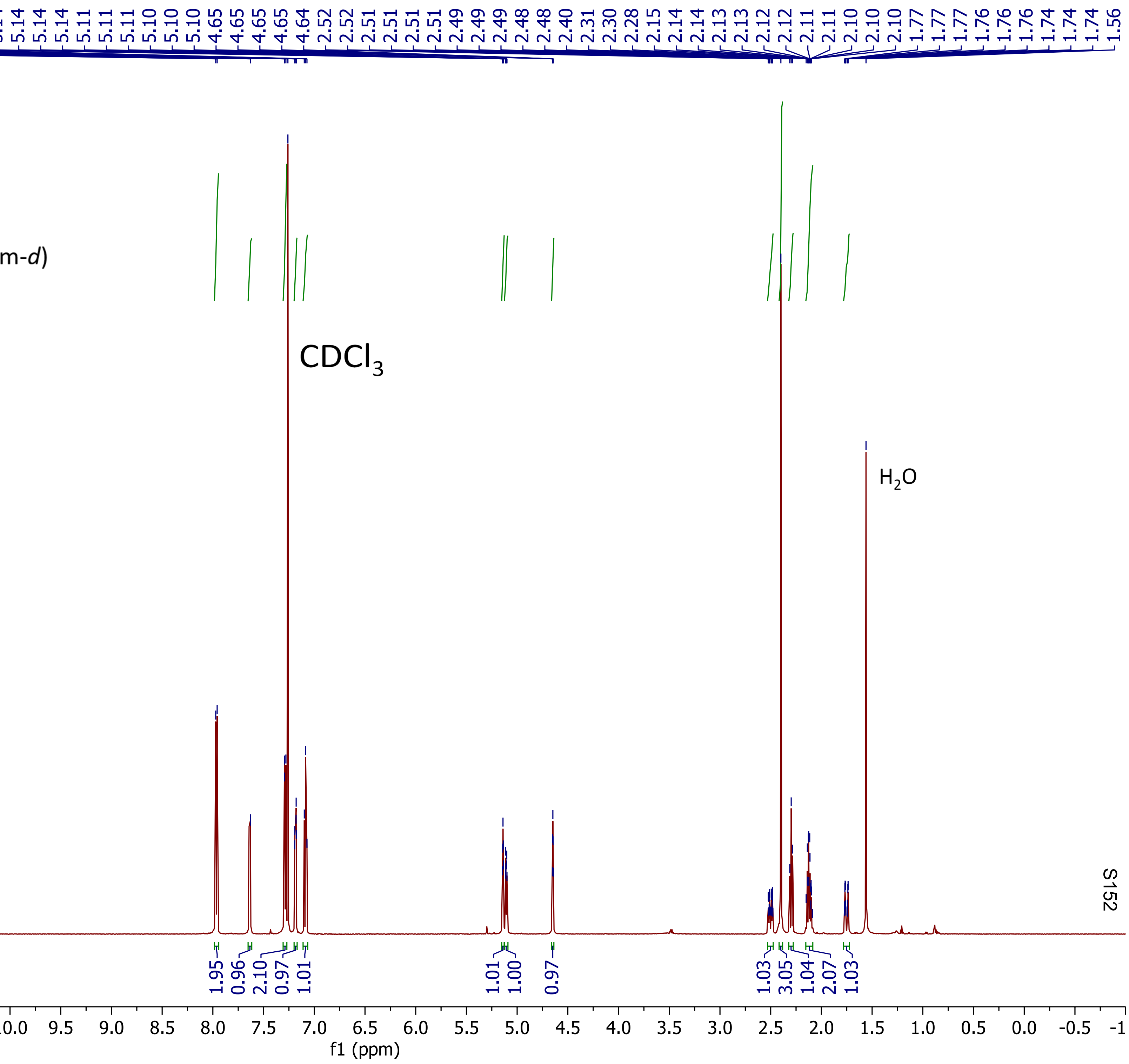

^{13}C NMR (151 MHz, Chloroform-*d*)

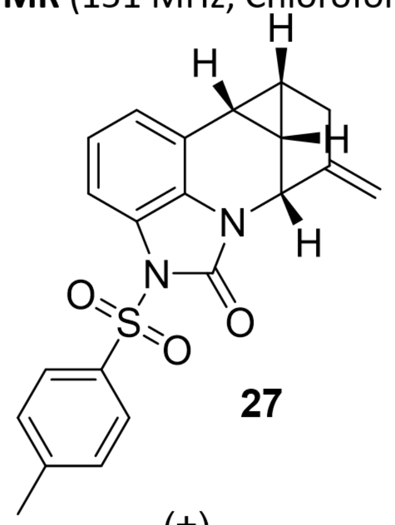

27

(±)

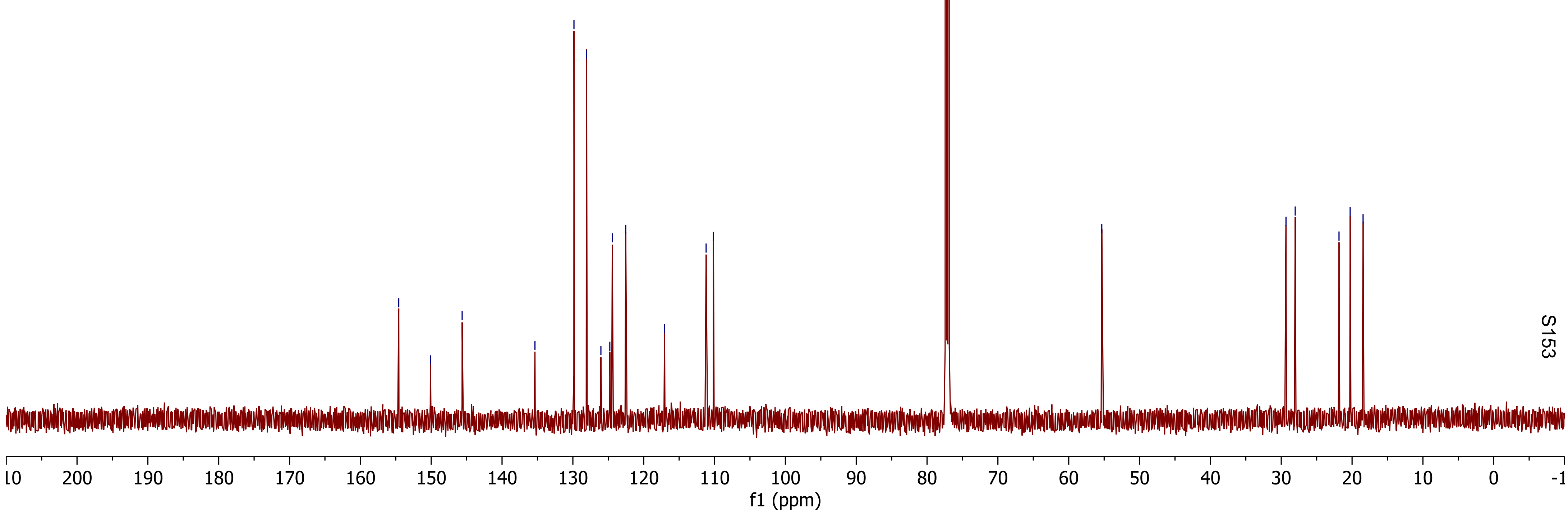

¹H NMR (601 MHz, Chloroform-*d*)

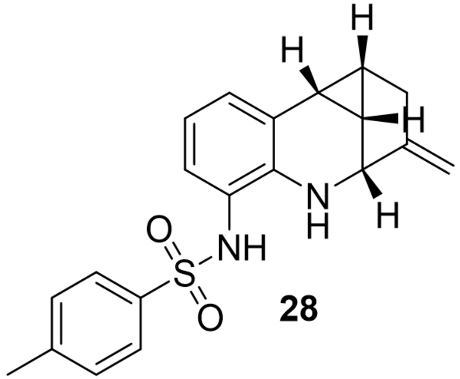

28

(±)

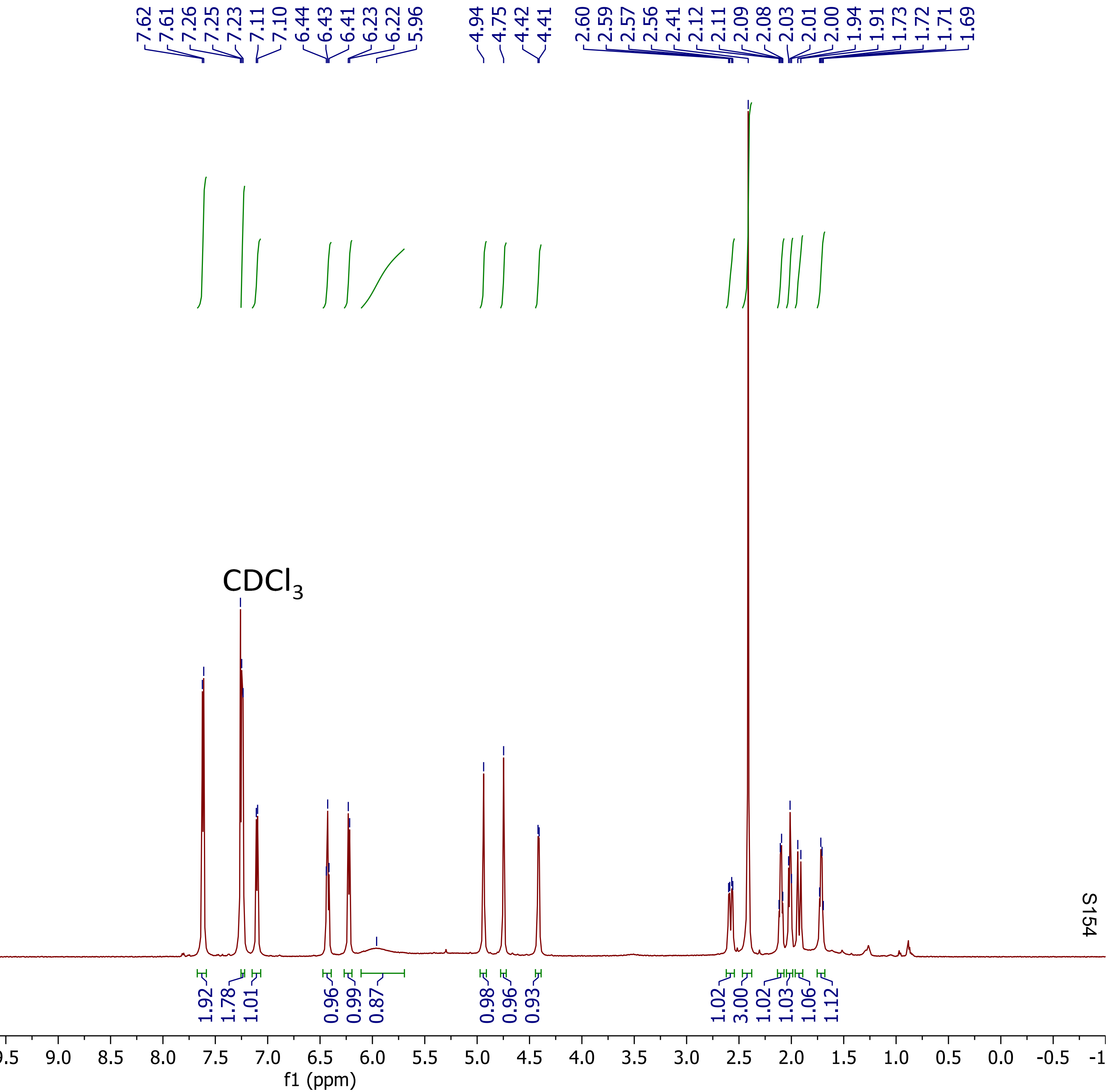

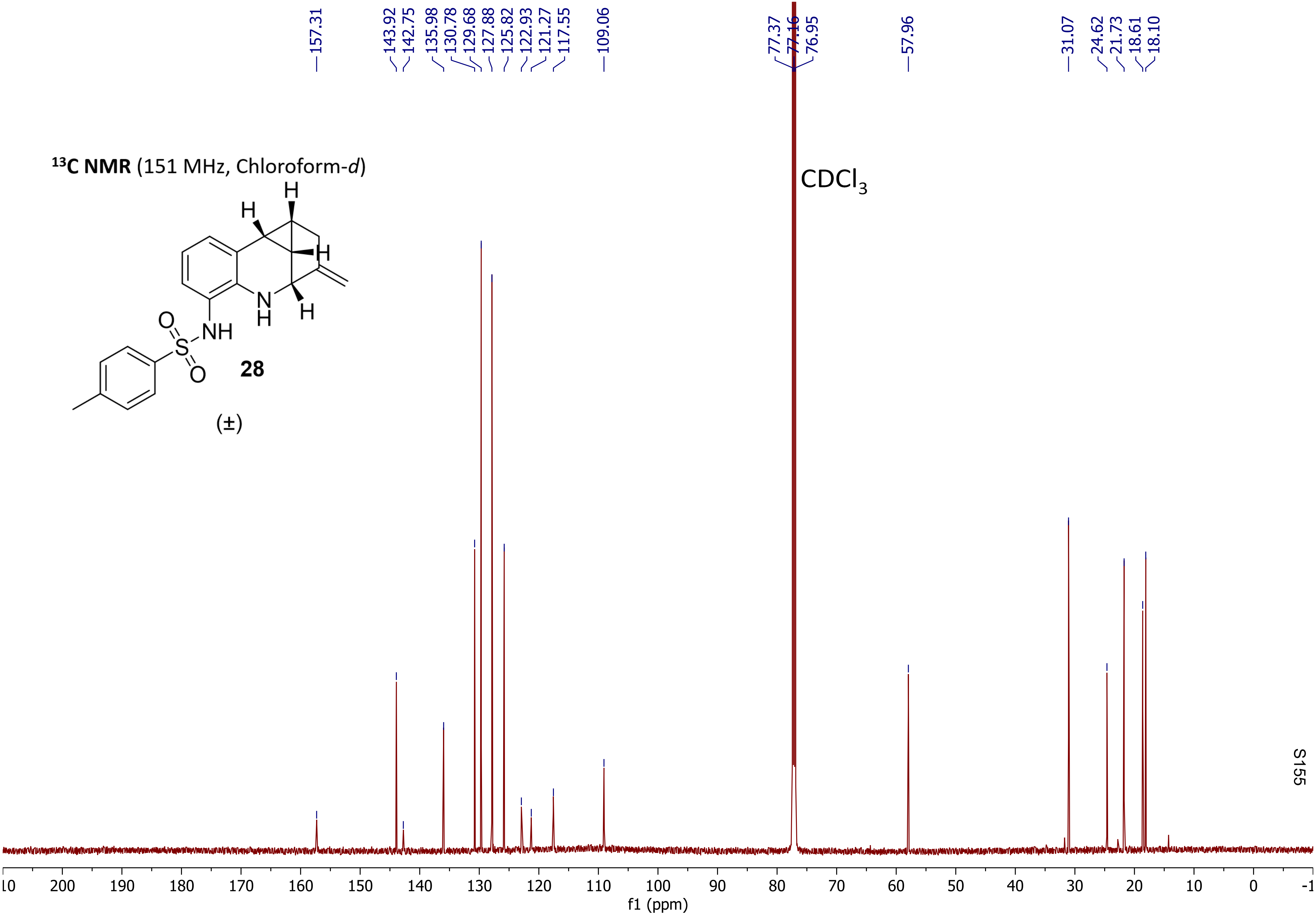
13C NMR (151 MHz, Chloroform-d)
28
(±)
CDCl3
157.31
143.92
142.75
135.98
130.78
129.68
127.88
125.82
122.93
121.27
117.55
109.06
77.37
77.16
76.95
57.96
31.07
24.62
21.73
18.61
18.10
f1 (ppm)

¹H NMR (500 MHz, Chloroform-*d*)

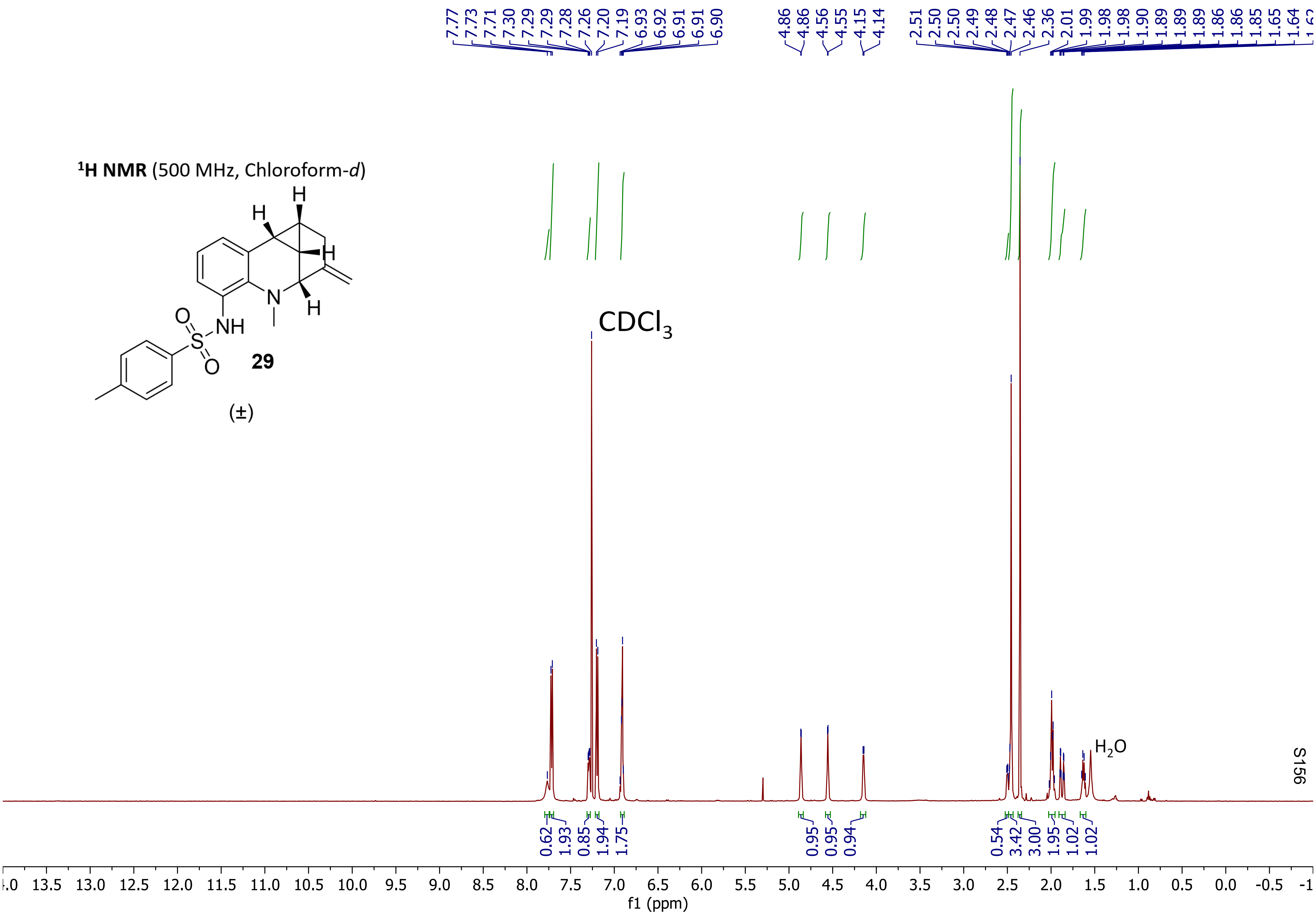

29

(±)

^{13}C NMR (151 MHz, Chloroform-*d*)

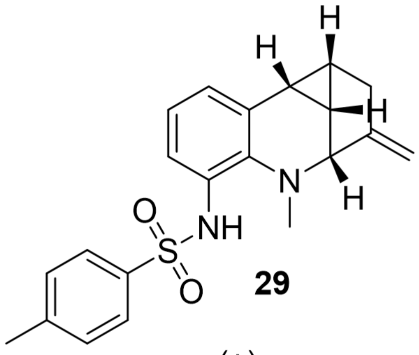

29

(±)

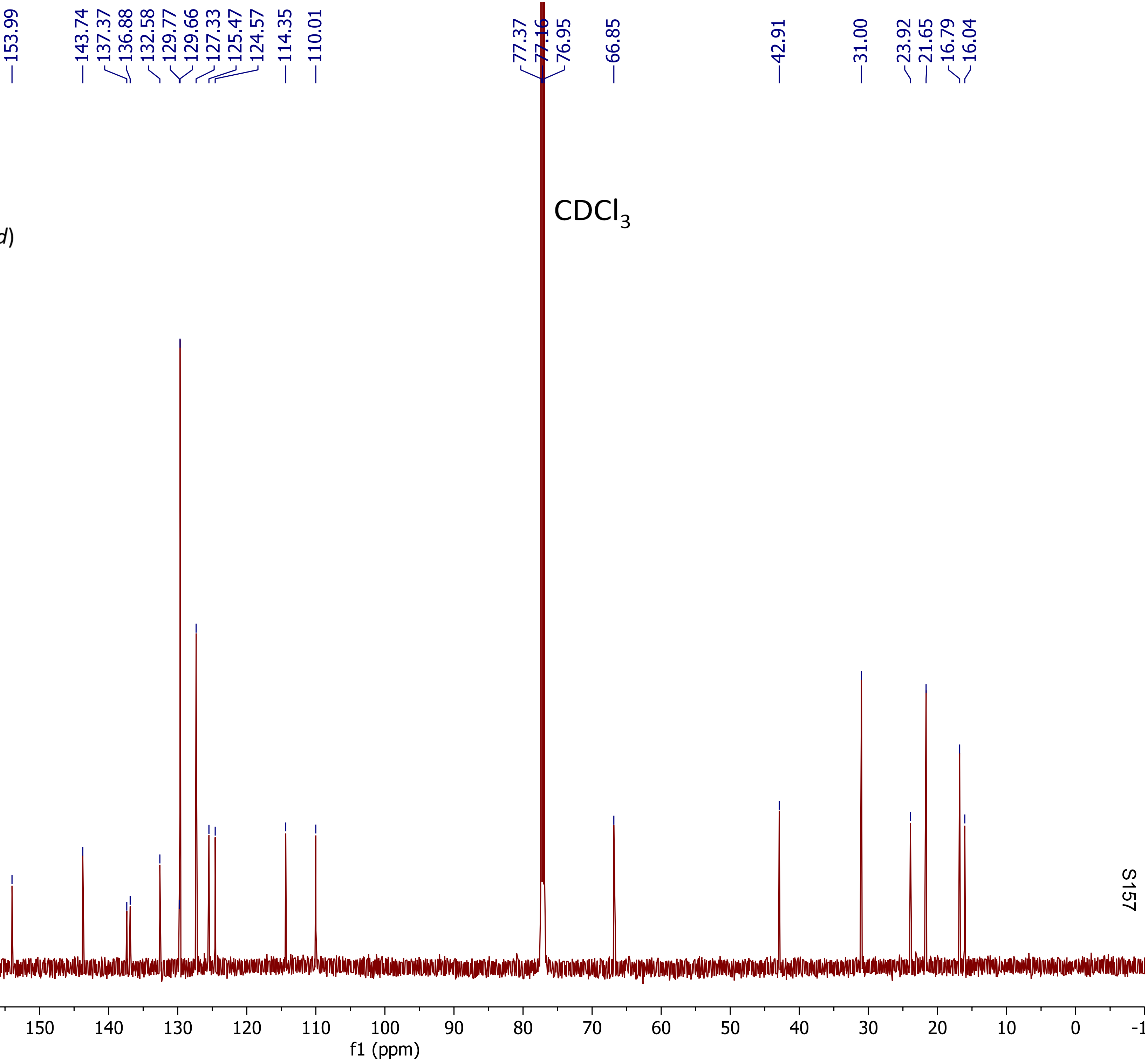

¹H NMR (601 MHz, DMSO-d_6)

30

(±)

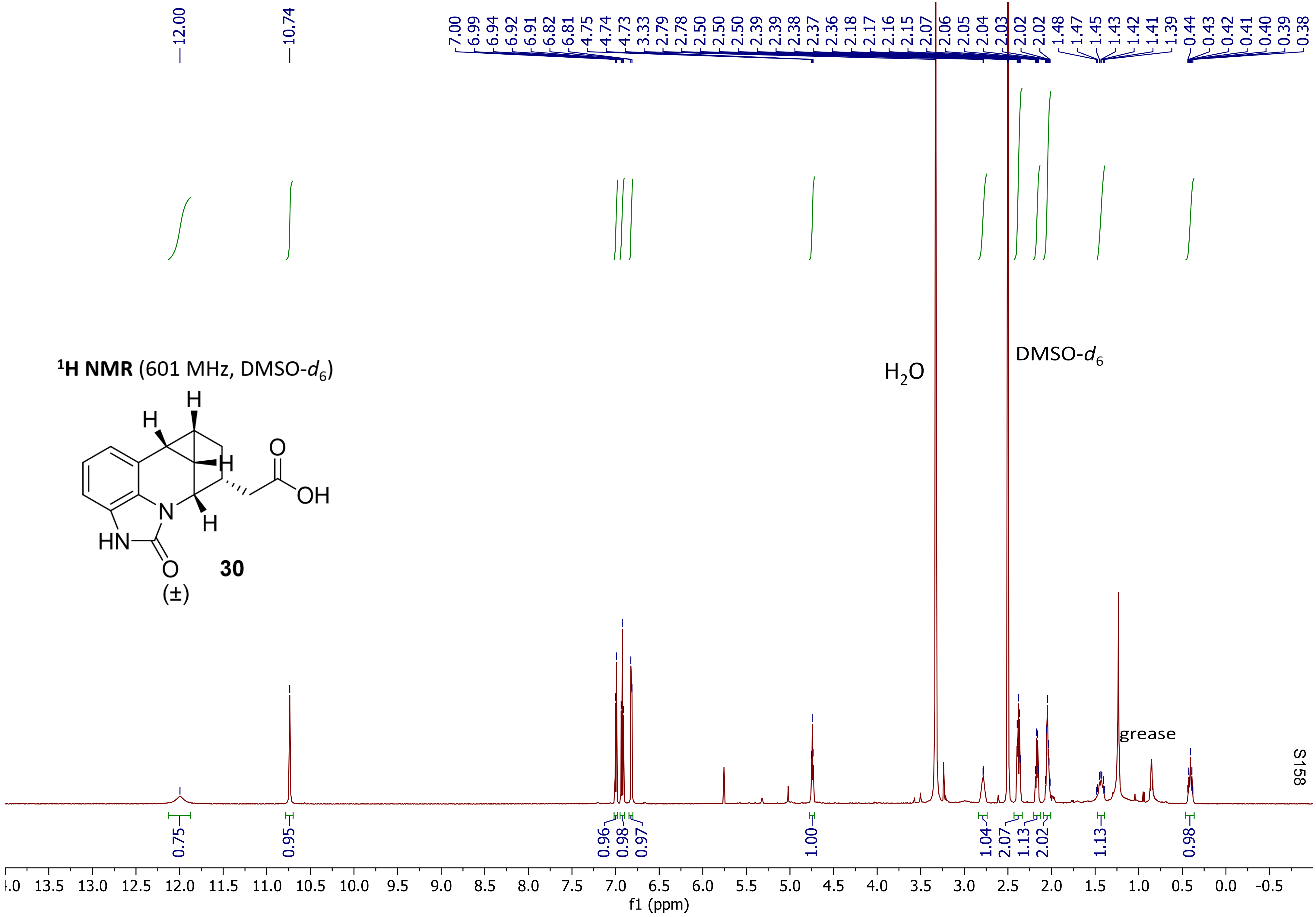

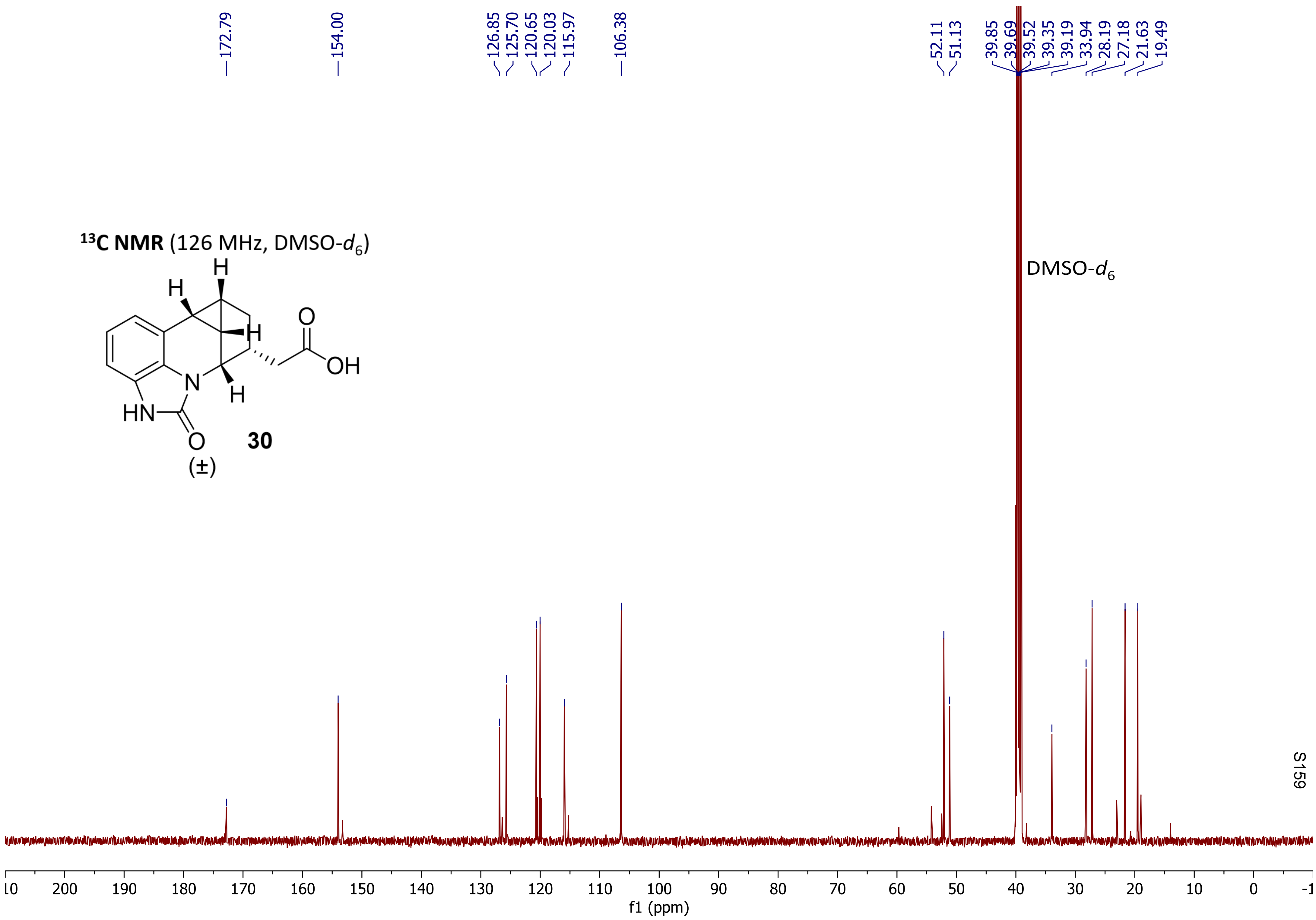

172.79
154.00
126.85
125.70
120.65
120.03
115.97
106.38
52.11
51.13
39.85
39.69
39.52
39.35
39.19
33.94
28.19
27.18
21.63
19.49
DMSO-d_6
^{13}C NMR (126 MHz, DMSO-d_6)
H
H
H
H
N
HN
O
O
OH
(±)
30
f1 (ppm)

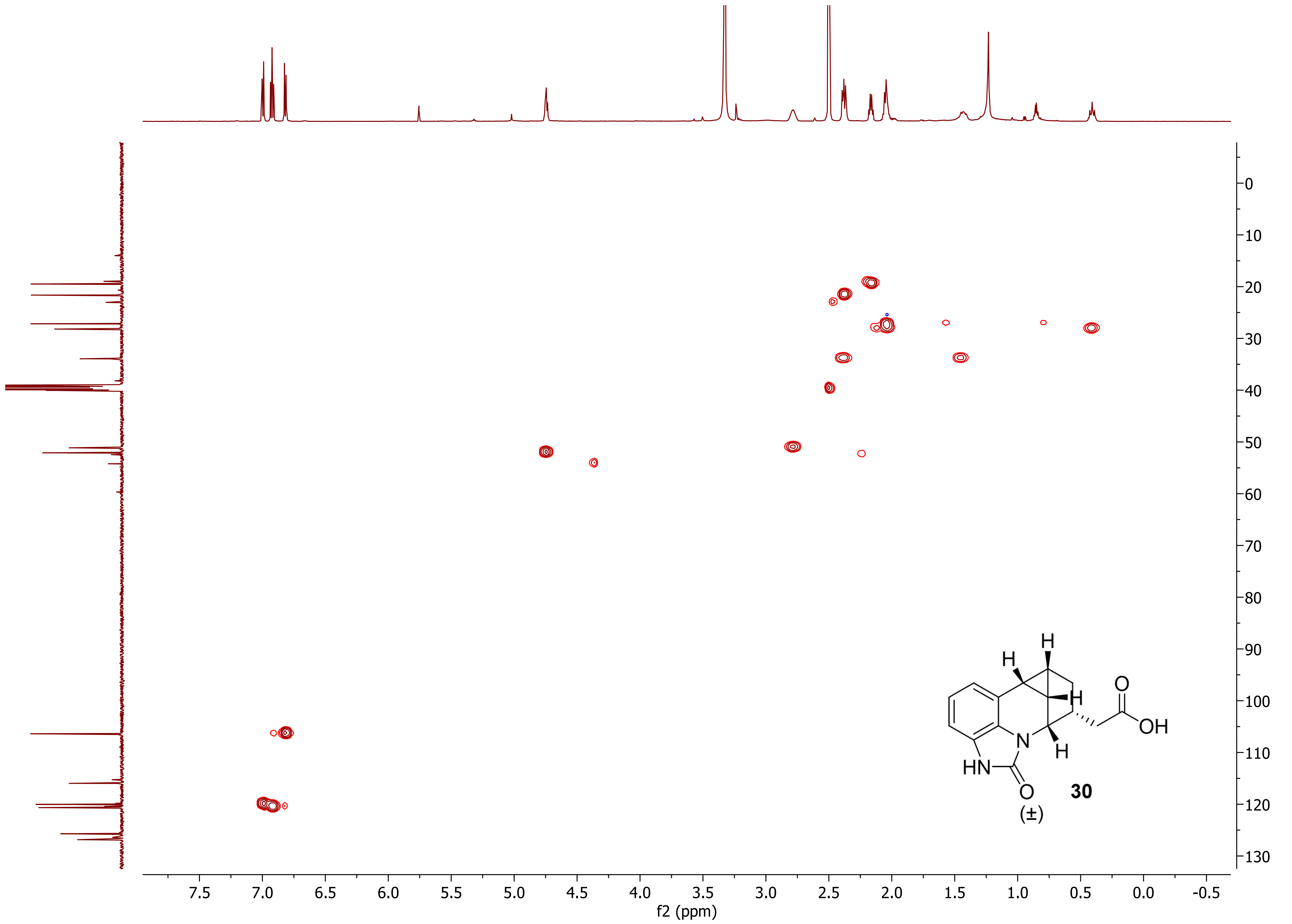

7.5
7.0
6.5
6.0
5.5
5.0
4.5
4.0
3.5
3.0
2.5
2.0
1.5
1.0
0.5
0.0
-0.5
f2 (ppm)
0
10
20
30
40
50
60
70
80
90
100
110
120
130
f1 (ppm)
H
H
H
H
O
OH
N
HN
O
30
(±)

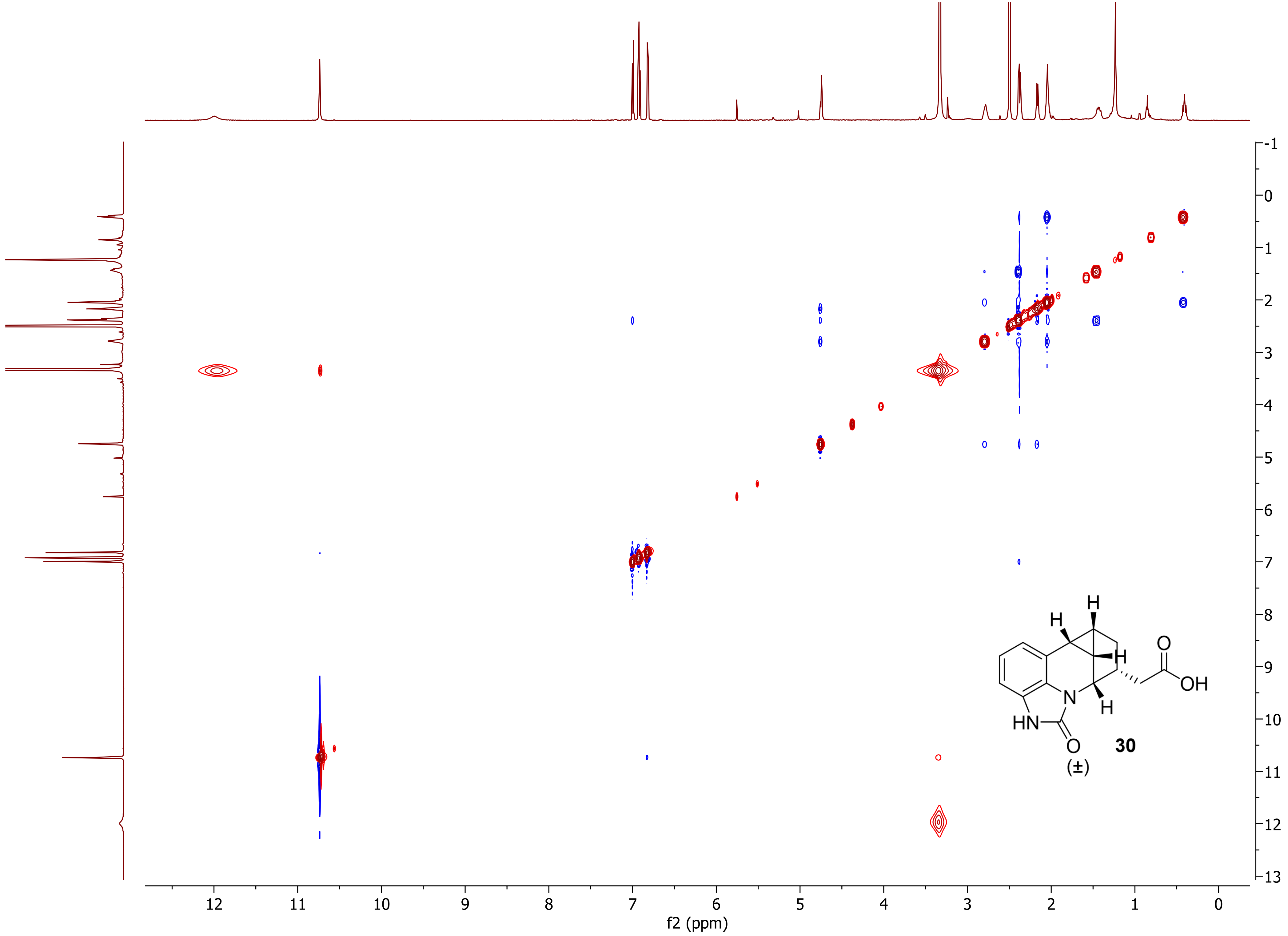

HN
N
O
H
H
H
H
O
OH
30
(±)
f2 (ppm)
f1 (ppm)

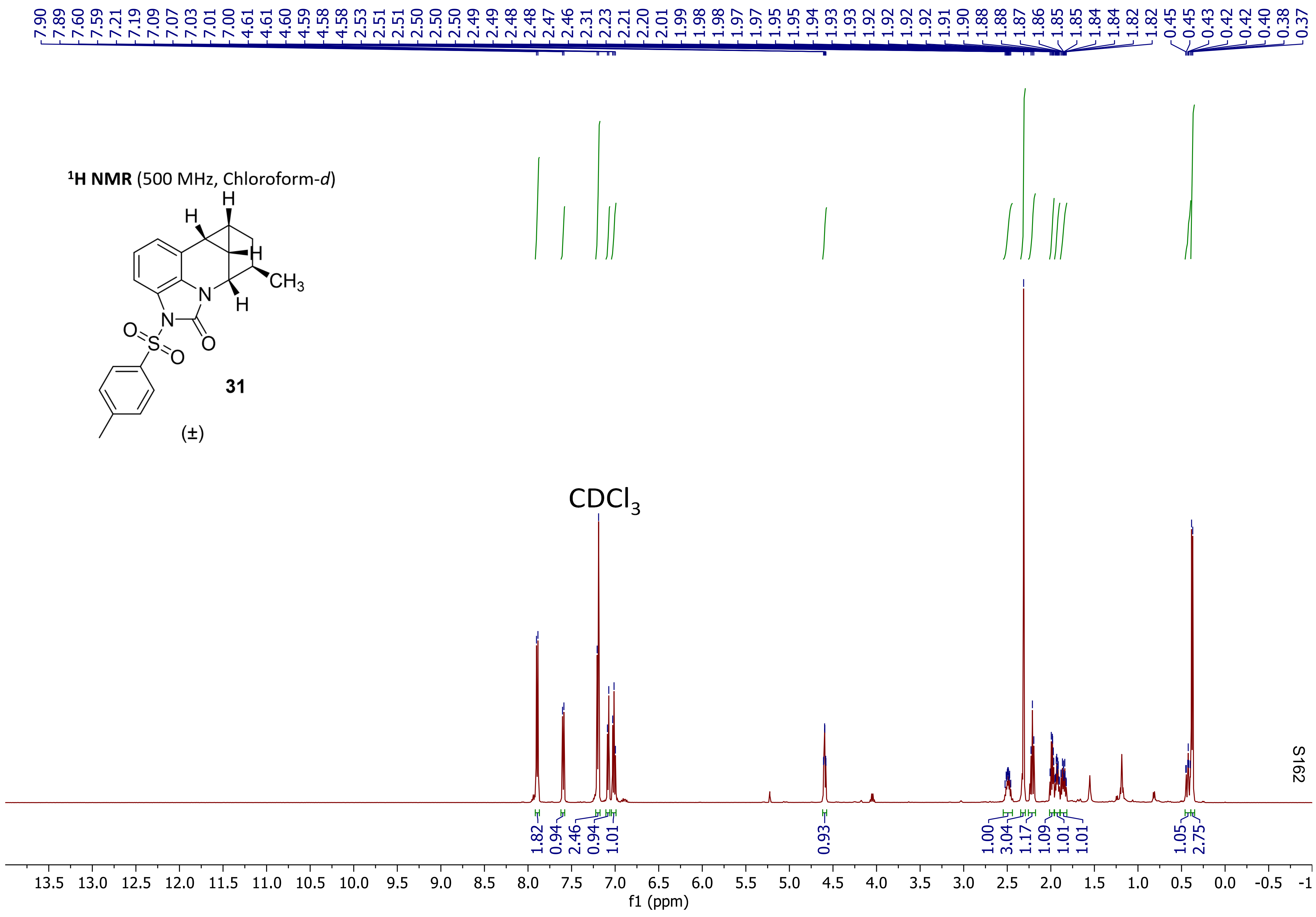

^{1}**H NMR** (500 MHz, Chloroform-*d*)
31
(±)
CDCl$_3$
7.90 7.89 7.60 7.59 7.21 7.19 7.09 7.07 7.03 7.01 7.00 4.61 4.61 4.60 4.59 4.58 4.58 2.53 2.51 2.51 2.50 2.50 2.50 2.49 2.49 2.48 2.48 2.47 2.46 2.31 2.23 2.21 2.20 2.01 1.99 1.98 1.98 1.97 1.97 1.95 1.95 1.94 1.93 1.93 1.92 1.92 1.92 1.92 1.91 1.90 1.88 1.88 1.87 1.86 1.85 1.85 1.84 1.84 1.82 1.82 0.45 0.45 0.43 0.42 0.42 0.40 0.38 0.37
1.82 0.94 2.46 0.94 1.01 0.93 1.00 3.04 1.17 1.09 1.01 1.01 1.05 2.75
13.5 13.0 12.5 12.0 11.5 11.0 10.5 10.0 9.5 9.0 8.5 8.0 7.5 7.0 6.5 6.0 5.5 5.0 4.5 4.0 3.5 3.0 2.5 2.0 1.5 1.0 0.5 0.0 -0.5 -1
f1 (ppm)

^{13}C NMR (151 MHz, Chloroform-*d*)

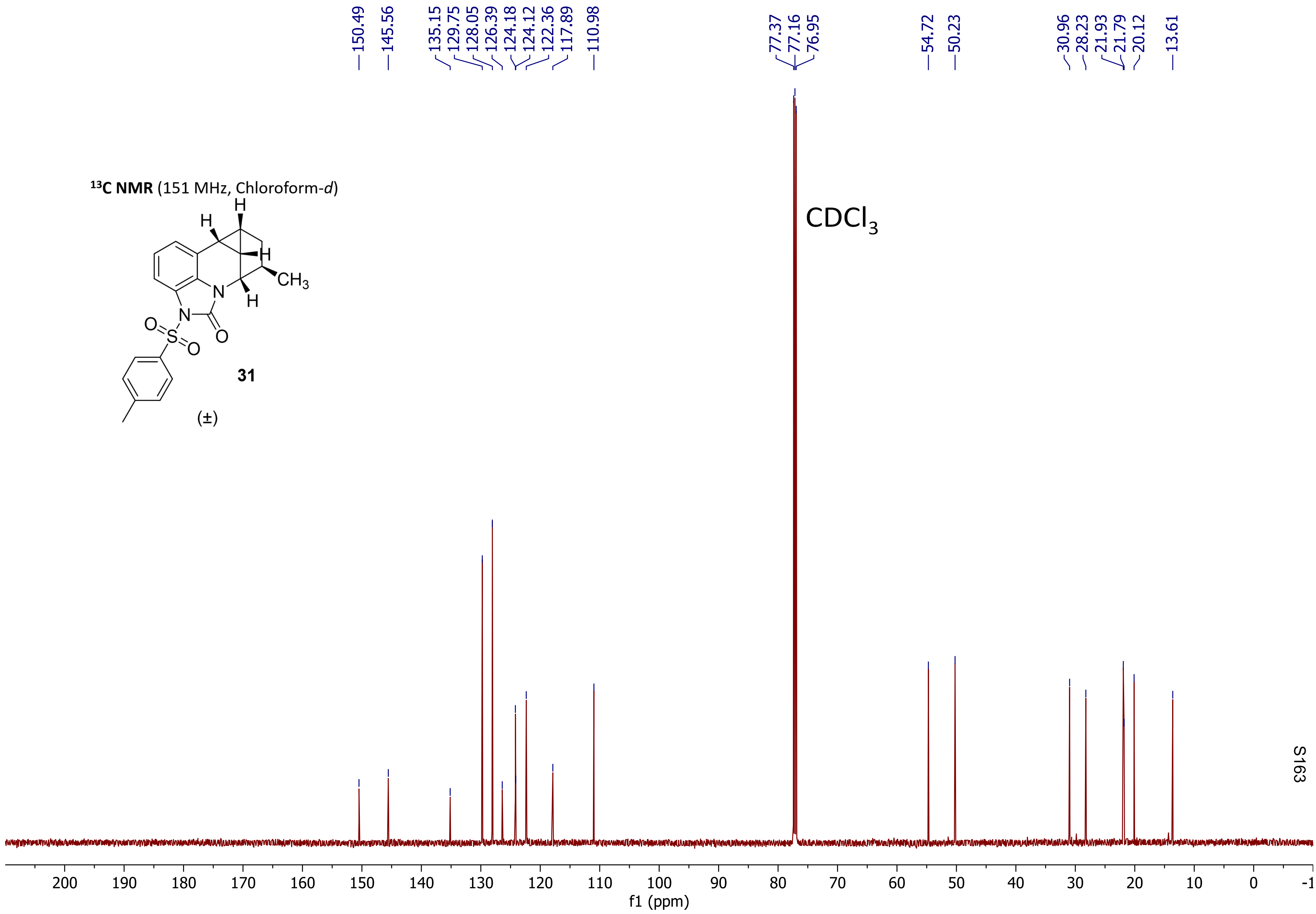

31

(±)

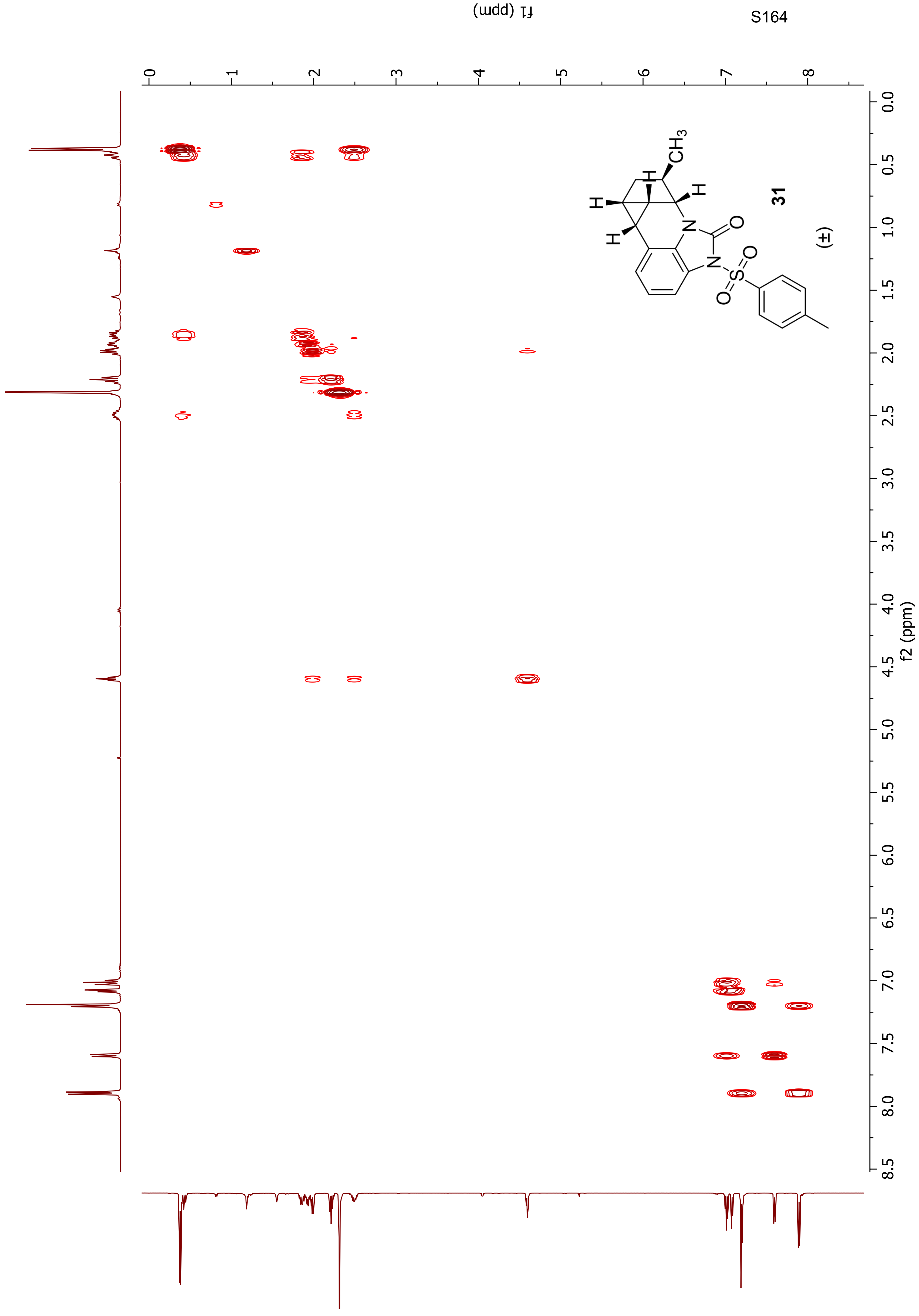

f1 (ppm)
f2 (ppm)
31
(±)

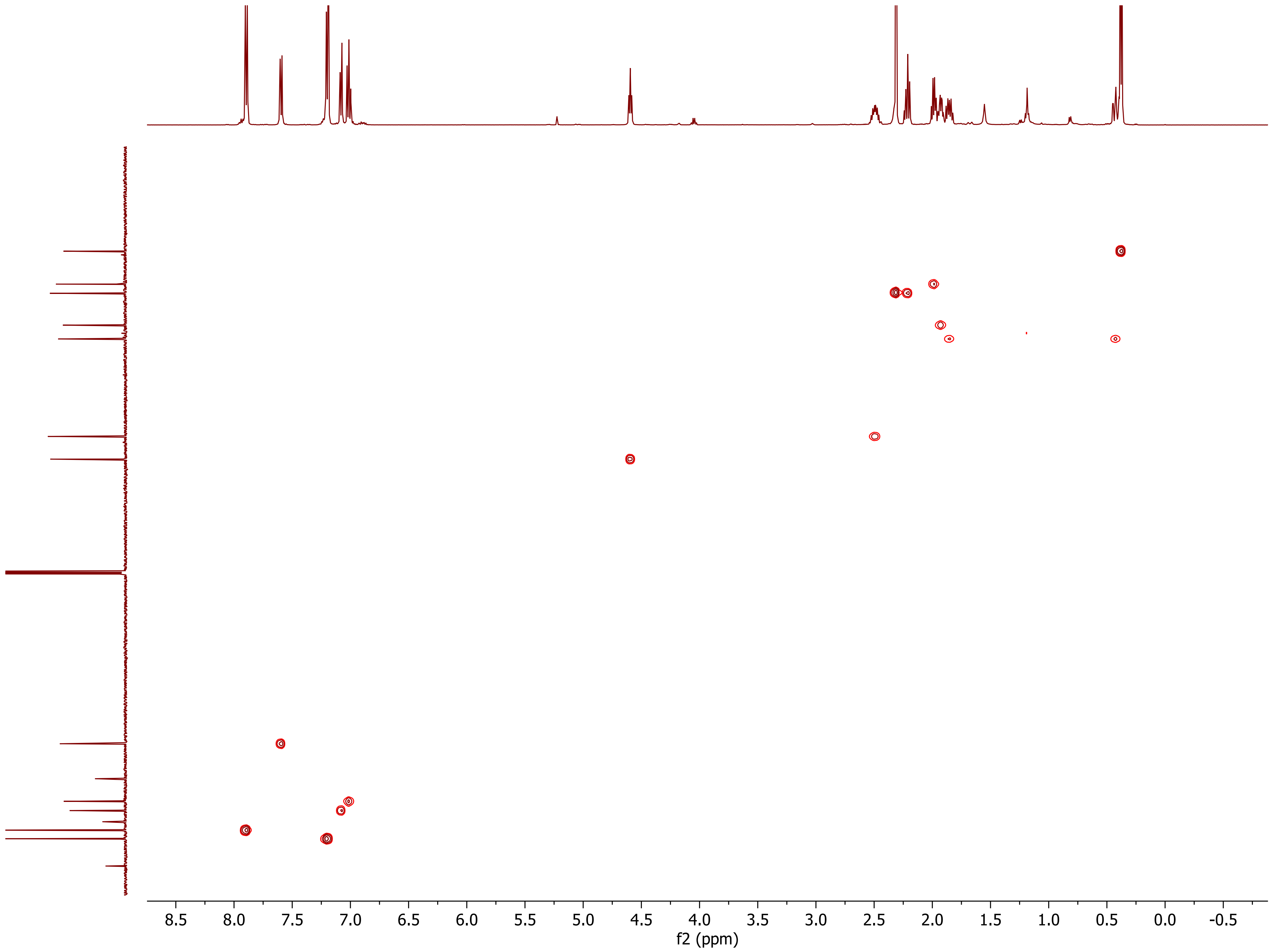
8.5
8.0
7.5
7.0
6.5
6.0
5.5
5.0
4.5
4.0
3.5
3.0
2.5
2.0
1.5
1.0
0.5
0.0
-0.5
f2 (ppm)
0
10
20
30
40
50
60
70
80
90
100
110
120
130
140
f1 (ppm)

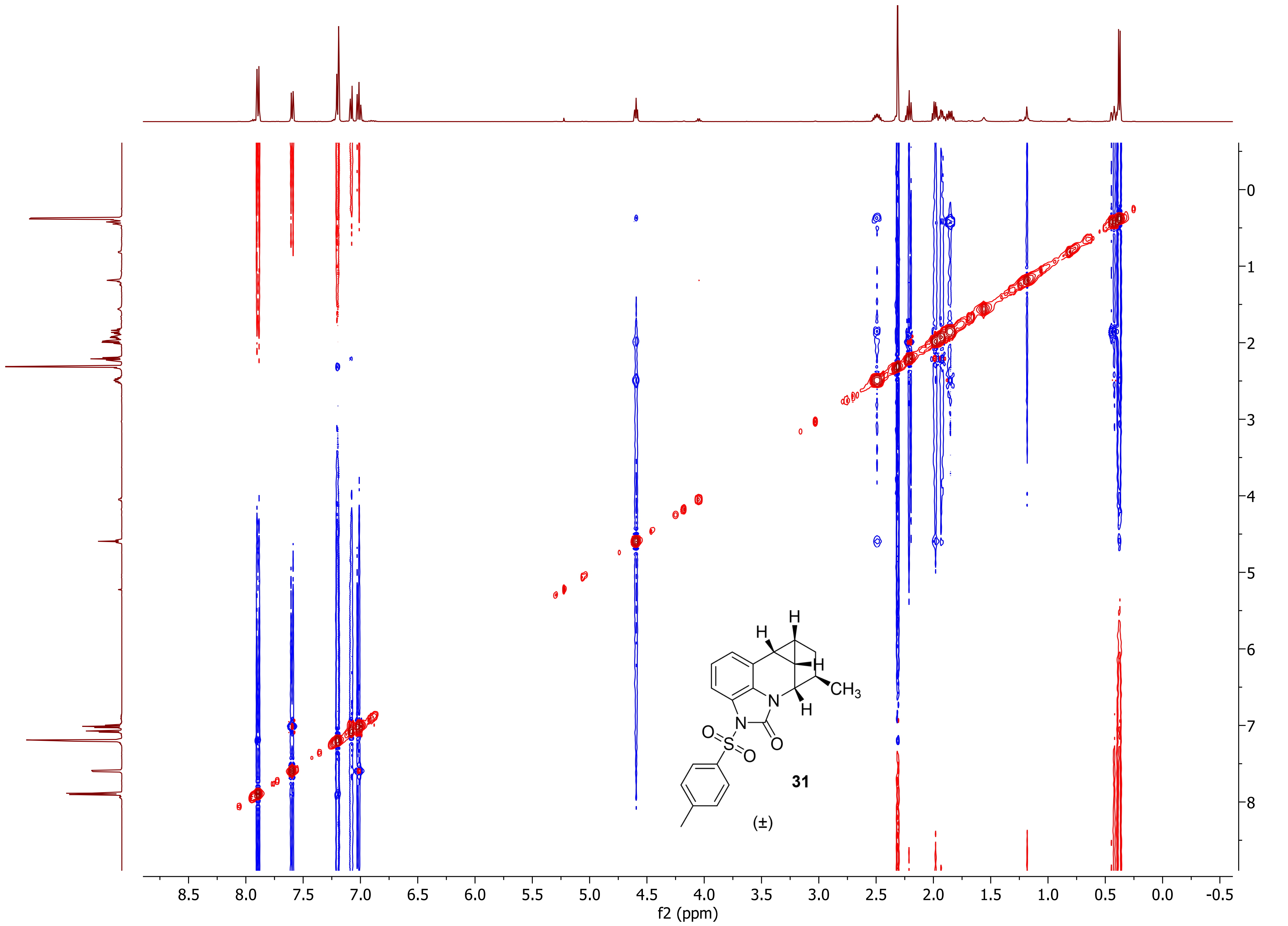
f2 (ppm)
f1 (ppm)
8.5
8.0
7.5
7.0
6.5
6.0
5.5
5.0
4.5
4.0
3.5
3.0
2.5
2.0
1.5
1.0
0.5
0.0
-0.5
0
1
2
3
4
5
6
7
8
H
H
H
H
CH_3
N
N
O
S
O
O

31

(±)

^{1}H NMR (500 MHz, Chloroform-*d*)

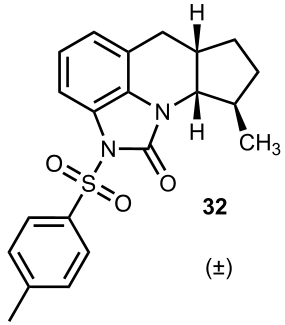

32

(±)

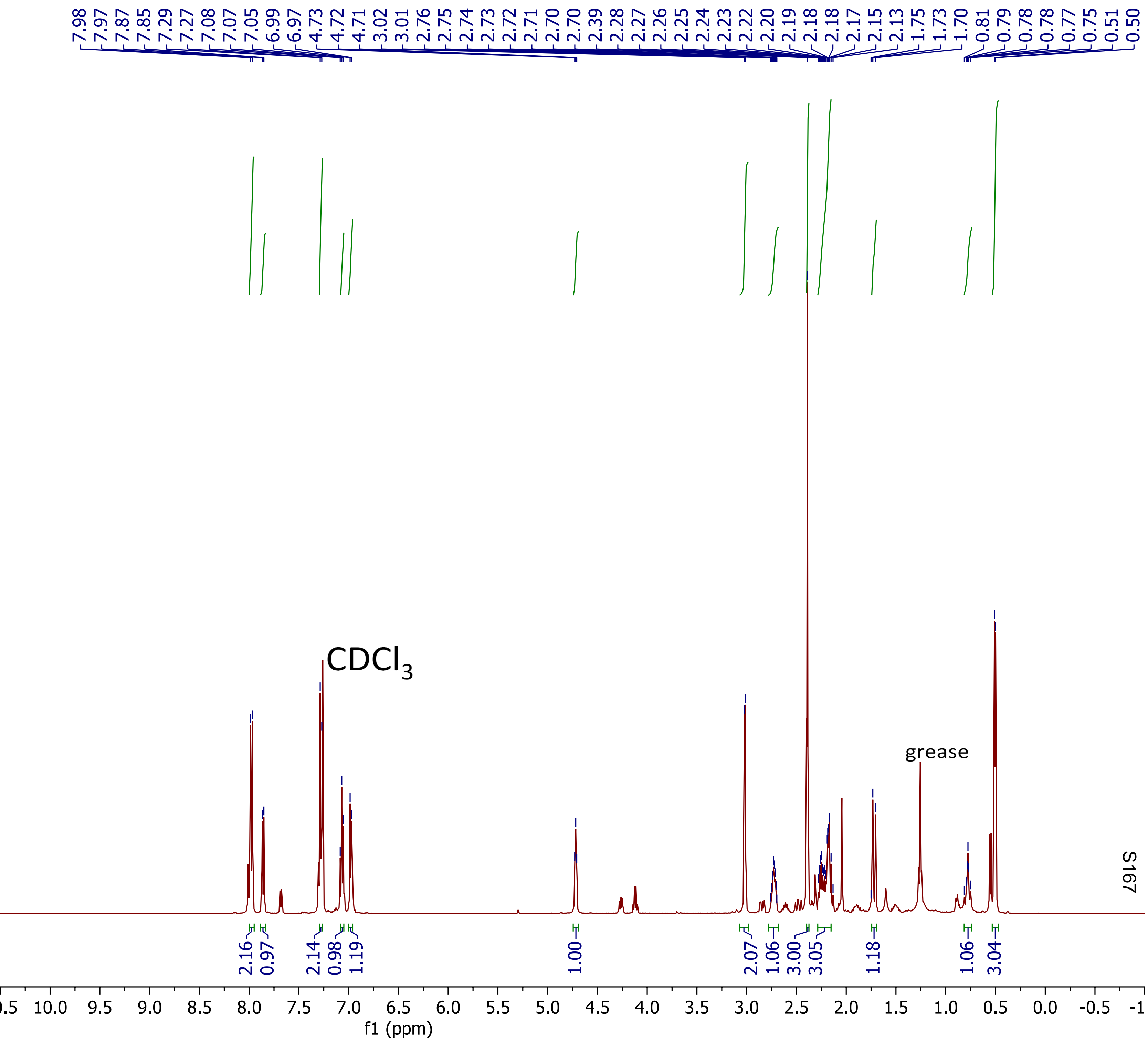

^{13}C NMR (151 MHz, Chloroform-*d*)

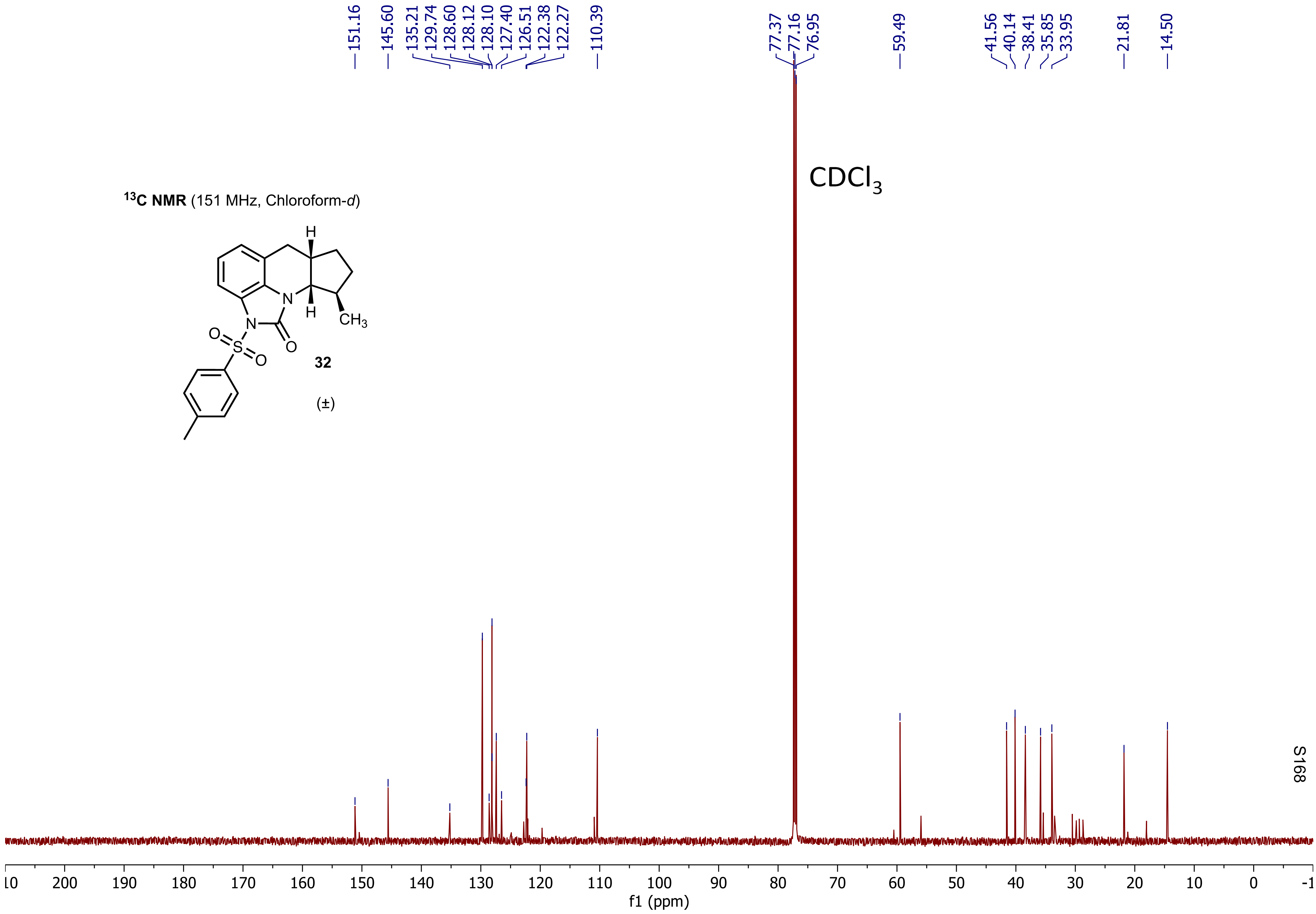

32

(±)

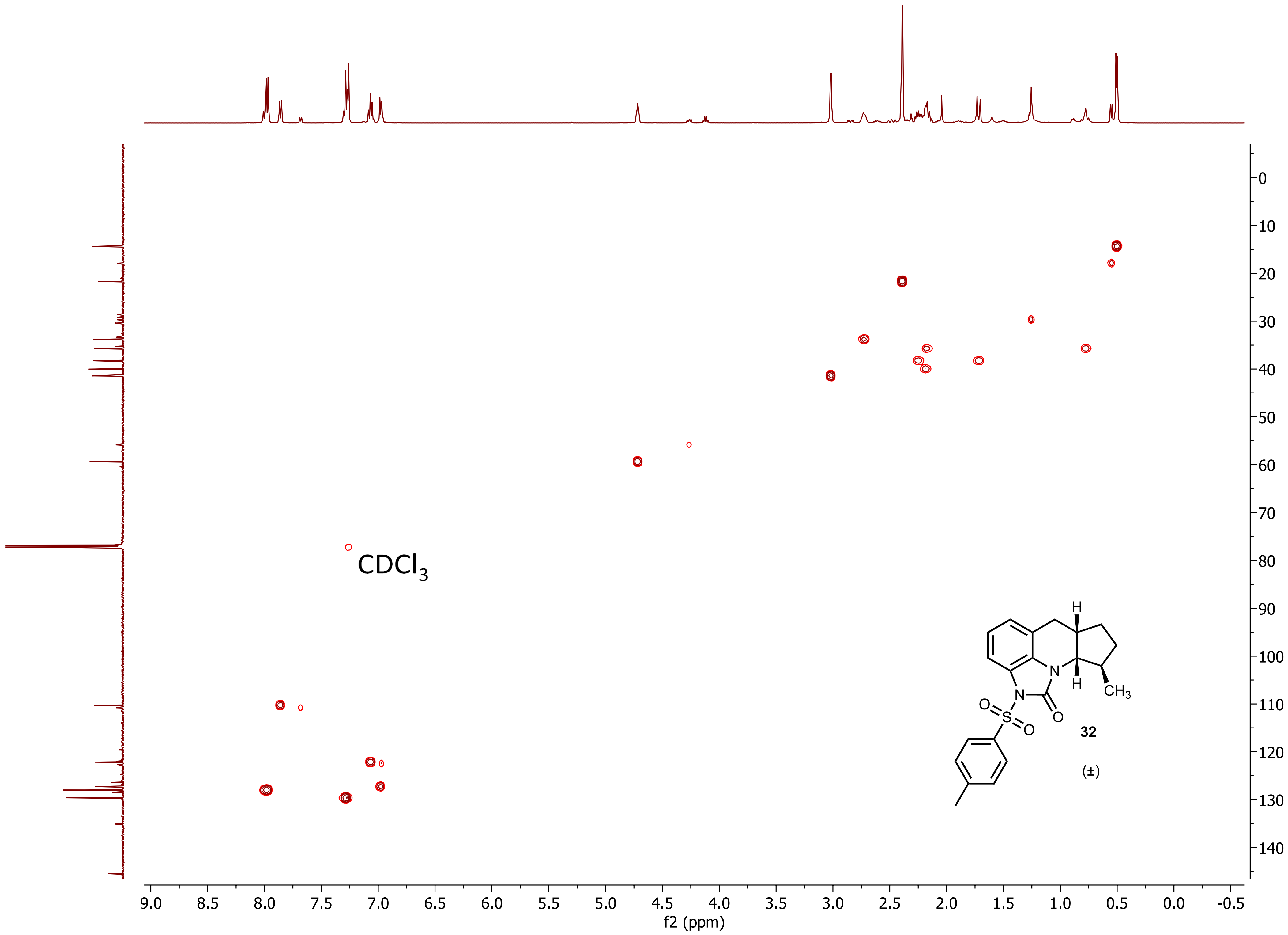
CDCl3
32
(±)
f2 (ppm)
f1 (ppm)

¹H NMR (400 MHz, Chloroform-*d*)

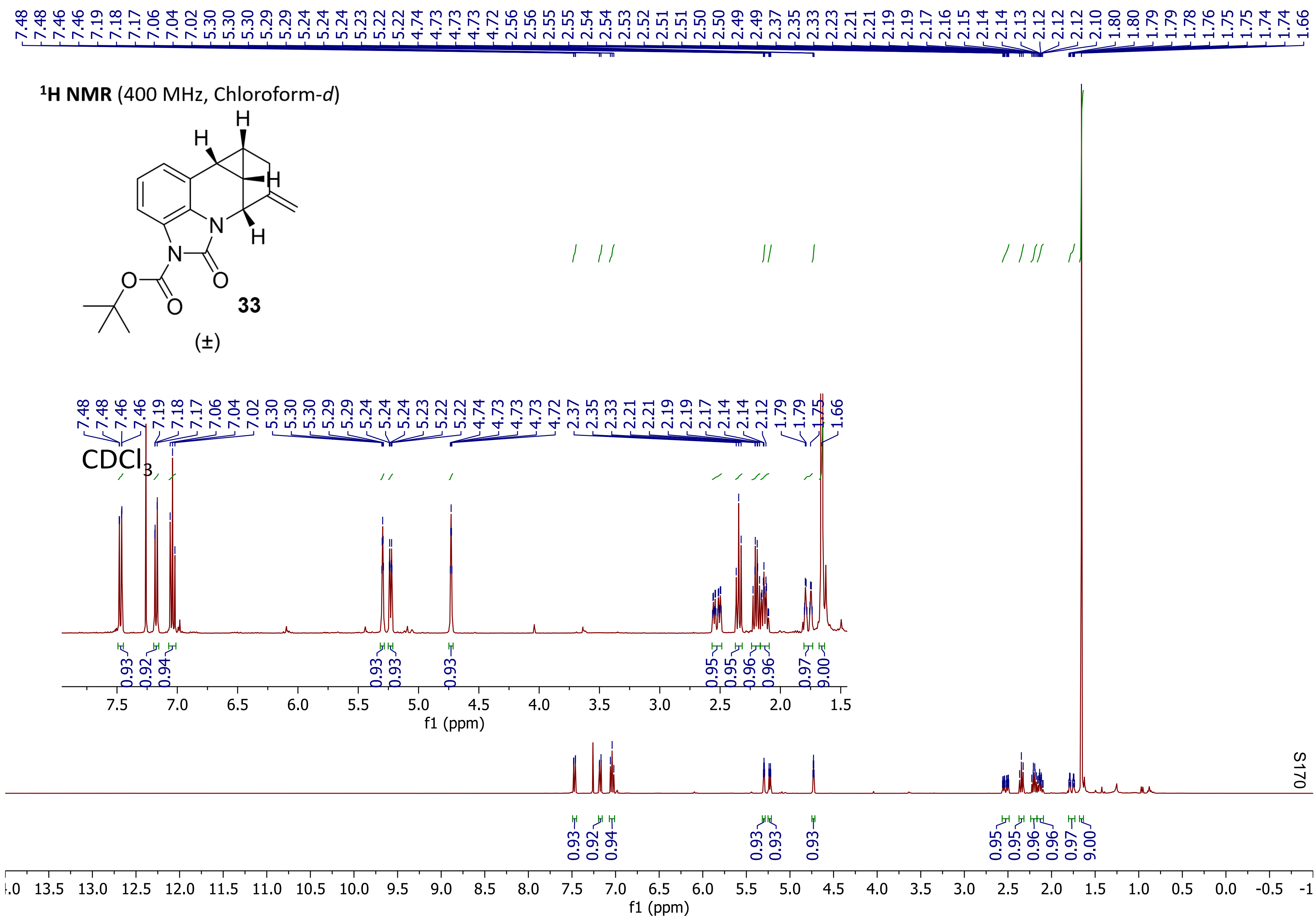

^{13}C NMR (126 MHz, Chloroform-*d*)

33

(±)

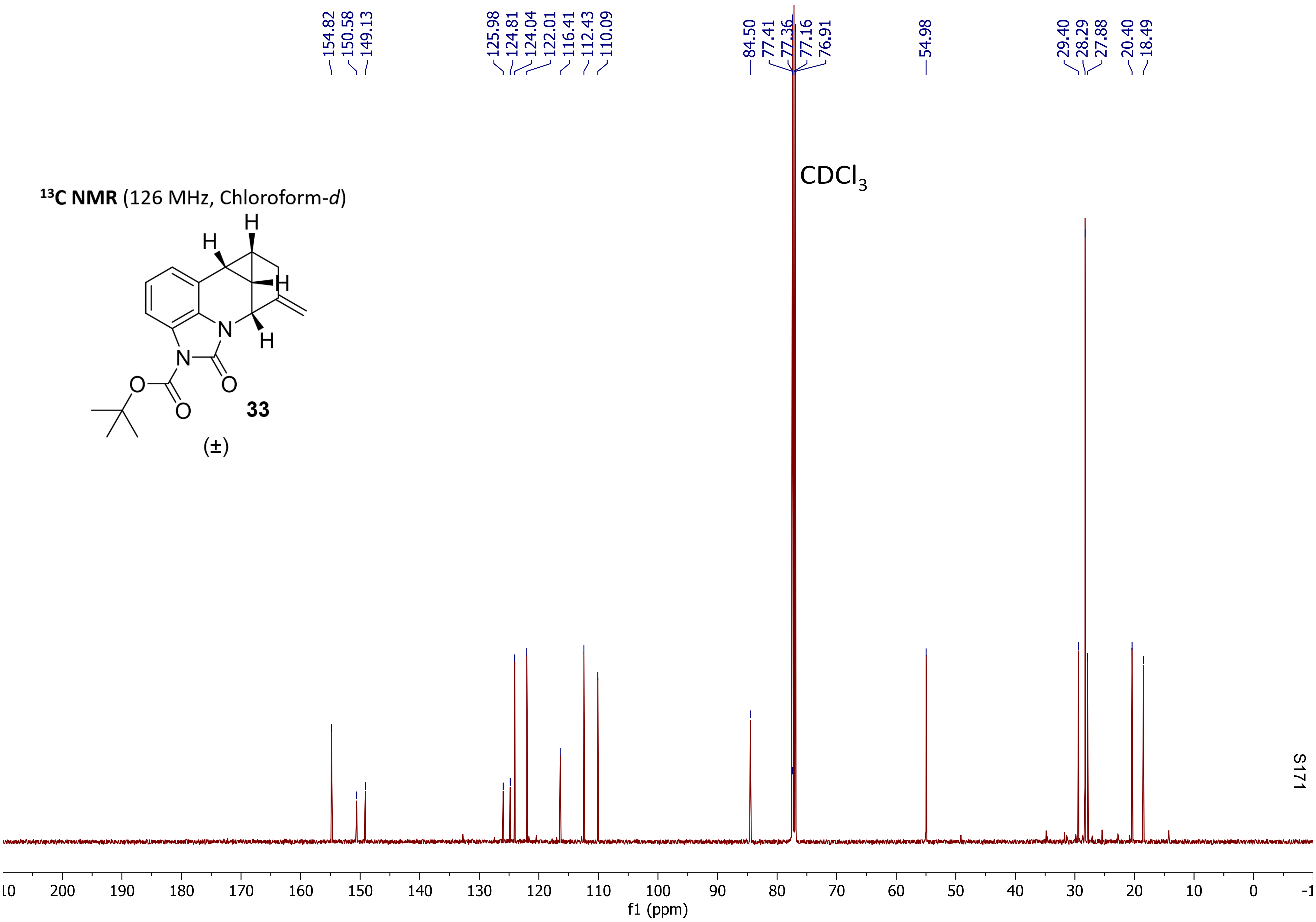

^{1}H NMR (601 MHz, Chloroform-*d*)

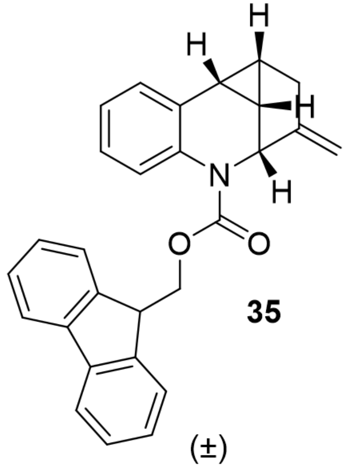

35

(±)

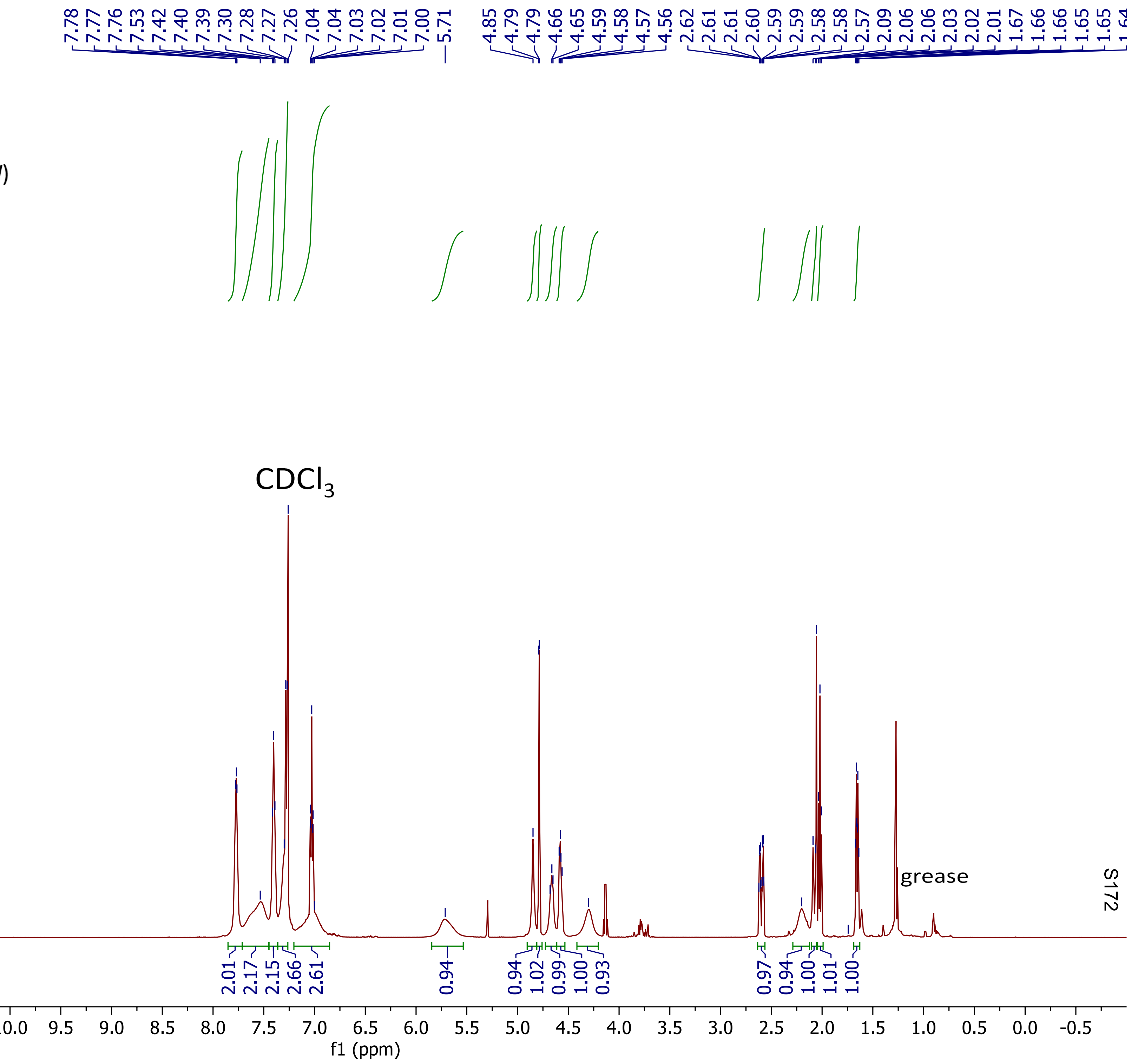

^{13}C NMR (151 MHz, Chloroform-*d*)

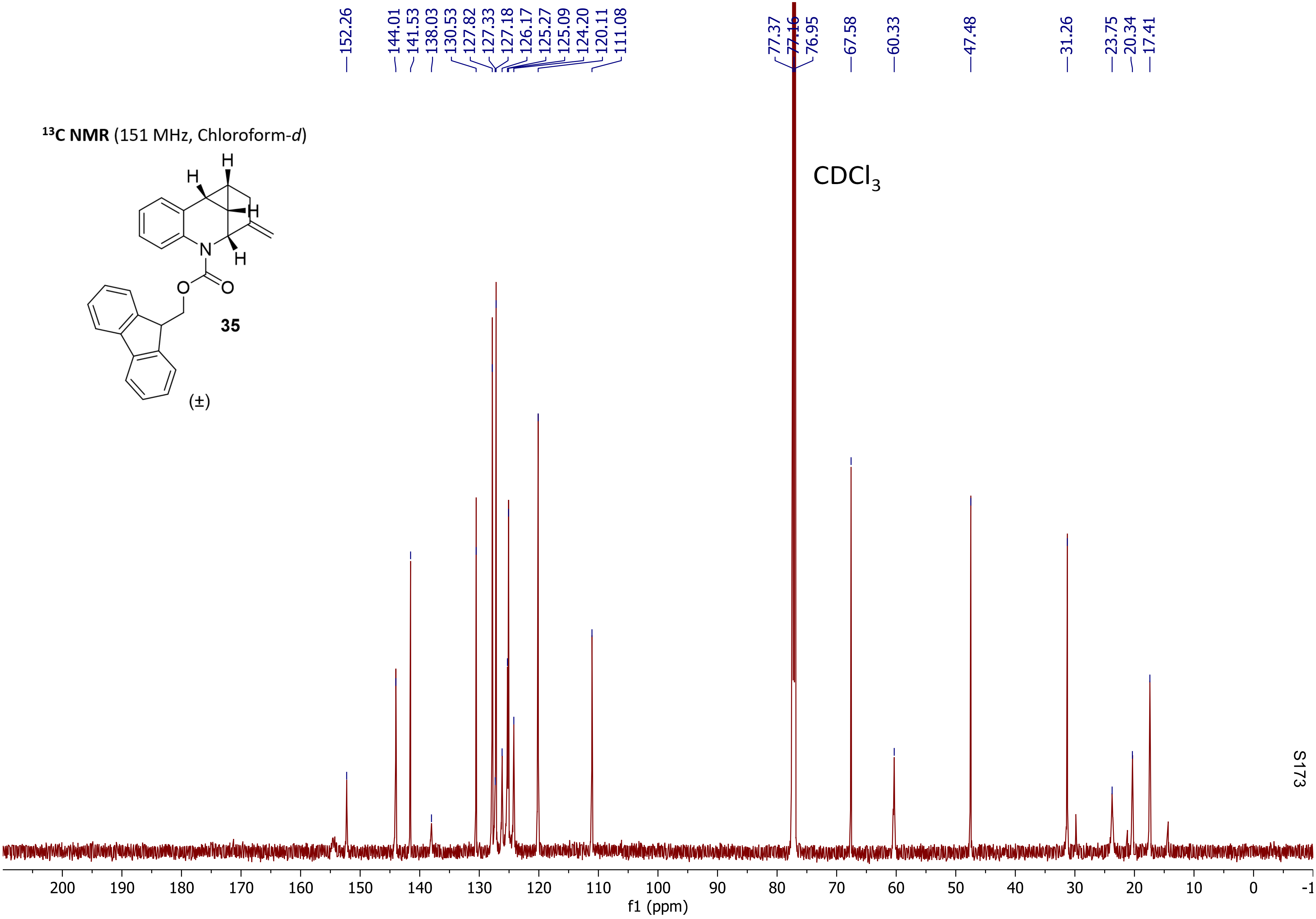

35

(±)

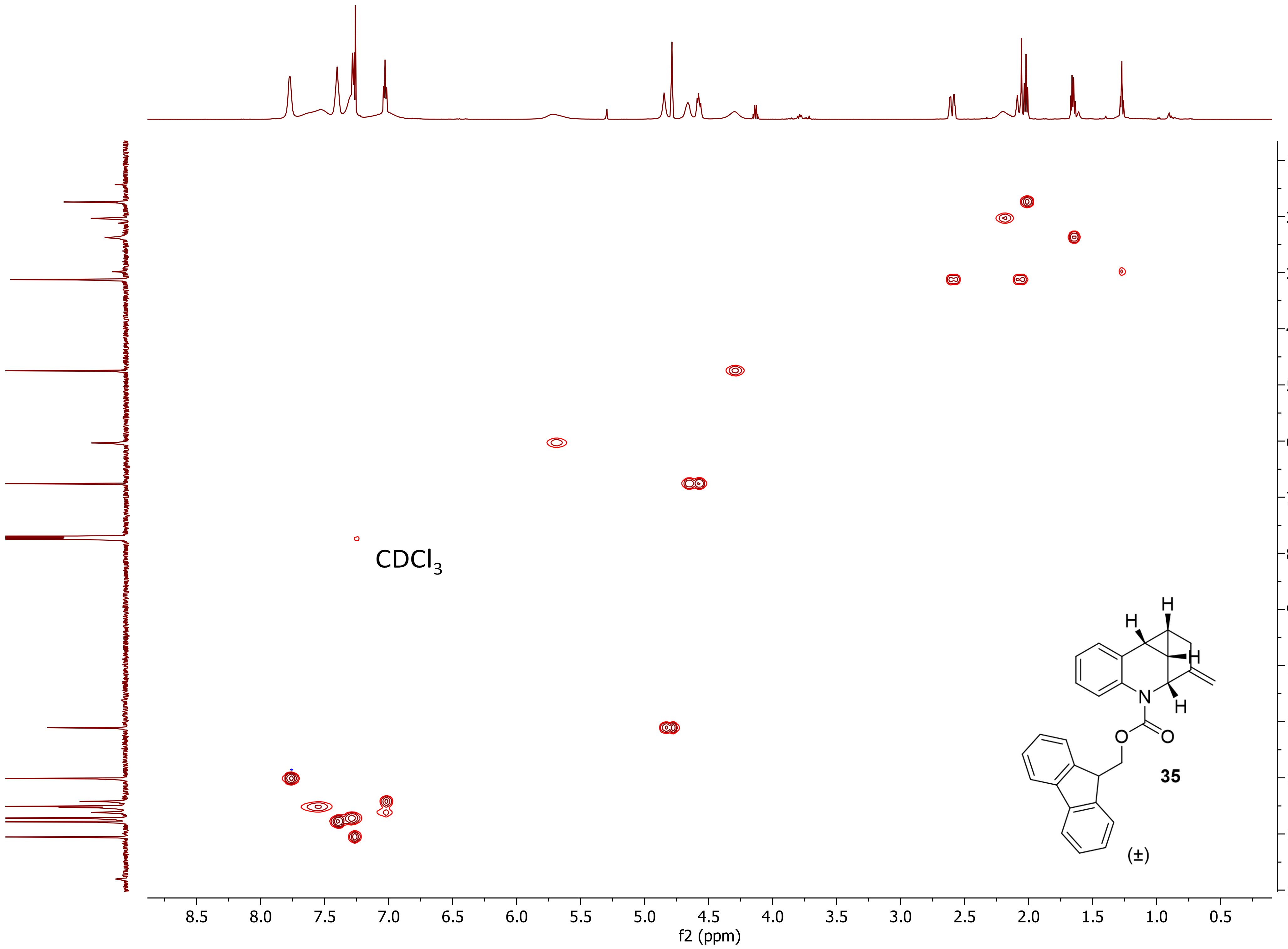
CDCl3
35
(±)
f2 (ppm)
f1 (ppm)
